# Structural Analysis of Variability and Interaction of the N-terminal of the Oncogenic Effector CagA of *Helicobacter pylori* with Phosphatidylserine

**DOI:** 10.3390/ijms19103273

**Published:** 2018-10-22

**Authors:** Cindy P. Ulloa-Guerrero, Maria del Pilar Delgado, Carlos A. Jaramillo

**Affiliations:** Laboratory of Molecular and Bioinformatic Diagnosis, Department of Biological Sciences, Universidad de los Andes, Bogotá 111711, Colombia; cp.ulloa530@uniandes.edu.co (C.P.U.-G.); cjaramil@uniandes.edu.co (C.A.J.)

**Keywords:** CagA, phosphatidylserine, phosphatidylserine mutations, conservation N-terminal CagA, homology modeling, molecular docking

## Abstract

*Helicobacter pylori* cytotoxin-associated gene A protein (CagA) has been associated with the increase in virulence and risk of cancer. It has been demonstrated that CagA’s translocation is dependent on its interaction with phosphatidylserine. We evaluated the variability of the N-terminal CagA in 127 sequences reported in NCBI, by referring to molecular interaction forces with the phosphatidylserine and the docking of three mutations chosen from variations in specific positions. The major sites of conservation of the residues involved in CagA–Phosphatidylserine interaction were 617, 621 and 626 which had no amino acid variation. Position 636 had the lowest conservation score; mutations in this position were evaluated to observe the differences in intermolecular forces for the CagA–Phosphatidylserine complex. We evaluated the docking of three mutations: K636A, K636R and K636N. The crystal and mutation models presented a ΔG of −8.919907, −8.665261, −8.701923, −8.515097 Kcal/mol, respectively, while mutations K636A, K636R, K636N and the crystal structure presented 0, 3, 4 and 1 H-bonds, respectively. Likewise, the bulk effect of the ΔG and amount of H-bonds was estimated in all of the docking models. The type of mutation affected both the ΔG ($\chi^{2}\left(1\right)=93.82$, *p*-value $<2.2\times 10^{-16}$) and the H-bonds ($\chi^{2}\left(1\right)=91.93$, *p*-value $<2.2\times 10^{-16}$). Overall, 76.9% of the strains that exhibit the K636N mutation produced a severe pathology. The average H-bond count diminished when comparing the mutations with the crystal structure of all the docking models, which means that other molecular forces are involved in the CagA–Phosphatidylserine complex interaction.

## 1. Introduction

*Helicobacter pylori* is a bacteria that colonizes and infects the digestive tract and is found in approximately 50% of the population [1]. Among the pathologies it causes are: gastritis, peptic ulcers, adenocarcinomas and mucosa-associated lymphoid tissue (MALT) lymphoma [2]. Nevertheless, only 1–5% of infected individuals has one of these severe gastric diseases [3,4]. Infection due to *H. pylori* has been recognized as an important risk factor for gastric cancer [5]. According to epidemiological data, 60–90% cases of gastric cancer can be attributed to the presence of this microorganism [6]. The relative risk of acquiring this pathology increases if patients are infected by positive cytotoxin-associated gene A (CagA) strains [5]. Therefore, the World Health Organization has classified this pathogen as a type I carcinogen [7].

CagA gene is part of the 40 kb pathogenicity island (cag PAI) that codes for a type IV secretion system (T4SS). This system is responsible for the translocation of CagA inside of epithelial cells through its injection [2,5]. Once inside the cell, CagA can modify the signaling pathways depending of the presence [5,8,9,10,11,12,13] or absence [4,14,15,16,17,18,19,20] of phosphorylation [21].

Phosphorylation of CagA is done by the proto-oncogene tyrosine-protein kinase (Src) and the mammalian Abelson murine leukemia viral oncogene (c-Abl) of the host in the tyrosine residues of EPIYA motifs (Glu-Pro-Ile-Tyr-Ala) of the C-terminal region of the protein [3,22,23]. Eventually, all this allows for interactions with cellular proteins to appear, leading to elongation, migration and dispersion of cells. This is because these cells interfere with signaling pathways that control adhesion between cells, cellular growth and motility [3,22,23]. Additionally, independent phosphorylation pathways activate β-catenin, which leads to the disruption of the apical union complexes and the loss of cellular polarity [3,12,22,23]. CagA can also form dimers in cells independent of phosphorylation [24]. Dimerization is mediated by a multimerization sequence (CM), which is essential for the union of CagA with the protease-activated receptor 1 (PAR1) complex (MARK: microtubule affinity-regulating kinase). Dimerization inhibits the kinase activity of PAR1 promoting the loss of cellular polarity [24]. Likewise, CagA can unite with tyrosine-protein kinase Met (c-Met). This union leads to an increase in the β-catenin regulation and the nuclear factor κB (NFκB); the former promotes proliferation and the latter inflammation [24]. Crystallographic studies have only allowed for the description of the N-terminal structure because the C-terminal is an intrinsically disordered region with versatile folds [16,25]. The N-terminal section possesses three domains: Domain I is composed by 10 α helices, has a small interaction area (374 Å${}^{2}$) and is structurally isolated from the other domains [25]. Domain II has a singular extended layer of β sheets and two helicoidal subdomains of α helices. Domain III is composed of four α helices that form a complex with the C-terminal [25].

The oncogenic effector CagA is an important virulence factor of *H. pylori* and it is strongly associated with the severity of the pathology [26,27,28,29,30,31]. In several studies, the number and type of EPIYA have been associated with this pathology; nevertheless, in Colombia and elsewhere, a direct association has not been observed [32,33,34]. Generally, the interactions with motifs located in the C-terminal have been studied. Nonetheless, little attention has been given to the possible role that the N-terminal may play in the observed pathophysiology.

Generally, signal induction of CagA in host cells happens because of its interaction with the motifs located in the C-terminal. However, the N-terminal might also play an important role in this pathophysiology. Bagnolli et al. demonstrated that the N-terminal of CagA helps to direct the proteins to the plasmatic membrane of epithelial cells independent of the C-terminal [22]. In addition, Pelz et al. proved that CagA consists of two independent domains (C-terminal and N-terminal) that interact with membrane structures of host cells [5]. The first 200 amino acids of the N-terminal act as an inhibitory domain to cellular responses evoked by the C-terminal [5]. The N-terminal increases the rate and strength of the newly formed contacts between cells, diminishes cellular elongation and the construction of the apical membrane induced by the C-terminal, and reduces the transcription activity of the transcription factor (TCF)/β catenin of the C-terminal. The former mediates the cell to cell adhesion by the E-cadherin-β catenin complex [5,35]. All this suggests that the N-terminal and the C-terminal also interact, and therefore influence the cellular response presented during the infection. Hence, the N-terminal is involved in the attachment to the cell membrane. From this, we can conclude that it is responsible for the localization of CagA in the phospholipid bilayer.

The medium domain presents a union site for the α5β1 integrin necessary for the delivery of CagA in epithelial gastric cells [16]. Additionally, it possesses a positively charged region that is important in the union with the membrane, especially with phosphatidylserine (PS) [17,25]. Murata-Kamiya et al. [17] reported that this union generates a rapid and transitory externalization of the PS, independent of apoptosis, in the bacterial union site [16]. The union of CagA with PS does not happen with domains that unite phospholipids, but with the Lys-Xn-Arg-X-Arg (K-Xn-R-X-R) motif encountered in the N-terminal region of CagA [16]. The electrostatic interaction between the negative charge of the PS and the positive charge of the lysine and arginine residues of the K-Xn-R-X-R motif are highly conserved on the binding region of acidic phospholipids such as PS [36]. Specifically, two arginine residues (R619 and R621) are conserved in Western strains of *H. pylori* [26695, G27, J99] and the F75 strain from East Asia [16]. Through the in-vitro preparation of CagA mutants, it was found that the residues K613, K614, K617, K621, R624, R626, K631, K635 and K636 are involved in the CagA–PS interaction [25]. In this interaction, CagA uses different association mechanisms depending on the polarity status of the epithelial cell. In a polar epithelial cell, CagA is distributed selectively to the inner face of the plasmatic membrane, initiating the disruption of stretch unions, causing epithelial apico-basal polarity losses and inhibiting the kinase activity of PAR1 through the physical formation of a complex [16,22]. In addition, in non-polarized cells, CagA is located in the membrane through a mechanism dependent on the EPIYA motif of the C-terminal [10].

CagA uses PS as a receptor, which allows it to enter the cell [17]. The CagA–PS interaction plays an important role in mediating the delivery, intracellular location and pathophysiologic action of CagA, which causes deregulation of several pathways that might eventually lead to cancerous’ cells formation [17].

Computational analysis, such as Docking assays, has allowed for the observation and quantification of interactions between different types of biomolecules. Working with proteins, specifically has been a great challenge since they have complex interactions at the atomic scale and because docking experiments require complete resolved structures [37]. Until now, computational models to obtain the entire molecular structure of proteins are limited. Therefore, techniques such as X-ray crystallography are essential [38]. Approximately 87% of all structures stored in Protein Databank (PDB) are obtained using this approach [39]. An extremely large and growing list of docking software is available (a comprehensive list is being kept up to date on the click2drug web portal, http://www.click2drug.org). Among them, SwissDock presents a graphical and a command line interface, which not only prepares the molecules, but also docks proteins with small molecules, such as the interaction between CagA and PS. Additionally, this server provides manually curated protein structures, or it receives PDB files that can be prepared through ad hoc scripts [37]. The structure of the ligand can also be selected directly from the ZINC database. SwissDock is based on the docking software EADock DSS [40]. First, a large number of binding models are generated in the vicinity of the target cavities of the entire protein. Conversely, a user-defined box can also be implemented. Simultaneously, CHARMM [41] energies are estimated on a grid. They are then calculated considering the solvent effect using the Fast Analytical Continuum Treatment of Solvation (FACTS) model [42]. Finally, models are ranked and clustered using the previous information.

In this study, the variability of the amino terminal of the oncogenic effector CagA was determined by referring to molecular interaction forces with the PS. This was done by: (i) evaluating the variability of CagA in the amino terminal region; (ii) determining the possible amino acid variation in the CagA specific positions that interact with the PS; (iii) assessing three mutations (K636A, K636R, and K636N) that were chosen from variations in specific positions shown by the multiple sequence alignment (MSA); and (iv) calculating the intermolecular interaction strength (free energy and Hydrogen bonds) from the chosen mutations. Finally, an association between the amino acid variability in position 636 and the severity of the pathology was found.

## 2. Results

### 2.1. Sequence Selection and Multiple Sequence Alignment (MSA)

Out of the 191 different sequences found in the NCBI search, only 127 met all the criteria (pathology, isolation region and type of EPIYA). These sequences where translated to amino acids and aligned using different methods. These alignment methods were required due to the sensitivity in the Consurf analysis; the calculation of the conservation scores was highly affected by major gaps in the sequences. This was improved using an alignment refinement (for details, see Section 4.3). To select the best alignment, the AQUA pipeline was implemented, and the highest NorMD score (NorMD = 1) was used as a reference. As a result, the MSA with the highest NorMD score was used for the Consurf analysis including all 127 sequences, even though two sequences from this MSA (88 and 94) generated gaps in the alignment.

### 2.2. Consurf Analysis

**Figure 1:**
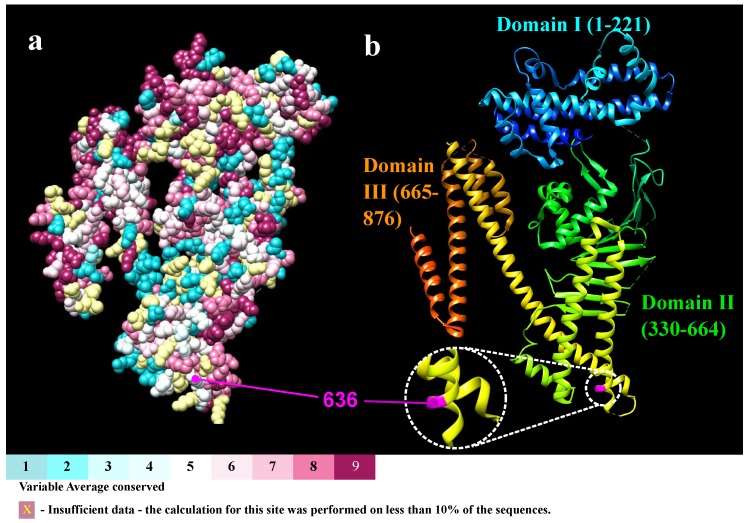
Consurf Results obtained from AQUA MSA: (**a**) amino acids with conservation scores of CagA indicating the location of residue 636; and (**b**) ribbon view of each domain is specified showing the location of residue 636 in magenta.

The major sites of conservation found in CagA basically correspond to alpha helices of all the three domains. In these areas, the conservation scores varied from 5 to 9. Domain I has helices α1, α2 and α7 in the approximate positions 29–38, 45–121 and 157–172, respectively; domain II has the middle helices α13, α14 and α18; and Domain III has the initial helices α19 and α23 (Figure 1).

**Figure 2:**
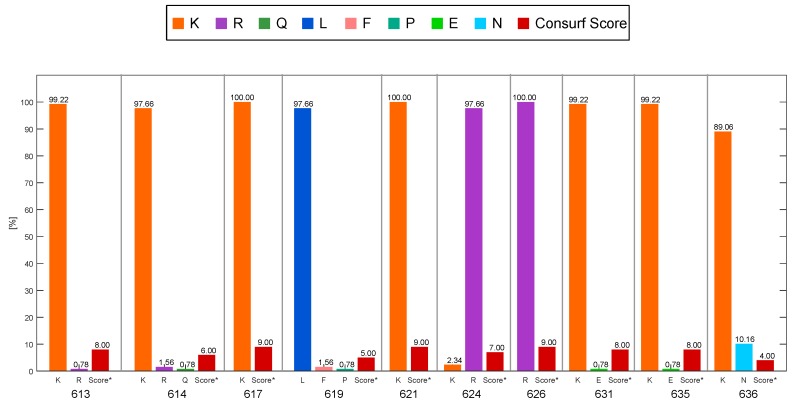
Percentage of Amino Acid Variation in CagA. These results were obtained from the Consurf platform. Amino acids in position 617, 621 and 626 had the highest conservation score, while the amino acid 636 had the lowest conservation score.

Helix α18 in Domain II is the specific region of interaction between CagA and PS. There is a positive patch that attaches in a Velcro-like way to the negatively charged PS found in the plasmatic membrane [25]. Most of the residues involved in this interaction are highly conserved with scores of 7–9 (Figure 2). In this region, the most conserved positions were shown to be 617, 621, and 626 which all had a score of 9 and a lysine/arginine amino acid, which is consistent with the findings by Roujeinikova [14].

Nevertheless, there were only two positions in which the score was below 5: 619 and 636. These results suggest that some positions may have considerable variability where an amino acid change could alter the interaction forces of the CagA–PS complex. The amino acid in position 619 had an unreliable result since it had excessively large confidence intervals [43]. Therefore, position 636 was used to create mutations in the crystal.

We evaluated three different mutations of the crystal (4DVY): K636A, K636N and K636R. The natural variation of lysine to asparagine was assessed in one of the mutations (K636N). The other two mutations were chosen in order to evaluate the reliability of the data obtained from the different dockings. The arginine mutation was used as a positive control since it belongs to the same group as the amino acid lysine in which the R groups are positively charged. On the other hand, the alanine mutation was evaluated as a negative control as it belongs to a non-polar amino acid. For each of the mutations, 256 different binding models were acquired. Nonetheless, only the models that interacted with the α18 were selected, resulting in 139 models. The data for each mutation evaluated were organized per cluster; each cluster represented different locations of the interaction between PS and the CagA protein; each datum of the cluster assessed different rotations of the PS molecule. The 139 resulting binding models corresponded to all the models within each cluster. For all models, the free energy (ΔG) and hydrogen bonds were obtained. The best model from each mutation was determined using the following criteria: the highest ΔG and number of hydrogen bonds.

### 2.3. Docking of All Mutations

**Figure 3:**
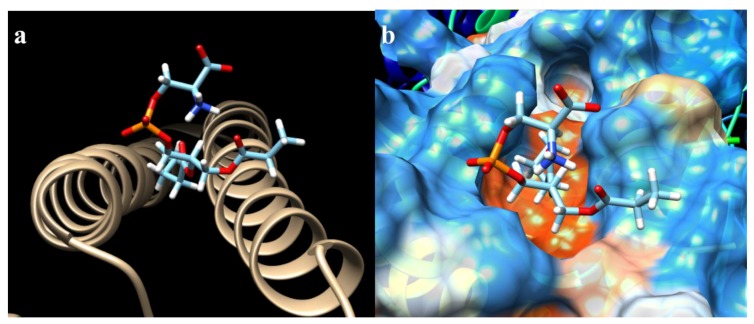
CagA–PS interaction of the mutation K636A obtained from Chimera: (**a**) hydrogen bonds were not found for this particular binding model; (**b**) hydrophobic surface view of CagA, color-coded from dodger blue for the most hydrophilic, to white, to orange-red for the most hydrophobic.

**Figure 4:**
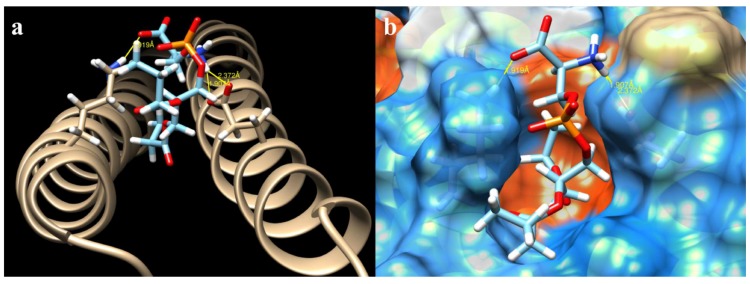
CagA–PS interaction of the mutation K636R obtained from Chimera: (**a**) hydrogen bonds and their distance (yellow lines) of interacting atoms are shown for both molecules; and (**b**) hydrophobic surface view of CagA, color-coded from dodger blue for the most hydrophilic, to white, to orange-red for the most hydrophobic.

**Figure 5:**
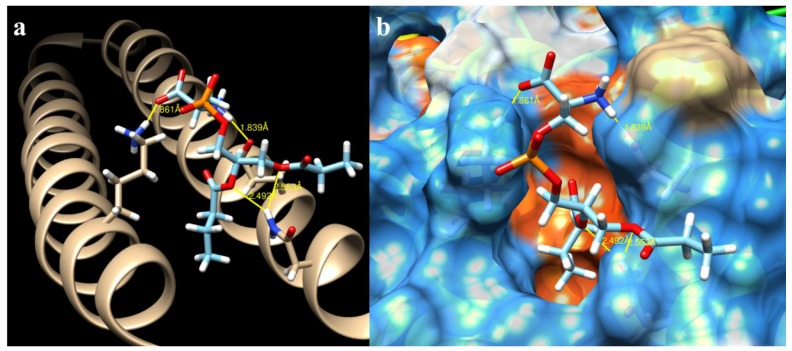
CagA–PS interaction of the mutation K636N obtained from Chimera: (**a**) hydrogen bonds and their distance (yellow lines) of interacting atoms are shown for both molecules; and (**b**) kydrophobic surface view of CagA, color-coded from dodger blue for the most hydrophilic, to white, to orange-red for the most hydrophobic.

**Figure 6:**
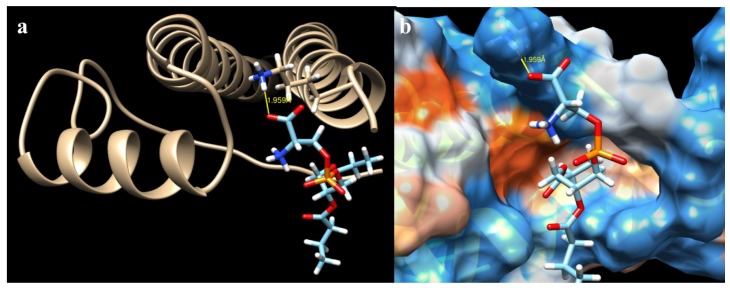
CagA–PS interaction of the 4DVY crystal obtained from Chimera: (**a**) hydrogen bonds and their distance (yellow lines) of interacting atoms are shown for both molecules; and (**b**) hydrophobic surface view of CagA, color-coded from dodger blue for the most hydrophilic, to white, to orange-red for the most hydrophobic.

The ΔG for all the mutations varied within a very small range (Table 1). The highest value obtained was from the crystal (−8.919907 Kcal/mol), as was expected. The lowest free energy value was from the mutation K636N (−8.515097 Kcal/mol). The number of hydrogen bonds had the inverse effect of ΔG. While mutations K636A, K636R, and K636N presented 0 (Figure 3), 3 (Figure 4), and 4 (Figure 5) hydrogen bonds, the crystal structure presented one bond of this type (Figure 6). Moreover, in each of the different interactions evaluated, there was a hydrophobic interaction pocket between the CagA and the PS surrounded by highly polar interactions. Hydrogen bonds were formed in the polar regions (Figure 3, Figure 4, Figure 5 and Figure 6).

The change of a lysine to asparagine at position 636 created two different hydrogen bridges between CagA and PS. This suggests an increase of the interactive force in the presence of this specific mutation (Figure 6). The asparagine is a non-polar amino acid that provides the same hydrogen bond donor and acceptor count as lysine [44], but it is a smaller residue that allows a more favorable interaction with the negatively charged membrane surface.

**Figure 7:**
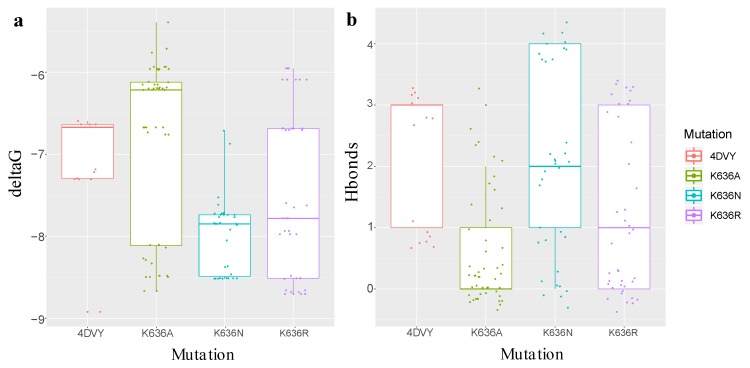
Boxplot comparing overall the Docking results: (**a**) ΔG values (kcal/mol) for each mutation; and (**b**) Hydrogen Count for each mutation. The median, interquartile range and maximum and minimum values are shown for each boxplot. Data points for each mutation are also shown.

Thus we proceeded to evaluate the sequences with this specific mutation in the entire database. We found that 13 of the 127 individuals (10.24%) had the K636N mutation, and 10 of those 13 sequences presented a severe pathology (76.9%) (Table 2). The aforementioned result suggests that the increase in interaction may cause a higher degree of pathology. To evaluate the bulk effect of the ΔG and the amount of hydrogen bonds on the entire docking model for each mutation (Figure 7), a likelihood ratio test comparing the linear mixed model (LMM) of these two response variables with a null model was performed. Wide error bars are observed when evaluating the ΔG and the quantity of hydrogen bonds. This is due to the high variance present in the data. We were aware that there was a difference in the sampling of each cluster per mutation (Table S2), and additionally, that the variance within each cluster was much smaller than the variance between cluster. This was acknowledged in the LMM generated (see Section 4.6 for details). The type of mutation affected both the free energy ($\chi^{2}\left(1\right)$ = 93.82, *p*-value $<2.2\times 10^{-16}$) and the hydrogen bonds ($\chi^{2}\left(1\right)$ = 91.93, *p*-value $<2.2\times 10^{-16}$). Mutation K636A diminished the ΔG by 1.105±0.20 Kcal/mol and the quantity of hydrogen bonds by 3.32±0.30. This matches the results obtained by Hayashi et al., in which a decrease in the interaction of co-immunoprecipitation assays was observed when comparing CagA–PS and CagA K636A mutation–PS [25]. Likewise, mutation K636R reduced the ΔG by 0.5729±0.16 Kcal/mol and the quantity of hydrogen bonds by 2.90±0.26 as was expected since the change of lysine to arginine, which are both positively charged amino acids, would have a smaller effect on the affinity of the interaction. However, the K636N mutation increased the ΔG by 0.6262±0.23 Kcal/mol and diminished the quantity of hydrogen bonds by 3.32±0.30. This means that the interaction increased the affinity of the molecule; however, the hydrogen bonding alone does not explain this conclusion. It may be that other types of interactions are playing an important role in the increase of free binding energy.

## 3. Discussion

Bacterial-borne effector protein CagA plays an essential role in pathogenic activity due to its tethering to the plasmatic membrane [25]. The translocation of the protein depends on the interaction interface of several regions with the phospholipid membrane [5,16,17,22,35]. PS is one of the phospholipids that composes the eukaryotic membrane and is characterized by having a negatively charged head group [45]. As PS is involved in a number of cellular signaling pathways where several molecules such as kinases, small GTPases and fusogenic proteins depend on this phospholipid to carry out their normal function. Its disruption would trigger a homeostatic imbalance and apoptotic interference [45].

Signaling is mediated by PS functions in two ways: either via domains that stereospecifically recognize the head group, or by electrostatic interactions with a negatively charged surface of membranes with rich PS and positively charged groups. In CagA, there is a positively charged helix α18 (residues 610–639) that has an exposed cluster of lysine/arginine residues at positions 613, 614, 617, 621, 624, 626, 631, 635 and 636 [17,25]. It is known that most of these residues of the positive patch of CagA–PS interaction are highly conserved between some *H. pylori* strains (26695, G27, J99, and F75) [16]. Recently, the similarity between the membrane tethering helices of CagA and eukaryotic F-BAR domains was revealed [14]. Since these domains are involved in the interaction with lipids, this would explain the interaction of CagA with lipid membranes of human epithelial cells. As was observed by their MSA, the positively charged patch of residues found on the lipid binding face in F-BAR domains are also present in CagA [14]. Therefore, mutations in these interacting areas change the pathogenesis of *H. pylori*, explicitly in the degree of the hummingbird phenotype observed in MDK cells [17]. Hence, we could assume that if we take all possible sequences of CagA into account and find the specific variations in the positively charged patch, variability in certain positions could explain the degree of pathology presented by a patient. This finding is important because of the lack of correlation between the number and type of EPIYA with the pathology found in some studies [32,33,34]. Thus, if we consider an additional factor, such as the variation and force of interaction in the N-terminal, we could predict the prognosis of a patient more accurately.

In this study, we found that major sites of conservation in CagA mainly correspond to alpha helices of all three domains. Residues in positions 617, 621 and 626 are highly conserved and have no amino acid variation, which is consistent with the results from Roujeinikova [14]. On the other hand, the position with the highest variability was 636; therefore, different mutations were performed in this position to evaluate how the amino acid change could alter the interaction forces of the complex CagA–PS. To test this hypothesis, the free energy and amount of hydrogen bonds were determined. We found that these values were comparable and had the same order of magnitude as in other studies involving computational and experimentally acquired ΔG and hydrogen bond count from interacting proteins with small ligands [46,47,48,49,50], thus validating our data.

Additionally, we showed that more than half of the sequences that exhibit the K636N mutation are linked to a severe pathology (76.9%). This may be due to the fact that there was an increase in the interacting forces shown by the generation of a higher, bulk-free binding energy and more hydrogen bonds in this specific model. However, when evaluating the entire model for each mutation, the average hydrogen count diminished as compared to the mutations with the crystal structure. This may be due to the increased variation of the different interacting clusters and the fact that there are other possible interactions that were not considered. In particular, the 636 residue in the CagA has a role in the electrostatic effect because the positively charged basic patch influences the strength of CagA binding to the negatively charged atoms in PS. One limitation of our study was that only the bulk free energy was considered. If other energies such as the degree of electrostatic interaction were calculated, this may have accounted for the discrepancies found with the amount of hydrogen bonds and the increase in the free energy.

To understand protein function and dynamics, a complete resolution of the protein is needed. It is important that the resolution at the atomic level of the 3D structures is accurate to obtain reliable results in the docking experiments. In CagA, the 4DVY crystal (PDB) has an adequate resolution based on structure refinement’s statistics. However, there may be some potential difficulties with the sidechain geometries that were not considered in this study. Furthermore, the parameters in the docking experiment also play a crucial role in the final results. In this experiment, the N-terminal of the CagA was modeled as a static macromolecule, which has to be carefully interpreted when analyzing the docking models. Flexibility of a protein allows for dynamic interactions with different molecules, from small ligands to other macromolecules. To evaluate this parameter, structural alphabets have been implemented, which analyze protein complexation, allostery and flexibility. These alphabets use information from the Debye–Waller factors, found in the X-ray crystallography experiments, which help identify conformational changes of domain motions and deformability of the protein’s backbone [51].

Moreover, studies have also suggested additional roles of the N-terminal CagA in the regulation and function of the entire protein. It is known that the N-terminal has a binding segment with the C-terminal that serves as a regulatory element. The interaction of the N-terminal and C-terminal enhances the localization of CagA via the positive patch, and strengthens the pathogenic scaffold/hub function of the protein [25]. This characteristic also accounts for the promiscuity of CagA as it promotes interaction with several host proteins [21,25,52]. The interaction of both segments allows for a determined, folded state of the protein that eventually leads to its oncogenic action. These effects were assessed evaluating the affinity of the interaction between the binding models of the entire CagA protein and two host proteins in two strains associated with severe and mild pathologies. The main differences in the interaction were due to energy contributions of the electrostatic energy, helix dipole energy, van der Waals clashes, torsional clash, backbone clash and cis bond energy; however, the bulk free energy did not play an essential role in describing the two strains. The strain that caused the severe pathology had the highest affinity regarding the previously described energies [53].

In addition, epithelial cells are not all the same; they display a different polarity status. CagA has different mechanisms to enter these cells depending on the degree of epithelial polarity. Polarized epithelial cells are rich in PS, so the CagA contains a binding motif to PS that initiates a disruption of tight junctions and causes loss of epithelial, apico-basal polarity by inhibiting kinase activity of PAR1 through physical complex formation [17,22]. In non-polarized epithelial cells, CagA situates itself in the plasma membrane through a C-terminal EPIYA motif in CagA [11]. These EPIYA motifs allow CagA to bind to several host cell proteins, a process which generates cellular elongation, migration and dispersion since these motifs interfere with signaling pathways involved in cellular adhesion, growth and motility [3,12,22,23].

Consequently, as mentioned above, CagA may include both pathways of N-terminal PS binding and EPIYA motif binding proteins via independent and dependent phosphorylation on polar and non-polar epithelial cells, respectively. Cellular disruptions caused by CagA give rise to the first steps in the transformation to neoplastic tissue. This tissue, in its transformed state, eventually causes carcinogenic gastric epithelial cells. Our study highlights the importance of considering the molecular forces for the CagA–PS interactions as this has implications for the pathogenic development and the consideration of several variables that influence the interaction of CagA with host cells. All of this could have important therapeutic implications for how *H. pylori* infections are handled in the future.

The study of molecular forces involved in the interaction of CagA binding with proteins in host cells helps us to have a better understanding of how it could cause different degrees of pathology. However, the development of different pathologies requires a multiple step process that involves several different variables of the N-terminal as well as the C-terminal. The interaction of CagA with PS requires a set of positively charged residues that are highly conserved among the sequences analyzed. The most variable position naturally found was the K636N mutation, which generated a higher free energy change and a lower hydrogen count with respect to the crystal structure 4DVY when considering all docking models. Nevertheless, the amount of hydrogen bonds increased when comparing the best model of all the mutations; specifically, when there was a lysine to asparagine change, it generated two additional hydrogen bonds. In approximately 80% of the cases observed with this mutation, a severe pathology was also found, which could indicate that other molecular forces are involved in the CagA–PS complex interaction that were not considered.That said, we acknowledge that this study has limitations regarding: the use of the free energy alone for the CagA–PS interaction; the lack of flexibility of the protein during the docking; potential difficulties in the geometry of the side chains; and the reduction in data by filtering of the models. For future studies, it is important to include other types of intermolecular interactions to evaluate the bulk effect of the affinity between CagA and PS, and to throughly assess how the geometry of the sidechains and the variability in the flexibility of the target and ligand may affect the observed interactions and/or run molecular dynamic simulations. Similarly, to have a complete model of how CagA affects the final pathology, both the N-terminal and C-terminal interactions must be considered. In this study, only the N-terminal was assessed due to limitations of the existing crystallographic structure on which all the evaluated results depended.

## 4. Material and Methods

### 4.1. Database

The National Center for Biotechnology Information (NCBI) was consulted for all the DNA sequences of the complete segment of cagA under “*Helicobacter pylori* CagA complete cds” with data up to January 2014 as the criteria. Sequences that presented information about the pathology, the region from which the sample was taken and EPIYA type were selected for the database construction. All samples from America and Europe were classified as Western and those from East Asian countries were labeled Eastern.

### 4.2. Translation of Gene Bank DNA Sequences

The translation from the DNA sequences to amino acids was performed by the AASA (Amino acid Sequence Analyzer) program [34] using an open reading frame that codified for the complete protein. The C-terminal was eliminated from each sequence after the 877th amino acid due to the size of the 4DVY crystal of 876 amino acids [25].

### 4.3. Multiple Sequence Alignment (MSA) of Translated Sequences and Quality Assessment

All sequences were aligned with the 4DVY crystal with MUSCLE [54], T-COFFEE [55] and MAFFT [56]. Residues from the sequences that presented amino acids before the initiation residue (methionine) were eliminated. RASCAL (v1.34) [57] was used to refine and improve all MSA. After this refinement process, the NorMD score was calculated using NORMD (v1.3) [58]. This score compared the alignment quality by identifying the “best” alignments as the ones with highest NorMD values, i.e., with a threshold value above 0.6 [57]. All of these programs were used within Automated Quality Improvement for Multiple Sequence alignments (AQUA) pipeline [59]. Default parameters were used in all alignments.

### 4.4. Conservation Level and Variability of the Interaction Residues with PS

Using the purified MSA, the Consurf [43] platform was used to determine the conservation level of the protein. From the alignment results, we observed the residues that are directly involved in the interaction with the PS (K613, K614, K617, K621, R624, R626, K631, K635 and K636) and determined which amino acid variations were present between each of these positions. Then, the one that presented the most variability was chosen (K636).

A mutation was generated in a Swiss PDB viewer [60] based on different variations that were found in the most variable residue (K636A, K636N, and K636R).

### 4.5. Determination of Interaction Forces between CagA N-Terminal and PS

From the PDBs generated from the respective mutations, SwissDock was used to dock with PS [37]. SwissDock is based on the docking software EADock DSS Ḋocking assays were carried out in the CHARMM22/27 all-hydrogen force field [41], for target proteins and ligands that had been uploaded as CHARMM-formatted files divided into protein and non-protein parts. The former was further decomposed into CHARMM segments, i.e., protein chains or units. The preparation of the target and ligand by CHARMM topology, parameters and coordinates were derived automatically from the Merck Molecular Force Field as described in [37]. The default parameters considered the whole target protein structure during docking. CHARMM formatted files, protein structure file (PSF), coordinate file (CRD) and parameter files (PAR), for each docking can be found in the Supplementary Materials. A total of 256 models were assessed for all docking assays, only models that showed interaction in any of the residues, which are known to be essential to maintain the bond between cagA-PS were considered to run the statistical tests (Table S2). The web server was run with all default settings and set to “Accurate” according to [40]. Using Chimera [61], we found the amount of hydrogen bonds present in each interaction. Finally, using the results obtained from the docking, we obtained different changes in the Gibbs free energy for all the models of each mutation. The free energy was calculated using the FACTs implicit solvation model based on the effective Born radius as described in detail in [42].

### 4.6. Statistical Analysis

For the statistical analysis of the data, we redefined certain groups: for the region, data were grouped according to providence, eastern and western. For the pathology, we took into account the mild and severe disease criteria. A slight gastric disease included patients with erythematous and/or chronic nodular gastritis. On the other hand, severe disease included patients that presented erosive gastric disease and an acute gastric ulcer, dysplasia/metaplasia and gastric cancer. For the analysis of the delta G and the hydrogen bond count of all the docking models, a linear mixed model (LMM) with random effects was calculated for each of the two response parameters using R programming [62] with the *lme4* and *ggplot2* packages. This LMM was constructed considering the clustering in each mutation as a random effect in the two models, followed by a likelihood ratio test using the ANOVA function comparing the LMM the effect of the free energy and hydrogen bond with a null model.

## Figures and Tables

**Table 1:** Results for the best model from each mutation. The ΔG obtained from the molecular docking in SwissDock and the hydrogen count obtained from Chimera.

Mutation	Model Number	Cluster	ΔG (Kcal/mol)	H-Bonds
Crystal	1.81	10	−8.919907	1
K636A	1.69	8	−8.665261	0
K636R	1.74	8	−8.701923	3
K636N	1.60	0	−8.515097	4

**Table 2:** Sequences from the dataset with the K636N mutation.

Sequence Number	Accession Number	Region *	Pathology
39	22,335,784	Eastern	Severe
104	259,123,360	Western	Severe
106	259,123,364	Eastern	Severe
112	307,135,434	Eastern	Severe
115	307,135,440	Eastern	Severe
116	307,135,442	Eastern	Severe
117	307,135,444	Eastern	Mild
119	307,135,448	Eastern	Severe
121	307,135,452	Eastern	Mild
125	307,135,460	Eastern	Severe
127	307,135,464	Eastern	Severe
135	335,335,488	Western	Severe
150	345,421,953	Eastern	Mild

* Region of origin of the sample.

## Supplementary Materials

The following are available online at http://www.mdpi.com/1422-0067/19/10/3273/s1.

## Abbreviations

The following abbreviations are used in this manuscript:

CagA	Cytotoxin-associated gen A
Cag PAI	CagA pathogenic island
c-Abl	Mammalian Abelson murine leukemia viral oncogene
CHARMM	Chemistry at Harvard Macromolecular Mechanics
CM	Multimerization sequence
c-Met	Tyrosine-protein kinase Met
FACTS	Fast Analytical Continuum Treatment of Solvation
LMM	Linear Mixed Model
MALT	Mucosa-associated lymphoid tissue
MARK	Microtubule affinity-regulating kinase
MSA	Multiple Sequence Alignment
NCBI	The National Center for Biotechnology Information
NFκB	Nuclear factor κB
PAR1	Protease-activated receptor 1
PDB	Protein Databank
PS	Phosphatidylserine
Src	Proto-oncogene tyrosine-protein kinase
TCF	Transcription factor
T4SS	Type IV secretion system
