# Supplementary material for: Structural Analysis of Variability and Interaction of the N-terminal of the Oncogenic Effector CagA of Helicobacter pylori with Phosphatidylserine
Source: Int J Mol Sci. 2018 Oct 22;19(10):3273. doi: 10.3390/ijms19103273 (PMC6214045; doi:10.3390/ijms19103273)
Supplement: Supplementary file 1 [file ijms-19-03273-s001.zip › ijms-331670-supplementary/Supplementary Material.docx]

**Supplementary Material**

**Table S1.** Sequence Database found in the search from NCBI**.** Parameters used for selection were: pathology, isolation region and type of EPIYA.

| Number | Accession (GI) | Country | Pathology | EPIYA |
| --- | --- | --- | --- | --- |
| 1 | 2073134 | Japan | Gastroduodenal Disease | ABCCC |
| 4 | 6573219 | Japan | Gastric Cancer | ABD |
| 7 | 12225001 | Japan | Gastric Cancer | ABBD |
| 8 | 12225003 | Japan | Chronic atrophic gastritis | ABD |
| 9 | 12225005 | Japan | Chronic atrophic gastritis | ABD |
| 10 | 12225007 | Japan | Chronic atrophic gastritis | ABD |
| 11 | 12225009 | Japan | Peptic Ulcer | ABD |
| 12 | 12225011 | Japan | Peptic Ulcer | ABD |
| 13 | 12225013 | Japan | Peptic Ulcer | ABD |
| 14 | 12225015 | Japan | Chronic atrophic gastritis | ABD |
| 15 | 12225017 | Japan | Peptic Ulcer | ABD |
| 19 | 12225027 | Japan | Peptic Ulcer | ABBD |
| 20 | 12225029 | Japan | Gastric Cancer | ABD |
| 21 | 12225031 | Japan | Gastric Cancer | ABD |
| 24 | 22121670 | USA | Duodenal Ulcer | ABD |
| 25 | 22335392 | Japan | Atrophic Gastritis | ABD |
| 26 | 22335396 | Japan | Atrophic Gastritis | ABD |
| 27 | 22335405 | Japan | Atrophic Gastritis | BABD |
| 28 | 22335409 | Japan | Atrophic Gastritis | AABD |
| 29 | 22335412 | East Asia | Atrophic Gastritis | ABABD |
| 30 | 22335416 | Japan | Atrophic Gastritis | ABBD |
| 31 | 22335420 | Japan | Atrophic Gastritis | ABD |
| 32 | 22335424 | Japan | Atrophic Gastritis | ABBD |
| 33 | 22335428 | Japan | Gastric Ulcer | ABCC |
| 34 | 22335433 | Japan | Duodenal Ulcer | ABDD |
| 35 | 22335437 | Japan | Atrophic Gastritis | ABABD |
| 36 | 22335442 | Japan | Atrophic Gastritis | ABD |
| 37 | 22335446 | Japan | Atrophic Gastritis | ABCCC |
| 38 | 22335449 | Japan | Atrophic Gastritis | ABD |
| 39 | 22335784 | Japan | Atrophic Gastritis | ABC |
| 40 | 22335788 | Japan | Atrophic Gastritis | ABD |
| 43 | 52693781 | East Asia | Atrophic Gastritis | ABD |
| 45 | 52693787 | Japan | Atrophic Gastritis | ABCC |
| 46 | 52693789 | East Asia | Duodenal Ulcer | ABD |
| 47 | 52693791 | Japan | Atrophic Gastritis | ABD |
| 48 | 52693793 | Occidental | Duodenal Ulcer | ABC |
| 49 | 52693795 | Occidental | Atrophic Gastritis | ABC |
| 50 | 52693797 | Occidental | Duodenal Ulcer | ABC |
| 51 | 52693799 | Occidental | Atrophic Gastritis | ABC |
| 52 | 52693801 | East Asia | Duodenal Ulcer | ABD |
| 53 | 52693803 | East Asia | Atrophic Gastritis | ABD |
| 54 | 52693805 | Occidental | Atrophic Gastritis | ABC |
| 55 | 52693807 | Occidental | Duodenal Ulcer | ABCCC |
| 56 | 52693809 | Occidental | Duodenal Ulcer | ABC |
| 57 | 52693811 | Occidental | Atrophic Gastritis | AB |
| 58 | 52693813 | Occidental | Duodenal Ulcer | ABC |
| 60 | 52693817 | East Asia | Atrophic Gastritis | ABD |
| 61 | 52693819 | Occidental | Duodenal Ulcer | ABC |
| 62 | 52693821 | Occidental | Duodenal Ulcer | ABD |
| 64 | 52693825 | Occidental | Duodenal Ulcer | ABC |
| 68 | 67483340 | Amsterdam | Dyspepsia | ABBC |
| 70 | 83701033 | China | Chronic atrophic gastritis | ABD |
| 71 | 110292846 | East Asia | Gastric Cancer | ABD |
| 72 | 110292848 | East Asia | Gastric Cancer | ABD |
| 73 | 110292850 | East Asia | Gastric Cancer | ABD |
| 74 | 110292852 | East Asia | Gastric Cancer | ABD |
| 76 | 110292856 | East Asia | Gastric Cancer | ABD |
| 77 | 110292858 | Occidental | Gastric Cancer | ABCC |
| 78 | 110292860 | East Asia | Gastric Cancer | ABD |
| 79 | 110292862 | East Asia | Gastric Cancer | ABD |
| 80 | 110292864 | East Asia | Gastric Cancer | ABD |
| 81 | 110292866 | East Asia | Gastric Cancer | ABD |
| 82 | 110292868 | East Asia | Gastric Cancer | ABD |
| 83 | 110292870 | East Asia | Gastric Cancer | ABD |
| 87 | 259123326 | Vietnam | Gastric Ulcer | ABD |
| 88 | 259123328 | Vietnam | Duodenal Ulcer | ABD |
| 89 | 259123330 | Vietnam | Duodenal Ulcer | ABD |
| 90 | 259123332 | Vietnam | Duodenal Ulcer | ABD |
| 91 | 259123334 | Vietnam | Gastric Cancer | ABD |
| 92 | 259123336 | Vietnam | Gastric Cancer | ABD |
| 93 | 259123338 | Vietnam | Gastric Cancer | ABD |
| 94 | 259123340 | Vietnam | Duodenal Ulcer | ABD |
| 95 | 259123342 | Vietnam | Duodenal Ulcer | ABD |
| 96 | 259123344 | Vietnam | Duodenal Ulcer | AABD |
| 97 | 259123346 | Vietnam | Gastric Cancer | ABD |
| 98 | 259123348 | Vietnam | Gastric Cancer | AD |
| 99 | 259123350 | Vietnam | Gastric Cancer | ABD |
| 100 | 259123352 | Vietnam | Duodenal Ulcer | ABD |
| 101 | 259123354 | Vietnam | Gastric Cancer | ABD |
| 102 | 259123356 | Vietnam | Gastric Ulcer | ABD |
| 104 | 259123360 | Occidental | Gastric Cancer | ABCC |
| 105 | 259123362 | Vietnam | Duodenal Ulcer | ABD |
| 106 | 259123364 | Vietnam | Gastric Cancer | ABD |
| 107 | 259123366 | Vietnam | Gastric Cancer | ABD |
| 108 | 259123368 | Vietnam | Duodenal Ulcer | ABD |
| 109 | 307135428 | Japan | Gastric Cancer | ABD |
| 110 | 307135430 | Japan | Gastritis | ABD |
| 111 | 307135432 | Japan | Gastritis | ABD |
| 112 | 307135434 | Japan | Gastric Cancer | ABCC |
| 113 | 307135436 | Japan | Gastric Ulcer | ABC |
| 114 | 307135438 | Japan | Gastric Ulcer | ABC |
| 115 | 307135440 | Japan | Gastric Ulcer | ABC |
| 116 | 307135442 | Japan | Duodenal Ulcer | ABC |
| 117 | 307135444 | Japan | Gastritis | ABC |
| 118 | 307135446 | Japan | Gastritis | ABD |
| 119 | 307135448 | Japan | Gastric Ulcer | ABC |
| 120 | 307135450 | Japan | Gastritis | ABC |
| 121 | 307135452 | Japan | Gastritis | ABCC |
| 122 | 307135454 | Japan | Gastritis | ABCC |
| 123 | 307135456 | Japan | Duodenal Ulcer | ABD |
| 124 | 307135458 | Japan | Gastritis | ABC |
| 125 | 307135460 | Japan | Gastric Ulcer | ABCC |
| 126 | 307135462 | Japan | Gastric Ulcer | ABC |
| 127 | 307135464 | Japan | Duodenal Ulcer | ABC |
| 135 | 335335488 | Colombia | Gastric Cancer | ABCCC |
| 136 | 335335490 | Colombia | Gastric Cancer | AACC |
| 137 | 335335492 | Colombia | Gastritis | ABCC |
| 138 | 335335494 | Colombia | Atrophic Gastritis | ABC |
| 139 | 335335496 | Colombia | Duodenal Ulcer | ABCC |
| 140 | 335335498 | Colombia | Atrophic Gastritis | ABABC |
| 150 | 345421953 | Senegal | Dolor abdominal | BC |
| 170 | 377652613 | Colombia | Duodenal Ulcer | ABCC |
| 171 | 377652609 | Colombia | Duodenal Ulcer | ABCC |
| 172 | 377652607 | Colombia | Gastric Cancer | ABCC |
| 174 | 548691481 | Colombia | Gastric Cancer | ABCC |
| 175 | 548691483 | Colombia | Gastritis | ABCC |
| 176 | 548691485 | Colombia | Atrophic Gastritis | ABCC |
| 177 | 548691487 | Colombia | Metaplasia | ABCC |
| 178 | 548691489 | Colombia | Metaplasia | ABCC |
| 179 | 548691491 | Colombia | Duodenal Ulcer | ABCC |
| 180 | 548691493 | Colombia | Gastric Cancer | ABC |
| 181 | 548691495 | Colombia | Gastric Cancer | ABCC |
| 182 | 548691497 | Colombia | Duodenal Ulcer | ABCC |
| 183 | 548691499 | Colombia | Duodenal Ulcer | ABD |
| 186 | 52693783 | Japan | Peptic Ulcer | ABD |
| 187 | 6573221 | Japan | Gastric Ulcer | ABCCC |
| 188 | 12225025 | Japan | Chronic atrophic gastritis | AB |

**S2 Table.** Energy and total of Hydrogen bonds for Crystal and Mutation Models. Only models the docking models obtained in SwissDock that presented an interaction with the N-terminal were selected to run the statistical tests in R. Were Cluster is the number of interacting model using the same location reference of PS to the N-Terminal CagA protein. The difference between models in the same cluster is the rotation of the allowed angles in the PS. ΔG is the Gibbs Free Energy (Kcal/mol). HBonds are the number of hydrogen bonds in each model.

| Mutation | Cluster | ΔG | Hbonds |
| --- | --- | --- | --- |
| 4DVY | 28 | -6.59372 | 3 |
| 4DVY | 28 | -6.63488 | 3 |
| 4DVY | 28 | -6.63488 | 3 |
| 4DVY | 28 | -6.63488 | 3 |
| 4DVY | 28 | -6.63488 | 3 |
| 4DVY | 28 | -6.67062 | 3 |
| 4DVY | 28 | -6.67062 | 3 |
| 4DVY | 28 | -6.67062 | 3 |
| 4DVY | 10 | -7.29307 | 1 |
| 4DVY | 10 | -7.30221 | 1 |
| 4DVY | 10 | -7.30221 | 1 |
| 4DVY | 10 | -7.30221 | 1 |
| 4DVY | 10 | -7.21762 | 1 |
| 4DVY | 10 | -7.1835 | 1 |
| 4DVY | 10 | -8.91991 | 1 |
| K636N | 0 | -8.50848 | 4 |
| K636N | 0 | -8.5151 | 4 |
| K636N | 0 | -8.51094 | 4 |
| K636N | 0 | -8.5151 | 4 |
| K636N | 0 | -8.5151 | 4 |
| K636N | 0 | -8.50848 | 4 |
| K636N | 0 | -8.5151 | 4 |
| K636N | 0 | -8.51094 | 4 |
| K636N | 6 | -7.91795 | 1 |
| K636N | 6 | -7.85934 | 1 |
| K636N | 6 | -7.61327 | 2 |
| K636N | 6 | -6.7117 | 2 |
| K636N | 18 | -7.73668 | 0 |
| K636N | 18 | -7.73668 | 0 |
| K636N | 18 | -7.73668 | 0 |
| K636N | 18 | -7.73668 | 0 |
| K636N | 18 | -7.70981 | 0 |
| K636N | 18 | -7.72128 | 0 |
| K636N | 18 | -7.72128 | 0 |
| K636N | 18 | -7.72128 | 0 |
| K636N | 21 | -7.84754 | 2 |
| K636N | 21 | -7.84754 | 2 |
| K636N | 21 | -7.84754 | 2 |
| K636N | 21 | -7.83932 | 2 |
| K636N | 21 | -7.83932 | 2 |
| K636N | 21 | -7.83932 | 2 |
| K636N | 21 | -7.76492 | 1 |
| K636N | 21 | -7.76492 | 1 |
| K636N | 25 | -8.36419 | 2 |
| K636N | 25 | -8.37319 | 2 |
| K636N | 25 | -7.52496 | 1 |
| K636N | 30 | -8.46167 | 4 |
| K636N | 30 | -8.51244 | 4 |
| K636N | 30 | -8.04953 | 4 |
| K636N | 30 | -6.87024 | 2 |
| K636R | 7 | -8.48093 | 1 |
| K636R | 7 | -8.51774 | 1 |
| K636R | 7 | -8.51051 | 1 |
| K636R | 7 | -8.51051 | 1 |
| K636R | 7 | -8.51051 | 1 |
| K636R | 8 | -8.65689 | 3 |
| K636R | 8 | -8.67863 | 3 |
| K636R | 8 | -8.65689 | 3 |
| K636R | 8 | -8.67863 | 3 |
| K636R | 8 | -8.70192 | 3 |
| K636R | 8 | -8.70192 | 3 |
| K636R | 8 | -8.70192 | 3 |
| K636R | 8 | -8.70192 | 3 |
| K636R | 9 | -7.77946 | 0 |
| K636R | 9 | -7.77946 | 0 |
| K636R | 9 | -7.77946 | 0 |
| K636R | 9 | -7.97347 | 2 |
| K636R | 9 | -7.97347 | 2 |
| K636R | 9 | -7.97347 | 2 |
| K636R | 9 | -7.93328 | 1 |
| K636R | 9 | -7.93328 | 1 |
| K636R | 28 | -6.08855 | 0 |
| K636R | 28 | -6.08855 | 0 |
| K636R | 28 | -6.08855 | 0 |
| K636R | 28 | -6.08855 | 0 |
| K636R | 28 | -6.08855 | 0 |
| K636R | 28 | -5.95155 | 0 |
| K636R | 28 | -5.95155 | 0 |
| K636R | 28 | -5.95155 | 0 |
| K636R | 29 | -6.6821 | 0 |
| K636R | 29 | -6.68549 | 0 |
| K636R | 29 | -6.68549 | 0 |
| K636R | 29 | -6.68549 | 0 |
| K636R | 29 | -6.68549 | 0 |
| K636R | 29 | -6.7039 | 0 |
| K636R | 29 | -6.7039 | 0 |
| K636R | 29 | -6.7039 | 0 |
| K636R | 33 | -7.64615 | 3 |
| K636R | 33 | -7.59375 | 3 |
| K636R | 33 | -7.62225 | 3 |
| K636A | 7 | -8.10353 | 2 |
| K636A | 7 | -8.13613 | 2 |
| K636A | 7 | -8.10676 | 2 |
| K636A | 7 | -8.32935 | 2 |
| K636A | 7 | -6.12246 | 1 |
| K636A | 7 | -6.11635 | 1 |
| K636A | 7 | -8.26822 | 3 |
| K636A | 7 | -8.28707 | 3 |
| K636A | 8 | -8.49543 | 2 |
| K636A | 8 | -8.49543 | 2 |
| K636A | 8 | -8.49543 | 2 |
| K636A | 8 | -8.48028 | 1 |
| K636A | 8 | -8.48028 | 1 |
| K636A | 8 | -8.48028 | 1 |
| K636A | 8 | -8.66526 | 1 |
| K636A | 8 | -8.66526 | 1 |
| K636A | 15 | -6.75848 | 0 |
| K636A | 15 | -6.75848 | 0 |
| K636A | 15 | -6.73222 | 0 |
| K636A | 15 | -6.67188 | 0 |
| K636A | 15 | -6.67188 | 0 |
| K636A | 15 | -6.67188 | 0 |
| K636A | 15 | -6.67188 | 0 |
| K636A | 15 | -6.67188 | 0 |
| K636A | 17 | -6.2147 | 0 |
| K636A | 17 | -6.2147 | 0 |
| K636A | 17 | -6.19683 | 0 |
| K636A | 17 | -6.19683 | 0 |
| K636A | 17 | -6.19683 | 0 |
| K636A | 17 | -6.19683 | 0 |
| K636A | 17 | -6.1852 | 0 |
| K636A | 17 | -6.1852 | 0 |
| K636A | 18 | -6.14987 | 0 |
| K636A | 18 | -6.14987 | 0 |
| K636A | 18 | -6.14987 | 0 |
| K636A | 18 | -6.21486 | 0 |
| K636A | 18 | -6.1918 | 0 |
| K636A | 18 | -5.7124 | 0 |
| K636A | 18 | -5.76062 | 0 |
| K636A | 18 | -5.39119 | 0 |
| K636A | 31 | -5.9708 | 0 |
| K636A | 31 | -5.96028 | 0 |
| K636A | 31 | -5.96028 | 0 |
| K636A | 31 | -5.96028 | 0 |
| K636A | 31 | -5.96028 | 0 |
| K636A | 31 | -5.93288 | 0 |
| K636A | 31 | -5.93288 | 0 |
| K636A | 31 | -5.93288 | 0 |
