# Supplementary material for: Structural Analysis of Variability and Interaction of the N-terminal of the Oncogenic Effector CagA of Helicobacter pylori with Phosphatidylserine
Source: Int J Mol Sci. 2018 Oct 22;19(10):3273. doi: 10.3390/ijms19103273 (PMC6214045; doi:10.3390/ijms19103273)
Supplement: Supplementary file 1 [file ijms-19-03273-s001.zip › ijms-331670-supplementary/MSA_best_alignment_AQUA.zip!/AnexB_ConSurf Color Coded MSA.pdf]

# ANNEX B

## CONSURF COLOR-CODED MSA

| | 1 … 50 |
|---|---|
| Sequence140-NP-GA-Col-ABABC | MTNETI--DQ-----Q--PQTEAAFNPQQFINNLQVAFLKLDNAVASFDP |
| Sequence123-NP-UD-Jp-ABD | MTNETI--DQTTTPDQ--TPNPTDFVPQRFINNLQVAFLKVDNAVASFDP |
| Sequence111-NP-G-Jp-ABD | MTNETI--DQTTTPDQ--TPNPTDFVPQRFINNLQVAFLKVDNAVASFDP |
| Sequence57-NP-GA-OCC-AB | MTNETI--DQ--------TPSQTAFNPQQFINNLQVAFLKVDNAVASFDP |
| Sequence172-P-CG-Col-ABCC | MTNETI--DQ-----Q--PQTEAAFNPQQFINNLQVAFLKLDNAVASFDP |
| Sequence110-NP-UD-Jp-ABD | MTNETI--DQTTTPNQ--TPNQTDFVPQRFINNLQVAFIKVDDAVASFDP |
| Sequence95-NP-UD-Vit-ABD | MTNETI--DQTTTPDQ--TLNPTDFVPQRFINNLQVAFLKVDKAVASYDP |
| Sequence107-P-CG-Vit-ABD | MTKETI--DQTTTPDQ--TLNPTDFVPQRFINNLQVAFLKVDDAVASFDP |
| Sequence106-P-CG-Vit-ABD | MTNETI--DQTTTPDQ--TLTQRILFRNQFINNLQVAFIKVDNAVASFDP |
| Sequence105-NP-UD-Vit-ABD | MTNETI--DQTTTPDQ--TPNPTDFVPQRFINNLQVAFLKVDRAVASFDP |
| Sequence126-NP-UG-Jp-ABC | MTNETI--DQ-----Q--PQTEAAFNPQQFINNLQVAFLKVDNAVASYDP |
| Sequence135-P-CG-Col-ABCCC | MTNETI--DQ-----Q--PQTEAAFNPQQFINNLQVAFLKLDNAVASFDP |
| Sequence138-NP-GA-Col-ABC | MTNETI--DQ-----Q--PQTEAAFNPQQFINNLQVAFLKLDNAVASFDP |
| Sequence13-NP-UP-Jp-ABD | MTNETI--DQTTTPDQ------TDFVPQRFINNLQVAFIKVDNAVASFDP |
| Sequence11-NP-UP-Jp-ABD | MTNETI--DQTTTPDQ--TPNQTDFVPQRFINNLQVAFIKVDNAVASFDP |
| Sequence50-NP-GA-OCC-ABC | MTNETI--------DQ--TPSQTAFNPQQFINNLQVAFLKVDNAVASFDP |
| Sequence26-NP-GA-Jp-ABD | MTNETI--DQTTTPDQ------TDFVPQRFINNLQVAFIKVDNAVASFDP |
| Sequence55-NP-UD-OCC-ABCCC | MTNETI--DQ--TPSQ------TAFNPQQFINNLQVAFLKVDNAVASFDP |
| Sequence178-NP-M-Col-ABCC | MTNETI--DQ-----Q--PQTEAAFNPQQFINNLQVAFLKLDNAVASFDP |
| Sequence40-NP-GA-Jp-ABD | MTNETI--DQTITPDQ------TDFVPQRFINNLQVAFIKVDNAVASFDP |
| Sequence43-NP-GA-Jp-ABD | MTNETI--DQTITPDQ------TDFVPQRFINNLQVAFIKVDSAVASFDP |
| Sequence139-NP-UD-Col-ABCC | MTNETI--DQ-----Q--PQTEAAFNPQQFINNLQVAFLKLDNAVASFDP |
| Sequence7-P-CG-Jp-ABBD | MTNETI--DQTTTPDQ--TPNQTDFVPQRFINNLQAAFIKVDSAVASFDP |
| Sequence91-P-CG-Vit-ABD | MTNETI--DQTTTPDQ--TLNPTDFVPHRFINNLQVAFLKVDNAVASYDP |
| Sequence29-NP-GA-Jp-ABABD | MTNETI--DQTTTPDQ--TPNQTDFVPQRFINNLQVAFIKVDNAVASFDP |
| Sequence78-P-CG-EA-ABD | MTSETI--EQ--TPD------QTDFVPQRFINNLQVAFIKVDSAVASFDP |
| Sequence188-NP-GCA-Jp-AB | MTNETI--DQ-----Q--PQTEAAFNPQQFINNLQVAFLKVDNAVASYDP |
| Sequence72-P-CG-EA-ABD | MTSETI--DQTITPDQ------TDFVPQRFINNLQVAFIKVDDAVASFDP |
| Sequence79-P-CG-EA-ABD | MANETI--DQ--------TPSQTAFNPQQFINNLQVAFLEVGNAVASFDP |
| Sequence61-NP-UD-OCC-ABC | MTNETI--DQTRTPDQ--TPNQADFVPQRFINNLQVAFIKVDNAVASFDP |
| Sequence125-NP-UG-Jp-ABCC | MTNETI--DQ-----Q--PQTETAFNPQQFINNLQVAFLKVDNAVASYDP |
| Sequence175-NP-G-Col-ABCC | MTNETI--DQ-----Q--PQTEAAFNPQQFINNLQVAFLKLDNAVASFDP |
| Sequence122-NP-G-Jp-ABCC | MTNETI--DQ-----Q--PQTEAAFNPQQFINNLQVAFLKLDNAVASFDP |
| Sequence36-NP-GA-Jp-ABD | MTNETI--DQTTTPDQ------TDFVPQRFINNAQVAFIEVDNAVASFDP |
| Sequence58-NP-UD-OCC-ABC | MANETIDQ----TPSQ------TAFNPQQFINNLQVAFLKVDNAVASFDP |
| Sequence27-NP-GA-Jp-ABBD | MTNETI--DQTTTLDQ--TPNQTDFVPQRFINNLQVAFLKVDNAVASFDP |
| Sequence93-P-CG-Vit-ABD | MTNETI--DQTTTPDQ--TLNPTDFVPQRFINNLQVAFLTVDNAVASFDP |
| Sequence177-NP-M-Col-ABCC | MTNETI--DQ-----Q--PQTEAAFNPQQFINNLQVAFLELDNAVASFDP |
| Sequence182-NP-UD-Col-ABCC | MTNETI--DQ-----Q--PQTEAAFNPQRFINNLQVAFLKLDNAVASFDP |
| Sequence114-NP-UG-Jp-ABC | MTNETI--DQ-----Q--PQTEAAFNPQQFINNLQVAFLKVDNAVASYDP |
| Sequence74-P-CG-EA-ABD | MTNETI--EQ--TPD------QTDFVPQRFINNLQVAFIKVDNAVASFDP |
| Sequence20-P-CG-Jp-ABD | MTNETI--DQTITPDQ------TDFVPQRFINNLQVAFIKVDSAVASFDP |
| Sequence14-NP-GCA-Jp-ABD | MTNETI--DQTITPDQ--TPNQTDFVPQRFINNLQVAFIKVDSAVASFDP |
| Sequence39-NP-GA-Jp-ABC | MTNETI--DQ-----Q--PQTEAAFNPQQFINNLQVAFLKVDNAVASFDP |
| Sequence170-NP-UD-Col-ABCC | MTNETI--DQ-----Q--PQTEVAFNPQQFINNLQVAFLKLDNAVASFDP |
| Sequence76-P-CG-EA-ABD | MTNETI--DQTTTPDQ--TPNQTDFVPQRFINNLQVAFLKVDNAVASFDP |
| Sequence53-NP-GA-EA-ABD | MTNETI--DQTTTPDQ------TDFVPQRFINNLQVAFIKVDNAVASFDP |
| Sequence174-P-CG-Col-ABCC | MTNETI--DQ-----Q--PQTEAAFNPQRFINNLQVAFLKLDNAVASFDP |
| Sequence33-NP-UG-Jp-ABD | MTNETI--DQ-----Q--PQTEVAFNPQQFINNLQVAFLKLDNAVASFDP |
| Sequence60-NP-GA-EA-ABD | MTNETI--DQTTTPDQ--TPNQTDFVPQRFINNLQVAFIKVDNAVASFDP |
| Sequence38-NP-GA-Jp-ABD | MTNETI--DQTTTPDQ--TPNQTDFVPQQFINNLQVAFLKVDNAVASFDP |
| Sequence1-NP-EG-Jp-ABCCC | MTNETI--AQ-----Q--PQTEAAFNPQQFINNLQVAFLKVDNAVASYDP |
| Sequence101-P-CG-Vit-ABD | MTNETI--DQTTTPDQ--TLNPTDFVPQRFINNLQVAFLKVDSAVALYDP |
| Sequence64-NP-UD-OCC-ABC | MTNETI--DQTRTPDQ--TQSQTAFDPQRFINNLQVAFIKVDNVVASFDP |

```
Sequence81-P-CG-EA-ABD        M T N E T I - - D Q T T T P D Q - - T P N Q T D F V P Q R F I N N L Q V A F I K V D N A V A S F D P
Sequence150-NP-DA-Sen-BC      M T N E T I - - N Q - - - - - Q - - P Q T E A A F N P Q Q F I N N L Q V A F L K V D N A V A S Y D P
Sequence90-NP-UD-Vit-ABD      M T N E T I - - D Q T T T P D Q - - T L N P T D F V P Q R F I N N L Q V A F L K V D S A V A S F D P
Sequence136-P-CG-Col-AACC     M T N E T I - - D Q - - - - - Q - - P Q T E A A F N P Q Q F I N N L Q V A F L K L D N A V A S F D P
Sequence15-NP-UP-Jp-ABD       M T N E T I - - D R T I T P D Q - - T P N Q T D F V P Q R F I N N L Q V A F I K V D D A V A S F D P
Sequence108-NP-UD-Vit-ABD     M T N E T I - - D Q T T T P V Q - - T L N P T E F V P Q R F I N N L Q V A F L K V D N A V A S F D P
Sequence104-P-CG-OCC-ABCC     M T N E T I - - D Q - - - - - P - - P Q T E A A F N P Q Q F I N N L Q V A F L K V D N A V A S Y D P
Sequence179-NP-UD-Col-ABCC    M T N E T I - - D Q - - - - - Q - - P Q T E A A F N P Q Q F I N N L Q V A F L K L D N A V A S F D P
Sequence37-NP-GA-Jp-ABCCC     M T N E T I - - D Q - - T P S Q - - - - - - T A F N P Q Q F I N N L Q V A F L K V D N A V A S F D P
Sequence89-NP-UD-Vit-ABD      M T N E T I - - D Q T T T P D Q - - T P N P T D F V P Q R F I N N L Q V A F I K V D N A V A S Y D P
Sequence92-P-CG-Vit-ABD       M T N E T I - - D Q T T T P D Q - - T L N P T D F V P Q R F I N N L Q V A F L K V D N A V A S Y D P
Sequence49-NP-GA-Jp-ABC       M T N E T I - - D Q - - - - - - - - T P S Q T A F N P Q Q F I N N L Q V A F L K V D N A V A S F D P
Sequence181-P-CG-Col-ABCC     M T N E T I - - D Q - - - - - Q - - P Q T E A A F N P Q R F I N N L Q V A F L K L D N A V A S F D P
Sequence25-NP-GA-Jp-ABD       M T N E T I - - D Q T T T P D Q - - T P N Q T D F V P Q R F I N N L Q V A F I K V D N A V A S F D P
Sequence4-P-CG-Jp-ABD         M T N E T I - - D Q T T T P D Q - - - - - - T G F V P Q R F I N N L Q V A F I K V D N A V A S F D P
Sequence121-NP-G-Jp-ABCC      M T N E T I - - D Q - - - - - Q - - P Q T E A A F N P Q Q F I N N L Q V A F L K F D N A V A S F D P
4dvy                          M T N E T I - - D Q T R T P D Q - - T Q S Q T A F D P Q Q F I N N L Q V A F I K V D N V V A S F D P
Sequence186-NP-UP-Jp-ABD      M T N E T I - - D Q - - - - - - - - T P N Q T D F V P Q R F I N N L Q V A F I K V D N A V A S F D P
Sequence62-NP-UD-OCC-ABD      M S N E T I - - - - - - - - D Q - - T P S Q T A F N P Q Q F I N N L Q V A F L K G D N S V A S F D P
Sequence9-NP-GCA-Jp-ABD       M T N E T I - - D Q T T T P D Q - - T P N Q T D F V P Q Q F I N N L Q V A F I K V D N A V A S F D P
Sequence12-NP-UP-Jp-ABD       M T N E T I - - D Q T I T P D Q - - - - - - T D F V P Q R F I N N L Q V A F L K V D N A V A S F D P
Sequence35-NP-GA-Jp-ABABD     M T N E T I - - D Q T T T P D Q - - T P N Q T D F V P Q R F I N N L Q V A F I K V D N A V A S F D P
Sequence54-NP-GA-OCC-ABC      M T N E T I - - - - - - - - D Q - - T P S Q T A F N P Q Q F I N N L Q V A F L E V D N A V A S F D P
Sequence109-P-CG-Jp-ABD       M T N E T I - - D Q T T T P D Q - - T L N P T D F V P Q R F I N N L Q V A F L K V D N A V A S F D P
Sequence96-NP-UD-Vit-ABD      M T N E T I - - D Q T T T P D Q - - T L N P T D F V P Q Q F I N N L Q V A F I K V D N A V A S Y D P
Sequence80-P-CG-EA-ABD        M T N E T I - - D Q T T T P D Q - - T L N Q T D F V P Q R F I N N L Q V A F I K V D N A V A S F D P
Sequence82-P-CG-EA-ABD        M T N E T I - - D Q T T T P D Q - - T L N P T D F V P Q R F I N N L Q V A F I K V D N A V A S F D P
Sequence10-NP-GCA-Jp-ABD      M T N E T I - - N Q T I T P D Q - - - - - - T D F V P Q R F I N N L Q V A F I K V D N A V A S F D P
Sequence19-NP-UP-Jp-ABBD      M T N E T I - - E Q A T T P D Q - - - - - - T D F V P Q R F I N N L Q V A F I E V D N A V A S F D P
Sequence115-NP-UG-Jp-ABC      M T N E T I - - D Q - - - - - Q - - P Q T E A A F N P Q Q F I N N L Q V A F L K V D N A V A S Y D P
Sequence45-NP-GA-Jp-ABCC      M T N E T I - - D Q - - - - - Q - - P Q T E V A F N P Q Q F I N N L Q V A F L K L D N A V A S F D P
Sequence119-NP-UG-Jp-ABC      M T N E T I - - D Q - - - - - Q - - P Q T E A A F N P Q Q F I N N L Q V A F L K V D N A V A S Y D P
Sequence70-NP-GA-Ch-ABD       M T N E T I - - D Q T T T P N Q - - - - - - T D F V P Q R F I N N L Q V A F L K V D S A V A S F D P
Sequence52-NP-UD-EA-ABD       M A N E T I - - D Q T I T P D Q - - - - - - T D F V P Q R F I N N L Q V A F I K V D N A V A S F D P
Sequence97-P-CG-Vit-ABD       M T N E T I - - D Q T T T P D Q - - T P N P T D F V P Q R F I N N L Q V A F L K V D S A V A S F D P
Sequence94-NP-UD-Vit-ABD      M T N E T I - - D Q - - - - - Q - - P Q T E A A F N P Q Q F I N N L Q V A F L K V D N A V A S Y D P
Sequence176-NP-GA-Col-ABCC    M T N E T I - - D Q - - - - - Q - - P Q T E A A F N P Q R F I N N L Q V A F L K L D N A V A S F D P
Sequence68-NP-D-Am-ABBC       M T N E T I - - N Q - - - - - Q - - P Q T E A A F N P Q Q F I N N L R A A F L K V D N A V A S Y D P
Sequence47-NP-GA-Jp-ABD       M T N E T I - - D Q T T T P D Q - - T P N Q T D F V P Q R F I N N L Q V A F L K V D N A V A S F D P
Sequence56-NP-UD-OCC-ABC      M T N E T I - - D Q - - T P S Q - - - - - - T A F N P Q Q F I N N L Q V A F L K V D N A V A S F D P
Sequence31-NP-GA-Jp-ABD       M T N E T I - - D Q - - - - - - - - T T N Q T D F V P Q R F I N N L Q V A F L K V G D A V A S F D P
Sequence46-NP-UD-Jp-ABD       M T N E T I - - D Q T T T P D Q - - T P N Q T D F V P Q R F I N N L Q V A F L K V D N A V A S F D P
Sequence102-NP-UG-Vit-ABD     M T N E T I - - D Q T T T P D Q - - T L N P T D F V P Q R F I N N L Q V A F L K V D S A V A L Y D P
Sequence71-P-CG-EA-ABD        M T S E T I - - D Q T T T P D Q - - - - - - T D F V P Q R F I N N L Q V A F I K V D N A V A S F D P
Sequence30-NP-GA-Jp-ABBD      M T N E T I - - D Q T T T P D Q - - T P N Q T D F V P Q R F I N N L Q A A F I K V D N A V A S F D P
Sequence28-NP-GA-Jp-AABD      M T N E T I - - D Q T T T P D Q - - T P N Q T D F V P Q R F I N N L Q V A F I K V D N A V A S F D P
Sequence98-P-CG-Vit-AD        M T N E T I - - D Q T T T P D Q - - T L N P T D F V P Q R F I N N L Q V A F L K V D N A V A S Y D P
Sequence99-P-CG-Vit-ABD       M T S E T I - - D Q T T T P D Q - - - - - - T D F V P Q R F I N N L Q V A F L K I G S A V A S F D P
Sequence24-NP-UD-USA-ABD      M T N E T I - - D Q T T T P D Q - - T P N Q T D F V P Q R F I N N L Q V A F I K V D N A V A S F D P
Sequence124-NP-G-Jp-ABC       M T N E T I - - D Q - - - - - Q - - P Q T E A A F N P Q Q F I N N L Q V A F L K V D N A V A S Y D P
Sequence77-P-CG-OCC-ABCC      M T N E T I - - - - - - - - D Q T I T P D Q T D F V P Q R F I N N L Q V A F I K V D S A V A S F D P
Sequence113-NP-UG-Jp-ABC      M T N E T I - - D Q - - - - - Q - - P Q T E A A F N P Q Q F I N N L Q V A F L K V D N A V A S Y D P
Sequence88-NP-UD-Vit-ABD      M T N E T I - - D Q T T T P D Q - - T L N P T D F V P Q R F I N N L Q V A F L K V D S T V A S F D P
Sequence100-NP-UD-Vit-ABD     - - - - - - - - M K T T T P D Q - - T L N P T D F V P Q R F I N N L Q V A F L K V D N A V A S Y D P
Sequence137-NP-G-Col-ABCC     M T N E T I - - D Q - - - - - Q - - P Q T E V A F N P Q Q F I N N L Q V A F L K L D N A V A S F D P
Sequence117-NP-G-Jp-ABC       M T N E T I - - D Q - - - - - Q - - P Q T E A A F N P Q Q F I N N L Q V A F L K V D N A V A S Y D P
Sequence171-NP-UD-Col-ABCC    M T N E T I - - D Q - - - - - Q - - P Q T E A A F N P Q R F I N N L Q V A F L K L D N A V A S F D P
Sequence180-P-CG-Col-ABC      M T N E T I - - D Q - - - - - Q - - P Q T E A A F N P Q R F I N N L Q V A F L K L D N A V A S F D P
Sequence112-P-CG-Jp-ABCC      M T N E T I - - D Q - - - - - Q - - P Q T E A A F N P Q Q F I N N L Q V A F L K L D N A V A S F D P
Sequence127-NP-UD-Jp-ABC      M T N E T I - - D Q - - - - - Q - - P Q T E A A F N P Q Q F I N N L Q V A F L K L D N A V A S Y D P
Sequence51-NP-GA-OCC-ABC      M T N E T I - - - - - - - - D Q - - T P S Q T A F N P Q Q F I N N L Q V A F L K V D N T V A S F D P
```

```
Sequence73-P-CG-EA-ABD          MTNETI--DQTITPDQ--TPNQTDFVPQRFINNLQVAFIKVDNAVASFDP
Sequence183-NP-UD-Col-ABD       MTNETI--NQ-----Q--PQTEAAFNPQRFINNLQVAFLKLDNAVASFDP
Sequence34-NP-UD-Jp-ABDD        MTNETI--DQ-----Q--PQTEAAFNPQQFINNLQVAFLKLDNSITSFDP
Sequence21-P-CG-Jp-ABD          MTNETI--DQTTTPDQ------TDFVPQRFINNLQVAFIKVDNAVASFDP
Sequence32-NP-GA-Jp-ABD         MTNETI--DQTTTPDQ--TPNQTDFVPQRFINNLQVAFIKVDNAVASFDP
Sequence116-NP-UD-Jp-ABC        MTNETI--DQ-----Q--PQTEAAFNPQQFINNLQVAFLKVDNAVASYDP
Sequence8-NP-GCA-Jp-ABD         MTNETI--DQTITPDQ--TPNQTDFVPQRFINNLQVAFIKVDNAVASFDP
Sequence83-P-CG-EA-ABD          MTNETI--DQTTTPDQ------TDFVPQRFINNLQVAFIKVDNAVASFDP
Sequence48-NP-UD-OCC-ABC        MTNETI--DQ--------TPSQTAFNPQQFINNLQVAFLKVDNAVASYDP
Sequence120-NP-G-Jp-ABC         MTNETI--DQ-----Q--PQTEAAFNPQQFINNLQVAFLKLDNAVASFDP
Sequence187-NP-UG-Jp-ABCCC      MTNETI--DQ-----Q--PQTEAAFNPQQFINNLQVAFLKVDNAVASYDP
Sequence118-NP-G-Jp-ABD         MSNETI--DQTTTPDQ--TPNQTDFVPQRFINNLQVAFIKVDNAVASFDP
Sequence87-NP-UG-Vit-ABD        MTNETI--DQTTTPDQ--TLNETDFVPQRFINNLQAAFLKVDSAVASFDP

                                51                                              100
Sequence140-NP-GA-Col-ABABC     DQKPIVDKNDRDNRQAFDGISQLREEYSNKAIKNPTKKNQYFSDFINKSN
Sequence123-NP-UD-Jp-ABD        DQKPIVDKNDRDNRQAFEKISQLREEYANKAIKNPAKKNQYFSDFINKSN
Sequence111-NP-G-Jp-ABD         DQKPIVDKNDRDNRQAFEKISQLREEYANKAIKNPAKKNQYFSDFINKSN
Sequence57-NP-GA-OCC-AB         DQKPIVDKNDRDNRQAFEKISQLREEFANKAIKNPAKKNQYFSNFISKSS
Sequence172-P-CG-Col-ABCC       DQKPIVDKNDRDNRQAFDGISQLREEYSNKAIKNPTKKNQYFSDFINKSN
Sequence110-NP-UD-Jp-ABD        DQKPIVDKNDRDNRQAFEKISQLREEYANKAIKNPAKKNQYFSDFINKSN
Sequence95-NP-UD-Vit-ABD        DQKPIVDKNDRDNRQAFEKISQLREEYANKAIKNPAKKNQYFSDFINKSN
Sequence107-P-CG-Vit-ABD        DQKPIVDKNDRDNRQAFEKISQLREEYANKAIKNPAKKNQYFSDFINRSN
Sequence106-P-CG-Vit-ABD        DQKPIVDKNDKDNRQAFEKISQLREEYSNKAIKNPAKKNQYFSDFINKSN
Sequence105-NP-UD-Vit-ABD       DQKPIVDKNDKDNRQAFEKISQLREEYANKAIKNPTKRNQYFSDFINKSN
Sequence126-NP-UG-Jp-ABC        DQKPIVDKNDRDNRQAFDEISQLREEYSNKAIKNPTKKNQYFSDFINKSN
Sequence135-P-CG-Col-ABCCC      DQKPIVDKNDRDNRQAFDGISQLREEYSNKAIKNPTKKNQYFSDFINKIN
Sequence138-NP-GA-Col-ABC       DQKPIVDKNDKDNRQAFDGISQLREEYSNKAIKNPTKKNQYFSDFINKSN
Sequence13-NP-UP-Jp-ABD         DQKPIVDKNDRDNRQAFEKISQLREDYANKAIKNPAKKNQYFSDFINKSN
Sequence11-NP-UP-Jp-ABD         DQKPIVDKNDRDNRQAFEKISQLREEYANKAIKNPAKKNQYFSDFINKSN
Sequence50-NP-GA-OCC-ABC        DQKPIVDKNDRDNRQAFEKISQLREEFANKAIKNPAKKNQYFSNFISKSS
Sequence26-NP-GA-Jp-ABD         DQKPIVDKNDRDNRQAFEKISQLREEYANKAIKNPTKKNQYFSDFINKSN
Sequence55-NP-UD-OCC-ABCCC      DQKPIVDKNDRDNRQAFEKISQLREEFANKAIKNPAKKNQYFSNFISKSS
Sequence178-NP-M-Col-ABCC       DQKPIVDKNDSDNRQAFDGISQLREEYSNKAIKNPTKKNQYFSDFINKSN
Sequence40-NP-GA-Jp-ABD         DQKPIVDKNDRDNRQAFEKISQLREEYANKAIKNPTKKNQYFSDFINKSN
Sequence43-NP-GA-Jp-ABD         DQKPIVDKNDRDNRQAFEKISQLREEYANKAIKNPAKKNQYFSDFINKSN
Sequence139-NP-UD-Col-ABCC      DQKPIVDKNDRDNRKAFDEISQLREEYSNKAIKNPTKKNQYFSDFINKSN
Sequence7-P-CG-Jp-ABBD          DQKPIVDKNDRDNRQAFEKISQLREEYANKAIKNPTKKNQYFSDFINKSN
Sequence91-P-CG-Vit-ABD         DQKPIVDKNDRDNRQAFEKISQLREEYANKAIKNPAKKNQYFSDFINKTN
Sequence29-NP-GA-Jp-ABABD       DQKPIVDKNDRDNRQAFEKISQLREEYANKAIKNPTKKNQYFSDFINKSN
Sequence78-P-CG-EA-ABD          DQKPIVDKNDRDNRQAFEKISQLMEEYANKAIKNPTKKNQYFSDFINKSN
Sequence188-NP-GCA-Jp-AB        DQKSIVDKNDRDNRQAFEGISQLREEYSNKAIKNPTKKNQYFSDFINKSN
Sequence72-P-CG-EA-ABD          DQKPIVDKNDRDNRQAFEKISQLREEYANKAIKNPTKKNQYFSDFINKSN
Sequence79-P-CG-EA-ABD          DQKPIVDKNDRDNRQAFEKISQLREEYANKAIKNPAKKNQYFSDFINKSN
Sequence61-NP-UD-OCC-ABC        DQKPIVDKNDRDNRQAFEKISQLREEYANKAIKNPAKKNQYFSDFINKSN
Sequence125-NP-UG-Jp-ABCC       DQKPIVDKNDRDNRQAFDGISQLREEYSNKAIKNPTKKNQYFSDFINKSN
Sequence175-NP-G-Col-ABCC       DQKPIVDKNDKDNRQAFDGISQLREEYSNKAIKNPTKKNQYFSDFINKSN
Sequence122-NP-G-Jp-ABCC        DQKPIVDKNDRDNRQAFDGLSQLREEYSNKAIKNPTQKNQYFSDFINKSN
Sequence36-NP-GA-Jp-ABD         DQKPIVDKNDRDNRQAFEKISQLREEYANKAIKNPTKKNQYFSDFINKSN
Sequence58-NP-UD-OCC-ABC        DQKPIVDKNDRDNRQAFEKISQLREEFANKAIKNPAKKNQYFSNFISKSS
Sequence27-NP-GA-Jp-ABBD        DQKPIVDKNDRDNRQAFEKISQLRKEYANKAIKNPTKKNQYFSDFINKSN
Sequence93-P-CG-Vit-ABD         DQKPIVDKNDRDNRQAFEKISQLREEYANKAIKNPAKKNQYFSDFINKSN
Sequence177-NP-M-Col-ABCC       DQKPIVDKNDRDNRQAFEGISQLREEYSNKAIKNPTKKNQYFSDFINKSN
Sequence182-NP-UD-Col-ABCC      DQKPIIDKNDRDNRQAFDGISQLREEYSNKAIKNPTKKNQYFSDFINKSN
Sequence114-NP-UG-Jp-ABC        DQKPIVDKNDRDNRQAFNGISQLREEYSNKAIKNPTKKNQYFSDFINKSN
Sequence74-P-CG-EA-ABD          DQKPIVDKNDRDNRQAFEKISQLREEYANKAIKNPTKKNQYFSDFINKSN
Sequence20-P-CG-Jp-ABD          DQKPIVDKNDRDNRQAFEKISQLREEYANKAIKNPAKKNQYFSDFINKSN
Sequence14-NP-GCA-Jp-ABD        DQKPIVDKNDRDNRQAFEKISQLREEYANKAIKNPAKKNQYFSDFINKSN
Sequence39-NP-GA-Jp-ABC         DQKPIVDKNDRDNRQAFDGISQLREEYSNKAIKNPTKKNQYFSDFINKSN
Sequence170-NP-UD-Col-ABCC      DQKPIVDKNDRDNRQAFDGISQLREEYSNKAIKNPTKKNQYFSDFINKSN
Sequence76-P-CG-EA-ABD          DQKPIVDKNDRDNRQAFEKISQLREEYANKAIKNPTKKNQYFSDFINKSN
```

| | |
|---|---|
| Sequence53-NP-GA-EA-ABD | DQKPIVDKNDRDNRQAFEKISQLREEYANKAIKNPTKKNQYFSDFINKSN |
| Sequence174-P-CG-Col-ABCC | DQKPIVDKNDRDNRQAFDGISQLREEYSNKAIKNPTKKNQYFSDFINKSN |
| Sequence33-NP-UG-Jp-ABD | DQKPIVDKNDRDNRQAFEKISQLREEYSNKAIKNPAKKNQYFSDFINKSN |
| Sequence60-NP-GA-EA-ABD | DQKPIVDKNDRDNRQAFEKISQLREEYANKAIKNPTKKNQYFSDFINKSN |
| Sequence38-NP-GA-Jp-ABD | DQKPIVDKNDRDNRQAFEKISQLREEYANKAIKNPTKKNQYFSDFINKSN |
| Sequence1-NP-EG-Jp-ABCCC | DQKPIVDKNDRDNRQAFDGISQLREEYSNKAIKNPTKKNQYFSDFINKSN |
| Sequence101-P-CG-Vit-ABD | DQKPIVDKNDRDNRQAFEKISQLREEYSNKAIKNPTKKNQYFSDFINKSN |
| Sequence64-NP-UD-OCC-ABC | NQKPIVDKNDRDNRQAFEKISQLREEFANKAIKNPAKKNQYFSSFISKSS |
| Sequence81-P-CG-EA-ABD | DQKPIVDKNDRDNRQAFEKISQLREEYANKAIKNPTKKNQYFSDFINKSN |
| Sequence150-NP-DA-Sen-BC | DQKPIVDKNDRDNRQAFDGISQLREEYSNKAIKNPTKKNQYFSDFINKSN |
| Sequence90-NP-UD-Vit-ABD | DQKPIVDKNDRDNRQAFEKISQLREEYANKAIKNPAKKNQYFSDFINKSN |
| Sequence136-P-CG-Col-AACC | DQKPIVDKNDRDNRQAFERISQLREEYSNKAIKNPTKKNQYFSDFINKSN |
| Sequence15-NP-UP-Jp-ABD | NQKPIVDKNDRDNRQAFEKISQLMEEFANKAIKNPAKKNQYFSDFINKSN |
| Sequence108-NP-UD-Vit-ABD | DQKPIVDKNDRDNRQAFEKISQLREEYANKAIKNPAKKNQYFSDFINKSN |
| Sequence104-P-CG-OCC-ABCC | DQKPIVDKNDRDNRQAFDGISQLREEYSNKAIKNPTKKNQYFSDFINKSN |
| Sequence179-NP-UD-Col-ABCC | DQKPIVDKNDKDNRKAFDGISQLREEYSNKAIKNPTKKNQYFSDFINKSN |
| Sequence37-NP-GA-Jp-ABCCC | DQKPIVDKNDRDNRQAFEKISQLMEEFANKAIKNPAKKNQYFSNFISKSN |
| Sequence89-NP-UD-Vit-ABD | DQKPIVDKNDRDNRQAFEKISQLREEYANKAIKNPTKKNQYFSDFINKSN |
| Sequence92-P-CG-Vit-ABD | DQKPIVDKNDRDNRQAFEKISQLREEYANKAIKNPAKKNQYFSDFINKTN |
| Sequence49-NP-GA-Jp-ABC | DQKPIVDKNDRDNRQAFEKISQLREEFANKAIKNPAKKNQYFSNFISKSS |
| Sequence181-P-CG-Col-ABCC | DQKPIVDKNDRDNRQAFDGISQLREEYSNKAIKNPTKKNQYFSDFINKIN |
| Sequence25-NP-GA-Jp-ABD | DQKPIVDKNDRDNRQAFEKISQLREEYANKAIKNPAKKNQYFSDFINKSN |
| Sequence4-P-CG-Jp-ABD | DQKPIVDKNDKDNRQAYEKISQLREEYANKAIKNPAKKNQYFSDFINKSN |
| Sequence121-NP-GA-Jp-ABCC | DQKPIVDKNDRDNRQAFDGISQLREEYSNKAIKNPTKKNQYFSDFINKSN |
| 4dvy | DQKPIVDKNDRDNRQAFDGISQLREEYSNKAIKNPTKKNQYFSDFIDKSN |
| Sequence186-NP-UP-Jp-ABD | DQKPIVDKNDRDNRQAFERISQLREEYANKAIKNPAKKNQYFSDFINKSN |
| Sequence62-NP-UD-OCC-ABD | DQKPIVDKNDRDNRQAFEKISQLREEYANKAIKNPAKKNQYFSNFISKSS |
| Sequence9-NP-GCA-Jp-ABD | DQKPIVDKNDRDNRQAFEKISQLMEEYANKAIKNPTKKNQYFSDFINKSN |
| Sequence12-NP-UP-Jp-ABD | DQKPIVDKNDRDNRQAFEKISQLREEYANKAIKNPAKKNQYFSDFINKSN |
| Sequence35-NP-GA-Jp-ABABD | DQKPIVDKNDRDNRQAFEKISQLREEYANKAIKNPAKKNQYFSDFINKSN |
| Sequence54-NP-GA-OCC-ABC | DQKPIVDKNDRDNRQAFEKISQLREEFANKAIKNPAKKNQYFSNFISKSN |
| Sequence109-P-CG-Jp-ABD | DQKPIVDKNDRDNRQAFEKISQLREEYANKAIKNPAKKNQYFSDFINKSN |
| Sequence96-NP-UD-Vit-ABD | DQKPIVDKNDRDNRQAFEKISQLREEYANKAIKNPAKKNQYFSDFINKSN |
| Sequence80-P-CG-EA-ABD | DQKPIVDKNDRDNRQAFEKISQLREEYANKAIKNPAKKNQYFSDFINKSN |
| Sequence82-P-CG-EA-ABD | DQKPIVDKNDRDNRQAFEKISQLREEYANKAIKNPAKKNQYFSDFINKSN |
| Sequence10-NP-GCA-Jp-ABD | DQKPIVDKNDRDNRHAFEKISQLREEYANKAIKNPTKKNQYFSDFINKSN |
| Sequence19-NP-UP-Jp-ABBD | DQKPIVDKNDRDNRQAFEKISQLREEYANKAIKNPAKKNQYFSDFINKSN |
| Sequence115-NP-UG-Jp-ABC | DQKPIVDKNDRDNRQAFDGISQLREEYSNKAIKNPTKKNQYFSDFINKSN |
| Sequence45-NP-GA-Jp-ABCC | DQKPIVDKNDRDNRQAFDGISQLREEYSNKAIKNPTKKNQYFSDFINKSN |
| Sequence119-NP-UG-Jp-ABC | DQKPIVDKNDRDNRQAFDGISQLREEYSNKAIKNPTKKNQYFSDFINKSN |
| Sequence70-NP-GA-Ch-ABD | DQKPIVDKNDRDNRQAFEKISQLREEYANKAIKNPAKKNQYFSDFINKSN |
| Sequence52-NP-UD-EA-ABD | DQKPIVDKNDRDNRQAFEKISQLREEYANKAIKNPTKKNQYFSDFINKSN |
| Sequence97-P-CG-Vit-ABD | DQKPIVDKNDRDNRQAFEKISQLREEYANKAIKNPAKKNQYFSDFINKSN |
| Sequence94-NP-UD-Vit-ABD | DQKPIVDKNDRDNRQAFEKISQLREEYANKAIKNPAKKNQYFSDFINKSN |
| Sequence176-NP-GA-Col-ABCC | DQKPIVDKNDRDNRQAFDGISQLREEYSNKAIKNPTKKNQYFSDFINKSN |
| Sequence68-NP-D-Am-ABBC | DQKPIVDKNDRDNRQAFDGISQLREEYSNKAIKNPAKKNQYFSSFISKSN |
| Sequence47-NP-GA-Jp-ABD | DQKPIVDKNDRDNRQAFEKISQLREEYANKAIKNPTKKNQYFSDFINKSN |
| Sequence56-NP-UD-OCC-ABC | DQKPIVDKNDRDNRQAFEKISQLREEFANKAIKNPAKKNQYFSNFISKSS |
| Sequence31-NP-GA-Jp-ABD | DQKPIVDKNDRDNRQAFEKISQLREEYANKAIKNPTKKNQYFSDFINKSN |
| Sequence46-NP-UD-Jp-ABD | DQKPIVDKNDRDNRQAFEKISQLREEFANKAIKNPAKKNQYFSNFISKSN |
| Sequence102-NP-UG-Vit-ABD | DQKPIVDKNDRDNRQAFEKISQLREEYSNKAIKNPTKKNQYFSDFINKSN |
| Sequence71-P-CG-EA-ABD | DQKPIVDKNDRDNRQAFEKISQLREEYANKAIKNPTKKNQYFSDFINKSN |
| Sequence30-NP-GA-Jp-ABBD | DQKPIVDKNDRDNRQAFEKISQLREEYANKAIKNPTKKNQYFSDFINKSN |
| Sequence28-NP-GA-Jp-AABD | DQKPIVDKNDRDNRQAFEKISQLREEYANKAIKNPTKKNQYFSDFINKSN |
| Sequence98-P-CG-Vit-AD | DQKPIVDKNDRDNRQAFEKISQLREEYANKAIKNPTKKNQYFSDFINKSN |
| Sequence99-P-CG-Vit-ABD | DQKPIVDKNDKDNRQAFEKISQLREEYANKAIKNPAKKNQYFSDFINKSN |
| Sequence24-NP-UD-USA-ABD | DQKPIVDKNDRDNRQAFEKISQLREEYANKAIKNPAKKNQYFSDFINKSN |
| Sequence124-NP-G-Jp-ABC | DQKPIVDKNDRDNRQAFGGISQLREEYSNKAIKNPTKKNQYFSDFINKSN |
| Sequence77-P-CG-OCC-ABCC | DQKPIVDKNDRDNRQAFEKISQLREEYANKAIKNPTKKNQYFSDFINKSN |
| Sequence113-NP-UG-Jp-ABC | DQKPIVDKNDRDNRQAFNGISQLREEYSNKAIKNPTKKNQYFSDFINKSN |
| Sequence88-NP-UD-Vit-ABD | DQKPIVDKNDKDNRQAFEKISQLREEYANKAIKNPAKKNQYFSDFINKSN |

```
Sequence100-NP-UD-Vit-ABD        DQKPIVDKNDRDNRQAFEKISQLREEYANKAIKNPAKKNQYFSDFINKSN
Sequence137-NP-G-Col-ABCC        DQKPIVDKNDRDNRQAFDGISQLREEYSNKAIKNPTKKNQYFSDFINKSN
Sequence117-NP-G-Jp-ABC          DQKPIVDKNDRDNRQAFDGISQLREEYSNKAIKNPTKKNQYFSDFINKSN
Sequence171-NP-UD-Col-ABCC       DQKPIVDKNDRDNRQAFDGISQLREEYSNKAIKNPTKKNQYFSDFINKSN
Sequence180-P-CG-Col-ABC         DQKPIVDKNDKDNRQAFDGISQLREEYSNKAIKNPTKKNQYFSDFINKSN
Sequence112-P-CG-Jp-ABCC         DQKPIVDKNDRDNRQAFDGISQLREEYSNKAIKNPTKKNQYFSDFINKSN
Sequence127-NP-UD-Jp-ABC         DQKPIVDKNDRDNRQAFDGISQLREEYSNKAIKNPTKKNQYFSDFINKSN
Sequence51-NP-GA-OCC-ABC         DQKPIVDKNDRDNRQAFEKISQLREEFANKAIKNPAKKNQYFSSFISKSS
Sequence73-P-CG-EA-ABD           DQKPIVDKNDRDNRQAFEKISQLREEYANKAIKNPTKKNQYFSDFINKSN
Sequence183-NP-UD-Col-ABD        DQKPIVDKNDRDNRQAFDGISQLREEYSNKAIKNPTKKNQYFSDFINKSN
Sequence34-NP-UD-Jp-ABDD         DQKPIVDKNDRDNRQAFDEISQLREEYSNKAIKNPTKKNQYFSDFINKSN
Sequence21-P-CG-Jp-ABD           DQKPIVDKNDRDNRQAFEKISQLREEYANKAIKNPAKKDQYFSDFINKSN
Sequence32-NP-GA-Jp-ABD          DQKPIVDKNDRDNRQAFEKISQLMEEYANKAIKNPTKKNQYFLDFINKSN
Sequence116-NP-UD-Jp-ABC         DQKPIVDKNDRDNRQAFDGISQLREEYSNKAIKNPTKKNQYFSDFINKSN
Sequence8-NP-GCA-Jp-ABD          DQKPIVDKNDRDNRQAFEKISQLREEYANKAIKNPTKKNQYFSDFINKSN
Sequence83-P-CG-EA-ABD           DQKPIVDKNDRDNRQAFEKISQLREEYANKAIKNPTKKNQYFSDFINKSN
Sequence48-NP-UD-OCC-ABC         DQKPIVDKNDRDNRQAFEKISQLREEFANKAIKNPAKKNQYFSNFISKSS
Sequence120-NP-G-Jp-ABC          DQKPIVDKNDRDNRKAFNGISQLREEYSNKAIKNPTKKNQYFSDFINKSN
Sequence187-NP-UG-Jp-ABCCC       DQKPIVDKNDRDNRQAFDGISQLREEYSNKAIKNPTKKNQYFSDFINKSN
Sequence118-NP-G-Jp-ABD          DQKPIVDKNDRDNRQAFEKISQLREEYANKAIKNPAKKNQYFSDFINKSN
Sequence87-NP-UG-Vit-ABD         DQKPIVDKNDRDNRQAFEKISQLREEYANKAIKNPAKKNQYFSDFINKSN

                                 101                                            150
Sequence140-NP-GA-Col-ABABC      DLINKDALIDVESSTKSFQKFGDQRY-RIFTSWVSHQNDPSKINTRSIRN
Sequence123-NP-UD-Jp-ABD         DLINKDNLIAVDSSVDSFKKFGDQRY-QIFTSWVSLQKDPSKINTQQIRN
Sequence111-NP-G-Jp-ABD          DLINKDNLIAVDSSVDSFKKFGDQRY-QIFTSWVSLQKDPSKINTQQIRN
Sequence57-NP-GA-OCC-AB          DLINKDSLIDTGSSIKSFQKFGTQRY-QIFMNWVSHQKDPSKINTQRIRS
Sequence172-P-CG-Col-ABCC        DLINKDNLIDVESSTKSFQKFGDQRY-RIFTSWVSHQNDPSKINTRSIRN
Sequence110-NP-UD-Jp-ABD         DLINKDNLIAVDSSVESFKKFGDQRY-QIFTSWVSLQKDPSKINTQQIRN
Sequence95-NP-UD-Vit-ABD         DLINKDNLIAVDSSVDSFKKFGDQRY-QIFTSWVSLQKDPSKINIQTIQN
Sequence107-P-CG-Vit-ABD         DLINKDNLIAVDSSVDSFKKFGDQRY-QIFTSWVSLQKDPSQINTRQIRN
Sequence106-P-CG-Vit-ABD         DLINKDALIAVNSSVDSFKKFGDQRY-QIFTSWVSLQKDPSKINTRSIRN
Sequence105-NP-UD-Vit-ABD        DLINKDNLIAVDSSVDSFKKFGDQRY-QMFTSWVSLQKDPSKINTQQIRN
Sequence126-NP-UG-Jp-ABC         DLINKDNLIDVESSTKSFQKFGDQRY-RIFTSWVSHQSDPSKINTRSIRN
Sequence135-P-CG-Col-ABCCC       ALINKDALIDVESSTKSFQKFGDQRY-QIFTSWVSHQNDPSKINTRSIRN
Sequence138-NP-GA-Col-ABC        DLINKDNLIDVESSTKSFQKFGDQRY-RIFTSWVSHQNDPSKINTRSIRN
Sequence13-NP-UP-Jp-ABD          DLINKDNLIAVDSSVESFRKFGDQRY-QIFTSWVSLQKDPSKINTQQIRN
Sequence11-NP-UP-Jp-ABD          DLINKDNLIAVDSSVESFRKFGDQRY-QIFTSWVSLQKDPSKINTQQIRN
Sequence50-NP-GA-OCC-ABC         DLINKDSLIDTGSSIKSFQKFGTQRY-QIFMNWVSHQKDPSKINTQRIRS
Sequence26-NP-GA-Jp-ABD          DLINKDNLIAVDSSVESFRKFGDQRY-QIFTSWVSLQKDPSKINTQQIQN
Sequence55-NP-UD-OCC-ABCCC       DLINKDSLIDTGSSIKSFQKFGTQRY-QIFMNWVSHQKDPSKINTQRIRS
Sequence178-NP-M-Col-ABCC        DLINKDALIDVESSTKSFQKFGDQRY-RIFTSWVSHQNDPSKINTRSIQN
Sequence40-NP-GA-Jp-ABD          DLINKDNLIAVDSSVESFRKFGDQRY-QIFTSWVSLQKDPSKINTQQIRD
Sequence43-NP-GA-Jp-ABD          DLINKDNLIAVDSSVESFRKFGDQRY-QIFTSWVSLQKDPSKINTQQIRY
Sequence139-NP-UD-Col-ABCC       DLINKDALIDVESSTKSFQKFGDQRY-RIFTSWVSHQNDPSKINTRSIRN
Sequence7-P-CG-Jp-ABBD           DLINKDNLIAVDSSVESFRKFGDQRY-QIFTSWVSLQKDPSKINTQQIRN
Sequence91-P-CG-Vit-ABD          DLINKDNLIAVDSSVDSFKKFGDQRY-QIFTSWVSLQKDPSEINTEQIRN
Sequence29-NP-GA-Jp-ABABD        DLINKDNLIAVDSSVESFRKFGDQRY-QIFTSWVSLQKDPSKINTQQIRN
Sequence78-P-CG-EA-ABD           DLINKDNLIAVDSSVESFRKFGDQRY-QIFTSWVSLQKDPSKINTKQIQN
Sequence188-NP-GCA-Jp-AB         DLINKDNLIDVESSTKSFQKFGDQRY-RIFTSWVSHQNDPSKINTRSIRN
Sequence72-P-CG-EA-ABD           DLINKDNLIAVDSSVESFKKFGDQRY-QIFTSWVSLQKDPSKINTQQIRD
Sequence79-P-CG-EA-ABD           DLINKDNLIAVDSSVESFQKFGDQRY-QIFTSWVSLQKDPSKINTQQIRD
Sequence61-NP-UD-OCC-ABC         DLINKDNLIAVDSSVESFRKFGDQRY-QIFTSWVSLQKDPSKINTQRIRD
Sequence125-NP-UG-Jp-ABCC        DLINKDALIDVESSTKSFQKFGDQRY-RIFTSWVSHQNDPSKINTRSIRN
Sequence175-NP-G-Col-ABCC        DLINKDNLIDVESSTKSFQKFGDQRY-RIFTSWVSHQNDPSKINTRSIRN
Sequence122-NP-G-Jp-ABCC         DLINKDNLIDVESSTKSFQKFGDQRY-RIFTSWVSHQNDPSKINTRSIRN
Sequence36-NP-GA-Jp-ABD          DLINKDNLIAVDSSVESFRKFGDQRY-QIFTSWVSLQKDPSKINTQQIRN
Sequence58-NP-UD-OCC-ABC         DLINKDSLIDPGSSIKSFQKFGTQRY-QIFMNWVSHQKDPSKINTQRIRS
Sequence27-NP-GA-Jp-ABBD         DLINKDNLIAVDSSVESFRKFGDQRY-QIFTSWVSLQKDPSEINTQQIRN
Sequence93-P-CG-Vit-ABD          DLINKDNLIAVDSSVDSFKKFGDQRY-QIFTSWVSLQKDPSKINTQQIRN
Sequence177-NP-M-Col-ABCC        DLINKDALIDVESSTKSFQKFGDQRY-RIFTSWVSHQNDPSKINTRSIRN
```

```
Sequence182-NP-UD-Col-ABCC    D L I N K D N L I D V E S S T E S F R K F G D Q R Y - R I F T S W V S H Q N D P S K I N T R S I R N
Sequence114-NP-UG-Jp-ABC      D L I N K D N L I D V E S S T K S F Q K F G D Q R Y - R I F T S W V S H Q N D P S K I N T R S I R N
Sequence74-P-CG-EA-ABD        D L I N K D N L I A V D S S V E S F R K F G D Q R Y - Q I F T S W V S L Q K D P S K I N T Q Q I Q N
Sequence20-P-CG-Jp-ABD        D L I N K D N L I A V D S S V E S F R K F G D Q R Y - Q I F T S W V S L Q K D P S K I N T Q Q I R N
Sequence14-NP-GCA-Jp-ABD      D L I N K D N L I A V D S S V E S F R K F G D Q R Y - L N F T S W V S L Q K D P S K I N T Q Q I Q N
Sequence39-NP-GA-Jp-ABC       E L I N K D N L I D V E S S T K S F Q K F G D Q R Y - R I F T S W V S H Q N D P S K I N T R S I R N
Sequence170-NP-UD-Col-ABCC    D L I N K D N L I D V E S S T K S F Q K F G D Q R Y - R I F T S W V S H Q N D P S K I N T R S I R N
Sequence76-P-CG-EA-ABD        D L I N K D N L I A V D S S V E S F Q K F G D Q R Y - Q I F T S W V S L Q K D P S K I N T Q Q I Q N
Sequence53-NP-GA-EA-ABD       D L I N K D N L I A V D S S V E S F R K F G D Q R Y - Q I F T S W V S L Q K D P S K I N T Q Q I R N
Sequence174-P-CG-Col-ABCC     D L I N K D N L I D V E S S T K S F Q K F G D Q R Y - Q I F T S W V S H Q N D P S K I N T R S T R N
Sequence33-NP-UG-Jp-ABD       D L I N K D N L I A V D S S V E S F Q K F G D Q R Y - Q I F T S W V S L Q N D P S K I N T R S I R N
Sequence60-NP-GA-EA-ABD       D L I N K D N L I A V D S S V E S F R K F G D Q R Y - Q I F T S W V S L Q K D P S K I N T Q Q I Q Y
Sequence38-NP-GA-Jp-ABD       D L I N K D N L I A V D S S V E S F R K F G D Q R Y - Q I F T S W V S L Q K D P S K I N T Q Q I R N
Sequence1-NP-EG-Jp-ABCCC      D L I N K D N L I D I G S S I K S F Q K F G T Q R Y - R I F T S W V S H Q N D P S K I N T R S I R N
Sequence101-P-CG-Vit-ABD      D L I N K D N L I A V D S S V D S F K K F G D Q R Y - Q I F T S W V S L Q K D P S K I N T Q Q I R N
Sequence64-NP-UD-OCC-ABC      D L I N K D T L I D T G S S I K S F Q K F G T Q R Y - Q I F M N W V S H Q K D P S K I N T Q R I R S
Sequence81-P-CG-EA-ABD        D L I N K D N L I A V D S S V E S F R K F G D Q R Y - Q I F T S W V S L Q K D P S K I D T Q Q I R N
Sequence150-NP-DA-Sen-BC      D L I N K D A L I D V E S S T K S F Q K F G D Q R Y - R I F T S W V S H Q N D P S K I N T R S I R N
Sequence90-NP-UD-Vit-ABD      D L I N K D N L I A V D S S V E S F K K F G D Q R Y - Q I F T S W V S L Q K D P S K I N T Q Q I Q N
Sequence136-P-CG-Col-AACC     D L I N K D A L I D V E S S T K S F Q K F G D Q R Y - Q I F T S W V S H Q N D P S K I N T R S I R N
Sequence15-NP-UP-Jp-ABD       D L I N K D N L I A V D S S V E S F R K F G D Q R Y - Q I F T S W V S L Q K D P S K I N I Q Q I R N
Sequence108-NP-UD-Vit-ABD     D L I N K D N L I A V D S S V D S F K K F G D Q R Y - Q I F T S W V S L Q K D P S K I N T Q Q I R N
Sequence104-P-CG-OCC-ABCC     D L I N K D A L I D V E S S T K S F Q K F G D Q R Y - R I F T S W V S H Q N D P S K I N T R S I R N
Sequence179-NP-UD-Col-ABCC    N L I N K D A L I D V E S S T K S F Q K F G D Q R Y - R I F T S W V S H Q N D P S K I N T R S I R N
Sequence37-NP-GA-Jp-ABCCC     D L I N K D S L I D I G S S T K S F Q K F G T Q R Y - Q I F M N W V S H Q K D P S K I N T Q R I R S
Sequence89-NP-UD-Vit-ABD      D L I N K D N L I A V D S S V D S F K K F G D Q R Y - Q I F T S W V S L Q K D P S K I N T Q Q I R N
Sequence92-P-CG-Vit-ABD       D L I N K D N L I A V D S S V D S F K K F G D Q R Y - Q I F T S W V S L Q K D P S E I N T E Q I R N
Sequence49-NP-GA-Jp-ABC       D L I N K D S L I D T G S S I K S F Q K F G T Q R Y - Q I F M N W V S H Q K D P S K I N T Q R I R S
Sequence181-P-CG-Col-ABCC     D L I N K D N L I D V E S S T K S F Q K F G D Q R Y - Q I F I S W V S H Q N D P S K I N T R S I R N
Sequence25-NP-GA-Jp-ABD       D L I N K D N L I A V D S S V E S F R K F G D Q R Y - Q I F T S W V S L Q K D P S K I N T Q Q I Q D
Sequence4-P-CG-Jp-ABD         D L I N K D N L I A V D S S V E S F R K F G D Q R Y - Q I F T S W V S L Q K D P S K I N T Q Q I R N
Sequence121-NP-G-Jp-ABCC      D L I N K D N L I D V E S S T K S F Q K F G D Q R Y - R I F T S W V S H Q S D P S K I N T R S I R N
4dvy                          D L I N K D N L I D V E S S T K S F Q K F G D Q R Y - Q I F T S W V S M Q K D P S K I N T R S I R N
Sequence186-NP-UP-Jp-ABD      D L I N K D N L I A V D S S V E S F R K F G D Q R Y - Q I F T S W V S L Q K D P S K I N T Q Q I R N
Sequence62-NP-UD-OCC-ABD      D L I N K D S L I D T G S S I K S F Q K F G T Q R Y - Q I F M N W V S H Q K D P S K I N T Q R I R S
Sequence9-NP-GCA-Jp-ABD       D L I N K D N L I A V D S S V E S F R K F G D Q R Y - Q I F T S W V S L Q K D P S K I N T Q Q I R N
Sequence12-NP-UP-Jp-ABD       D L I N K D N L I A V D S S V E S F R K F G D Q R Y - Q I F T S W V S L Q K D P S K I N T Q Q I R N
Sequence35-NP-GA-Jp-ABABD     D L I N K D N L I A V D S S V E S F R K F G D Q R Y - Q I F T S W V S L Q K D P S K I N T Q Q I R N
Sequence54-NP-GA-OCC-ABC      D L I N K D S L I D I G S S T K S F Q K F G T Q R Y - Q I F M D W V S H Q K D P S K I N T Q R I R S
Sequence109-P-CG-Jp-ABD       D L I N K D N L I A V D S S V D S F K K F G D Q R Y - Q I F T S W V S L Q K D P S K I N T Q Q I R N
Sequence96-NP-UD-Vit-ABD      D L I N K D N L I A V D S S V D S F K K F G D Q R Y - Q I F T S W V S L Q K D P S K I N T Q Q I Q N
Sequence80-P-CG-EA-ABD        D L I N K D N L I A V D S S V E S F R K F G D Q R Y - Q I F T S W V S L Q K D P S K I N T Q Q I R D
Sequence82-P-CG-EA-ABD        D L I N K D N L I A V D S S V D S F K K F G D Q R Y - Q I F T S W V S L Q K D P S K I N T Q Q I R N
Sequence10-NP-GCA-Jp-ABD      D L I N K D N L I A V D S S V E S F R K F G D Q R Y - Q I F T S W V S L Q K D P S K I N T Q Q I R N
Sequence19-NP-UP-Jp-ABBD      D L I N K D N L I A V D S S V E S F G K F G D Q R Y - Q I F T S W V S L Q K D P S E I N T Q Q I R N
Sequence115-NP-UG-Jp-ABC      D L I N K D A L I D V E S S T K S F Q K F G N Q R Y - R I F T T W V S H Q N D P S K I N T R S I R N
Sequence45-NP-GA-Jp-ABCC      D L I N K D N L I D V E S S T K S F Q K F G D Q R Y - R I F T S W V S H Q N D P S K I N T R S I R N
Sequence119-NP-UG-Jp-ABC      D L I N K D A L I D V E S S T K S F Q K F G D Q R Y - R I F T S W V S H Q N D P S K I N T R S I R N
Sequence70-NP-GA-Ch-ABD       D L I N K D N L I A V D S S V D S F K K F G D Q R Y - Q I F T S W V S L Q K D P S E I N T Q Q I R N
Sequence52-NP-UD-EA-ABD       D L I N K D N L I A V D S S V E S F R K F G D Q R Y - Q I F T S W V S L Q K D P S K I N T Q Q I R N
Sequence97-P-CG-Vit-ABD       D L I N K D N L I A V D S S V D S F K K F G D Q R Y - Q I F T S W V S L Q K D P S E I N T Q Q I R N
Sequence94-NP-UD-Vit-ABD      D L I D K D N L I A V D S C V D S F K K F G D Q R Y - Q I F T S W V S L Q K D P S K I N T Q Q I R N
Sequence176-NP-GA-Col-ABCC    D L I N K D A L I D V E S S T K S F Q K F G D Q R Y - R I F T S W V S H Q N D P S K I N T R S I R N
Sequence68-NP-D-Am-ABBC       D L I N K D N L I D V D S S T K S F Q K F G D Q R Y - Q I F M D W V S H Q N D P S K I N T R S I R N
Sequence47-NP-GA-Jp-ABD       D L I N K D N L I A V D S S V E S F R K F G D Q R Y - Q I F T S W V S L Q K D P S K I N T Q Q I R N
Sequence56-NP-UD-OCC-ABC      D L I N K D S L I D T G S S I K S F Q K F G T Q R Y - Q I F M N W V S H Q K D P S K I N T Q R I R S
Sequence31-NP-GA-Jp-ABD       D L I N K D N L I A V D S S V E S F R K F G D Q R Y - Q I F T S W V S L Q K D P S K I N T Q Q I R N
Sequence46-NP-UD-Jp-ABD       D L I D K D S L I D I G S S I K S F Q K F G T Q R Y - Q I F M N W V S H Q K D P S K I N T Q R I R S
Sequence102-NP-UG-Vit-ABD     D L I N K D N L I A V D S S V D S F K K F G D Q R Y - Q I F T S W V S L Q K D P S K I N T Q Q I R N
Sequence71-P-CG-EA-ABD        D L I N K D N L I A V D S S V E S F R K F G D Q R Y - Q I F T S W V S L Q K D P S K I N T Q Q I Q N
Sequence30-NP-GA-Jp-ABBD      D L I N K D N L I A V D S S V D S F K K F G D Q R Y - Q I F T S W V S L Q K D P S K I N T Q Q I Q N
```

Sequence28-NP-GA-Jp-AABD D L I N K D N L I A V D S S V E S F R K F G D Q R Y - Q I F T S W V S L Q K D P S K I N T Q Q I R N
Sequence98-P-CG-Vit-AD D L I N K D N L I A V D S S V D S F K K F G D Q R Y - Q I F T S W V S L Q K D P S K I N T Q T I R N
Sequence99-P-CG-Vit-ABD D L I N K D N L I A V D S S V D S F K K F G D Q R Y - Q I F T S W V S L Q K D P S Q I N A Q Q I R N
Sequence24-NP-UD-USA-ABD D L I N K D N L I A V D S S V E S F R K F G D Q R Y - Q I F T S W V S L Q K D P S K I N T Q Q I Q N
Sequence124-NP-G-Jp-ABC D L I N K D N L I D V E S S V K S F Q K F G D Q R Y - R I F T S W V S H Q S D P S K I N T R S I R N
Sequence77-P-CG-OCC-ABCC D L I N K D N L I A V D S S V E S F R K F G D Q R Y - Q I F T S W V S L Q K D P S K I N T Q K I R D
Sequence113-NP-UG-Jp-ABC D L I N K D N L I D V E S S T K S F Q K F G D Q R Y - R I F T S W V S H Q N D P S K I N T R S I R N
Sequence88-NP-UD-Vit-ABD D L I N K D N L I A V D S S V D S F K K F G D T A L P N P L R V G C P F K K I R L K S T P K Q Y R N
Sequence100-NP-UD-Vit-ABD D L I N K D N L I A V N S S V D S F K K F G D Q R Y - Q I F T S W V S L Q K D P S K I N T Q Q I R N
Sequence137-NP-G-Col-ABCC D L I N K D N L I D V E S S T K S F Q K F G D Q R Y - R I F T S W V S H Q N D P S K I N T R S I R N
Sequence117-NP-G-Jp-ABC D L I N K D A L I D V E S S T K S F Q K F G D Q R Y - R I F T S W V S H Q N D P S K I N T R S I R N
Sequence171-NP-UD-Col-ABCC D L I N K D N L I D V E S S T K S F Q K F G D Q R Y - R I F T S W V S H Q N D P S K I N T R S I R N
Sequence180-P-CG-Col-ABC D L I N K D N L I D V E S S T K S F Q K F G D Q R Y - R I F T S W V S H Q N D P S K I N T R S I Q N
Sequence112-P-CG-Jp-ABCC D L I N K D N L I D V E S S T E S F R K F G D Q R Y - R I F T S W V S H Q N D P S K I N T R S I R N
Sequence127-NP-UD-Jp-ABC D L I N K D A L I D V E S S T K S F Q K F G D Q R Y - R I F T S W V S H Q N D P S K I N T R S I R N
Sequence51-NP-GA-OCC-ABC D L I N K D S L I D T G S S I K S F Q K F G T Q R Y - Q I F M N W V S H Q K D P S K I N T Q R I R S
Sequence73-P-CG-EA-ABD D L I N K D N L I A V D S S V E S F R K F G D Q R Y - Q I F T S W V S L Q K D P S K I N T Q Q I R N
Sequence183-NP-UD-Col-ABD D L I N K D A L I D V E S S T K S F Q K F G D Q R Y - R I F T S W V S H Q N D P S K I N T R S T R N
Sequence34-NP-UD-Jp-ABDD N L I N K D A L I D V E S S T K S F Q K F G D Q R Y - R I F T S W V S H Q N D P S K I N T R S I R N
Sequence21-P-CG-Jp-ABD D L I N K D N L I A V D S S V E S F R K F G D Q R Y - Q I F T S W V S L Q K D P S K I N T Q Q I Q N
Sequence32-NP-GA-Jp-ABD D L I N K D N L I A V D S S V E S F R K F G D Q R Y - Q I F T S W V S L Q K D P S K I N T Q Q I R N
Sequence116-NP-UD-Jp-ABC D L I N K D A L I D V E S S T K S F Q K F G N Q R Y - R I F T T W V S H Q N D P S K I N T R S I R N
Sequence8-NP-GCA-Jp-ABD D L I N K D N L I A V D S S V E S F Q K F G D Q R Y - Q I F T S W V S L Q K D P S K I N T Q Q I R N
Sequence83-P-CG-EA-ABD D L I N K D N L I A V D S S V E S F R K F G D Q R Y - Q I F T S W V S L Q K D P S K I N T Q Q I R D
Sequence48-NP-UD-OCC-ABC D L I N K D S L I D T G S S I K S F Q K F G T Q R Y - Q I F M N W V S H Q K D P S K I N T Q R I R S
Sequence120-NP-G-Jp-ABC D L I N K D N L I D V E S S T K S F Q K F G D Q R Y - R I F T S W V S H Q N D P S K I N T R S I R N
Sequence187-NP-UG-Jp-ABCCC D L I N K D N L I D I G S S I K S F Q K F G T Q R Y - R I F T S W V S H Q N D P S K I N T R S I R N
Sequence118-NP-G-Jp-ABD D L I N K D N L I A V D S S V E S F K K F G D Q R Y - Q I F T S W V S L Q K D P S K I N T Q Q I R N
Sequence87-NP-UG-Vit-ABD D L I N K D N L I D V N S S V G S F K K F G D Q R Y - Q I F T S W V S L Q K D P S K I N T Q Q I R N

151 … 200

Sequence140-NP-GA-Col-ABABC F M E N I I Q P P I S D D K E K A E F L K S A K Q S F A G I I I G N Q I R T D Q K F M G V - F D E S
Sequence123-NP-UD-Jp-ABD F M E N I I Q P P I S D D K E K A E F L R S A K Q S F A G I I I G N Q I R S D Q K F M G V - F D E S
Sequence111-NP-G-Jp-ABD F M E N I I Q P P I S D N K E K A E F L R S A K Q S F A G I I I G N Q I R S D Q K F M G V - F D E S
Sequence57-NP-GA-OCC-AB F M E N I I Q P P I S D D K E K A E F L R S A K Q A F A G I I I G N Q I Q S D Q K F M G V - F E E S
Sequence172-P-CG-Col-ABCC F M E N I I Q P P I H D D K E K A E F L K S A K Q S F A G I I I G N Q I R T D Q K F M G V - F D E S
Sequence110-NP-UD-Jp-ABD F M E N I I Q P P I S D D K E K A E F L R S A K Q S F A G I I I G N Q I R S D Q K F M G V - F D E S
Sequence95-NP-UD-Jp-ABD F M E N I I Q P P I S D D K E K A E F L R S A K Q S F A G I I I G N Q I R S D Q K F M G V - F D E S
Sequence107-P-CG-Vit-ABD F M E N I I Q P P I S D D K E K A E F L R S A K Q S F A G I I I G N Q I Q S D Q K F M G V - F D E P
Sequence106-P-CG-Vit-ABD F M E N I I Q P P I S D D K E N S R V F E I C Q T I F C R N H Y R E S N P N G S K V H G S Y F T E S
Sequence105-NP-UD-Vit-ABD F M E N I I Q P P I S D D K E K A E F L R S A K Q S F A G I I I G N Q I R S D Q K F M G V - F D E S
Sequence126-NP-UG-Jp-ABC F M E H T I Q P P I P D D K E K A E F L K S A K Q S F A G I I I G N Q I R T D Q K F M G V - F D E S
Sequence135-P-CG-Col-ABCCC F M E N I I Q P P I P D D K E K A E F L K S A K Q S F A G I I I G N Q I R T D Q K F M G V - F D G S
Sequence138-NP-GA-Col-ABC F M E H T I Q P P I P D D K E K A E F L K S A K Q S F A G I I I G N Q I R T D Q K F M G V - F D E S
Sequence13-NP-UP-Jp-ABD F M E N I I Q P P I S D D K E K A E F L R S A K Q S F A G I I I G N Q I R S D E K F M G V - F D E S
Sequence11-NP-UP-Jp-ABD F M E N I I Q P P I S D D K E K A E F L R S A K Q S F A G I I I G N Q I R S D E K F M G V - F D E S
Sequence50-NP-GA-OCC-ABC F M E N I I Q P P I S D D K E K A E F L R S A K Q A F A G I I I G N Q I Q S D Q K F M G V - F E E S
Sequence26-NP-GA-Jp-ABD F M E N I I Q P P I S D D K E K A E F L R S A K Q S F A G I I I G N Q I R S D Q K F M G V - F D E F
Sequence55-NP-UD-OCC-ABCCC F M E N I I Q P P I S D D K E K A E F L R S A K Q A F A G I I I G N Q I Q S D Q K F M G V - F E E S
Sequence178-NP-M-Col-ABCC F M E H T I Q P P I P D D K E K A E F L K S A K Q S F A G I I I G N Q I R T D Q K F M G V - F D E S
Sequence40-NP-GA-Jp-ABD F M E N I I Q P P I S D D K E K A E F L R S A K Q S F A G I I I G N Q I R S D E K F M G V - F D E S
Sequence43-NP-GA-Jp-ABD F M E N I I Q P P I S D D K E K A E F L R S A K Q S F A G I I I G N Q I R S D E K F M G V - F D E S
Sequence139-NP-UD-Col-ABCC F M G N I I Q P P I S D D K E K A E F L K S A K Q S F A G I I I G N Q I R T D Q K F M G V - F D E S
Sequence7-P-CG-Jp-ABBD F M E N I I Q P P I S D D K E K A E F L R S A K Q S F A G I I I G N Q I R S D E K F M G V - F D E S
Sequence91-P-CG-Vit-ABD F M E N I I Q P P I S D D K E K A E F L R S A K Q S F A G I I I G N Q I R S D Q K F L G V - V D E S
Sequence29-NP-GA-Jp-ABABD F M E N I I Q P P I S D D K E K A E F L R S A K Q S F A G I I I G N Q I R S D E K F M G V - F D E S
Sequence78-P-CG-EA-ABD F M E N I I Q P P I S D D K E K A E F L R S A K Q S F A G I I I G N Q I R S D E K F M G V - F D E S
Sequence188-NP-GCA-Jp-AB F M E N I I Q P P I L D D K E K A E F L K S A K Q S F A G I I I G N Q I R T D Q K F M G V - F D E S
Sequence72-P-CG-EA-ABD F M E N I I Q P P I S D D K E K A E F L R S A K Q S F A G I I I G N Q I R S D E K F M G V - F D E S
Sequence79-P-CG-EA-ABD F M E N I M Q P P I S D D K E K A E F L R S A K Q S F V G I I I G N Q I R S D E K F M G V - F D E F
Sequence61-NP-UD-OCC-ABC F M E N I I Q P P I S D D K E K A E F L R S A K Q S F A G I I I G N Q I R S D E K F M G V - F D E S

| | |
|---|---|
| Sequence125-NP-UG-Jp-ABCC | FMENIIQPPISDDKEKAEFLKSAKQSFAGIIIGNQIRTDQKFMGV-FDES |
| Sequence175-NP-G-Col-ABCC | FMENIIQPPIPDDKEKAEFLKSAKQSFAGIIIGNQIRTDQKFMGV-FDES |
| Sequence122-NP-G-Jp-ABCC | FMENIIQPPIPDDKEKAEFLKSAKQSFAGIIIGNQIRTDQKFMGV-FDES |
| Sequence36-NP-GA-Jp-ABD | FMENIIQPPISDDKEKAEFLRSAKQSFAGIIIGNQIRSDEKFMGV-FDES |
| Sequence58-NP-UD-OCC-ABC | FMENIIQPPIFDDKEKAEFLRSAKQAFAGIIIGNQIQSDQKFMGV-FEES |
| Sequence27-NP-GA-Jp-ABBD | FMENIIQPPISDDKEKAEFLRSAKQSFAGIIIGNQIRSDEKFMGV-FDES |
| Sequence93-P-CG-Vit-ABD | FMENVIQPPISDDKEKAEFLRSAKQSFAGIIIGNQIRSDEKFMGV-FDES |
| Sequence177-NP-M-Col-ABCC | FMENIIQPPISDDKEKAEFLKSAKQSFAGIIIGNQIRTDQKFMGV-FDES |
| Sequence182-NP-UD-Col-ABCC | FMEHTIQPPIPDDKEKAEFLKSAKQSFAGIIIGNQIRTDQKFMGV-FDES |
| Sequence114-NP-UG-Jp-ABC | FMENIIQPPIPDDKEKAEFLKSAKQSFAGIIIGNQIRTDQKFMGV-FDES |
| Sequence74-P-CG-EA-ABD | FMENIIQPPISDDKEKAEFLRSAKQSFAGIIIGNQIRSDQKFMGV-FDES |
| Sequence20-P-CG-Jp-ABD | FMENIIQPPISDDKEKAEFLRSAKQSFAGIIIGNQIRSDEKFMGV-FDES |
| Sequence14-NP-GCA-Jp-ABD | FMENIIQPPISDDKEKAEFLRSAKQSFAGIIIGNQIRSDEKFMGV-FDES |
| Sequence39-NP-GA-Jp-ABC | FMEHTIQPPIPDDKEKAEFLKSAKQSFAGIIIGNQIRTDQKFMGV-FDES |
| Sequence170-NP-UD-Col-ABCC | FMEHTIQPPIPDDKEKAEFLKSAKQSFAGIIIGNQIRTDQKFMGV-FDES |
| Sequence76-P-CG-EA-ABD | FMENIIQPPISDDKEKAEFLRSAKQSFAGIIIGNQIRSDEKFMGV-FDES |
| Sequence53-NP-GA-EA-ABD | FMENIIQPPISDDKEKAEFLRSAKQSFAGIIIGNQIRSDEKFMGV-FDES |
| Sequence174-P-CG-Col-ABCC | FMEHTIQPPIPDDKEKAEFLKSAKQSFAGIIIGNQIRTDQKFMGV-FDES |
| Sequence33-NP-UG-Jp-ABD | FMEHTIQPPIPDDKEKAEFLKSAKQSFAGIIIGNQIRTDQKFMDV-FDES |
| Sequence60-NP-GA-EA-ABD | FMENIIQPPISDDKEKAEFLRSAKQSFAGIIIGNQIRSDEKFMGV-FDES |
| Sequence38-NP-GA-Jp-ABD | FMENIIQPPISDDKEKAEFLRSAKQSFAGIIIGNQIRSDEKFMGV-FDEF |
| Sequence1-NP-EG-Jp-ABCCC | FMENIIQPPTPDDKERAEFVKSAKQSFAGIIIGNQIRTDQKFMGV-FDEF |
| Sequence101-P-CG-Vit-ABD | FMENIIQPPISDDKEKAEFLRSAKQSFAGIIIGNQIRSDQKFMGV-FDES |
| Sequence64-NP-UD-OCC-ABC | FIENIIQPPISDDKEKAEFLRSAKQAFAGIIIGNQIQSDQKFMGV-FDES |
| Sequence81-P-CG-EA-ABD | FMENIIQPPISDDKEKAEFLRSAKQSFAGIIIGNQIRSDEKFMGV-FDES |
| Sequence150-NP-DA-Sen-BC | FMENIIQPPIPDDKEKAEFLKSAKQSFAGIIIGNQIRTDQKFMGV-FDES |
| Sequence90-NP-UD-Vit-ABD | FMENIIQPPISDDKEKAEFLRSAKQSFAGIIIGNQIRSDEKFMGV-FDVS |
| Sequence136-P-CG-Col-AACC | FMEHTIQPPIPDDKKKAQFLKSAKQSFAGIIIGNQIRTEQKFMGV-FDES |
| Sequence15-NP-UP-Jp-ABD | FMENIIQPPISDDKEKAEFLRSAKQSFAGIIIGNQIRSDEKFMGV-FDES |
| Sequence108-NP-UD-Vit-ABD | FMENIIQPPIPDDKEKAEFLRSAKQSFAGIIIGNQIRSDQKFMGV-FDES |
| Sequence104-P-CG-OCC-ABCC | FMENIIQPPISDDKEKAEFLKSAKQSFAGIIIGNQIRTDQKFMGV-FEES |
| Sequence179-NP-UD-Col-ABCC | FMENIIQPPIPDDKEKAEFLKSAKQSFAGIIIGNQIRTDQKFMGV-FDES |
| Sequence37-NP-GA-Jp-ABCCC | FMENIIQPPISDDKEKAEFLRSAKQSFAGIIIGNQIQSDQKFMGV-FEES |
| Sequence89-NP-UD-Vit-ABD | FMENIIQPPISDDKEKAEFLRSAKQSFAGIIIGNQIRSDQKFMGV-FDES |
| Sequence92-P-CG-Vit-ABD | FMENIIQPPISDDKEKAEFLRSAKQSFAGIIIGNQIRSDQKFMGV-FDES |
| Sequence49-NP-GA-Jp-ABC | FMENIIQPPISDDKEKAEFLRSAKQAFAGIIIGNQIRSDEKFMGV-FDES |
| Sequence181-P-CG-Col-ABCC | FMEHTIQPPIPDDKEKAEFLKSAKQSFAGIIIGNQIRTDQKFMGV-FDES |
| Sequence25-NP-GA-Jp-ABD | FMENIIQPPISDDKEKAEFLRSAKQSFAGIIIGNQIRSDEKFMGV-FDES |
| Sequence4-P-CG-Jp-ABD | FMENVIKPPISDDKEKAEFLRSAKQSFAGIIIGNQIRSDEKFMGV-FDES |
| Sequence121-NP-G-Jp-ABCC | FMEHTIQPPIPDDKEKAEFLKSAKQSFAGIIIGNQIRTDQKFMGV-FDES |
| 4dvy | FMENIIQPPIPDDKEKAEFLKSAKQSFAGIIIGNQIRTDQKFMGV-FDES |
| Sequence186-NP-UP-Jp-ABD | FMENIIQPPISDDKEKAEFLRSAKQSFAGIIIGNQIRSDEKFMGV-FDES |
| Sequence62-NP-UD-OCC-ABD | FMENIIQPPISDDKEKAEFLRSAKQAFAGIIIGNQIQSDQKFMGV-FDES |
| Sequence9-NP-GCA-Jp-ABD | FMENIIQPPISDDKEKAEFLRSAKQSFAGIIIGNQIRSDEKFMGV-FDES |
| Sequence12-NP-UP-Jp-ABD | FMENIIQPPISDDKEKAEFLRSAKQSFAGIIIGNQIRSDEKFMGV-FDES |
| Sequence35-NP-GA-Jp-ABABD | FMENIIQPPISDDKEKAEFLRSAKQSFAGIIIGNQIRSDQKFMGV-FDES |
| Sequence54-NP-GA-OCC-ABC | FMENIIQPPISDDKEKAEFLRSAKQSFAGIIIGNQIQSDQKFMGV-FDES |
| Sequence109-P-CG-Jp-ABD | FMENIIQPPISDDKEKAEFLRSAKQSFAGIIIGNQIRSDQKFMGV-FDES |
| Sequence96-NP-UD-Vit-ABD | FMENIIQPPISDDKEKAEFLRSAKQSFAGIIIGNQIRSDQKFMGV-FDES |
| Sequence80-P-CG-EA-ABD | FMENIIQPPISDDKEKAEFLRSAKQSFAGIIIGNQIRSDEKFMGV-FDES |
| Sequence82-P-CG-EA-ABD | FMENIIQPPISDDKEKAEFLRSAKQSFAGIIIGNQIRSDEKFMGV-FDES |
| Sequence10-NP-GCA-Jp-ABD | FMENIIQPPISDDKEKAEFLRSAKQSFAGIIIGNQIRSDEKFMGV-FDES |
| Sequence19-NP-UP-Jp-ABBD | FMENIIQPPISDDKEKAEFLRSAKQSFAGIIIGNQIRSDEKFMGV-FDES |
| Sequence115-NP-UG-Jp-ABC | FMENIIQPPIHDDKEKAEFLKSAKQSFAGIIIGNQIRTDQKFMGV-FDES |
| Sequence45-NP-GA-Jp-ABCC | FMENIIQPPIPDDKEKAEFLKSAKQSFAGIIIGNQIRTDQKFMGV-FDES |
| Sequence119-NP-UG-Jp-ABC | FMENIIQPPIPDDKEKAEFLKSAKQSFAGIIIGNQIRTDQKFMGV-FDES |
| Sequence70-NP-GA-Ch-ABD | FMENIIQPPISDDKEKAEFLRSAKQSFAGIIIGNQIRSDEKFMGV-FDES |
| Sequence52-NP-UD-EA-ABD | FMENIIQPPIFDDKEKAEFLRSAKQSFAGIIIGNQIRSDEKFMGV-FDES |
| Sequence97-P-CG-Vit-ABD | FMENIIQPPISDDKEKAEFLRSAKQSFAGIIIGNQIRSDQKFMGV-FDES |
| Sequence94-NP-UD-Vit-ABD | FMENIIQPPISDDKEKAEFLRSAKQSFAGIIIGNQIRSDEKFIGV-FDES |
| Sequence176-NP-GA-Col-ABCC | FMEHTIQPPIPDDKEKAEFLKSAKQSFAGIIIGNQIRTDQKFMGV-FDES |

| | |
|---|---|
| Sequence68-NP-D-Am-ABBC | FMENIIQPPIPDDKEKAEFLRSAKQSFAGIIIGNQIRTDQKFMGV-FDES |
| Sequence47-NP-GA-Jp-ABD | FMENIIQPPISDDKEKAEFLRSAKQSFAGIIIGNQIRSDEKFMGV-FDES |
| Sequence56-NP-UD-OCC-ABC | FMENIIQPPISDDKEKAEFLRSAKQAFAGIIIGNQIQSDQKFMGV-FEES |
| Sequence31-NP-GA-Jp-ABD | FMENIIQPPISDDKEKAEFLRSAKQSFAGIIIGNQIRSDEKFMGV-FDES |
| Sequence46-NP-UD-Jp-ABD | FMENIIQPPISDDKEKAEFLRSAKQTFAGIIIGNQIQSDQKFMGV-FEES |
| Sequence102-NP-UG-Vit-ABD | FMENIIQPPISDDKEKAEFLRSAKQSFAGIIIGNQIRSDQKFMGV-FDES |
| Sequence71-P-CG-EA-ABD | FMENIIQPPISDDKEKAEFLRSAKQSFAGIIIGNQIRSDEKFMGV-FDEF |
| Sequence30-NP-GA-Jp-ABBD | FMENIIQPPISDDKEKAEFLRSAKQSFAGIIIGNQIRSDEKFMGV-FDES |
| Sequence28-NP-GA-Jp-AABD | FMENIIQPPISDDKEKAEFLRSAKQSFAGIIIGNQIRSDEKFMGV-FDES |
| Sequence98-P-CG-Vit-AD | FMENIIQPPISDDKEKAEFLRSAKQSFAGIIIGNQIRSDQKFMGV-FDES |
| Sequence99-P-CG-Vit-ABD | FMENIIQPPISDDKEKAEFLRSAQQSFAGIIRGNQIRSDQKFRGV-FDES |
| Sequence24-NP-UD-USA-ABD | FMENIIQPPISDDKEKAEFLRSVKQSFAGIIIGNQIRSDQKFMGV-FDES |
| Sequence124-NP-G-Jp-ABC | FMEHTIQPPIPDDKEKAEFLKSAKQSFAGIIIGNQIRTDQKFMGV-FDES |
| Sequence77-P-CG-OCC-ABCC | FMENIIQPPISDDKEKAEFLRSAKQSFAGIIIGNQIRSDQKFMGV-FDES |
| Sequence113-NP-UG-Jp-ABC | FMENIIQPPIPDDKEKAEFLKSAKQSFAGIIIGNQIRTDQKFMGV-FDES |
| Sequence88-NP-UD-Vit-ABD | FMENIIQPPISDDKEKAEFLRSAKQSFAGIIIGNQIRSDQKFMGV-FDES |
| Sequence100-NP-UD-Vit-ABD | FMENIIQPPISDDKEKAEFLRSAKQSFAGIIIGNQIRSDQKFMGV-FDES |
| Sequence137-NP-G-Col-ABCC | FMENIIQPPIHDDKEKAEFLKSAKQSFAGIIIGNQIRTDQKYRGV-FDES |
| Sequence117-NP-G-Jp-ABC | FMENIIQPPISDDKEKAEFLKSAKQSFAGIIIGNQIRTDQKFMGV-FDES |
| Sequence171-NP-UD-Col-ABCC | FMEYTIQPPIPDDKEKAEFLKSAKQSFAGIIIGNQIRTDQKFMGV-FDES |
| Sequence180-P-CG-Col-ABC | FMENIIQPPIPDAKEKAEFLKSAKQSFAGIIIGNQIRTDQKFMGV-FDES |
| Sequence112-P-CG-Jp-ABCC | FMEHTIQPPIPDDKEKAEFLKSAKQSFAGIIIGNQIRTDQKFMGV-FDES |
| Sequence127-NP-UD-Jp-ABC | FMENIIQPPISDDKEKAEFLKSAKQSFAGIIIGNQIQTDQKFMGV-FEES |
| Sequence51-NP-GA-OCC-ABC | FIENIIQPPISDDKEKAEFLRSAKQAFAGIIIGNQIQSDQKFMGV-FDES |
| Sequence73-P-CG-EA-ABD | FMENIIQPPISDDKEKAEFLRSAKQSFAGIIIGNQIRSDEKFMGV-FDES |
| Sequence183-NP-UD-Col-ABD | FMEHTIQPPIPDDKEKAEFLKSAKQSFAGIIIGNQIRTDQKFMGV-FDES |
| Sequence34-NP-UD-Jp-ABDD | FMENIIQPPISDDKEKAEFLKSAKQAFAGIIIGNQIRSDQKFMGV-FDES |
| Sequence21-P-CG-Jp-ABD | FMENIIQPPISDDKEKAEFLRSAKQSFAGIIIGNQIRSDEKFMGV-FDES |
| Sequence32-NP-GA-Jp-ABD | FMENIIQPPISDDKEKAEFLRSAKQSFAGIIIGNQIRSDEKFMGV-FDES |
| Sequence116-NP-UD-Jp-ABC | FMENIIQPPIHDDKEKAEFLKSAKQSFAGIIIGNQIRTDQKFMGV-FDES |
| Sequence8-NP-GCA-Jp-ABD | FMENIIQPPISDDKEKAEFLRSAKQSFAGIIIGNQIRSDEKFMGV-FDES |
| Sequence83-P-CG-EA-ABD | FMENIIQPPISDDKEKAEFLRSAKQSFAGIIIGNQIRSDEKFMGV-FDES |
| Sequence48-NP-UD-OCC-ABC | FMENIIQPPISDDKEKAEFLRSAKQAFAGIIIGNQIQSDQKFMGV-FEES |
| Sequence120-NP-G-Jp-ABC | FMENIIQPPISDDKEKAEFLKSAKQSFAGIIIGNQIRTDQKFMGV-FEES |
| Sequence187-NP-UG-Jp-ABCCC | FMENIIQPPIPDDKEKAEFLKSAKQSFAGIIIGNQIRTDQKFMGV-FDEF |
| Sequence118-NP-G-Jp-ABD | FMENIIQPPISDDKEKAEFLRSAKQSFAGIIIGNQIRSDQKFMGV-FDES |
| Sequence87-NP-UG-Vit-ABD | FMENIIQPPISDDKEKAEFLRSAKQSFAGIIIGNQIRSDQKFMGV-FDES |

| | 201 … 250 |
|---|---|
| Sequence140-NP-GA-Col-ABABC | LKERQEAEKN----GGPTGGDWLDI--FLS-FIF-DKKQSSDV-KEAINQ |
| Sequence123-NP-UD-Jp-ABD | LKARQEAEKN----AEPAGGDWLDI--FLS-FVF-NKKQSSDL-KETLHQ |
| Sequence111-NP-G-Jp-ABD | LKERQEAEKN----AEPAGGDWLDI--FLS-FVF-NKKQSSDL-KETLHQ |
| Sequence57-NP-GA-OCC-AB | LKERQEAEKNGEPTGEPTGGDWLDI--FLS-FVF-NKKQSSDL-KETLNQ |
| Sequence172-P-CG-Col-ABCC | LKERQEAEKN----GEPTGGDWLDI--FLS-FIF-DKKQSSDV-KEAINQ |
| Sequence110-NP-UD-Jp-ABD | LKERQEAEKN----AEPAGGDWLDI--FLS-FVF-NKKQSSDL-KETLHQ |
| Sequence95-NP-UD-Vit-ABD | LKERQEAEKN----AEPA-GDWLDI--FLS-FVF-NKKQSSDL-KETLHQ |
| Sequence107-P-CG-Vit-ABD | LKARQEAEKN----AEPSGGDWLDI--FLS-FVF-NKKQSSDL-KETLHQ |
| Sequence106-P-CG-Vit-ABD | LERKARSRKK-------WRAYWWGLVGYFS-LIYIDKKQSSDV-KEAINQ |
| Sequence105-NP-UD-Vit-ABD | LKERQEAEKN----AEPSGGDWLDI--FLS-FVF-NKKQSSDL-KETLHQ |
| Sequence126-NP-UG-Jp-ABC | LKERQEAEKN----GGPTGGDWLDI--FLS-FIF-DKKQSSDI-KEAINQ |
| Sequence135-P-CG-Col-ABCCC | LKERQEAEKN----GEPTGVDWLDI--FLS-FIF-DKKQSSDV-KEAINQ |
| Sequence138-NP-GA-Col-ABC | LKERQEAEKN----GETTGGDWLDI--FLS-FVF-NKKQSSDD-KEAINQ |
| Sequence13-NP-UP-Jp-ABD | LKARQEAEKN----AEPAGGDWLDI--FLS-FVF-NKKQSSDL-KETLNQ |
| Sequence11-NP-UP-Jp-ABD | LKARQEAEKN----AEPAGGDWLDI--FLS-FVF-NKKQSSDL-KETLNQ |
| Sequence50-NP-GA-OCC-ABC | LKERQEAEKNGEPTGEPTGGDWLDI--FLS-FVF-NKKQSSDL-KETLNQ |
| Sequence26-NP-GA-Jp-ABD | LKERQEAEKN----AEPAGGDWLDI--FLS-FVF-NKKQSSDL-KETLNQ |
| Sequence55-NP-UD-OCC-ABCCC | LKERQEAEKNGEPTGEPTGGDWLDI--FLS-FVF-NKKQSSDL-KETLNQ |
| Sequence178-NP-M-Col-ABCC | LKERQEAEKN----GEPTGGDWLDI--FLS-FVF-NKKQSSDV-KEAINQ |
| Sequence40-NP-GA-Jp-ABD | LKARQEAEKN----AEPAGGDWLDI--FLS-FVF-NKKQSSDL-KETLNQ |
| Sequence43-NP-GA-Jp-ABD | LKARQEAEKN----AEPAGGDWLDI--FLS-FVF-NKKQSSDL-KETLNQ |
| Sequence139-NP-UD-Col-ABCC | LKERQEAEKN----GEPTGGDWLDI--FLS-FIF-DKKQSSDV-KEAINQ |

| | |
|---|---|
| Sequence7-P-CG-Jp-ABBD | LKERQEAEKN----AEPAGGDWLDI--FLS-FVF-NKKQSSDL-KETLNQ |
| Sequence91-P-CG-Vit-ABD | LKERQEAEKN----AEPAGGDWVDI--FLS-FGF-QQKTIFRS-QERSFK |
| Sequence29-NP-GA-Jp-ABABD | LKARQEAEKN----AEPAGGDWLDI--FLS-FVF-NKKQSSDL-KETLNQ |
| Sequence78-P-CG-EA-ABD | LKARQEAEKN----AEPAGGDWLDI--FLS-FVF-NKKQSSDL-KETLNQ |
| Sequence188-NP-GCA-Jp-AB | LKERQEAEKN----GEPTGGDWLDI--FLS-FIF-DKKQSSDV-KEAINQ |
| Sequence72-P-CG-EA-ABD | LKARQEAEKN----AEPAGEDWLDI--FLS-FVF-NKKQSSDL-KETLNQ |
| Sequence79-P-CG-EA-ABD | LKARQEAEKN----AEPAGGDWLDI--FLS-FVF-NKKQSSDL-KETLNQ |
| Sequence61-NP-UD-OCC-ABC | LKARQEAEKN----ADPAGGDWLDI--FLS-FVF-NKKQSSDL-KETLNQ |
| Sequence125-NP-UG-Jp-ABCC | LKERQEAEKN----GEPSGGDWLDI--FLS-FIF-DKKQSSDV-KETINQ |
| Sequence175-NP-G-Col-ABCC | LKERQEAEKN----GGPTGGDWLDI--FLS-FVF-NKKQSSDV-KEAINQ |
| Sequence122-NP-G-Jp-ABCC | LKERQEAEKN----GEPTGGDWLDI--FLS-FVF-NKKQSSDV-KEAINQ |
| Sequence36-NP-GA-Jp-ABD | LKERQEAEKN----AEPAGGDWLDI--FLS-FVF-NKKQSSDL-KETLNQ |
| Sequence58-NP-UD-OCC-ABC | LKERQEAEKNGEPTGEPTGGDWLDI--FLS-FVF-NKKQSSDL-KETLNQ |
| Sequence27-NP-GA-Jp-ABBD | LKARQEAEKN----AEPAGGDWLDI--FLS-FVF-NKKQSSDL-KETLNQ |
| Sequence93-P-CG-Vit-ABD | LKARQEAEKN----AEPAGGDWLDI--FLS-FVF-NKKQSSDL-KETLNQ |
| Sequence177-NP-M-Col-ABCC | LKERQEAEKN----GGPSGGDWLDI--FLS-FVF-GKKQSSDV-KEAINQ |
| Sequence182-NP-UD-Col-ABCC | LKERQEAEKN----GEPTGGDWLEI--FLS-FIF-DKKQSSDV-KEVINQ |
| Sequence114-NP-UG-Jp-ABC | LKERQEAEKN----GGPTGGDWLDI--FLS-LIF-DKKQSSDV-QEAINQ |
| Sequence74-P-CG-EA-ABD | LKARQEAEKN----AEPAGGDWLDI--FLS-FVF-NKKQSSDL-KETLNQ |
| Sequence20-P-CG-Jp-ABD | LKERQEAEKN----AEPAGGDWLDI--FLS-FVF-NKKQSSDL-KETLNQ |
| Sequence14-NP-GCA-Jp-ABD | LKARQEAEKN----EQPAGGDWLDI--FLS-FVF-NKKQSSDL-KETLNQ |
| Sequence39-NP-GA-Jp-ABC | LKERQEAEKN----GGPTGGAWLDI--FLS-FIF-DKKQSSDV-KEAINQ |
| Sequence170-NP-UD-Col-ABCC | LKERQEAEKN----GGPSGGDWLDI--FLS-FVF-NKKQSSDV-KEAINQ |
| Sequence76-P-CG-EA-ABD | LKARQEAEKN----AEPAGGDWLDI--FLS-FVF-NKKQSSDL-KETLNQ |
| Sequence53-NP-GA-EA-ABD | LKERQEAEKN----AEPAGGDWLDI--FLS-FVF-NKKQSSDL-KETLNQ |
| Sequence174-P-CG-Col-ABCC | LKERQEAEKN----GGPTGGDWLDI--FLS-FIF-DKKQSSDV-KEAINQ |
| Sequence33-NP-UG-Jp-ABD | LKERQEAEKN----GEPAGGDWLDI--FLS-FIF-DKKQSSDV-KEAINQ |
| Sequence60-NP-GA-EA-ABD | LKARQEAEKN----AEPAGGDWLDI--FLS-FVF-NKKQSSDL-KETLNQ |
| Sequence38-NP-GA-Jp-ABD | LKERQEAEKN----EEPAGGDWLDI--FLS-FVF-NKKQSSDL-KETLNQ |
| Sequence1-NP-EG-Jp-ABCCC | LKERQEAEKN----GEPTGGDWLDI--FLS-FVF-NKEQSSDV-KEAINQ |
| Sequence101-P-CG-Vit-ABD | LKERQEAEKN----AEPSGGDWLDI--FLS-FVF-NKKQSSDL-KETLHQ |
| Sequence64-NP-UD-OCC-ABC | LKERQEAEKNGGSTGEPIGGDWLDI--FLS-FVF-NKKQSSDL-KETLDQ |
| Sequence81-P-CG-EA-ABD | LKERQEAEKN----GEPAGGDWLDI--FLS-FVF-NKKQSSDL-KETLDQ |
| Sequence150-NP-DA-Sen-BC | LKERQEAEKN----GGSTGGDWLDI--FLS-FIF-DKKQSSDV-KETLNQ |
| Sequence90-NP-UD-Vit-ABD | LKERQEAEKN----AEPAGGDWLDI--FLS-FGV-NKKQSSDL-KKRSIK |
| Sequence136-P-CG-Col-AACC | LKERQEAEKN----GGPTGGDWLDI--FLS-FIF-DKKQSSDV-KEAINQ |
| Sequence15-NP-UP-Jp-ABD | LKARQEAEKN----AEPAGGDWLDI--FLS-FVF-NKKQSSDL-KETLNQ |
| Sequence108-NP-UD-Vit-ABD | LKERQEAEKN----AEPAGGDWLDI--FLS-FVF-NKEQSSDL-KETLHQ |
| Sequence104-P-CG-OCC-ABCC | LKERQEAEKN----GGPTGGDWLDI--FLS-FIF-DKKQSSDV-KEAINQ |
| Sequence179-NP-UD-Col-ABCC | LKERQEAEKN----GGPTGGDWLDI--FLS-FTF-GKKQSSDV-KEAINQ |
| Sequence37-NP-GA-Jp-ABCCC | LKERQEAEKNGEPTGEPTGGDWLDI--FLS-FVF-NKKQSSDL-KETLNQ |
| Sequence89-NP-UD-Vit-ABD | LKERQEAEKN----AEPAGGDWLDI--FLS-FVF-NKKQSSDL-KETLHQ |
| Sequence92-P-CG-Vit-ABD | LKERQEAEKN----AEPAGGDWLDI--FLS-FVF-NKKQSSDL-KETLHQ |
| Sequence49-NP-GA-Jp-ABC | LKERQEAEKNGGSTGEPTGGDWLDI--FLS-FVF-NKKQSSDL-KETLDQ |
| Sequence181-P-CG-Col-ABCC | LKERQEAEKN----GEPTGGDWLDI--FLS-FIF-DKKQSSDV-KEAINQ |
| Sequence25-NP-GA-Jp-ABD | LKARQEAEKN----AEPTGGDWLDI--FLS-FVF-NKKQSSDL-KETLNQ |
| Sequence4-P-CG-Jp-ABD | LKARQEAEKN----AEPAGGDWLDI--FLS-FVF-NKKQSSDL-KETLNQ |
| Sequence121-NP-G-Jp-ABCC | LKERQEAEKN----GEPTGGDWLDI--FLS-FIF-DKKQSSDV-KEAINQ |
| 4dvy | LKERQEAEKN----GGPTGGDWLDI--FLS-FIF-NKKQSSDV-KEAINQ |
| Sequence186-NP-UP-Jp-ABD | LKARQEAEKN----AEPAGGDWLDI--FLS-FVF-NKKQSSDL-KETLNQ |
| Sequence62-NP-UD-OCC-ABD | LKERQEAEKNGGSTGEPTGGDWLDI--FLS-FVF-NKKQSSDL-KETLDQ |
| Sequence9-NP-GA-Jp-ABD | LKARQEAEKN----AEPAGGDWLDI--FLS-FVF-NKKQSSDL-KETLNQ |
| Sequence12-NP-UP-Jp-ABD | LKARQEAEKN----EEPAGGDWLDI--FLS-FVF-NKKQSSDL-KETLNQ |
| Sequence35-NP-GA-Jp-ABABD | LKARQEAEKN----EEPTGGDWLDI--FLS-FVF-NKKQSSDL-KETLNQ |
| Sequence54-NP-GA-OCC-ABC | LKERQEAEKNGGSTGEPTGGDWLDI--FLS-FVF-NKKQSSDL-KETLDQ |
| Sequence109-P-CG-Jp-ABD | LKERQEAEKN----AEPA-GDWLDI--FLS-FVF-NKKQSSDL-KETLHQ |
| Sequence96-NP-UD-Vit-ABD | LKERQEAEKN----AELA-GDWLDI--FLS-FVF-NKKQSSDL-KETLHQ |
| Sequence80-P-CG-EA-ABD | LKARQEAEKN----AEPAGGDWLDI--FLS-FVF-NKKQSSDL-KETLNQ |
| Sequence82-P-CG-EA-ABD | LKERQEAEKN----AEPAGGDWLDI--FLS-FVF-NKKQSSDL-KETLHQ |
| Sequence10-NP-GCA-Jp-ABD | LKARQEAEKN----AEPAGGDWLDI--FLS-FVF-NKKQSSDL-KETLNQ |
| Sequence19-NP-UP-Jp-ABBD | LKERQEAEKN----AEPAGGDWLDI--FLS-FVF-NKKQSSDL-KETLNQ |

```
Sequence115-NP-UG-Jp-ABC       LKERQEAEKN----GGPTGGDWLDI--FLS-FIF-DKKQSSDV-KKAINQ
Sequence45-NP-GA-Jp-ABCC       LKERQEAEKN----GGPTGGDWLDI--FLS-FIF-DKKQSSDV-KEAINQ
Sequence119-NP-UG-Jp-ABC       LKARQEAEKN----GGPTSGDWLDI--FLS-FIF-DKKQSSDL-KETLNQ
Sequence70-NP-GA-Ch-ABD        LKARQEAEKN----AEPAGGDWLDI--FLS-FVF-NKKQSSDL-KETLNQ
Sequence52-NP-UD-EA-ABD        LKARQEAEKN----AEPAGGDWLDI--FLS-FVF-NKKQSSDL-KETLNQ
Sequence97-P-CG-Vit-ABD        LKERQEAEKN----AEPA-GDWLDI--FLS-FVF-NKKQSSDL-KETLHQ
Sequence94-NP-UD-Vit-ABD       LKERQEAEKN----VEPAGGDWFDI--FLS-FVF-NKKQSSDL-KETLHQ
Sequence176-NP-GA-Col-ABCC     LKERQEAEKN----GEPTGGDWLDI--FLS-FVF-NKKQSSDV-KEAINQ
Sequence68-NP-D-Am-ABBC        LKERQEAEKT--------GGDWLDI--FLS-FIF-DKKQSSDL-KETLNQ
Sequence47-NP-GA-Jp-ABD        LKARQEAEKN----AEPAGGDWLDI--FLS-FVF-NKKQSSDL-KETLNQ
Sequence56-NP-UD-OCC-ABC       LKERQEAEKNGEPTGEPTGGDWLDI--FLS-FVF-NKKQSSDL-KETLNQ
Sequence31-NP-GA-Jp-ABD        LKARQEAEKN----AEPAGGDWLDI--FLS-FVF-NKKQSSDL-KETLNQ
Sequence46-NP-UD-Jp-ABD        LKERQEAEKNGEPTGEPTGGDWLDI--FLS-FVF-NKKQSSDL-KETLNQ
Sequence102-NP-UG-Vit-ABD      LKERPEAEKN----AKPSGGDWLDI--FLS-FGF-NKKQSSDL-KETLPQ
Sequence71-P-CG-EA-ABD         LKERQEAEKN----AEPAGGDWLDI--FLS-FVF-NKKQSSDL-KETLNQ
Sequence30-NP-GA-Jp-ABBD       LKARQEAEKN----AEPAGGDWLDI--FLS-FVF-NKKQSSDL-KETLNQ
Sequence28-NP-GA-Jp-AABD       LKARQEAEKN----AEPAGGDWLDI--FLS-FVF-NKKQSSDL-KETLNQ
Sequence98-P-CG-Vit-AD         LKERQEAKKN----AEPRGGDWLDI--FLS-FVF-NKKQSSDL-QETLPP
Sequence99-P-CG-Vit-ABD        LKERQEAEKN----AKPA-GDWLDI--LLS-FVF-NKKQSSDL-RETLHQ
Sequence24-NP-UD-USA-ABD       LKERQEAEKN----AGPAGGDWLDI--FLS-FVF-NKKQSSDL-RETLNQ
Sequence124-NP-G-Jp-ABC        LKERQEAEKN----GGPTGGDWLDI--FLS-FIF-DEKQSSDI-KEAINQ
Sequence77-P-CG-OCC-ABCC       LKERQEAEKN----AEPAGGDWLDI--FLS-FVF-NKKQSSDL-KETLDQ
Sequence113-NP-UG-Jp-ABC       LKERQEAEKN----GGPTGGDWLDI--FLS-FIF-DKKQSSDV-KEAINQ
Sequence88-NP-UD-Vit-ABD       LKERQEAEKR---MQSLVGGDWLDI--FFI-ILD-STKNNLPISKETLHP
Sequence100-NP-UD-Vit-ABD      LKERQEAEKN----AEPA-GDWLDI--FLS-FVF-NKKQSSDL-KETLHQ
Sequence137-NP-G-Col-ABCC      LKERQEAEKN----GEPTGGDWLDI--FLS-FIF-DKKQSSDV-KEAINQ
Sequence117-NP-G-Jp-ABC        LKERQEAEKN----GGPTGGDWLDI--FLS-FIF-DKKQSSDV-KETLNQ
Sequence171-NP-UD-Col-ABCC     LKERQEAEKN----GEPTGGDWLDI--FLS-FVF-NKKQSSDV-KEAINQ
Sequence180-P-CG-Col-ABC       LKERQEAEKN----GEPTGGDWLDI--FLS-FVF-NKKQSSDV-KEAINQ
Sequence112-P-CG-Jp-ABCC       LKERQEAEKN----GGPSGGDWLDI--FLS-FIF-DKKQSSDV-KEAINQ
Sequence127-NP-UD-Jp-ABC       LKDRQEAEKN----GEPTGGDWLDI--FLS-FIF-DKKQSSDV-KETLNQ
Sequence51-NP-GA-OCC-ABC       LKERQEAEKNGGSTGEPTGGDWLDI--FLS-FVF-NKKQSSDL-KETLDQ
Sequence73-P-CG-EA-ABD         LKARQEAEKN----AEPAGGDWLDI--FLS-FVF-NKKQSSDL-KETLDQ
Sequence183-NP-UD-Col-ABD      LKERQEAEKN----GEPTGGDWLDI--FLS-FVF-NKKQSSDV-KEAINQ
Sequence34-NP-UD-Jp-ABDD       LKERQEAEKN----GEPTGGDWLDI--FLS-FIF-HKKQYSDV-KEAINQ
Sequence21-P-CG-Jp-ABD         LKARQEAEKN----AEPAGGDWLDI--FLS-FVF-NKKQSSDL-KETLNQ
Sequence32-NP-GA-Jp-ABD        LKERQEAEKN----AEPAGGDWLDI--FLS-FVF-NKKQSSDL-KETLNQ
Sequence116-NP-UD-Jp-ABC       LKERQEAEKN----GGPTGGDWLDI--FLS-FIF-DKKQSSDV-KKAINQ
Sequence8-NP-GCA-Jp-ABD        LKARQEAEKN----AEPAGGDWLDI--FLS-FVF-NKKQSSDL-KETLNQ
Sequence83-P-CG-EA-ABD         LKARQEAEKN----AEPAGGDWLDI--FLS-FVF-NKKQSSDL-KETLNQ
Sequence48-NP-UD-OCC-ABC       LKERQEAEKNGEPTGEPTGGDWLDI--FLS-FVF-NKKQSSDL-KETLNQ
Sequence120-NP-G-Jp-ABC        LKERQEAEKN----GGPTGGDWLDI--FLS-FIF-DKKQSSDV-QETLNQ
Sequence187-NP-UG-Jp-ABCCC     LKERQEAEKN----GEPTGGDWLDI--FLS-FVF-NKEQSSDV-KEAINQ
Sequence118-NP-G-Jp-ABD        LKERQEAEKN----AEPAGGDWLDI--FLS-FVF-NKKQSSDL-KETLNQ
Sequence87-NP-UG-Vit-ABD       LKERQGSRKK-------CRACWGLVGYFFYHLCS-TKKQSSDL-QETLHQ

                               251                                              300
Sequence140-NP-GA-Col-ABABC    EPVPHVQPDIATTTTDIQGLPPEAR-------DL-LDERGNFSKFTL---
Sequence123-NP-UD-Jp-ABD       EPRPDFEQNVATTTTDIQGLPPEAR-------DL-LDERGNFFKFTL---
Sequence111-NP-G-Jp-ABD        EPRPDFEQNLATTTTDIQGLPPEAR-------DL-LGERGKFFKFTL---
Sequence57-NP-GA-OCC-AB        EPVPHVQPDIATTTTDIQSLPPEAR-------DL-LDERGNFSKFTL---
Sequence172-P-CG-Col-ABCC      EPVPHVQPDIATTTTDIQGLPPESR-------DL-LDERGNFSKFTL---
Sequence110-NP-UD-Jp-ABD       EPRPDFEQNLATTTTDIQGLPPEAR-------DL-LDERGNFFKFTL---
Sequence95-NP-UD-Vit-ABD       EPRPDFEQNIATTTTDIQGLPPEAR-------DL-LDERGNFSKFTL---
Sequence107-P-CG-Vit-ABD       EPRPDFEQNLATTTTDIQGLPPEAR-------DL-LDERGNFSKFTL---
Sequence106-P-CG-Vit-ABD       EPVPHVQPDMATTTTHIQGLPPESR-------DL-LDERGNFSKFTL---
Sequence105-NP-UD-Vit-ABD      EPRPDFEQNLATTTTDIQGLPPEAR-------DL-LDERGNFSKFTL---
Sequence126-NP-UG-Jp-ABC       EPVPHIQPDIATSTTHIQGLPPESR-------DL-LDERGNFSKFTL---
Sequence135-P-CG-Col-ABCCC     EPVPHVQPDIATTTTDIQGLPPESR-------DL-LDERGNFSKFTL---
Sequence138-NP-GA-Col-ABC      EPVPHVQPDIATTTTDIQGLPPESR-------DL-LDERGNFSKFTL---
Sequence13-NP-UP-Jp-ABD        EPRPDFEQNLATTTTDIQGLPPEAR-------DL-LDERGNFFKFTL---
```

```
Sequence11-NP-UP-Jp-ABD       EPRPDFEQNLATTTTDIQGLPPESR-------DL-LDERGNFSKFTL---
Sequence50-NP-GA-OCC-ABC      EPRPDFEQNLATTTTDIQGLPPEAR-------DL-LDERGNFFKFTL---
Sequence26-NP-GA-Jp-ABD       EPRPDFEQNLATTTTDIQGLPPEAR-------DL-LDERGNFFKFTL---
Sequence55-NP-UD-OCC-ABCCC    EPVPHVQPDIATTTTDIQSLPPEAR-------DL-LDERGNFSKFTL---
Sequence178-NP-M-Col-ABCC     EPVPHVQPDIATTTTDIQGLPPESR-------DL-LDERGNFSKFTL---
Sequence40-NP-GA-Jp-ABD       EPRPDFEQNLATTTTDIQGLPPEAR-------DL-LDERGNFFKFTL---
Sequence43-NP-GA-Jp-ABD       EPRPDFEQNLATTTTDIQGLPPESR-------DL-LDERGNFSKFTL---
Sequence139-NP-UD-Col-ABCC    EPVPHVQPDIATTTTDIQGLPPESR-------DL-LDERGNFSKFTL---
Sequence7-P-CG-Jp-ABBD        EPRPDFEQNLATTTTDIQGLPPEAR-------DL-LDERGNFFKFTL---
Sequence91-P-CG-Vit-ABD       SQGLILNQNLATTAPDIQGFPPEAR-------DL-LDERGNFFKFTL---
Sequence29-NP-GA-Jp-ABABD     EPRPDFEQNIATTTTSIQGLPPEAR-------DL-LDERGNFFKFTL---
Sequence78-P-CG-EA-ABD        EPRPDFEQNLATTTTDIQGLPPESR-------DL-LDERGNFSKFTL---
Sequence188-NP-GCA-Jp-AB      EPVPHVQPDIATTTTDIQGLPPEAR-------DL-LDERGNFSKFTL---
Sequence72-P-CG-EA-ABD        EPRPDFEQNLPTTTTDIQGLPPEAR-------DL-LDERGNFFKFTL---
Sequence79-P-CG-EA-ABD        EPMPNVEQNLATTTTDIQGLPPEAR-------DL-LDERGNFFKFTL---
Sequence61-NP-UD-OCC-ABC      EPMPNVEQNLATTTTDIQGLPPEAR-------DL-LDERGNFFKFTL---
Sequence125-NP-UG-Jp-ABCC     EPVPHVQPDIATTTTHIQGLPPESR-------DL-LDERGNFSKFTL---
Sequence175-NP-G-Col-ABCC     EPVPHVQPDIATTTTDIQGLPPESR-------DL-LDERGNFSKFTL---
Sequence122-NP-G-Jp-ABCC      EPVPHVQPDIATTTTDIQGLPPESR-------DL-LDERGNFSKFTL---
Sequence36-NP-GA-Jp-ABD       EPRPDFEQNLATTTTDIQGLPPEAR-------DL-LDERGNFFKFTL---
Sequence58-NP-UD-OCC-ABC      EPVPHVQPDIATTTTDIQSLPPEAR-------DL-LDERGNFSKFTL---
Sequence27-NP-GA-Jp-ABBD      EPRPDFEQNLATTTTDIQGLPPEAR-------DL-LDERGNFFKFTL---
Sequence93-P-CG-Vit-ABD       EPRPDFEQNLATTTTDIQGLPPEAR-------DL-LDERGNFSKFTL---
Sequence177-NP-M-Col-ABCC     EPVPHVQPDIATTTTDIQGLPPEAR-------DL-LDERGNFSKFTL---
Sequence182-NP-UD-Col-ABCC    EPVPHVQPDIATTTTHIQGLPPESR-------DL-LDERGNFSKFTL---
Sequence114-NP-UG-Jp-ABC      EPVPPVQPDIATTTTDIQGLPPEAR-------DL-LDERGNFSKFTL---
Sequence74-P-CG-EA-ABD        EPRPDFEQNIATTTTSIQGLPPEAR-------DL-LDERGNFFKFTL---
Sequence20-P-CG-Jp-ABD        EPRPDFEQNLATTTTDIQGLPPEAR-------DL-LDERGNFFKFTL---
Sequence14-NP-GCA-Jp-ABD      EPRPSVEQNIATTTTDIQGLPPEAR-------DL-LDERGNFFKFTL---
Sequence39-NP-GA-Jp-ABC       EPLPHVQPDIATSTTHIQGLPPESR-------DL-LDERGNFSKFTL---
Sequence170-NP-UD-Col-ABCC    EPVPHVQPDIATTTTDIQGLPPESR-------DL-LDERGNFSKFTL---
Sequence76-P-CG-EA-ABD        EPRPDFEQNLATTTTDIQGLPPEAR-------DL-LDERGNFFKFTL---
Sequence53-NP-GA-EA-ABD       EPRPDFEQNLATTTTDIQGLPPEAR-------DL-LDERGNFFKFTL---
Sequence174-P-CG-Col-ABCC     EPVPHVQPDIATTTTHIQGLPPESR-------DL-LDERGNFSKFTL---
Sequence33-NP-UG-Jp-ABD       EPVPHVQPDIATTTTDIQGLPPESR-------DL-LDERGNFSKFTL---
Sequence60-NP-GA-EA-ABD       EPRPDFEQNLATTTTDIQGLPPEAR-------DL-LDERGNFFKFTL---
Sequence38-NP-GA-Jp-ABD       EPRPDFEQNLATTTTDIQGLPPEAR-------DL-LDERGNFFKFTL---
Sequence1-NP-EG-Jp-ABCCC      EPVPHVQPDIATTTTHIQGLPPESR-------DL-LDERGNFSKFTL---
Sequence101-P-CG-Vit-ABD      EPRPDFEQNLATTTTDIQGLPPEAR-------DL-LDERGNFSKFTL---
Sequence64-NP-UD-OCC-ABC      EPVPHVQPDIATTTTDIQGLPPESR-------DL-LDERGNFSKFTL---
Sequence81-P-CG-EA-ABD        EPVPHVQPDIATTTTDIQGLPPESR-------DF-LDERGNFSKFTL---
Sequence150-NP-DA-Sen-BC      EPVPHVQPDVATTTTDIQGLPPEAR-------DL-LDERGNFSKFTL---
Sequence90-NP-UD-Vit-ABD      SQGLILNKISPLPPPTYKGYRLKLE-------IC-LMKGVIFLNSLL---
Sequence136-P-CG-Col-AACC     EPVPHVQPDIATTTTDIQGLPPESR-------DL-LDERGNFSKFTL---
Sequence15-NP-UP-Jp-ABD       EPRPDFEQNLATTTTSIQGLPPEAR-------DL-LDERGNFFKFTL---
Sequence108-NP-UD-Vit-ABD     EPRPDFEQNLATT-TDIQGLPPEAR-------DL-LDERGNFFKFTL---
Sequence104-P-CG-OCC-ABCC     EPVPHVQPDIATTTTHIQGLPPESR-------DL-LDERGNFSKFTL---
Sequence179-NP-UD-Col-ABCC    EPVPHVQPDIATTTTDIQGLPPESR-------DL-LDERGNFSKFTL---
Sequence37-NP-GA-Jp-ABCCC     EPVPHVQPDIATTTTDIQSLPPEAR-------DL-LDERGNFSKFTL---
Sequence89-NP-UD-Vit-ABD      EPRPDFEQNLATTTTDIQGLPPEAR-------DL-LDERGNFSKFTL---
Sequence92-P-CG-Vit-ABD       EPRPDFEQNLATTATDIQGLPPEAR-------DL-LDERGNFFKFTL---
Sequence49-NP-GA-Jp-ABC       EPVSHVQPDIATTTTDIQGLPPESR-------DL-LDERGNFSKFTL---
Sequence181-P-CG-Col-ABCC     EPVPHVQPDIATTTTDIQGLPPESR-------DL-LDERGNFSKFTL---
Sequence25-NP-GA-Jp-ABD       EPRPDFEQNLATTTTDIQGLPPEAR-------DL-LDERGNFFKFTL---
Sequence4-P-CG-Jp-ABD        EPRPDFEQNLATTTTDIQGLPPEAR-------DL-LDERGNFFKFTL---
Sequence121-NP-G-Jp-ABCC      EPVPHVQPDIATTTTHIQGLPPEAR-------DL-LDERGNFSKFTL---
4dvy                          EPVPHVQPDIATTTTDIQGLPPEAR-------DL-LDERGNFSKFTM---
Sequence186-NP-UP-Jp-ABD      EPRPDFEQNLSTTTTDIQGLPPEAR-------DL-LDERGNFFKFTL---
Sequence62-NP-UD-OCC-ABD      EPVSHVQPDIATTTTDIQGLPPESR-------DL-LDERGNFSKFTL---
Sequence9-NP-GCA-Jp-ABD       EPRPDFEQNLATTTTDIQGLPPEAR-------DL-LDERGNFFKFTL---
Sequence12-NP-UP-Jp-ABD       EPRPDFEQNLATTTTDIQGLPPEAR-------DL-LDERGNFFKFTL---
```

```
Sequence35-NP-GA-Jp-ABABD     EPRPDFEQNLATTTTDIQGLPPEAR-------DL-LDERGNFFKFTL---
Sequence54-NP-GA-OCC-ABC      EPVSHVQPDIATTTTDIQGLPPESR-------DL-LDERGNFSKFTL---
Sequence109-P-CG-Jp-ABD       EPRPDFEQNIATTTTDIQGLPPEAR-------DL-LDERGNFFKFTL---
Sequence96-NP-UD-Vit-ABD      EPRPDFEQNLATTTTDIQGLPPEAR-------DL-LDERGNFSKFTL---
Sequence80-P-CG-EA-ABD        EPMPNVEQNLATTTTDIQGLPPEAR-------DL-LDERGNFFKFTL---
Sequence82-P-CG-EA-ABD        EPRPDFEQNLATTTTDIQGLPPEAR-------DL-LDERGNFFKFTL---
Sequence10-NP-GCA-Jp-ABD      EPRPDFEQNLATTTTSIQGLPPEAR-------DL-LDERGNFFKFTL---
Sequence19-NP-UP-Jp-ABBD      EPRPDFEQNIATTTTSIQGLPPEAR-------DL-LDERGNFFKFTL---
Sequence115-NP-UG-Jp-ABC      EPVPHVQPDIATTTTDIQGLPPEAR-------DL-LDERGNFSKFTL---
Sequence45-NP-GA-Jp-ABCC      EPVPHVQPDIATTTTDIQGLPPEAR-------DL-LDERGNFSKFTL---
Sequence119-NP-UG-Jp-ABC      EPVPHVQPDIATTTTDIQGLPPEAR-------DL-LDERGNFSKFTL---
Sequence70-NP-GA-Ch-ABD       EPRPDFEQNLATTTTDIQGLPPEAR-------DL-LDERGNFSKFTL---
Sequence52-NP-UD-EA-ABD       EPRPDFEQNIATTTTDIQGLPPEAR-------DL-LDERGNFFKFTL---
Sequence97-P-CG-Vit-ABD       EPRADFEQNLATTTTDIRGLPPEAR-------DL-LEERGNFSKFTL---
Sequence94-NP-UD-Vit-ABD      EPRPDFEQNLATTTTDIQGLPPEAR-------DL-LDERGNFSKFTL---
Sequence176-NP-GA-Col-ABCC    EPVPHVQPDIATTTTDIQGLPPESR-------DL-LDERGNFSKFTL---
Sequence68-NP-D-Am-ABBC       EPVPHVQPDIATTTTDIQGLPPEAR-------DL-LDERGNFSKFTL---
Sequence47-NP-GA-Jp-ABD       EPRPDFEQNLATTTTDIQGLPPEAR-------DL-LDERGNFFKFTL---
Sequence56-NP-UD-OCC-ABC      EPVPHVQPDIATTTTDIQSLPPEAR-------DL-LDERGNFSKFTL---
Sequence31-NP-GA-Jp-ABD       EPRPDFEQNLATTTTDIQGLPPEAR-------DL-LDERGNFFKFTL---
Sequence46-NP-UD-Jp-ABD       EPVPHVQPDIATTTTDIQSLPPEAR-------DF-LDERGNFSKFTL---
Sequence102-NP-UG-Vit-ABD     EPRPDFEQNLATTTTDIQGLPPEAR-------DL-LDERGNFSNSLL---
Sequence71-P-CG-EA-ABD        EPRPDFEQNLATTTTDIQGLPPEAR-------DL-LDERGNFFKFTL---
Sequence30-NP-GA-Jp-ABBD      EPRPDFEQNLATTTTDIQGLPPEAR-------DL-LDERGNFFKFTL---
Sequence28-NP-GA-Jp-AABD      EPRPDFEQNLATTTTDIQGLPPEAR-------DL-LDERGNFFKFTL---
Sequence98-P-CG-Vit-AD        EPRADFEQNIATTTTDIQGLPPEAR-------DL-LDVRGNFFKFTL---
Sequence99-P-CG-Vit-ABD       EPRPDFEQNLATTTTDIQGLPPEAR-------DL-LDERGNFFKFTL---
Sequence24-NP-UD-USA-ABD      KPRPDFEQNLATTTTDIQGLPPEVR-------DL-LDERGYFFKFTL---
Sequence124-NP-G-Jp-ABC       EPVPHIQPDIATSTTHIQGLPPESR-------DL-LDERGNFSKFTL---
Sequence77-P-CG-OCC-ABCC      EPVPHVQPDIATTTTDIQGLPPESR-------DL-LDERGNFSKFTL---
Sequence113-NP-UG-Jp-ABC      EPVPHVQPDIATTTTDIQGLPPEAR-------DL-LDERGNFSKFTL---
Sequence88-NP-UD-Vit-ABD      GAR-------ALILNKIKPLPPPTYKAYRLKLEICLMKGVIFLNSLL---
Sequence100-NP-UD-Vit-ABD     EPRPDFEQNLATTTTDIQGLPPEAR-------DL-LDERGNFSKFTL---
Sequence137-NP-G-Col-ABCC     EPVPHVQPDIATSTTHIQGLPPESR-------DL-LDERGNFSKFTL---
Sequence117-NP-G-Jp-ABC       EPVPHVQPDIATTTTDIQGLPPEAR-------DL-LDERGNFSKFTL---
Sequence171-NP-UD-Col-ABCC    EPVPHIQPDIATTTTDIQGLPPESR-------DL-LDERGNFSKFTL---
Sequence180-P-CG-Col-ABC      EPVPHVQPDIATTTTDIQGLPPESR-------DL-LDERGNFSKFTL---
Sequence112-P-CG-Jp-ABCC      EPVPHVQPDIATTTTDIQGLPPESR-------DL-LDERGNFSKFTL---
Sequence127-NP-UD-Jp-ABC      EPVPHVQPDIATTTTDIQGLPPESR-------DL-LDERGNFSKFTL---
Sequence51-NP-GA-OCC-ABC      EPVPHVQPDIATTTTDIQGLPPESR-------DL-LDERGNFSKFTL---
Sequence73-P-CG-EA-ABD        EPRPDFEQNLATTTTDIQGLPPEAR-------DL-LDERGNFFKFTL---
Sequence183-NP-UD-Col-ABD     EPVPHVQPDIATTTTPIQGLPPESR-------DL-LDERGNFSKFTL---
Sequence34-NP-UD-Jp-ABDD      EPVPHVQPDIATTTTHIQGLPPESR-------DL-LDERGNFSKFTL---
Sequence21-P-CG-Jp-ABD        EPRPDFEQNLATTTTDIQGLPPEAR-------DL-LDERGNFFKFTL---
Sequence32-NP-GA-Jp-ABD       EPRPDFEQNIATTTTSIQGLPPEAR-------DL-LDERGNFFKFTL---
Sequence116-NP-UD-Jp-ABC      EPVPHVQPDIATTTTDIQGLPPEAR-------DL-LDERGNFSKFTL---
Sequence8-NP-GCA-Jp-ABD       EPRPDFEQNLATTTTDIQGLPPEAR-------DL-LDERGNFFKFTL---
Sequence83-P-CG-EA-ABD        EPRPDFEQNLATTTTDIQGLPPEAR-------DL-LDERGNFFKFTL---
Sequence48-NP-UD-OCC-ABC      EPVPHVQPDIATTTTDIQSLPPEAR-------DL-LDERGNFSKFTL---
Sequence120-NP-G-Jp-ABC       EPVPPVQPDIATTTTDIQGLPPESR-------DL-LDERGNFSKFTL---
Sequence187-NP-UG-Jp-ABCCC    EPVPHVQPDIATTTTHIQGLPPESR-------DL-LDERGNFSKFTL---
Sequence118-NP-G-Jp-ABD       EPRPDFEQNLATTTTDIQGLPPEAR-------DL-LDERGNFFKFTL---
Sequence87-NP-UG-Vit-ABD      EPRSGFSTKFSPYHHRHTRLTARSL-------EICLMRGGNFSKFPLGGF

                              301                                            350
Sequence140-NP-GA-Col-ABABC   GDMEMLDVEGVADIDPNYKF-NQLLIHNNALSSVLM---GSH-NGIEPEK
Sequence123-NP-UD-Jp-ABD      GDMEMLDVEGVADKDPNYKF-NQLLIHNNALSSVLM---GSH-SNIEPEK
Sequence111-NP-G-Jp-ABD       GDVEMLDVEGVADKDPNYKF-NQLLIHNNALSSVLM---GSH-SSIEPEK
Sequence57-NP-GA-OCC-AB       GDMEMLDVEGVADIDPNYKF-NQLLIHNNALSSVLM---GSH-NGIEPEK
Sequence172-P-CG-Col-ABCC     GDMEMLDVEGVADIDPNYKF-NQLLIHNNALSSVLM---GSH-NGIEPEK
Sequence110-NP-UD-Jp-ABD      GDVEMLDVEGVADKDPNYKF-NQLLIHNNALSSVLM---GGH-SNIEPEK
```

| | |
|---|---|
| Sequence95-NP-UD-Vit-ABD | GDMEMLDVEGVADKDPNYSS----INYSSTITLYLLCYGGVI-AVSNLKS |
| Sequence107-P-CG-Vit-ABD | GDMEMLDVEGVADKDPNYKF-NQLLIHNNALSSVLM---GGH-SNIEPEK |
| Sequence106-P-CG-Vit-ABD | GDMEMLDVEGVADIDPNYKF-NQLL-YPQVYALIFCVIWESH--TENPEK |
| Sequence105-NP-UD-Vit-ABD | GDMEMLDVEGVADKDPNYKF-NQLLIHNNALSSVLM---GGH-SSIEPEK |
| Sequence126-NP-UG-Jp-ABC | GDMEMLDVEGVADIDPNYKF-NQLLIHNNALSSVLM---GSH-NGIEPEK |
| Sequence135-P-CG-Col-ABCCC | GDMEMLDVEGVADIDPNYKF-NQLLIHNNALSSVLM---GSH-NGIEPEK |
| Sequence138-NP-GA-Col-ABC | GDMEMLDVEGVADIDPNYKF-NQLLIHNNTLSSVLM---GSH-DGIEPEK |
| Sequence13-NP-UP-Jp-ABD | GDVEMLDVEGVADKDPNYKF-NQLLIHNNALSSMLM---GGH-SNIEPEK |
| Sequence11-NP-UP-Jp-ABD | GDMELLDVEGVADIDPNYKF-NQLLIHNNALSSVLM---GGH-SNIEPEK |
| Sequence50-NP-GA-OCC-ABC | GDVEMLDVEGVADKDPNYKF-NQLLIHNNALSSVLM---GSH-SSIEPEK |
| Sequence26-NP-GA-Jp-ABD | GDVEMLDVEGVADKDPNYKF-NQLLIHNNALSSVLM---GSH-SNIEPEK |
| Sequence55-NP-UD-OCC-ABCCC | GDMEMLDVEGVADIDPNYKF-NQLLIHNNALSSVLM---GSH-NGIEPEK |
| Sequence178-NP-M-Col-ABCC | GDMEMLDVEGVADIDPNYKF-NQLLIHNNALSSVLM---GSH-NGIEPEK |
| Sequence40-NP-GA-Jp-ABD | GDVEMLDVEGVADKDPNYKF-NQLLIHNNALSSVLM---GGH-SNIEPEK |
| Sequence43-NP-GA-Jp-ABD | GDMEMLDVEGVADKDPNYKF-NQLLIHNNALSSVLM---GGH-SNIEPEK |
| Sequence139-NP-UD-Col-ABCC | GDMEMLDVEGVADIDPNYKF-NQLLIHNNALSSVLM---GSH-NGIEPEK |
| Sequence7-P-CG-Jp-ABBD | GDVEMLDVEGVADKDPNYKF-NQLLIHNNALSSVLM---GSH-SSIEPEK |
| Sequence91-P-CG-Vit-ABD | GDMKMLDVEGVADKDPNYKF-NQLLIHNNALSSVLM---GGH-SSIEPEK |
| Sequence29-NP-GA-Jp-ABABD | GDVEMLDVEGVADKDPNYKF-NQLLIHNNALSSVLM---GSH-SSIEPEK |
| Sequence78-P-CG-EA-ABD | GDMEMLDVEGVADIDPNYKF-NQLLIHNNALSSVLM---GSH-NGIEPEK |
| Sequence188-NP-GCA-Jp-AB | GDMEMLDVEGVADIDPNYKF-NQLLIHNNALSSVLM---GSH-NGIEPEK |
| Sequence72-P-CG-EA-ABD | GDVEMLDVEGVADKDPNYKF-NQLLIHNNALSSMLM---GSH-SNIEPEK |
| Sequence79-P-CG-EA-ABD | GDVEMLDVEGVADKDPNYKF-NQLLIHNNALSSVLM---GGH-SNIEPEK |
| Sequence61-NP-UD-OCC-ABC | GDVEMLDVEGVADKDPNYKF-NQLLIHNNALSSVLM---GGH-SNIEPEK |
| Sequence125-NP-UG-Jp-ABCC | GDMEMLDVEGVADIDPNYKF-NQLLIHNNALSSVLM---GSH-NGIEPEK |
| Sequence175-NP-G-Col-ABCC | GDMEMLDVEGVADIDPNYKF-NQLLIHNNALSSVLM---GSH-NGIEPEK |
| Sequence122-NP-G-Jp-ABCC | GDMEMLDVEGVADIDPNYKF-NQLLIHNNALSSVLM---GSH-NGIEPEK |
| Sequence36-NP-GA-Jp-ABD | GDVEMLDVEGVADKDPNYKF-NQLLIHNNALSSVLM---GSH-SSIEPEK |
| Sequence58-NP-UD-OCC-ABC | GDMEMLDVEGVADIDPNYKF-NQLLIHNNALSSVLM---GSH-NGIEPEK |
| Sequence27-NP-GA-Jp-ABBD | GDVEMLDVEGVADKDPNYKF-NQLLIHNNALSSVLM---GGH-SNIEPEK |
| Sequence93-P-CG-Vit-ABD | GDMEMLDVEGVADKDP-ITG-SSILIHNNALSSVLM---GGH-SSYRTRK |
| Sequence177-NP-M-Col-ABCC | GDVEMLDVEGVADMDPNYKF-NQLLIHNNALSSVLM---GSH-NGIEPEK |
| Sequence182-NP-UD-Col-ABCC | GDMEMLDVEGVADIDPNYKF-NQLLIHNNALSSVLM---GSH-NGIEPEK |
| Sequence114-NP-UG-Jp-ABC | GDMEMLDVEGVADIDPNYKF-NQLLIHNNALSSVLM---GSH-NGIEPEK |
| Sequence74-P-CG-EA-ABD | GDVEMLDVEGVADKDPNYKF-NQLLIHNNALSSVLM---GSH-NNIEPEK |
| Sequence20-P-CG-Jp-ABD | GDVEMLDVEGVADKDPNYKF-NQLLIHNNALSSVLM---GSH-SNIEPEK |
| Sequence14-NP-GCA-Jp-ABD | GDVEMLDVEGVPDKDPNYKF-NQLLIHNNALSSVLM---GSH-SNIEPEK |
| Sequence39-NP-GA-Jp-ABC | GDMEMLDVEGVADMDPNYKF-NQLLIHNNALSSVLM---GSH-DGIEPEK |
| Sequence170-NP-UD-Col-ABCC | GDMEMLDVEGVADIDPNYKF-NQLLIHNNALSSVLM---GSH-DGIEPEK |
| Sequence76-P-CG-EA-ABD | GDVEMLDVEGVADKDPNYKF-NQLLIHNNTLSSVLM---GSH-SNIEPEK |
| Sequence53-NP-GA-EA-ABD | GDVEMLDVEGVADKDPNYKF-NQLLIHNNALSSVLM---GSH-SSIEPEK |
| Sequence174-P-CG-Col-ABCC | GDMEMLDVEGVADMDPNYKF-NQLLIHNNALSSVLM---GSH-NGIEPEK |
| Sequence33-NP-UG-Jp-ABD | GDMEMLDVEGVADIDPNYKF-NQLLIHNNALSSVLM---GSH-NGIEPEK |
| Sequence60-NP-GA-EA-ABD | GDVEMLDVEGVADKDPNYKF-NQLLIHNNALSSVLM---GSH-SSIEPEK |
| Sequence38-NP-GA-Jp-ABD | GDVEMLDVEGVADKDPNYKF-NQLLIHNNALSSMLM---GSH-NNIEPEK |
| Sequence1-NP-EG-Jp-ABCCC | GDMEMLDVEGVADIDPNYKF-NQLLIHNNALSSVLM---GSH-NGIEPEK |
| Sequence101-P-CG-Vit-ABD | GDMEMLDVEGVADNDPNYKF-NQLLIHNNALSSVLM---GGH-SSIEPEK |
| Sequence64-NP-UD-OCC-ABC | GDMEMLDVEGVADIDPNYKF-NQLLIHNNALSSVLM---GSH-NGVEPEK |
| Sequence81-P-CG-EA-ABD | GDMEMLDVEGVADIDPNYKF-NQLLIHNNALSSVLM---GGH-GNIEPEK |
| Sequence150-NP-DA-Sen-BC | GDMEMLDVEGVADIDPNYKF-NQLLIHNNALSSVLM---GSH-NGIEPEK |
| Sequence90-NP-UD-Vit-ABD | VIWKCWMLRESLIRIPNYKFQSNYSFHNNALSSVTN---GGFISSIEPEK |
| Sequence136-P-CG-Col-AACC | GDMEMLDVEGVADMDPNYKF-NQLLIHNNALSSVLM---GSH-NGIEPEK |
| Sequence15-NP-UP-Jp-ABD | GDVEMLDVEGVADKDPNYKF-NQLLIHNNALSSVLM---GGH-SSIEPEK |
| Sequence108-NP-UD-Vit-ABD | GDVEMLDVEGVADKDPNYSS----INYGSTITLYLLCQWGVH-SNIEPEK |
| Sequence104-P-CG-OCC-ABCC | GDMEMLDVEGVADLDPNYKF-NQLLIHNNALSSVLM---GSH-NGIEPEK |
| Sequence179-NP-UD-Col-ABCC | GDMEMLDVEGVADIDPNYKF-NQLLIHNNALSSVLM---GSH-NGIEPEK |
| Sequence37-NP-GA-Jp-ABCCC | GDMEMLDVEGVADIDPNYKF-NQLLIHNNALSSVLM---GSH-NGIEPEK |
| Sequence89-NP-UD-Vit-ABD | GDMEMLDVEGVADIDPNYKF-NQLLIHNNALSSVLM---GSH-NGIEPEK |
| Sequence92-P-CG-Vit-ABD | GDMEMLDVEGVADKDPNYKF-NQLLIPNNALSSVLM---GGH-SSIEPEK |
| Sequence49-NP-GA-Jp-ABC | GDMEMLDVEGVADIDPNYKF-NQLLIHNNALSSVLM---GSH-NGIEPEK |
| Sequence181-P-CG-Col-ABCC | GDMEMLDVEGVADIDPNYKF-NQLLIHNNALSSVLM---GSH-NGIEPEK |

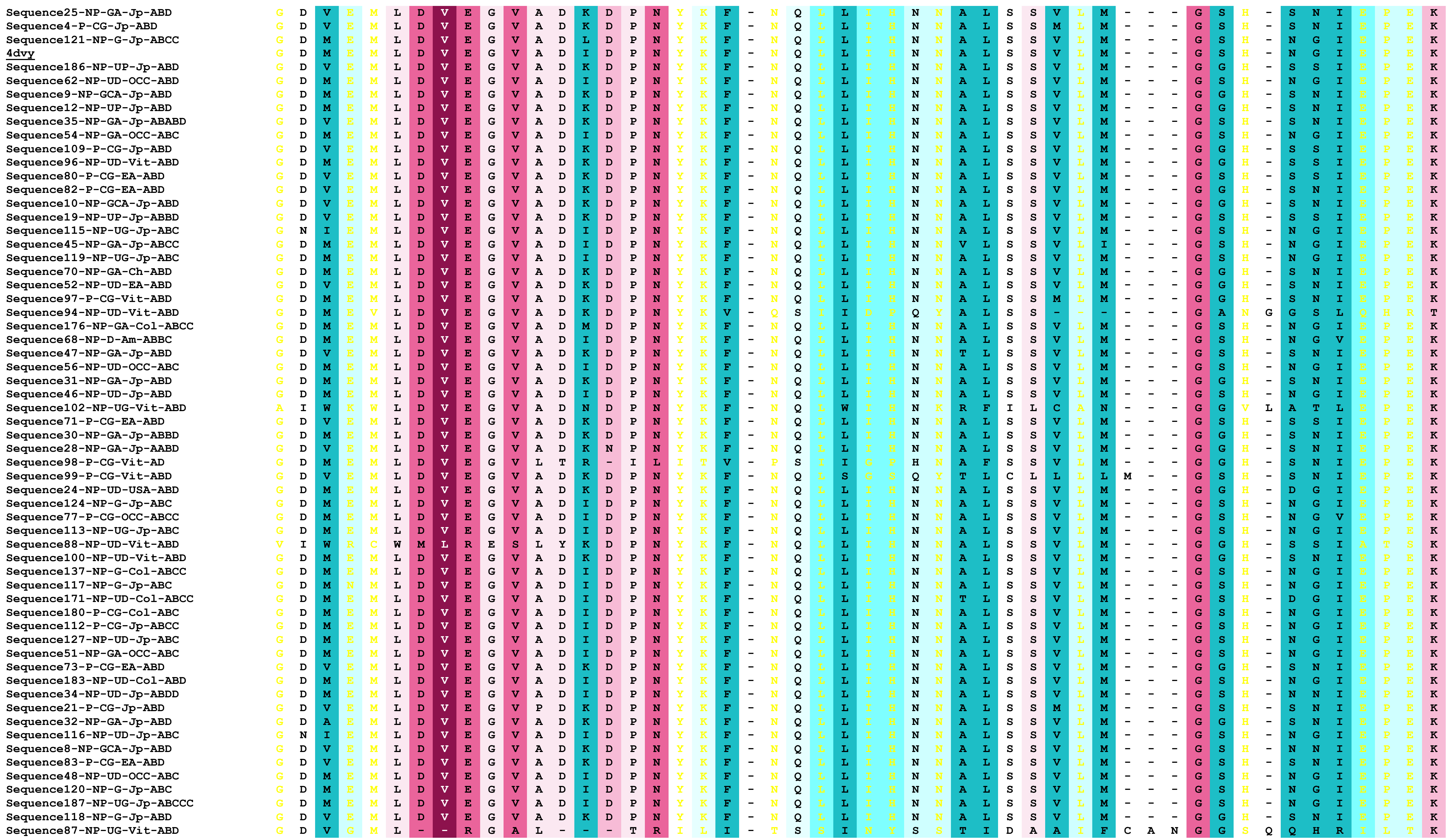

```
                                     351                                                                                    400
Sequence140-NP-GA-Col-ABABC    - - - - - - - - - - - V S L L Y A G N G G F G D K H D W N A T V G Y K D Q Q G N N V A T I I N V H M
Sequence123-NP-UD-Jp-ABD       - - - - - - - - - - - V S L L Y G D N G G P E A R H D W N A T V G Y K D Q Q G S N V A T L I N A H L
Sequence111-NP-G-Jp-ABD        - - - - - - - - - - - V S L L Y G D N G G P E A R H D W N A T V G Y K D Q Q G S N V A T L I N A H L
Sequence57-NP-GA-OCC-AB        - - - - - - - - - - - V S L L Y G G N G G P E A R H D W N A T V G Y K N Q Q G D N V A T L I N V H M
Sequence172-P-CG-Col-ABCC      - - - - - - - - - - - V S L L Y A G N G G F G A K H D W N A T V G Y K D Q Q G N N V A T I I N V H M
Sequence110-NP-UD-Jp-ABD       - - - - - - - - - - - V S L L Y G D N G G P E A R H D W N A T V G Y K N Q Q G N N V A T L I N A H L
Sequence95-NP-UD-Vit-ABD       - - - - - - - - - - - S Y Y C M G I M V A L N L G M I G T L P L V I K T N K A V M W P A L I N A H L
Sequence107-P-CG-Vit-ABD       - - - - - - - - - - - V S L L Y G D N G G P E A R H D W N A T V G Y K D Q Q G S N V A T L I N A H L
Sequence106-P-CG-Vit-ABD       - - - - - - - - - - - V S L L Y A G N G G P E A R H A W N A T V G Y K D Q Q G S N V A T L I N A H L
Sequence105-NP-UD-Vit-ABD      - - - - - - - - - - - V S L L Y G D N G S L E A R H D W N A T V G Y K D Q Q G S N V A T L I N A H L
Sequence126-NP-UG-Jp-ABC       - - - - - - - - - - - V S L L Y A G N G G F G A K H D W N A T V G Y K D Q Q G N N V A T I I N V H M
Sequence135-P-CG-Col-ABCCC     - - - - - - - - - - - V S L L Y A G N G G F G A K H D W N A T V G Y K D Q Q G N N V A T I I N V H M
Sequence138-NP-GA-Col-ABC      - - - - - - - - - - - V S L L Y A G N G G F G A K H D W N A T V G Y K D Q Q G N N V A T I I N V H M
Sequence13-NP-UP-Jp-ABD        - - - - - - - - - - - V S L L Y G D N G G P E A R H D W N A T V G Y K N Q Q G S N V A T L I N A H L
Sequence11-NP-UP-Jp-ABD        - - - - - - - - - - - V S L L Y G D N G G P E A R H D W N A T V G Y K D Q Q G N N V A T L I N V H L
Sequence50-NP-GA-OCC-ABC       - - - - - - - - - - - V S L L Y G D N G G P E A R H D W N A T V G Y K N Q Q G N N V A T L I N A H L
Sequence26-NP-GA-Jp-ABD        - - - - - - - - - - - V S L L Y G D N G G P E A R H D W N A T V G Y K N Q Q G N N V A T L I N A H L
Sequence55-NP-UD-OCC-ABCCC     - - - - - - - - - - - V S L L Y G G N G G P E A R H D W N A T V G Y K N Q Q G D N V A T L I N V H M
Sequence178-NP-M-Col-ABCC      - - - - - - - - - - - V S L L Y A G N G G F G A K H D W N A T V G Y K D Q Q G N N V A T I I N V H M
Sequence40-NP-GA-Jp-ABD        - - - - - - - - - - - V S L L Y G D N G G P E A R H D W N A T V G Y K N Q Q G S N V A T L I N A H L
Sequence43-NP-GA-Jp-ABD        - - - - - - - - - - - V S L L Y G D N G G P E A R H D W N A T V G Y K D Q Q G S N V A T L I N A H L
Sequence139-NP-UD-Col-ABCC     - - - - - - - - - - - V S L L Y A G N G G F G A K H D W N A T V G Y K D Q Q G N N V A T I I N V H M
Sequence7-P-CG-Jp-ABBD         - - - - - - - - - - - V S L L Y G D N G G P E A R H D W N A T V G Y K N Q Q G S N V A T L I N A H L
Sequence91-P-CG-Vit-ABD        - - - - - - - - - - - V S L L Y G D N G G P E A R H D W N A T V G Y K N Q Q G N N V A T L I N A H L
Sequence29-NP-GA-Jp-ABABD       - - - - - - - - - - - V S L L Y G D N G G P E A R H D W N A T V G Y K N Q Q G S N V A T L I N A H L
Sequence78-P-CG-EA-ABD         - - - - - - - - - - - V S L L Y G G N G G P E A R H D W N A T V G Y K N Q Q G N N V A T L I N A H L
Sequence188-NP-GCA-Jp-AB       - - - - - - - - - - - V S L L Y G G N G G P G A R H D W N A T V G Y K D Q Q G N N V A T I I N V H M
Sequence72-P-CG-EA-ABD         - - - - - - - - - - - V S L L Y G D N G G P E A R H D W N A T V G Y K N Q Q G N N V A T L I N A H L
Sequence79-P-CG-EA-ABD         - - - - - - - - - - - V S L L Y G D N G G P E A R H D W N A T V G Y K N Q Q G N N V A T L I N A H L
Sequence61-NP-UD-OCC-ABC       - - - - - - - - - - - V S L L Y G G N G G P E A R H D W N A T V G Y K N Q Q G N N V A T L I N A H L
Sequence125-NP-UG-Jp-ABCC      - - - - - - - - - - - V S L L Y A G N G G F G A K H D W N A T V G Y K D Q Q G N N V A T I I N V H M
Sequence175-NP-G-Col-ABCC      - - - - - - - - - - - V S L L Y A G N G G F G D K H D W N A T V G Y K D Q Q G D N V A T I I N V H M
Sequence122-NP-G-Jp-ABCC       - - - - - - - - - - - V S L L Y A G N G G F G A K H D W N A T V G Y K D Q Q G N N V A T I I N V H M
Sequence36-NP-GA-Jp-ABD        - - - - - - - - - - - V S L L Y G D N G G P E A R H D W N A T V G Y K N Q Q G N N V A T L I N A H L
Sequence58-NP-UD-OCC-ABC       - - - - - - - - - - - V S L L Y G G N G G P E A R H D W N A T V G Y K N Q Q G D N V A T L I N V H M
Sequence27-NP-GA-Jp-ABBD       - - - - - - - - - - - V S L L Y G D N G G P E A R H D W N A T V G Y K D Q Q G N N V A T L I N A H L
Sequence93-P-CG-Vit-ABD        - - - - - - - - - - - S F I I V W G N G G P E A R H D W N A T V G Y K N Q Q G N N V A T L I N A H L
Sequence177-NP-M-Col-ABCC      - - - - - - - - - - - V S L L Y A G N G G F G A K H D W N A T V G Y K D Q Q G N N V A T I I N V H M
Sequence182-NP-UD-Col-ABCC     - - - - - - - - - - - V S L L Y A G N G G F G D K H D W N A T V G Y K D Q Q G N N V A T I I N V H M
Sequence114-NP-UG-Jp-ABC       - - - - - - - - - - - V S L L Y A G N G G F G A K H D W N A T V G Y K D Q Q G N N V A T I I N V H M
Sequence74-P-CG-EA-ABD         - - - - - - - - - - - V S L L Y G D N G G P E A R H D W N A T V G Y K N Q Q G N N V A T L I N A H L
Sequence20-P-CG-Jp-ABD         - - - - - - - - - - - V S L L Y G D N G G P E S R H D W N A T V G Y K D Q Q G S N V A T L I N A H L
Sequence14-NP-GCA-Jp-ABD       - - - - - - - - - - - V S L L Y G D N G G P E A R H D W N A T V G Y K D Q Q G N N V A T L I N A H L
Sequence39-NP-GA-Jp-ABC        - - - - - - - - - - - V S L L Y A G N G G F G A K H D W N A T V G H K D Q Q G N N V A T I I N V H M
Sequence170-NP-UD-Col-ABCC     - - - - - - - - - - - V S L L Y A G N G G F G D K H D W N A T V G Y K D Q Q G N N V A T I I N V H M
Sequence76-P-CG-EA-ABD         - - - - - - - - - - - V S L L Y G D N G G P E A R H D W N A T V G Y K N Q Q G N N V A T L I N V H L
Sequence53-NP-GA-EA-ABD        - - - - - - - - - - - V S L L Y G D N G G F E A R H D W N A T V G Y K N Q Q G N N V A T L I N A H L
Sequence174-P-CG-Col-ABCC      - - - - - - - - - - - V S L L Y A G N G G F G A K H D W N A T V G Y K D Q Q G N N V A T I I N V H M
Sequence33-NP-UG-Jp-ABD        - - - - - - - - - - - V S L L Y A G N G G F G A K H D W N A T V G Y K D Q Q G N N V A T I I N V H M
Sequence60-NP-GA-EA-ABD        - - - - - - - - - - - V S L L Y G D N G G P E A R H D W N A T V G Y K N Q Q G S N V A T L I N A H L
Sequence38-NP-GA-Jp-ABD        - - - - - - - - - - - V S L L Y G D N G G L E A R H D W N A T V G Y K N Q Q G N N V A T L I N A H L
Sequence1-NP-EG-Jp-ABCCC       - - - - - - - - - - - V S L L F A G N G G F G A K H D W N A T V G Y K N Q Q G D N V A T L I N V H M
Sequence101-P-CG-Vit-ABD       - - - - - - - - - - - V S L L D G D N G G P E A R H D W N A T V G Y K N Q Q G N N V A T L I N A H L
Sequence64-NP-UD-OCC-ABC       - - - - - - - - - - - V S L L Y G G N G G P E A R H D W N A T V G Y K N Q Q G N N V A T L I N V H M
Sequence81-P-CG-EA-ABD         - - - - - - - - - - - V S L L Y G G N G G P E A R H D W N A T V G Y K N Q Q G N N V A T L I N V H M
Sequence150-NP-DA-Sen-BC       - - - - - - - - - - - V S L L Y A G N G G F G A K H D W N A T V G Y K D Q Q G N N V A T I I N V H M
Sequence90-NP-UD-Vit-ABD       - - - - - - - - - - - V S L L Y G D N G G P E A R H D W N A T V G Y K N Q Q G S N V A T L I N A H L
Sequence136-P-CG-Col-AACC      - - - - - - - - - - - V S L L Y A G N G G F G A K H D W N A T V G Y K D Q Q G N N V A T I I N M H M
Sequence15-NP-UP-Jp-ABD        - - - - - - - - - - - V S L L Y G D N G G P E A R H D W N A T V G Y K N Q Q G N N V A T L I N A H L
```

```
Sequence108-NP-UD-Vit-ABD     -----------VSLLYGDNGGPEARHDWNATVGYKDQQGSNVATLINAHL
Sequence104-P-CG-OCC-ABCC     -----------VSLLYAGNGGFGAKHDWNATVGYKDQQGNNVATIINVHM
Sequence179-NP-UD-Col-ABCC    -----------VSLLYAGNGGFGDKHDWNATVGYKDQQGNNVATIINVHM
Sequence37-NP-GA-Jp-ABCCC     -----------VSLLYGGNGGPEARHDWNATVGYKNQQGDNVATLINVHM
Sequence89-NP-UD-Vit-ABD      -----------VSLLYGDNGGPEARHDWNATVGYKDQQGNNVATLINVHL
Sequence92-P-CG-Vit-ABD       -----------VSLLYGDNGGPEARHDWNATVGYKNQQGNNVATLINAHL
Sequence49-NP-GA-Jp-ABC       -----------VSLLYGGNGGPEARHDWNATVGYKNQQGDNVATLINVHM
Sequence181-P-CG-Col-ABCC     -----------VSLLYAGNGGFGAKHDWNATVGYKDQQGNNVATIINVHM
Sequence25-NP-GA-Jp-ABD       -----------VSLLYGDNGGPEARHDWNATVGYKNQQGDNVATLINAHL
Sequence4-P-CG-Jp-ABD         -----------VSLLYGDNGGPEARHDWNATVGYKNQQGNNVATLINAHL
Sequence121-NP-G-Jp-ABCC      -----------VSLLYGGNGGPGARHDWNATVGYKDQQGNNVATIINVHM
4dvy                          -----------VSLLYAGNGGFGDKHDWNATVGYKDQQGNNVATLINVHM
Sequence186-NP-UP-Jp-ABD      -----------VSLLYGDNGGPEARHDWNATVGYKNQQGSNVATLINAHL
Sequence62-NP-UD-OCC-ABD      -----------VSLLYGGNGGPEARHDWNATVGYKNQQGNNVATLINVHM
Sequence9-NP-GCA-Jp-ABD       -----------ISLLYGDNGGPEARHDWNATVGYKNQQGNNVATLINAHL
Sequence12-NP-UP-Jp-ABD       -----------VSLLYGDNGGPEARHDWNATVGYKNQQGSNVATLINAHL
Sequence35-NP-GA-Jp-ABABD     -----------VSLLYGDNGGPEARHDWNATVGYKDQQGNNVATLINAHL
Sequence54-NP-GA-OCC-ABC      -----------VSLLYGGNGGPEARHDWNATVGYKNQQGDNVATLINVHM
Sequence109-P-CG-Jp-ABD       -----------VSLLYGDNGGPEARHDWNATVGYKDQQGNNVATLINAHL
Sequence96-NP-UD-Vit-ABD      -----------VSLLYGDNGGPEARHDWNATVGYKNQQGSNVATLINAHL
Sequence80-P-CG-EA-ABD        -----------VSLLYGDNGGPEARHDWNATVGYKNQQGNNVATLINAHL
Sequence82-P-CG-EA-ABD        -----------VSLLYGDNGGPEARHDWNATVGYKNQQGNNVATLINAHL
Sequence10-NP-GCA-Jp-ABD      -----------VSLLYGDNGGPEARHDWNATVGYKNQQGNNVATLINAHL
Sequence19-NP-UP-Jp-ABBD      -----------VSLLYGDNGGPEARHDWNATVGYKNQQGNNVATLISAHL
Sequence115-NP-UG-Jp-ABC      -----------VSLLYAGNGGFGAKHDWNATVGYKDQQGNNVATIINVHM
Sequence45-NP-GA-Jp-ABCC      -----------VSLLYAGNGGFGAKHDWNATVGYKDQQGNNVATIINVHM
Sequence119-NP-UG-Jp-ABC      -----------VSLLYAGNGGFGAKHDWNATVGYKDQQGNNVATIINVHM
Sequence70-NP-GA-Ch-ABD       -----------VSLLYGDNGGPEARHDWNATVGYKNQQGNNVATLINAHL
Sequence52-NP-UD-EA-ABD       -----------VSLLYGDNGGPEARHDWNATVGYKNQQGNNVATLINVHL
Sequence97-P-CG-Vit-ABD       -----------VSLLYGDNGGPEARHDWNATVGYKNQQGNNVATLINAHL
Sequence94-NP-UD-Vit-ABD      CKSFITCMGIMVVLKLGMIGTPPLVIKTNKAVMWPH---SLVRILITAV-
Sequence176-NP-GA-Col-ABCC    -----------VSLLYAGNGGFGAKHDWNATVGYKDQQGNNVATIINVHM
Sequence68-NP-D-Am-ABBC       -----------VSLLYGGNGGPEARHDWNATVGYKDQQGNNVATIINVHM
Sequence47-NP-GA-Jp-ABD       -----------ASLLYGDNGGPEARHDWNATVGYKNQQGNNVATLINAHL
Sequence56-NP-UD-OCC-ABC      -----------VSLLYGGNGGPEARHDWNATVGYKNQQGDNVATLINVHM
Sequence31-NP-GA-Jp-ABD       -----------VSLLYGDNGGPEARHDWNATVGYKDQQGSNVATLINAHL
Sequence46-NP-UD-Jp-ABD       -----------VSLLYGGNGGPEARHEWNATVGYKNQQGDNVATLINVHM
Sequence102-NP-UG-Vit-ABD     -----------VPFCLGKMAVLKLGMIRNATVGYKNQQGNNVATLINAHL
Sequence71-P-CG-EA-ABD        -----------VSLLYGDNGGPEARHDWNATVGYKNQQGNNVATLINAHL
Sequence30-NP-GA-Jp-ABBD      -----------VSLLYGDNGGPEARHDWNATVGYKDQQGNNVATLINAHL
Sequence28-NP-GA-Jp-AABD      -----------VSLLYGDNGGPEARHDWNATVGYKNQQGNNVATLINAHL
Sequence98-P-CG-Vit-AD        -----------VSLLYGDNGGPEARHDGNATVGYKDQQGNNVATLINAHL
Sequence99-P-CG-Vit-ABD       -----------VSLLYGDNGGPEARHDWNATVGYKDQQGNNVATLINAHL
Sequence24-NP-UD-USA-ABD      -----------VSLLYGDNGCPEARHDWNATVGYKNQQGNNVATLINAHL
Sequence124-NP-G-Jp-ABC       -----------VSLLYAGNGGFGAKHDWNATVGYKDQQGNNVATIINVHM
Sequence77-P-CG-OCC-ABCC      -----------VSLLYGGNGGPEARHDWNATVGYKNQQGSNVATLINVHM
Sequence113-NP-UG-Jp-ABC      -----------VSLLYAGNGGFGAKHDWNATVGYKDQQGNNVATIINVHM
Sequence88-NP-UD-Vit-ABD      -----------SFIIVWGNGGPEARHDWNATVGYQDQQGSNVATLINAHL
Sequence100-NP-UD-Vit-ABD     -----------VSLLYGDNGGPEARHDWNASVGYKDQQGSNVATLINPHL
Sequence137-NP-G-Col-ABCC     -----------VSLLYAGNGGFGAKHDWNATVGYKDQQGNNVATIINVHM
Sequence117-NP-G-Jp-ABC       -----------VSLLYGGNGGPGARHDWNATVGYKDQQGNNVATIINVHM
Sequence171-NP-UD-Col-ABCC    -----------VSLLYAGNGGFGAKHDWNATVGYKDQQGNNVATIINVHM
Sequence180-P-CG-Col-ABC      -----------VSLLYAGNGGFGAKHDWNATVGYKDQQGNNVATIINVHM
Sequence112-P-CG-Jp-ABCC      -----------VSLLYGGNGGPGARHDWNATVGYKDQQGNNVATIINIHM
Sequence127-NP-UD-Jp-ABC      -----------VSLLYGGNGGPGARHDWNATVGYKDQQGNNVATLINVHM
Sequence51-NP-GA-OCC-ABC      -----------VSLLYGGNGGPEARHDWNATVGYKNQQGNNVATLINVHM
Sequence73-P-CG-EA-ABD        -----------VSLLYGDNGGPEARHDWNATVGYKNQQGNNVATLINAHL
Sequence183-NP-UD-Col-ABD     -----------VSLLYAGNGGFGDKHDWNATVGYKDQQGNNVATIINVHM
Sequence34-NP-UD-Jp-ABDD      -----------VSLLYGDNGGPRARHDWNATVGYKDQQGNNVATLINAHL
Sequence21-P-CG-Jp-ABD        -----------VSLLYGDNGGPEARHDWNATVGYKDQQGNNVATLINAHL
Sequence32-NP-GA-Jp-ABD       -----------VSLLYGDNGGPEARHDWNATVGYKNQQGSNVATLINAHL
```

Sequence116-NP-UD-Jp-ABC - - - - - - - - - - - V S L L Y A G N G G F G A K H D W N A T V G Y K D Q Q G N N V A T I I N V H M
Sequence8-NP-GCA-Jp-ABD - - - - - - - - - - - V S L L Y G D N G G P E A R H D W N A T V G Y K N Q Q G N N V A T L I N A H L
Sequence83-P-CG-EA-ABD - - - - - - - - - - - V S L L Y G D N G G P E A R H D W N A T V G Y K N Q Q G N N V A T L I N A H L
Sequence48-NP-UD-OCC-ABC - - - - - - - - - - - V S L L Y G G N G G P E A R H D W N A T V G Y K N Q Q G D N V A T L I N V H M
Sequence120-NP-G-Jp-ABC - - - - - - - - - - - V S L L Y A G N G G F G A K H D W N A T V G Y K D Q Q G N N V A T I I N V H M
Sequence187-NP-UG-Jp-ABCCC - - - - - - - - - - - V S L L Y A G N G G F G A K H D W N A T V G Y K N Q Q G D N V A T L I N V H M
Sequence118-NP-G-Jp-ABD - - - - - - - - - - - V S L L Y G D N G G S E A R H D W N A T V G Y K N Q Q G N N V A T L I N A H L
Sequence87-NP-UG-Vit-ABD - - - - - - - - - - - F P L L Y G D N G G P E A R H D W N A T V G Y K D Q Q G S N V A T L I N A H L

401 450

Sequence140-NP-GA-Col-ABABC K N G S G L V I A G G E K G I N N P S F Y L Y K E D Q L T G S Q R A L S Q E E I R N K I D F M - - E
Sequence123-NP-UD-Jp-ABD N N G S G L I I A G N E N G I K N P S F Y L Y K E D Q L T G L K Q A M S Q E E I Q N K V D F M - - E
Sequence111-NP-G-Jp-ABD N N G S G L V I A G N E N G I K N P S F Y L Y K E D Q L T G L K Q A M S Q E E I Q N K V D F M - - E
Sequence57-NP-GA-OCC-AB K N G S G L V I A G G E K G V N N P S F Y L Y K E D Q L T G L K Q A L S Q E E I Q N K V D F M - - E
Sequence172-P-CG-Col-ABCC K N G S G L V I A G G E K G I N N P S F Y L Y K E D Q L T G S Q R A L S Q E E I R N K I D F M - - E
Sequence110-NP-UD-Jp-ABD N N G S G L I I A G N E N G I K N P S F Y L Y K E D Q L T G L K Q A M S Q E E I Q N K V D F M - - E
Sequence95-NP-UD-Vit-ABD N N G S G L I I A G N E D G I K N P S F Y L Y K E D Q L T G L K Q A M S Q E E I Q N K V D F M - - E
Sequence107-P-CG-Vit-ABD N N G S G L I I A G N E D G I K N P S F Y L Y K E D Q L T G L K Q A M S Q E E I Q N K V D F M - - E
Sequence106-P-CG-Vit-ABD H N G S G L L I A G N E N G I K N P S F Y L Y K E D Q L T G S E T S N E S R R D P K Q S G F H - - G
Sequence105-NP-UD-Vit-ABD N N G S G L I I A G N E D G I K N P S F Y L Y K E D Q L T G L K Q A L S Q E E I Q N K V D F M - - E
Sequence126-NP-UG-Jp-ABC K N G S G L V I A G G E K G I N N P S F Y L Y K E D Q L T G S Q R A L S Q E E I R N K I D F M - - E
Sequence135-P-CG-Col-ABCCC K N G S G L V I A G G E K G I N N P S F Y L Y K E D Q L T G S Q R A L S Q E E I Q N K I D F M - - E
Sequence138-NP-GA-Col-ABC K N G S G L V I A G G E K G I N N P S F Y L Y K E D Q L T G S Q R A L S Q E E I R N K I D F M - - E
Sequence13-NP-UP-Jp-ABD N N G S G L I I A G N K D G I K N P S F Y L Y K E D Q L T G L K Q A L S Q E E I Q N K V D F M - - E
Sequence11-NP-UP-Jp-ABD N N G S G L I I A G N E D G I K N P S F Y L Y K E D Q L T G L K Q A M S Q E E I Q N K V D F M - - E
Sequence50-NP-GA-OCC-ABC N N G S G L V I A G N E N G I K N P S F Y L Y K E D Q L T G L K Q A L S Q E E I Q N K V D F M - - E
Sequence26-NP-GA-Jp-ABD N N G S G L I I V G N E N G I K N P S F Y L Y K E D Q L T G L K Q A L S Q E E I Q N K V D F M - - E
Sequence55-NP-UD-OCC-ABCCC K N G S G L V I A G G E K G V N N P S F Y L Y K E D Q L T G L K Q A L S Q E E I Q N K V D F M - - E
Sequence178-NP-M-Col-ABCC K N G S G L V I A G G E K G I N N P S F Y L Y K E D Q L T G S Q R A L S Q E E I Q N K I D F M - - E
Sequence40-NP-GA-Jp-ABD H N G S G L V I A G N E D G I K N P S F Y L Y K E D Q L T G L K Q A M S Q E E I Q N K V D F M - - E
Sequence43-NP-GA-Jp-ABD N N G S G L I I A G N E N G I K N P S F Y L Y K E D Q L T G L K Q A L S Q E E I Q N K V D F M - - E
Sequence139-NP-UD-Col-ABCC K N G S G L V I A G G E K G I N N P S F Y L Y K E D Q L T G S Q R A L S Q E E I R N K I D F M - - E
Sequence7-P-CG-Jp-ABBD N N G S G L V I A G N E N G I K N P S F Y L Y K E D Q L T G L K Q A L S Q E E I Q N K V D F M - - E
Sequence91-P-CG-Vit-ABD N N G S G L V I A G N E D G I K N P S F Y L Y K E D Q L T G L K Q A M S Q E E I Q N I V D F M - - E
Sequence29-NP-GA-Jp-ABABD N N G S G L V I A G N E N G I K N P S F Y L Y K E D Q L T G L K Q A L S Q E E I Q N K V D F M - - E
Sequence78-P-CG-EA-ABD N N G S G L V I A G N E D G I K N P S F Y L Y K E D Q L T G L K Q A L S Q K E I Q N K V D F M - - E
Sequence188-NP-GCA-Jp-AB K N G S G L V I A G G E K G I N N P S F Y L Y K E D Q L T G S Q R A L S Q E E I Q N K I D F M - - E
Sequence72-P-CG-EA-ABD N N G S G L V I A G N E D G I K N P S F Y L Y K E D Q L T G L K Q A M S Q E E I Q N K V D F M - - E
Sequence79-P-CG-EA-ABD N N G S G L I I A G N E D G I K N P S F Y L Y K E D Q L T G L K Q A L S Q E E I Q N K V D F M - - E
Sequence61-NP-UD-OCC-ABC N N G S G L I I A G N E D G I K N P S F Y L Y K E D Q L T G L K Q A L S Q E E I Q N K V D F M - - E
Sequence125-NP-UG-Jp-ABCC K N G S G L V I A G G E K G I N N P S F Y L Y K E D Q L T G S Q R A L S Q E E I R N K V D F M - - E
Sequence175-NP-G-Col-ABCC K N G S G L V I A G G E K G I N N P S F Y L Y K E D Q L T G S Q R A L S Q E E I R N K I D F M - - E
Sequence122-NP-G-Jp-ABCC K N G S G L V I A G G E K G I N N P S F Y L Y K E D Q L T G S Q R A L S Q E E I R N K V D F M - - E
Sequence36-NP-GA-Jp-ABD N N G S G L V I A G N E D G I K N P S F Y L Y K E D Q L T G L K Q A M S Q E E I Q N K V D F M - - E
Sequence58-NP-UD-OCC-ABC K N G S G L V I A G G E K G V N N P S F Y L Y K E D Q L T G L K Q A L S Q E E I Q N K V D F M - - E
Sequence27-NP-GA-Jp-ABBD N N G S G L I I V G N E D G I K N P S F Y L Y K T D Q L T G L K Q A L S Q E E I Q N K V D F M - - E
Sequence93-P-CG-Vit-ABD H N G S G L I I A G N E N G I K N P S F Y L Y K E D Q L T G L K Q A M S Q E E I Q N K V D F M - - E
Sequence177-NP-M-Col-ABCC K N G S G L V I A G G E K G I N N P S F Y L Y K E D Q L T G S Q R A L S Q E E I R N K I D F M - - E
Sequence182-NP-UD-Col-ABCC K N G S G L V I A G G E K G I N N P S F Y L Y K E D Q L T G S Q R A L S Q E E I L N K V D F M - - E
Sequence114-NP-UG-Jp-ABC K N G S G L V I A G G E K G I N N P S F Y L Y K E D Q L T G S Q R A L S Q E E I R N K V D F M - - E
Sequence74-P-CG-EA-ABD N N G S G L V I A G N E D G I K N P S F Y L Y K E D Q L T G L K Q A M S Q E E I Q N K V D F M - - E
Sequence20-P-CG-Jp-ABD N N G S G L V I A G N E E G I K N P S F Y L Y K Q D Q L T G L K Q A M S Q E E I Q N K V D F M - - E
Sequence14-NP-GCA-Jp-ABD N N G S G L I I A G N E D G I K N P S F Y L Y K E D Q L T G L K Q A W S Q K E I Q N K V D F M - - E
Sequence39-NP-GA-Jp-ABC K N G S G L V I A G G E K G I N N P S F Y L Y K E D Q L T G S Q R A L S Q E E I L N K V D F M - - E
Sequence170-NP-UD-Col-ABCC K N G S G L V I A G G E K G I N N P S F Y L Y K E D Q L T G S Q R A L S Q E E I L N K I D F M - - E
Sequence76-P-CG-EA-ABD N N G S G L V I A G N E D G I K N P S F Y L Y K E D Q L T G L K Q A M S Q E E I Q N K V D F M - - E
Sequence53-NP-GA-EA-ABD N N G S G L V I A G N E D G I K N P S F Y L Y K E D Q L T G L K Q A M S Q E E I Q N K V D F M - - E
Sequence174-P-CG-Col-ABCC K N G S G L V I A G G E K G I N N P S F Y L Y K E D Q L T G S Q R A L S Q E E I R N K I D F M - - E
Sequence33-NP-UG-Jp-ABD K N G S G L V I A G G E K G I N N P S F Y L Y K E D Q L T G S Q R A L S Q E E I Q N K I D F M - - E
Sequence60-NP-GA-EA-ABD N N G S G L V I A G N E N G I K N P S F Y L Y K E D Q L T G L K Q A L S Q E E I Q N K V D F M - - E
Sequence38-NP-GA-Jp-ABD N N G S G L V I A G G E K G V N N P S F Y L Y K E D Q L T G L K Q A M S Q E E I Q N K V D F M - - E

| Sequence | Alignment |
| --- | --- |
| Sequence1-NP-EG-Jp-ABCCC | KNGSGLVIAGGEKGINNPSFYLYKEDQLTGSQRALSQEEIRNKIDFM--E |
| Sequence101-P-CG-Vit-ABD | NNGSGLVIAGNEDGIKNPSFYLYKEDQLTGLKQAMSQEEIQNKVDFM--E |
| Sequence64-NP-UD-OCC-ABC | KNGSGLVIAGGEKGVNNPSFYLYKEDQLTGLKQALSQKEIQNKVDFM--E |
| Sequence81-P-CG-EA-ABD | KNGSGLVIAGGEKGVNNPSFYLYKEDQLTGLKQALSQKEIQNKVDFM--E |
| Sequence150-NP-DA-Sen-BC | KNGSGLVIAGGEKGINNPSFYLYKEDQLTGSQRALSQEEIRNKIDFM--E |
| Sequence90-NP-UD-Vit-ABD | NNGSGLVIAGNEDGIKNPSFYLYKEDQLTGLKQAMSQEEIQNKVGFM--E |
| Sequence136-P-CG-Col-AACC | KNGSGLVIAGGEKGINNPSFYLYKEDQLTGSQRALSQEEIRNKIDFM--E |
| Sequence15-NP-UP-Jp-ABD | NNGSGLVIAGNEDGIKNPSFYLYKEDQLTGLKQALSQEEIRNKVDFM--E |
| Sequence108-NP-UD-Vit-ABD | YNGSGLIIAGNEDGIKNPSFYLYKEDQLTGLKQAMSQEEIQNKVDFL--E |
| Sequence104-P-CG-OCC-ABCC | KNGSGLVIAGGEKGINNPSFYLYKEDQLTGSQRALSQEEIRNKIDFM--E |
| Sequence179-NP-UD-Col-ABCC | KNGSGLVIAGGEKGINNPSFYLYKEDQLTGSQRALSQEEIQNKIDFM--E |
| Sequence37-NP-GA-Jp-ABCCC | KNGSGLVIAGGEKGVNNPSFYLYKEDQLTGLKQALSQEEIKNKVDFM--E |
| Sequence89-NP-UD-Vit-ABD | YNGSGLVIAGNEDGIKNPSFYLYKEDQLTGLKQAMSQEEIQNKVDFM--E |
| Sequence92-P-CG-Vit-ABD | NNGSGLVIAGNEDGIKNPSFYLYKEDQLTGLKQAMSQEEIQNKVDFM--E |
| Sequence49-NP-GA-Jp-ABC | KNGSGLVIAGGEKGVNNPSFYLYKEDQLTGLKQALSQEEIQNKVDFM--E |
| Sequence181-P-CG-Col-ABCC | KNGSGLVIAGGEKGINNPSFYLYKEDQLTGSQRALSQEEIRNKIDFM--E |
| Sequence25-NP-GA-Jp-ABD | NNGSGLVIAGNENGIKNPSFYLYKEDQLTGLKQALSQEEIQNKVDFM--E |
| Sequence4-P-CG-Jp-ABD | NNGSGLIIAGNEDGIKNPSFYLYKEDQLTGLKQALSQEEIQNKVDFM--E |
| Sequence121-NP-G-Jp-ABCC | KNGSGLVIAGGEKGINNPSFYLYKEDQLTGSQRALSQEEIRNKIDFM--E |
| 4dvy | KNGSGLVIAGGEKGINNPSFYLYKEDQLTGSQRALSQEEIRNKVDFM--E |
| Sequence186-NP-UP-Jp-ABD | NNGSGLIIAGNENGIKNPSFYLYKEDQLTGLKQALSQEEIQNKVDFM--E |
| Sequence62-NP-UD-OCC-ABD | KNGSGLVIAGGEKGVNNPSFYLYKEDQLTGLKQALSQKEIQNKVDFM--E |
| Sequence9-NP-GCA-Jp-ABD | NNGSGLIIAGNEDGIKNPSFYLYKEDQLTGLKQALSQEEIQNKVDFM--E |
| Sequence12-NP-UP-Jp-ABD | NNGSGLVIAGNENGIKNPSFYLYKEDQLTGLKQALSQEEIQNKVDFM--E |
| Sequence35-NP-GA-Jp-ABABD | NNGSGLIIAGNEDGIKNPSFYLYKTDQLTGLKQALSQEEIQNKVDFM--E |
| Sequence54-NP-GA-OCC-ABC | KNGSGLVIAGGEKGVNNPSFYLYKEDQLTGLKQALSQEEIQNKVDFM--E |
| Sequence109-P-CG-Jp-ABD | HNGSGLVIAGNENGIKNPSFYLYKEDQLTGLKQAMSQEEIQNKVDFM--E |
| Sequence96-NP-UD-Vit-ABD | CNGSGLVIAGNEDGIKNRSFYLYKEDQLTGLKQAMSQEESQNKVDFM--E |
| Sequence80-P-CG-EA-ABD | NNGSGLIIAGNEDGIKNPSFYLYKEDQLTGLKQALSQEEIQNKVDFM--E |
| Sequence82-P-CG-EA-ABD | NNGSGLVIAGNEDGIKNPSFYLYKEDQLTGLKQAMSQEEIQNKVDFM--E |
| Sequence10-NP-GCA-Jp-ABD | NNGSGLIIAGNEDGIKNPSFYLYKTDQLTGLKQALSQEEIQNKVDFM--E |
| Sequence19-NP-UP-Jp-ABBD | NNGSGLVIAGNENGIKNPSFYLYKEDQLTGLKQAMSQEEIQNKVDFM--E |
| Sequence115-NP-UG-Jp-ABC | KNGSGLVIAGGEKGINNPSFYLYKEDQLTGSQRALSQEEIRNKIDFM--E |
| Sequence45-NP-GA-Jp-ABCC | KNGSGLVIAGGEKGINNPSFYLYKEDQLTGSQRALSQEEIRNKVDFM--E |
| Sequence119-NP-UG-Jp-ABC | KNGSGLVIAGGEKGINNPSFYLYKEDQLTGSQRALSQEEIRNKIDFM--E |
| Sequence70-NP-GA-Ch-ABD | NNGSGLIIAGNENGIKNPSFYLYKEDQLTGLKQALSQEEIQNKVDFM--E |
| Sequence52-NP-UD-EA-ABD | NNGSGLVIAGNENGIKNPSFYLYKEDQLTGLKQALSQEEIQNKVDFM--E |
| Sequence97-P-CG-Vit-ABD | NNGSGLIIAGNEDGIKNPSFYLYKEDQLTGLKQAMSQEEIQNKVDFM--E |
| Sequence94-NP-UD-Vit-ABD | --GKSLRVM--KMGLKTLASISTKKTNSQACNKQLSPEK-RSKTKWVSWN |
| Sequence176-NP-GA-Col-ABCC | KNGSGLVIAGGEKGINNPSFYLYKEDQLTGSQRALSQEEIRNKIDFM--E |
| Sequence68-NP-D-Am-ABBC | KNGSGLVIAGGEKGINNPSFYLYKEDQLTGSQRALSQEEIRNKVDFM--E |
| Sequence47-NP-GA-Jp-ABD | NNGSGLVIAGNEDGIKNPSFYLYKEDQLTGLKQAMSQEEIQNKVDFM--E |
| Sequence56-NP-UD-OCC-ABC | KNGSGLVIAGGEKGVNNPSFYLYKEDQLTGLKQALSQEEIQNKVDFM--E |
| Sequence31-NP-GA-Jp-ABD | NNGSGLIIAGNEDGIKNPSFYLYKEDQLTGLKQAMSQEEIQNKVDFM--E |
| Sequence46-NP-UD-Jp-ABD | KNGSGLVIAGGEKGVNNPSFYLYKEDQLTGLKQALSQEEIQNKVDFM--E |
| Sequence102-NP-UG-Vit-ABD | NNGSGLVIAGNEDGIKNPSFYLYKEDQLTGLKQAMSQEEIQNKVDFM--E |
| Sequence71-P-CG-EA-ABD | NNGSGLVIAGNEDGIKNPSFYLYKEDQLTGLKQAMSQEEIQNKVDFM--E |
| Sequence30-NP-GA-Jp-ABBD | NNGSGLIIAGNEDGIKNPSFYLYKEDQLTGLKQALSQEEIQNKVDFM--E |
| Sequence28-NP-GA-Jp-AABD | HNGSGLVIAGNEDGIKNPSFYLYKADQLTGLKQAMSQEEIQNKVDFM--E |
| Sequence98-P-CG-Vit-AD | NNGSGLIIAGNEDGIKNPSFYLYKEDQPTGLKQAMSQEEIQNKVDFM--E |
| Sequence99-P-CG-Vit-ABD | HNGSGLIISGNENGIKNPSFYLYKEDQLTGLKQALSQEEIQNKVDFM--E |
| Sequence24-NP-UD-USA-ABD | NNGSGLIIAGNEDGIKNPSFYLYKEDQLTGLKQAMSQEEIQNKVDFM--E |
| Sequence124-NP-G-Jp-ABC | KNGSGLVIAGGEKGINNPSFYLYKEDQLTGSQRALSQEEIRNKIDFM--E |
| Sequence77-P-CG-OCC-ABCC | KNGSGLVIAGGEKGVNNPSFYLYKEDQLTGLKQALSQKEIQNKVDFM--E |
| Sequence113-NP-UG-Jp-ABC | KNGSGLVIAGGEKGINNPSFYLYKEDQLTGSQRALSQEEIRNKVDFM--E |
| Sequence88-NP-UD-Vit-ABD | NNGNGLIIAGNEDGIKNPSFYLYKEDQLTGLKQAMSQEEIQNKVDFM--E |
| Sequence100-NP-UD-Vit-ABD | YNGSGLIIAGNEDGIKNPSFYLYKEDQLTGLKQAMSQEEIQNKVDFM--E |
| Sequence137-NP-G-Col-ABCC | KNGSGLVIAGGEKGINNPSFYLYKEDQLTGSQRALSQEEIRNKIDFM--E |
| Sequence117-NP-G-Jp-ABC | KNGSGLVIAGGEKGINNPSFYLYKEDQLTGSQRALSQEEIRNKIDFM--E |
| Sequence171-NP-UD-Col-ABCC | KNGSGLVIAGGEKGINNPSFYLYKEDQLTGSRRALSQEEIRNKIDFM--E |
| Sequence180-P-CG-Col-ABC | KNGSGLVIAGGEKGINNPSFYLYKEDQLTGSQRALSQEEIRNKIDFM--E |

```
Sequence112-P-CG-Jp-ABCC       KNGSGLVIAGGEKGINNPSFYLYKEDQLTGSQRALSQEEIRNKIDFM--E
Sequence127-NP-UD-Jp-ABC       KNGSGLVIAGGEKGINNPSFYLYKEDQLTGSQRALSQEEIRNKIDFM--E
Sequence51-NP-GA-OCC-ABC       KNGSGLVIAGGEKGVNNPSFYLYKEDQLTGLKQALSQKEIQNKVDFM--E
Sequence73-P-CG-EA-ABD         NNGSGLVIVGNEDGIKNPSFYLYKEDQLTGLKQAMSQEEIQNKVDFM--E
Sequence183-NP-UD-Col-ABD      KNGSGLVIAGGEKGINNPSFYLYKEDQLTGSQRALSQEEIRNKIDFM--E
Sequence34-NP-UD-Jp-ABDD       NNGSGLIIAGNEDGIKNPSFYLYKEDQLTGLKQALSQEEIQNKVDFM--E
Sequence21-P-CG-Jp-ABD         NNGSGLIIAGNENGIKNPSFYLYKEDQLTGLKQAMSQEEIQNKVDFM--E
Sequence32-NP-GA-Jp-ABD        NNGSGLVIAGNEDGIKNPSFYLYKEDQLTGLKQALSQEEIQNKVDFM--E
Sequence116-NP-UD-Jp-ABC       KNGSGLVIAGGEKGINNPSFYLYKEDQLTGSQRALSQEEIQNKIDFM--E
Sequence8-NP-GCA-Jp-ABD        NNGSGLVIAGNENGIKNPSFYLYKEDQLTGLKQALSQGEIQNKVDFM--E
Sequence83-P-CG-EA-ABD         NNGSGLVIAGNEDGIKNPSFYLYKEDQLTGLKQALSQEEIQNKVDFM--E
Sequence48-NP-UD-OCC-ABC       KNGSGLVIAGGEKGVNNPSFYLYKEDQLTGLKQALSQEEIQNKVDFM--E
Sequence120-NP-G-Jp-ABC        KNGSGLVIAGGEKGINNPSFYLYKEDQLTGSQRALSQEEIRNKIDFM--E
Sequence187-NP-UG-Jp-ABCCC     KNGSGLVIAGGEKGINNPSFYLYKEDQLTGSQRALSQEEIRNKIDFM--E
Sequence118-NP-G-Jp-ABD        NNGSGLIIAGNEDGIKNPSFYLYKEDQLTGLKQAMSQEEIQNKVDFM--E
Sequence87-NP-UG-Vit-ABD       NNGSGLIIAGNEDGIKNPSFYLYKEDQLTGLKKALSQEEIQNKVDFM--E
```

```
                               451                                            500
Sequence140-NP-GA-Col-ABABC    FLAQNN-AKLDNLSEKEKEKFRNEIKDFQKDSKAYLDALGNDRIAFVSKK
Sequence123-NP-UD-Jp-ABD       FLAKNN-AKLDNLSEKEKEK-------FQKDRKAYLDALGNDHVTFVSKK
Sequence111-NP-G-Jp-ABD        FLVKNN-AKLDNLSEKEKEKFQTEIGKFQKDRKAYLDALGNDHVAFVSKK
Sequence57-NP-GA-OCC-AB        FLAQNN-AKLDNLSKKEKEKFQNEIEDFQKDSKAYLDALGNDHIAFVSKK
Sequence172-P-CG-Col-ABCC      FLAQNN-AKLDNLSEKEKEKFRNEIKDFQKDSKAYLDALGNDRIAFVSKK
Sequence110-NP-UD-Jp-ABD       FLAKNN-AKLDNLSKEEKEKFQTEIENFQKDRKAYLDALGNDHVAFVSKK
Sequence95-NP-UD-Vit-ABD       FLAKNN-AKLDNLSEKEKEKFQTEIENFQKDRKAYLDALGNDHVAFVSKK
Sequence107-P-CG-Vit-ABD       FLARNN-AKLDNLSEKEKEKFQTEIENFQKDRKAYLDALGNDHVAFVSKK
Sequence106-P-CG-Vit-ABD       ISCTKTIAKLDNLSEKEKEKFRTEIEDFQKDRKAYLDALGNDHVAFVSKK
Sequence105-NP-UD-Vit-ABD      FLAKNN-AKLDNLSEKEKEKFQTEIGNFQKDRKAYLDALGNDHIAFVSKK
Sequence126-NP-UG-Jp-ABC       FLAQNN-AKLDNLSEKEKEKFRTEIKDFQKDSKAYLDALGNDRIAFVSKK
Sequence135-P-CG-Col-ABCCC     FLAQNN-AKLDNLSEKEKEKFRTEIKDFQKDSKPYLDALGNDRIAFVSKK
Sequence138-NP-GA-Col-ABC      FLAQNN-AKLDNLSEKEKEKFRTEIKDFQKDSKPYLDALGNDRIAFVSKK
Sequence13-NP-UP-Jp-ABD        FLARNN-AKLDNLSKKEKEKFQTEIENFQKDRKAYLDALGNDHIAFVSKK
Sequence11-NP-UP-Jp-ABD        FLAQNN-AKLGNLSEKEKEKFQTEIENFQKDRKAYLDALGNDHIAFVSKK
Sequence50-NP-GA-OCC-ABC       FLAKNN-AKLDNLSEKEKEKFQTEIENFQKDRKAYLDALGNDHIAFVSKK
Sequence26-NP-GA-Jp-ABD        FLVQNN-AKLDNLSEKEKEKFQTEIEDFQKDRKAYLDALGNDHIAFVSKK
Sequence55-NP-UD-OCC-ABCCC     FLAQNN-AKLDNLSKKEKEKFQNEIEDFQKDSKAYLDALGNDHIAFVSKK
Sequence178-NP-M-Col-ABCC      FLAQNN-AKLDNLSEKEKEKFRTEIKDFQKDSKPYLDALGNDRIAFVSKK
Sequence40-NP-GA-Jp-ABD        FLAKNN-AKLDNLSEKEKEKFQTEIGNFQKDRKAYLDALGNDHIAFVSKK
Sequence43-NP-GA-Jp-ABD        FLVRNN-AKLDNLSKKEKEKFQTEIENFQKNPKAYLDALGNDHIAFVSKK
Sequence139-NP-UD-Col-ABCC     FLAQNN-AKLDNLSEKEKEKFRNEIKDFQKDSKAYLDALGNDRIAFVSKK
Sequence7-P-CG-Jp-ABBD         FLAQNN-AKLDNLSEKEKEKFQTEIENFQKDRKAYLDALGNDHIAFVSKK
Sequence91-P-CG-Vit-ABD        FLAQNN-AKLDNLSEKEKEKFQAEIENFPKDRKAYLDALGNDHIAFVSKK
Sequence29-NP-GA-Jp-ABABD      FLAKNN-AKLDNLSEKEKEKFQTEIENFQKDRKAYLDALGNDHIAFVSKK
Sequence78-P-CG-EA-ABD         FLAKNN-ARLDNLSEKEKEEFQTEIEDFQKNPKAYLDALGNDHIAFVSKK
Sequence188-NP-GCA-Jp-AB       FLAQNN-AKLDNLSEKEKEKFRTEIKDFQKDSKAYLDALGNDRIAFVSKK
Sequence72-P-CG-EA-ABD         FLAKNN-AKLDNLSEKEKEKFQTEIGNFQKDRKAYLDALGNDHIAFVSKK
Sequence79-P-CG-EA-ABD         FLAKNN-AKLDNLSEKEKEKFQTEIEDFQKNRKAYLDALGNDRVAFVSKK
Sequence61-NP-UD-OCC-ABC       FLAKNN-AKLDNLSEKEKEKFQTEIEDFQKNRKAYLDALGNDRVAFVSKK
Sequence125-NP-UG-Jp-ABCC      FLAQNN-AKLDNLSEKEKEKFRNEIKDFQKDSKPYLDALGNDRIAFVSKK
Sequence175-NP-G-Col-ABCC      FLAQNN-AKLDNLSEKEKEKFQNEIKDFQKDSKAYLDALGNDRIAFVSKK
Sequence122-NP-G-Jp-ABCC       FLAQNN-AKLDNLSEKEKEKFRNEIKDFQKDSKAYLDALGNDRIAFVSKK
Sequence36-NP-GA-Jp-ABD        FLAKNN-AKLDNLSEKEKEKFQTEIGNFQKDRKAYLDALGNDHVASVSKK
Sequence58-NP-UD-OCC-ABC       FLAQNN-AKLDNLSKKEKEKFQNEIEDFQKDSKAYLDALGNDHIAFVSKK
Sequence27-NP-GA-Jp-ABBD       FLAKNN-AKLDNLSEKEKEKFQTEIENFQKDRKAYLDALGNDHIAFVSKK
Sequence93-P-CG-Vit-ABD        FLARNN-AKLDNLSEKEKEKFQTEIEYFQKDRKAYLDALGNDHVAFVSKK
Sequence177-NP-M-Col-ABCC      FLAQNN-AKLDNLSEKEKEKFQNEIKDFQKDSKAYLDALGNDRIAFVSKK
Sequence182-NP-UD-Col-ABCC     FLAQNN-AKLDNLSEKEKEKFRNEIKDFQKDSKAYLDALGNDRIAFVSKK
Sequence114-NP-UG-Jp-ABC       FLAQNN-AKLDNLSEKEKEKFRTEIKDFQKDSKAYLDTLGNDRIAFVSKK
Sequence74-P-CG-EA-ABD         FLAQNN-AKLDNLSEKEKEKFQTEIEYFQKDRKAYLDALGNDHIAFVSKK
Sequence20-P-CG-Jp-ABD         FLAKNN-AKLDNLSEKEKEKFQTEIGNFQKDRKAYLDALGNDHIAFVSKK
Sequence14-NP-GCA-Jp-ABD       FLAQNN-AKLDNLSEKEKEKFQTEIEYFQKDRKAYLDALGNDHIAFVSKK
```

| | |
|---|---|
| Sequence39-NP-GA-Jp-ABC | FLAQNN-AKLDNLSEKEKEKFRTEIKDFQKDSKPYLDALGNDRIAFVSKK |
| Sequence170-NP-UD-Col-ABCC | FLAQNN-AKLDNLSEKEKEKFRNEIKDFQKDSKPYLDALGNDRIAFVSKK |
| Sequence76-P-CG-EA-ABD | FLAKNN-AKLDNLSEKEKEKFQTEIGNFQKDRKAYLDALGNDHIAFVSKK |
| Sequence53-NP-GA-EA-ABD | FLAKNN-AKLDNLSEKEKEKFQTEIGNFQKDRKAYLDALGNDHVAFVSKK |
| Sequence174-P-CG-Col-ABCC | FLAQNS-AKLDNLSEKEKEKFRNEIKDFQKDSKAYLDALGNDRIAFVSKK |
| Sequence33-NP-UG-Jp-ABD | FLAQNN-AKLDNLSEKEKEKFQTEIEYFQKDSKAYLDALGNDRIAFVSKK |
| Sequence60-NP-GA-EA-ABD | FLVKNN-AKLDNLSEKEKEKFQTEIENFQKDRKAYLDALGNDHIAFVSKK |
| Sequence38-NP-GA-Jp-ABD | FLARNN-AKLDNLSEKEKEKFQTEIEYFQKDRKAYLDALGNDHIAFVSKK |
| Sequence1-NP-EG-Jp-ABCCC | FLAQNN-AKLDNLSEKEKEKFQNEIEDFQKDSKAYLDALGNDRIAFVSKK |
| Sequence101-P-CG-Vit-ABD | FLAKNN-AKLDNLSEKEKEKFQTEIENFQKDRKAYLDALGNDHVAFVSKK |
| Sequence64-NP-UD-OCC-ABC | FLAKNN-ARLDNLSEKEKEKFQTEIEDFQKNPKAYLDALGNDHIAFVSKK |
| Sequence81-P-CG-EA-ABD | FLAQNN-AKLDNLSEKEKEKFQTEIENFQKDRKAYLDALGNDHIAFVSKK |
| Sequence150-NP-DA-Sen-BC | FLAQNN-AKLDDLSEKEKEKFRTEIKDFQKDSKAYLDALGNDRIAFVSKK |
| Sequence90-NP-UD-Vit-ABD | FLAQNN-AKLDNLSEKEKEKFQTEIEYFQKDRKAYLDALGNDRIAFVSKK |
| Sequence136-P-CG-Col-AACC | FLAQNN-AKLDNLSEKEKEKFRTEIKDFQKDSKPYLDALGNDRIAFVSKK |
| Sequence15-NP-UP-Jp-ABD | FLVQNN-AKLDNLSEKEKEKFQTEIKNFQKDRKAYLDALGNDHIAFVSKK |
| Sequence108-NP-UD-Vit-ABD | FLAKNN-AKLDNLSEKEKEKFQTEIENFQKDRKAYLDALGNDHVAFVSKK |
| Sequence104-P-CG-OCC-ABCC | FLAQNN-AKLENLSEKEKEKFRTEIKDFQKDSKAYLDALGNDRIAFVSKK |
| Sequence179-NP-UD-Col-ABCC | FLAQNN-AKLDNLSEKEKEKFRNEIENFQKNSKAYLDALGNDRIAFVSKK |
| Sequence37-NP-GA-Jp-ABCCC | FLAQNN-AKLDNLSKKEKEKFQNEIEDFQKDSKAYLDALGNDHIAFVSKK |
| Sequence89-NP-UD-Vit-ABD | FLARNN-AKLDNLSEKEKEKFQTEIENFQKDRKAYLDALGNDHIAFVSKK |
| Sequence92-P-CG-Vit-ABD | FLAKNN-AKLDNLSEKEKEKFQAEIENFQKDRKAYLDALGNDHIAFVSKK |
| Sequence49-NP-GA-Jp-ABC | FLAQNN-AKLDNLSKKEKEKFQNEIEDFQKDSKAYLDALGNDHIAFVSKK |
| Sequence181-P-CG-Col-ABCC | FLAQNN-AKLDNLSEKEKEKFRNEIKDFQKDSKPYLDALGNDRIAFVSKK |
| Sequence25-NP-GA-Jp-ABD | FLAKNN-AKLDNLSEKEKEKFQTEIENFQKDRKAYLDALGNDHIAFVSKK |
| Sequence4-P-CG-Jp-ABD | FLAQNN-AKLDNLSEKEKEKFQTEIENFQKDRKAYLDALGNDHIAFVSKK |
| Sequence121-NP-G-Jp-ABCC | FLAQNN-AKLDDLSEKEKEKFRNEIKDFQKDSKAYLDALGNDRIAFVSKK |
| 4dvy | FLAQNN-TKLDNLSEKEKEKFQNEIEDFQKDSKAYLDALGNDRIAFVSKK |
| Sequence186-NP-UP-Jp-ABD | FLAQNN-AKLDNLSEKEKEKFQAEIENFQKDRKAYLDALGNDHIAFVSKK |
| Sequence62-NP-UD-OCC-ABD | FLAKNN-ARLDNLSKKEKEKFQNEIEDFQKDSKAYLDALGNDHIAFVSKK |
| Sequence9-NP-GCA-Jp-ABD | FLAQNN-AKLDNLSEKEKEKFQTEIENFQKDRKAYLDALGNDHIAFVSKK |
| Sequence12-NP-UP-Jp-ABD | FLAKNN-AKLDNLSEKEKEKFQTEIENFQKDRKAYLDALGNDHIAFVSKK |
| Sequence35-NP-GA-Jp-ABABD | FLAKNN-AKLDNLSEKEKEKFQTEIENFQKDRKAYLDALGNDHIAFVSKK |
| Sequence54-NP-GA-OCC-ABC | FLAQNN-AKLDNLSKKEKEKFQNEIEDFQKDSKAYLDALGNDHIAFVSKK |
| Sequence109-P-CG-Jp-ABD | FLAKNN-AKLDNLSEEEKEKFQTEIGNFQKDRKAYLDALGNDHVAFVSKK |
| Sequence96-NP-UD-Vit-ABD | FLARNN-AKLDNLSEKEKEKFQTEIGNFQKDRKAYLDALGNDHVAFVSKK |
| Sequence80-P-CG-EA-ABD | FLAKNN-AKLDNLSEKEKEKFQTEIEDFQKNRKAYLDALGNDRVAFVSKK |
| Sequence82-P-CG-EA-ABD | FLAKNN-AKLDNLSEKEKEKFQTEIENFQKDRKAYLDALGNDHIAFVSKK |
| Sequence10-NP-GCA-Jp-ABD | FLAKNN-AKLDNLSEKEKEKFQTEIENFQKDRKAYLDALGNDHIAFVSKK |
| Sequence19-NP-UP-Jp-ABBD | FLVQNN-AKLDNLSEKEKEKFQTEIENFQKDRKAYLDALGNDHIAFVSKK |
| Sequence115-NP-UG-Jp-ABC | FLAQNN-AKLDNLSVKEKEKFKNEIKDFQKDSKPYLDALGNDRIAFVSKK |
| Sequence45-NP-GA-Jp-ABCC | FLAQNN-AKLDNLSEKEKEKFRTEIKDFQKDSKAYLDALGNDRIAFVSKK |
| Sequence119-NP-UG-Jp-ABC | FLAQNN-AKLDNLSEKEKEKFRTEIKDFQKDSKAYLDALGNDRIAFVSKK |
| Sequence70-NP-GA-Ch-ABD | FLAKNN-AKLDNLSEKEKETFQTEIENFQKDRKAYLDALGSDHIAFVSKK |
| Sequence52-NP-UD-EA-ABD | FLAQNN-AKLDNLSEKEKEKFQTEIEYFQKDRKAYLDALGNDHIAFVSKK |
| Sequence97-P-CG-Vit-ABD | FLAKNN-AKLDNLSEKEKEKFQTEIENFQKDRKAYLDALGNDHIAFVSKK |
| Sequence94-NP-UD-Vit-ABD | FLHETM-LSETTCARKRKKNSKLRLKISKKTVRLIWTPYGMITLLLFLKK |
| Sequence176-NP-GA-Col-ABCC | FLAQNN-AKLDNLSEKEKEKFRNEIKDFQKDSKAYLDALGNDRIAFVSKK |
| Sequence68-NP-D-Am-ABBC | FLAQNN-AKLDNLSEKEKEKFQTEIKDFQKDSKAYLDALGNDRIAFVSKK |
| Sequence47-NP-GA-Jp-ABD | FLAKNN-AKLDNLSEKEKEKFQTEIGNFQKDRKAYLDALGNDHIAFVSKK |
| Sequence56-NP-UD-OCC-ABC | FLAQNN-AKLDNLSKKEKEKFQNEIEDFQKDSKAYLDALGNDHIAFVSKK |
| Sequence31-NP-GA-Jp-ABD | FLAQNN-AKLDNLSEKEKEKFQNEIEDFQKNRKAYLDALGNDHIAFVSKK |
| Sequence46-NP-UD-Jp-ABD | FLAQNN-AKLDNLSKKEKEKFQNEIEDFQKDSKAYLDALGNDHIAFVSKK |
| Sequence102-NP-UG-Vit-ABD | FLAKNN-AKLDNLSEKEKEKFQTEIENFPKDRKAYLDALGNDHVAFVSKK |
| Sequence71-P-CG-EA-ABD | FLAKNN-AKLDNLSEKEKEKFQTEIGNFQKDRKAYLDALGNDHVAFVSKK |
| Sequence30-NP-GA-Jp-ABBD | FLAKNN-AKLDNLSEKEKEKFQTEIENFQKDRKAYLDALGNDHIAFVSKK |
| Sequence28-NP-GA-Jp-AABD | FLAQNN-AKLDNLSEKEKEKFQAEIGNFQKDRKAYLDALGNDHIAFVSKK |
| Sequence98-P-CG-Vit-AD | FLAKNN-AKLDNLSEKEKEKFQTEIENFQKDRKAYLDALGSDHIAFVSKK |
| Sequence99-P-CG-Vit-ABD | FLAQNN-AKLDNLSEKEKEKFQAEIENFQKDRKAYLDALGNDHVAFVSKK |
| Sequence24-NP-UD-USA-ABD | FLAQNN-SKLDNLSEKEKEKFQTEIKNFQKDRKPYLDALWNDHIALVSKK |
| Sequence124-NP-G-Jp-ABC | FLAQNN-AKLDNLSEKEKEKFRTEIKDFQKDSKAYLDALGNDRIAFVSKK |

```
Sequence77-P-CG-OCC-ABCC      F L A K N N - A R L D N L S E K E K E K F Q T E I E D F Q K N P K A Y L D A L G N D H I A F V S K K
Sequence113-NP-UG-Jp-ABC      F L A Q N N - A K L D N L S E K E K E K F R T E I K D F Q K D S K A Y L D T L G N D R I A F V S K K
Sequence88-NP-UD-Vit-ABD      F L A K N N - A K L D N L S E K E K E K F Q T E I E Y F Q K D R K A Y L D A L G N D H V A F V S K K
Sequence100-NP-UD-Vit-ABD     F L A Q N N - A K L D N L S E K E K E K F Q T E I E Y F Q K D R K A Y L D A L G N D H V A F V S K K
Sequence137-NP-G-Col-ABCC     F L A Q N N - A K L D N L S E K E K E K F R N E I K D F Q K D S K A Y L D A L G N D R I A F V S K K
Sequence117-NP-G-Jp-ABC       F L A Q N N - A K L D N S S E K E K E K F Q T E I G N F Q K D R K A Y L D A L G N D H I A F V S K K
Sequence171-NP-UD-Col-ABCC    F L A Q N N - A K L D N L S E K E K E K F R T E I K D F Q K D S K P Y L D A L G N D R I A F V S K K
Sequence180-P-CG-Col-ABC      F L A Q N N - A K L D N L S E K E K E K F R N E I K D F Q K D S K A Y L D A L G N D R I A F V S K K
Sequence112-P-CG-Jp-ABCC      F L A Q N N - A K L D N L S E K E K E K F R T E I K D F Q K D S K A Y L D A L G N D R I A F V S K K
Sequence127-NP-UD-Jp-ABC      F L A Q N N - A K L D N L S E K E K E K F R T E I K D F Q K D P K A Y L D A L G N D R I A F V S K K
Sequence51-NP-GA-OCC-ABC      F L A K N N - A R L D N L S E K E K E K F Q T E I E D F Q K N P K A Y L D A L G N D H I A F V S K K
Sequence73-P-CG-EA-ABD        F L A Q N N - A K L D N L S E K E K E K F Q T E I E N F Q K D R K A Y L D A L G N D H I A F V S K K
Sequence183-NP-UD-Col-ABD     F L A Q N N - A K L D N L S E K E K E K F R N E I K D F Q K D S K A Y L D A L G N D R I A F V S K K
Sequence34-NP-UD-Jp-ABDD      F L A R N N - A K L D N L S K K E K E K F Q T E I E N F Q K N P K A Y L D A L G N D R I A F V S K K
Sequence21-P-CG-Jp-ABD        F L A K N N - A K L D N L S E K E K E K F Q T E I G N F Q K D R K A Y L D A L G N D H I A F V S K K
Sequence32-NP-GA-Jp-ABD       F L A K N N - A K L D N L S E K E K E K F Q T E I G N F Q K D R K A Y L D A L G N D H I A F V S K K
Sequence116-NP-UD-Jp-ABC      F L A Q N N - A K L D N L S V K E K E K F K N E I K D F Q K D S K P Y L D A L G N D R I A F V S K K
Sequence8-NP-GCA-Jp-ABD       F L A Q N N - A K L D N L S E K E I R K F P S E I E N F Q K N P K A Y L D A L G N D H I V F V S K K
Sequence83-P-CG-EA-ABD        F L A K N N - A K L D N L S E K E K E K F Q T K I E N F Q K D R K A Y L D A L G N D H I A F V S K K
Sequence48-NP-UD-OCC-ABC      F L A Q N N - A K L D N L S K K E K E K F Q N E I E D F Q K D S K A Y L D A L G N D H I A F V S K K
Sequence120-NP-G-Jp-ABC       F L A Q N N - A K L D N L S E K E K E K F R N E I K D F Q K D S K A Y L D A L G N D R I A F V S K K
Sequence187-NP-UG-Jp-ABCCC    F L A Q N N - A K L D N L S E K E K E K F Q N E I E D F Q K D S K A Y L D A L G N D R I A F V S K K
Sequence118-NP-G-Jp-ABD       F L A Q N N - A K L D N L S E K E K E K F Q T E I K N F Q K D P K A Y L D A L G N D H I A F V S K K
Sequence87-NP-UG-Vit-ABD      F L A Q N N - A E L D N L S E K E K E K F Q T E I E N F Q K D R K A Y L D A L G N D H I A F V S K K

                              501                                                                                            550
Sequence140-NP-GA-Col-ABABC   D T K - - - - H S A L I T E F G K G D L S Y T L K D Y G K K A D K A L D R E K N V T L Q - - G N L K
Sequence123-NP-UD-Jp-ABD      D P K - - - - H L A L V T E F G N G E L S Y T L K D Y G K K Q D K A L D G E T K T T L Q - - G N L K
Sequence111-NP-G-Jp-ABD       D P K - - - - H L A L V T E F G N G E L S Y T L K D Y G K K Q D K A L D G E T K T T L Q - - G S L E
Sequence57-NP-GA-OCC-AB       D K K - - - - H L A L V A E F G N G E L S Y T L K D Y G K K Q D K A L D R E T K T T L Q - - G N L K
Sequence172-P-CG-Col-ABCC     D P K - - - - H S A L I T E F G K G D L S Y T L K D Y G K K A D K A L D R E K N V T L Q - - G N L K
Sequence110-NP-UD-Jp-ABD      D P K - - - - H L A L V T E F G N G E V S Y T L K D Y G K K Q D K A L D G E T K T T L Q - - G S L K
Sequence95-NP-UD-Vit-ABD      D P K - - - - H L A L V T E F G N G E V S Y T L K D Y G K K Q D K A L D G E V K T T L Q - - G S L K
Sequence107-P-CG-Vit-ABD      D P K - - - - H L A L V T E F G N G E V S Y T L K D Y G K K Q D K A L D G E V K T T L Q - - G S L K
Sequence106-P-CG-Vit-ABD      D T K - - - - H S A L V T E F G N G E V S Y T L K D Y G K K A D K A L D R E K N A T L Q - - G S L K
Sequence105-NP-UD-Vit-ABD     D P K - - - - H L A L V T E F G N G E V S Y T L K D Y G K K Q D K A F D G E T K T T L Q - - G S L K
Sequence126-NP-UG-Jp-ABC      D P K - - - - H S A L I T E F G K G D L S Y T L K D Y G K K A D K A L D R E K N V T L Q - - G N L K
Sequence135-P-CG-Col-ABCCC    D P K - - - - H S A L I T E F G R G D L S Y T L K D Y G K K A D K A L D R E K N V T L Q - - G N L K
Sequence138-NP-GA-Col-ABC     D T K - - - - H S A L I T E F G K G D L S Y T L K D Y G K K A D K A L D R E K N L T L Q - - G S L K
Sequence13-NP-UP-Jp-ABD       D P K - - - - H L A L V T E F G N G E V S Y T L K D Y G K K Q D K A L D G E T K T T L Q - - G S L K
Sequence11-NP-UP-Jp-ABD       D P K - - - - H L A L V T E F G N G E V S Y T L K D Y G K K Q D K A L D G E I K T T L Q - - G S L K
Sequence50-NP-GA-OCC-ABC      D K K - - - - H L A L V A E F G N G E L S Y T L K D Y G K K Q D K A L D R E T K T T L Q - - G N L K
Sequence26-NP-GA-Jp-ABD       D P K - - - - H L A L V T E F G N G E V S Y T L K D Y G K K Q D K A L D G E V K T T L Q - - G S L K
Sequence55-NP-UD-OCC-ABCCC    D K K - - - - H L A L V A E F G N G E L S Y T L K D Y G K K Q D K A L D R E T K T T L Q - - G N L K
Sequence178-NP-M-Col-ABCC     D P K - - - - H S A L I T E F G K G D L S Y T L K D Y G K K A D K A L D R E K N V T L Q - - G N L K
Sequence40-NP-GA-Jp-ABD       D P K - - - - H L A L V T E F G N G E L S Y T L K D Y G K K Q D K A L D G E T K T T L Q - - G S L K
Sequence43-NP-GA-Jp-ABD       D P K - - - - H L A L V T E F G N G E V S Y T L K D Y G K K Q D K A L D G E T K T T L Q - - G S L K
Sequence139-NP-UD-Col-ABCC    D P K - - - - H L A L I T E F G K G D L S Y T L K D Y G K K A D R A L D R E K N V T L Q - - G S L K
Sequence7-P-CG-Jp-ABBD        D P K - - - - H L A L V T E F G N G E V S Y T L K D Y G K K Q D K A L D G E T K T T L Q - - G S L K
Sequence91-P-CG-Vit-ABD       D P N I Q L W L L S L V - - M G N G A I L S K I - - M G K K Q D K A L D G E T K T T L Q - - G N L N
Sequence29-NP-GA-Jp-ABABD     D P K - - - - H L A L V T E F G N G E V S Y T L K D Y G K K Q D K A L D G E T K T T L Q - - G S L K
Sequence78-P-CG-EA-ABD        D K K - - - - H L A L V T E F G N G E V S Y T L K D Y G K K Q D K A L D G E T K T T L Q - - G S L K
Sequence188-NP-GCA-Jp-AB      D T K - - - - H S A L I T E F G N G D L S Y T L K D Y G K K A D K A L D R E K N V T L Q - - G S L K
Sequence72-P-CG-EA-ABD        D P K - - - - H L A L V T E F G N G E V S Y T L K D Y G K K Q D K A L D G E T K T T L Q - - G S L K
Sequence79-P-CG-EA-ABD        D P K - - - - H L A L V T E F G N G E V S Y T L K D Y G K K Q D K A L D G E T K T T L Q - - G S L K
Sequence61-NP-UD-OCC-ABC      D P K - - - - H L A L V T E F G N G E V S Y T L K D Y G K K Q D K A L D R E T K T T L Q - - G N L K
Sequence125-NP-UG-Jp-ABCC     D P K - - - - H S A L T T E F N K G D L S Y T L K D Y G K K A D K A L D R E K N V T L Q - - G S L K
Sequence175-NP-G-Col-ABCC     D P K - - - - H S A L I T E F N K G D L S Y T L K D Y G K K A D K A L D R E K N V T L Q - - G N L K
Sequence122-NP-G-Jp-ABCC      D P K - - - - H S A L I T E F G K G D L S Y T L K D Y G K K A D K A L D R E K N V T L Q - - G N L K
Sequence36-NP-GA-Jp-ABD       D P K - - - - H L A L V T E F G N G E V S Y T L K D Y G K K Q D K A L D G E T K T T L Q - - G S L K
Sequence58-NP-UD-OCC-ABC      D K K - - - - H L A L V A E F G N G E L S Y T L K D Y G K K Q D K A L D R E T K T T L Q - - G N L K
```

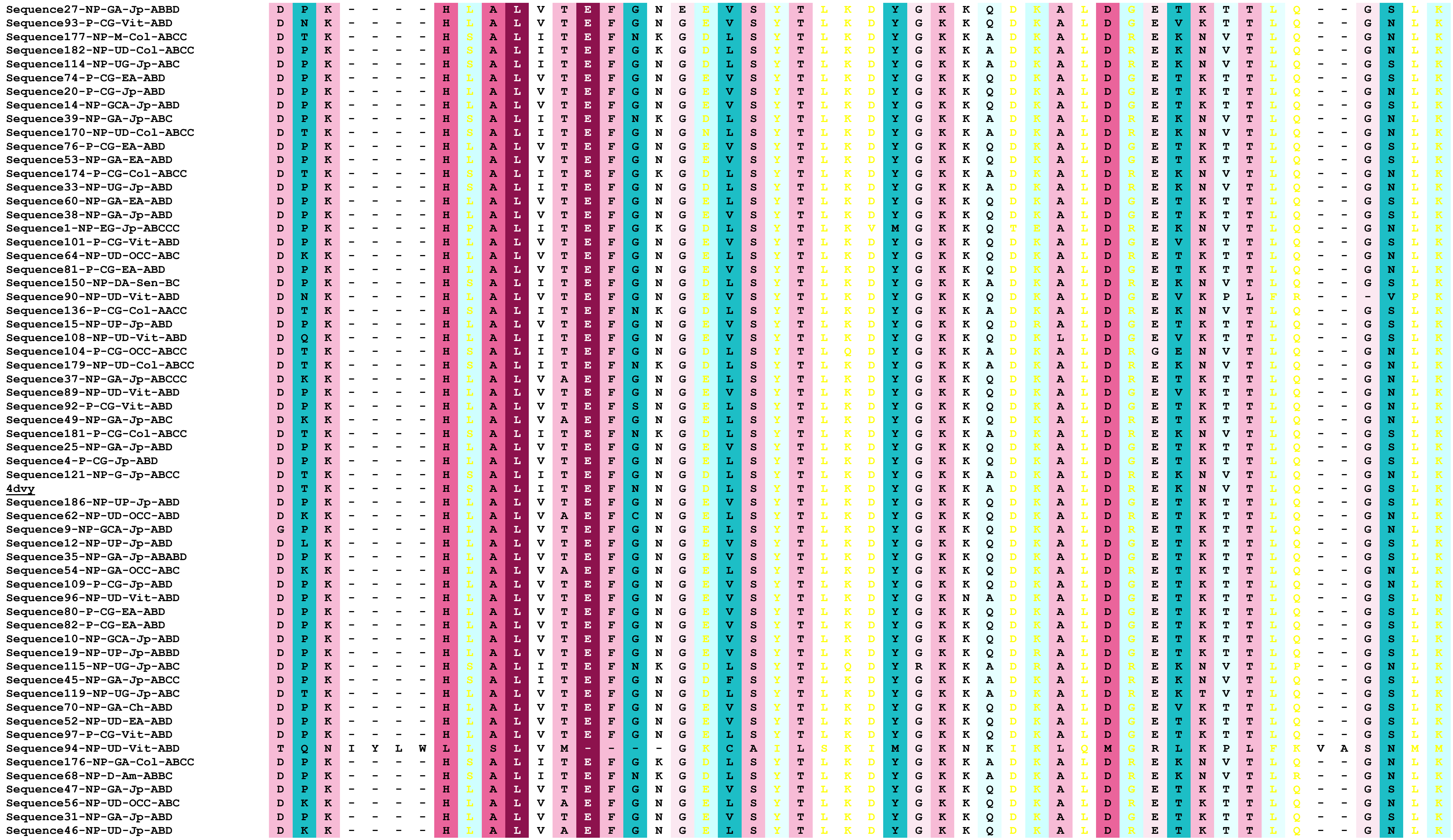
Sequence27-NP-GA-Jp-ABBD D P K - - - - H L A L V T E F G N E E V S Y T L K D Y G K K Q D K A L D G E T K T T L Q - - G S L K
Sequence93-P-CG-Vit-ABD D N K - - - - H L A L V T E F G N G E V S Y T L K D Y G K K Q D K A L D G E V K T T L Q - - G N L K
Sequence177-NP-M-Col-ABCC D T K - - - - H S A L I T E F N K G D L S Y T L K D Y G K K A D K A L D R E K N V T L Q - - G N L K
Sequence182-NP-UD-Col-ABCC D P K - - - - H S A L I T E F G K G D L S Y T L K D Y G K K A D K A L D R E K N V T L Q - - G N L K
Sequence114-NP-UG-Jp-ABC D P K - - - - H S A L I T E F G N G D L S Y T L K D Y G K K A D K A L D R E K N V T L Q - - G S L K
Sequence74-P-CG-EA-ABD D P K - - - - H L A L V T E F G N G E V S Y T L K D Y G K K Q D K A L D G E T K T T L Q - - G S L K
Sequence20-P-CG-Jp-ABD D P K - - - - H L A L V T E F G N G E V S Y T L K D Y G K K Q D K A L D G E T K T T L Q - - G N L K
Sequence14-NP-GCA-Jp-ABD D P K - - - - H L A L V T E F G N G E V S Y T L K D Y G K K Q D K A L D G E T K T T L Q - - G S L K
Sequence39-NP-GA-Jp-ABC D P K - - - - H S A L I T E F N K G D L S Y T L K D Y G K K A D K A L D R E K N V T L Q - - G N L K
Sequence170-NP-UD-Col-ABCC D T K - - - - H S A L I T E F G N G N L S Y T L K D Y G K K A D K A L D R E K N V T L Q - - G N L K
Sequence76-P-CG-EA-ABD D P K - - - - H L A L V T E F G N G E V S Y T L K D Y G K K Q D K A L D G E T K T T L Q - - G S L K
Sequence53-NP-GA-EA-ABD D P K - - - - H L A L V T E F G N G E V S Y T L K D Y G K K Q D K A L D G E T K T T L Q - - G S L K
Sequence174-P-CG-Col-ABCC D T K - - - - H S A L I T E F G K G D L S Y T L K D Y G K K A D K A L D R E K N V T L Q - - G N L K
Sequence33-NP-UG-Jp-ABD D P K - - - - H S A L I T E F G N G D L S Y T L K D Y G K K A D K A L D R E K N V T L Q - - G N L K
Sequence60-NP-GA-EA-ABD D P K - - - - H L A L V T E F G N G E L S Y T L K D Y G K K Q D K A L D G E T K T T L Q - - G S L K
Sequence38-NP-GA-Jp-ABD D P K - - - - H L A L V T E F G N G E V S Y T L K D Y G K K Q D K A L D G E T K T T L Q - - G S L K
Sequence1-NP-EG-Jp-ABCCC D P K - - - - H P A L I T E F G K G D L S Y T L K V M G K K Q T E A L D R E K N V T L Q - - G N L K
Sequence101-P-CG-Vit-ABD D P K - - - - H L A L V T E F G N G E V S Y T L K D Y G K K Q D K A L D G E V K T T L Q - - G S L K
Sequence64-NP-UD-OCC-ABC D K K - - - - H L A L V T E F G N G E L S Y T L K D Y G K K Q D K A L D R E T K T T L Q - - G N L K
Sequence81-P-CG-EA-ABD D P K - - - - H L A L V T E F G N G E V S Y T L K D Y G K K Q D K A L D G E T K T T L Q - - G N L K
Sequence150-NP-DA-Sen-BC D P K - - - - H S A L I T E F G N G D L S Y T L K D Y G K K A D K A L D R E K N V T L Q - - G S L K
Sequence90-NP-UD-Vit-ABD D N K - - - - H L A L V T E F G N G E V S Y T L K D Y G K K Q D K A L D G E V K P L F R - - - V P K
Sequence136-P-CG-Col-AACC D T K - - - - H S A L I T E F N K G D L S Y T L K D Y G K K A D K A L D R E K N V T L Q - - G S L K
Sequence15-NP-UP-Jp-ABD D P K - - - - H L A L V T E F G N G E V S Y T L K D Y G K K Q D R A L D G E T K T T L Q - - G S L K
Sequence108-NP-UD-Vit-ABD D Q K - - - - H L A L I T E F G N G E V S Y T L K D Y G K K Q D K L L D G E V K T T L Q - - G S L K
Sequence104-P-CG-OCC-ABCC D T K - - - - H S A L I T E F G N G D L S Y T L Q D Y G K K A D K A L D R G E N V T L Q - - G N L K
Sequence179-NP-UD-Col-ABCC D T K - - - - H S A L I T E F N K G D L S Y T L K D Y G K K A D K A L D R E K N V T L Q - - G N L K
Sequence37-NP-GA-Jp-ABCCC D K K - - - - H L A L V A E F G N G E L S Y T L K D Y G K K Q D K A L D R E T K T T L Q - - G N L K
Sequence89-NP-UD-Vit-ABD D P K - - - - H L A L V T E F G N G E V S Y T L K D Y G K K Q D K A L D G E V K T T L Q - - G N L K
Sequence92-P-CG-Vit-ABD D P K - - - - H L A L V T E F S N G E L S Y T L K D Y G K K Q D K A L D G E T K T T L Q - - G N L K
Sequence49-NP-GA-Jp-ABC D K K - - - - H L A L V A E F G N G E L S Y T L K D Y G K K Q D K A L D R E T K T T L Q - - G N L K
Sequence181-P-CG-Col-ABCC D T K - - - - H S A L I T E F N K G D L S Y T L K D Y G K K A D K A L D R E K N V T L Q - - G S L K
Sequence25-NP-GA-Jp-ABD D P K - - - - H L A L V T E F G N G E V S Y T L K D Y G K K Q D K A L D G E T K T T L Q - - G S L K
Sequence4-P-CG-Jp-ABD D P K - - - - H L A L V T E F G N G E L S Y T L K D Y G K K Q D K A L D G E T K T T L Q - - G S L K
Sequence121-NP-G-Jp-ABCC D T K - - - - H S A L I T E F G N G D L S Y T L K D Y G K K A D K A L D R E K N V T L Q - - G N L K
4dvy D T K - - - - H S A L I T E F N N G D L S Y T L K D Y G K K A D K A L D R E K N V T L Q - - G S L K
Sequence186-NP-UP-Jp-ABD D P K - - - - H L A L V T E F G N G E V S Y T L K D Y G K K Q D K A L D G E T K T T L Q - - G S L K
Sequence62-NP-UD-OCC-ABD D K K - - - - H L A L V A E F C N G E L S Y T L K D Y G K K Q D K A L D R E T K T T L Q - - G N L K
Sequence9-NP-GCA-Jp-ABD G P K - - - - H L A L V T E F G N G E L S Y T L K D Y G K K Q D K A L D R E T K T T L Q - - G N L K
Sequence12-NP-UP-Jp-ABD D L K - - - - H L A L V T E F G N G E V S Y T L K D Y G K K Q D K A L D G E T K T T L Q - - G S L K
Sequence35-NP-GA-Jp-ABABD D P K - - - - H L A L V T E F G N G E V S Y T L K D Y G K K Q D K A L D G E T K T T L Q - - G S L K
Sequence54-NP-GA-OCC-ABC D K K - - - - H L A L V A E F G N G E L S Y T L K D Y G K K Q D K A L D R E T K T T L Q - - G N L K
Sequence109-P-CG-Jp-ABD D P K - - - - H L A L V T E F G N G E V S Y T L K D Y G K K Q D K A L D G E T K T T L Q - - G N L K
Sequence96-NP-UD-Vit-ABD D P K - - - - H L A L V T E F G N G E V S Y T L K D Y G K N A D K A L D G E T K T T L Q - - G S L N
Sequence80-P-CG-EA-ABD D P K - - - - H L A L V T E F G N G E V S Y T L K D Y G K K Q D K A L D G E T K T T L Q - - G S L K
Sequence82-P-CG-EA-ABD D P K - - - - H L A L V T E F G N G E V S Y T L K D Y G K K Q D K A L D G E T K T T L Q - - G S L K
Sequence10-NP-GCA-Jp-ABD D P K - - - - H L A L V T E F G N G E V S Y T L K D Y G K K Q D K A L D G E T K T T L Q - - G S L K
Sequence19-NP-UP-Jp-ABBD D P K - - - - H L A L V T E F G N G E V S Y T L K D Y G K K Q D R A L D G E T K T T L Q - - G S L K
Sequence115-NP-UG-Jp-ABC D P K - - - - H S A L I T E F N K G D L S Y T L Q D Y R K K A D R A L D R E K N V T L P - - G N L K
Sequence45-NP-GA-Jp-ABCC D P K - - - - H S A L I T E F G N G D F S Y T L K D Y G K K A D K A L D R E K N V T L Q - - G S L K
Sequence119-NP-UG-Jp-ABC D T K - - - - H L A L V T E F G N G D L S Y T L K D Y G K K A D K A L D R E K T V T L Q - - G S L K
Sequence70-NP-GA-Ch-ABD D P K - - - - H L A L V T E F G N G E V S Y T L K D Y G K K Q D K A L D G E V K T T L Q - - G S L K
Sequence52-NP-UD-EA-ABD D P K - - - - H L A L V T E F G N G E V S Y T L K D Y G K K Q D K A L D G E T K T T L Q - - G S L K
Sequence97-P-CG-Vit-ABD D P K - - - - H L A L V T E F G N G E L S Y T L K D Y G K K Q D K A L D G E T K T T L Q - - G S L K
Sequence94-NP-UD-Vit-ABD T Q N I Y L W L L S L V M - - - - G K C A I L S K I M G K N K I K L Q M G R L K P L F K V A S N M M
Sequence176-NP-GA-Col-ABCC D P K - - - - H S A L I T E F G K G D L S Y T L K D Y G K K A D K A L D R E K N V T L Q - - G N L K
Sequence68-NP-D-Am-ABBC D P K - - - - H S A L I T E F N K G D L S Y T L K D Y G K K A D K A L D R E K N V T L R - - G N L K
Sequence47-NP-GA-Jp-ABD D P K - - - - H L A L V T E F G N G E V S Y T L K D Y G K K Q D K A L D G E T K T T L Q - - G S L K
Sequence56-NP-UD-OCC-ABC D K K - - - - H L A L V A E F G N G E L S Y T L K D Y G K K Q D K A L D R E T K T T L Q - - G N L K
Sequence31-NP-GA-Jp-ABD D P K - - - - H L A L V T E F G N G E V S Y T L K D Y G K K Q D K A L D G E T K T T L Q - - G S L K
Sequence46-NP-UD-Jp-ABD D K K - - - - H L A L V A E F G N G E L S Y T L K D Y G K K Q D K A L D R E T K T T L Q - - G N L K

```
Sequence102-NP-UG-Vit-ABD      DPK----HLALVTEFGNGEVSYTLKDYGKKQDKALDGEVKTTLQ--GSLK
Sequence71-P-CG-EA-ABD         DPK----HLALVTEFGNGEVSYTLKDYGKKQDKALDGETKTTLQ--GSLK
Sequence30-NP-GA-Jp-ABBD       DPK----HLALVTEFSNGEVSYTLKDYGKKQDKALDGETKTTFQ--GSLK
Sequence28-NP-GA-Jp-AABD       DPK----HLALVTEFGNGEVSYTLKDYGKKQDKALDGETKTTLQ--GSLK
Sequence98-P-CG-Vit-AD         DNK----HLALVTEFGNGEVSYTLKIMGKNKIKLSDGGGKNHSS--RLPK
Sequence99-P-CG-Vit-ABD        DNK----HLALVTEFGNGEVSYTLKDYGKKQDKALDGEVKTTLQ--GNLK
Sequence24-NP-UD-USA-ABD       DPK----HLALVTEFGNGEVSYTLQDYGKNKIKPLDGEVKTTLQ--GSLK
Sequence124-NP-G-Jp-ABC        DPK----HSALITEFGKGDLSYTLKDYGKKADKALDREKNVTLQ--GNLK
Sequence77-P-CG-OCC-ABCC       DKK----HLALVTEFGNGELSYTLKDYGKKQDKALDRETKTTLQ--GNLK
Sequence113-NP-UG-Jp-ABC       DPK----HSALITEFGNGDLSYTLKDYGKKADKALDREKNVTLQ--GSLK
Sequence88-NP-UD-Vit-ABD       DQK----HLALVTEFGNGGSELHSQRLWEKQDKALDGEVKTTLQ--GSLK
Sequence100-NP-UD-Vit-ABD      DPK----HLALVTEFGNGEVSYTLKDYGKKQDKALDGEVKTTLQ--GNLE
Sequence137-NP-G-Col-ABCC      DPK----HSALITEFGKGDLSYTLKDYGKKADKALDREKNVTLQ--GNLK
Sequence117-NP-G-Jp-ABC        DPK----HLALVTEFGNGEVSYTLKDYGKKADKALDREKNVTLQ--GSLK
Sequence171-NP-UD-Col-ABCC     DTK----HSALITEFGKGDLSYTLKDYGKKADKALDREKNLTLQ--GNLK
Sequence180-P-CG-Col-ABC       DTK----HSALITEFGKGDLSYTLKDYGKKADKALDREKNVTLQ--GNLK
Sequence112-P-CG-Jp-ABCC       DTK----HSALITEFGNGDLSYTLKDYGKKADKALDREKNVTLQ--GSLK
Sequence127-NP-UD-Jp-ABC       DTK----HLALITEFGNGDLSYTLKDYGKKADKALDGEKNVTLQ--GSLK
Sequence51-NP-GA-OCC-ABC       DKK----HLALVTEFGNGELSYTLKDYGKKQDKALDRETKTTLQ--GNLK
Sequence73-P-CG-EA-ABD         DPK----HLALVTEFGNGEVSYTLKDYGKKQDKALDGETKTTLQ--GSLK
Sequence183-NP-UD-Col-ABD      DPK----HSALITEFGKGDLSYTLKDYGKKADKALDREKNVTLQ--GNLK
Sequence34-NP-UD-Jp-ABDD       DPK----HLALVTEFGNGEVSYTLKDYGKKQDKALDGETKTTLQ--GNLK
Sequence21-P-CG-Jp-ABD         DPK----HLALVTEFGNREVSYTLKDYGKKQDKALDGETKTTLQ--GSLK
Sequence32-NP-GA-Jp-ABD        DPK----HLALVTEFGNGEVSYTLKDYGKKQDKALDGETKTTLQ--GSLK
Sequence116-NP-UD-Jp-ABC       DPK----HSALITEFNKGDLSYTLKDYRKKADRALDREKNVTLQ--GNLK
Sequence8-NP-GCA-Jp-ABD        DPK----HLALVTEFGNGEVSYTLKDYGKKQDKALDGETKTTLQ--GSLK
Sequence83-P-CG-EA-ABD         DKK----HLALVTEFGNGEVSYTLKDYGKKQDKALDGETKTTLQ--GSLK
Sequence48-NP-UD-OCC-ABC       DKK----HLALVAEFGNGELSYTLKDYGKKQDKALDRETKTTLQ--GNLK
Sequence120-NP-G-Jp-ABC        DPK----HSALITEFNKGDLSYTLKDYGKKADKALDREKNVTLQ--GNLK
Sequence187-NP-UG-Jp-ABCCC     DPK----HSALITEFGKGDLSYTLKDYGKKADRALDREKNVTLQ--GNLK
Sequence118-NP-G-Jp-ABD        DPK----HLALVTEFGNGEVSYTLKDYGTSQDKALDGETKTTLQ--GSLK
Sequence87-NP-UG-Vit-ABD       DPK----HLALVTEFGNGEVSYTLQDYGKKQDKALDGEVKTTLQ--GSLN

                               551                                             600
Sequence140-NP-GA-Col-ABABC    HDGV-MFVDYSNFKYT-NASKNPNKGVGVTNG-VSHLDAG-FNKVAVF-N
Sequence123-NP-UD-Jp-ABD       YDGV-MFVNYSNFKYT-NASQSPDKGVGATNG-VSHLEAN-FSKVAVF-N
Sequence111-NP-G-Jp-ABD        YDGV-MFVNYSNFKYT-NASKSPDKGVGTTNG-VSHLEAN-FSKVAVF-N
Sequence57-NP-GA-OCC-AB        HDGV-MFVNYSNFKYT-NASKSPDKGVGATNG-VSHLEAN-LSKVAVF-N
Sequence172-P-CG-Col-ABCC      HDGV-MFVDYSNFKYT-NASKNPNKGVGVTNG-VSHLDAG-FSKVAVF-N
Sequence110-NP-UD-Jp-ABD       YDGV-MFVDYSNFKYT-NASKSPDKGVGATNG-VSHLEAN-FSKVAVF-N
Sequence95-NP-UD-Vit-ABD       YDGM-MFVDYSNFKYT-NASQSPDKGVGTTNG-VSHLEAN-FSKVAVF-N
Sequence107-P-CG-Vit-ABD       YDGM-MFVDYSNFKYT-NASKSPDKGVGATNG-VSHLEAN-FSKVAVF-N
Sequence106-P-CG-Vit-ABD       YDGV-MFVNYSNFKYT-NVSKSPDKGVGATNG-VSHLEAG-FDKVAVF-D
Sequence105-NP-UD-Vit-ABD      YDGV-MFVDHSNSNTP-TPPRVLITAWVLRMA-FPIWKRI-LARELSL-I
Sequence126-NP-UG-Jp-ABC       HDGV-MFVNYSNFKYT-NASKSPNKGVGVTNG-VSHLEAG-FSKVAVF-N
Sequence135-P-CG-Col-ABCCC     HDGV-MFVDYSNFKYT-NASKNPNKGVGVTNG-VSHLEAG-FNKVAVF-N
Sequence138-NP-GA-Col-ABC      HDGV-MFVDYSNFKYT-NASKNPNKGVGATNG-VSHLDAG-FSKVAVF-N
Sequence13-NP-UP-Jp-ABD        YDGV-MFVNYSNFKYT-NASKSPDKGVGATNG-VSHLEAN-FSKVAVF-N
Sequence11-NP-UP-Jp-ABD        YDGV-MFVNYSNFKYT-NASKSPNKGVGATNG-VSHLEAN-LSKVAVF-N
Sequence50-NP-GA-OCC-ABC       HDGV-MFVNYSNFKYT-NASKSPDKGVGATNG-VSHLEAN-LSKVAVF-N
Sequence26-NP-GA-Jp-ABD        YDGM-MFVDYSNFKYT-NASKSPNKGVGATNG-VSHLEAN-FSKVAVF-N
Sequence55-NP-UD-OCC-ABCCC     HDGV-MFVNYSNFKYT-NASKSPDKGVGATNG-VSHLEAN-LSKVAVF-N
Sequence178-NP-M-Col-ABCC      HDGV-MFVDYSNFKYT-NASKNPNKGVGATNG-VSHLDAG-FSKVAVF-N
Sequence40-NP-GA-Jp-ABD        YDGV-MFVNYSNFKYT-NASKSPNKGLGATNG-VSHLEAN-FSKVAVF-N
Sequence43-NP-GA-Jp-ABD        YDGV-MFVNYSNFKYT-NASKSPDKGVGTTNG-VSRLEAN-FSKVAVS-N
Sequence139-NP-UD-Col-ABCC     HDGV-MFVDYSNFKYT-NASKNPNKGVGATNG-VSHLDAG-FNKVAVF-N
Sequence7-P-CG-Jp-ABBD         YDGV-MFVNYSNFKYT-NASKSPDKGVGATNG-VSHLEAN-FSKVAVF-N
Sequence91-P-CG-Vit-ABD        MMAW-MFVHYSNFKYT-TPPRVLIRGLGATNG-VSHLEAN-FSKVAVFIC
Sequence29-NP-GA-Jp-ABABD      YDGV-MFVDYSNFKYT-NASKSPDKGVGATNG-VSHLEAN-FSKVAVF-N
Sequence78-P-CG-EA-ABD         YDGV-MFVNYSNFKYT-NASKSPDKGVGTTNG-VSHLEAN-FSKVAVF-N
Sequence188-NP-GCA-Jp-AB       HDGV-MFVDYSNFKYT-NASKNPNKGVGVTNG-VSHLEVG-FNKVAIF-N
```

```
Sequence72-P-CG-EA-ABD        YDGV-MFVNYSNFKYT-DASKSPNKGLGATNG-VSHLEAN-FSKVAVF-N
Sequence79-P-CG-EA-ABD        YDGV-MFVNYSNFKYT-NASKSPNKGLGTTNG-VSHLEAN-FSKVAVF-N
Sequence61-NP-UD-OCC-ABC      HDGV-MFVNYSNFKYT-NVSKSPNKGLGTTNG-VSHLEAN-FSKVAVF-N
Sequence125-NP-UG-Jp-ABCC     HDGV-MFVDYSNFKYT-NASKNPNKGVGTTNG-VSHLDAG-FSKVAVF-N
Sequence175-NP-G-Col-ABCC     HDGV-MFVDYSNFKYT-NASKNPNKGVGVTNG-VSHLEAG-FNKVAVF-N
Sequence122-NP-G-Jp-ABCC      HDGV-MFVDYSNFKYT-NASKSPNKGVGTTNG-VSHLDAG-FSKVAVF-N
Sequence36-NP-GA-Jp-ABD       YDGV-MFVNYSNFKYT-NASKSPNKGLGATNG-VSHLEAN-FSKVAVF-N
Sequence58-NP-UD-OCC-ABC      HDGV-MFVNYSNFKYT-NASKSPDKGVGATNG-VSHLEAN-LSKVAVF-N
Sequence27-NP-GA-Jp-ABBD      YDGV-MFVDYSNFKYT-NASKSPNKGLGATNG-VSHLEAN-FSKVAVF-N
Sequence93-P-CG-Vit-ABD       YDGV-MFVNYSNFKYT-NASKSPDKGVSATNG-VSHLEAN-FSKVAVF-N
Sequence177-NP-M-Col-ABCC     HDGV-MFVDYSNFKYT-NASKNPNKGVGATNG-VSHLDAG-FNKVAVF-N
Sequence182-NP-UD-Col-ABCC    HDGV-MFVDYSNFKYT-NASKNPNKGVGVTNG-VSHLDAG-FSKVAVF-N
Sequence114-NP-UG-Jp-ABC      HDGV-MFVDYSNFKYT-NASKSPNKGVGVTNG-VSHLEAG-FNKVAVF-N
Sequence74-P-CG-EA-ABD        YDGV-MFVNYSNFKYT-NASKSPNKGLGTTNG-VSHLEAN-FSKVAVF-N
Sequence20-P-CG-Jp-ABD        YDGV-MFVNYSNFKYT-NASKSPDKGVGATNG-VSHLEAN-FSKVAVF-N
Sequence14-NP-GCA-Jp-ABD      YDGV-MFVNYSNFKYT-NASKSPDKGVGATNG-VSHLEAN-FSKVAVF-N
Sequence39-NP-GA-Jp-ABC       HDGV-MFVNYSNFKYT-NASKSPNKGVGVTNG-VSHLEAG-FSKVAVF-N
Sequence170-NP-UD-Col-ABCC    HDGV-MFVDYSNFKYT-NASKNPNKGVGATNG-VSHLEAG-FNKVAVF-N
Sequence76-P-CG-EA-ABD        YDGV-MFVNYSNFKYT-NASKSPNKGLGATNG-VSHLEAN-FSKVAVF-N
Sequence53-NP-GA-EA-ABD       YDGV-MFVNYSNFKYT-NASKSPNKGLGATNG-VSHLEAN-FSKVAVF-N
Sequence174-P-CG-Col-ABCC     HDGV-MFVDYSNFKYT-NASKNPNKGVGVTNG-VSHLDAG-FSKVAVF-N
Sequence33-NP-UG-Jp-ABD       YDGV-MFVDYSNFKYT-NASKNPNKGVGATNG-VSHLDAG-FNKVAVF-N
Sequence60-NP-GA-EA-ABD       YDGV-MFVNYSNFKYT-NASKSPNKGLGATNG-VSHLEAN-FSKVAVF-N
Sequence38-NP-GA-Jp-ABD       YDGV-MFVNYSNFKYT-NASKSPNKGVGATNG-VSHLEAN-FSKVAVF-N
Sequence1-NP-EG-Jp-ABCCC      HDSV-MFVNYSNFKYT-NASKSPDKGVGVTNG-VSHLDAG-FSKVAVF-N
Sequence101-P-CG-Vit-ABD      YDGV-MFVDYSNFKYT-NASKSPDKGVSATNG-VSHLEAN-FSKVAVF-N
Sequence64-NP-UD-OCC-ABC      HDGV-MFVNYSNFKYT-NASKSPDKGVGATNG-VSHLEAN-LSKVAVF-N
Sequence81-P-CG-EA-ABD        YDGV-MFVNYSNFKYT-NASKSPDKGVGATNG-VSHLEAN-FSKVAVF-N
Sequence150-NP-DA-Sen-BC      HDGV-MFVDYSNFKYT-NASKNPNKGVGVTNG-VSHLEAG-FDKVAVF-N
Sequence90-NP-UD-Vit-ABD      YDGV-MFVNYSNSNTP-TPPRVLIKGVGTTNG-VSHLEAN-FSKVAVF-Y
Sequence136-P-CG-Col-AACC     HDGV-MFVDYSNFKYT-NASKNPNKGVGVTNG-VSHLEAG-FSKVAVF-N
Sequence15-NP-UP-Jp-ABD       YDGV-MFVDYSNFKYT-NASKSPDKGVGATNG-VSHLEAN-FSKVAVF-N
Sequence108-NP-UD-Vit-ABD     YDGM-MFVDYSNFKYT-NASKSPDKGVSATNG-VSHLEAN-FSKVAVF-N
Sequence104-P-CG-OCC-ABCC     HDGRGWFVNYSYFPNTPTAPRVLIRVVGATNG-VSHLGSKVLTKEAVF-D
Sequence179-NP-UD-Col-ABCC    HDGV-MFVDYSNFKYT-NASKNPNKGVGATNG-VSHLEAG-FDKVAIF-N
Sequence37-NP-GA-Jp-ABCCC     HDGV-MFVNYSNFKYT-NASKSPDKGVGATNG-CFPLEAN-LSKVAVF-N
Sequence89-NP-UD-Vit-ABD      YDGV-MFVDYSNFKYT-NASKSPDKGVGATNG-VSHLEAN-FSKVAVF-N
Sequence92-P-CG-Vit-ABD       YDGV-MFVNYSNFKYT-NASKSPDKGLGATNG-VSHLEAN-FSKVAVF-N
Sequence49-NP-GA-Jp-ABC       HDGV-MFVNYSNFKYT-NASKSPDKGVGATNG-VSHLEAN-LSKVAVF-N
Sequence181-P-CG-Col-ABCC     HDGV-MFVDYSNFKYT-NASKNPNKGVGATNG-VSHLDAG-FSKVAVF-N
Sequence25-NP-GA-Jp-ABD       YDGV-MFVDYSNFKYT-NASKSPNKGLGATNG-VSHLEVN-FSKVAVF-N
Sequence4-P-CG-Jp-ABD         YDGV-MFVNYSNFKYT-NASKSPNKGLGTTNG-VSHLEAN-FSKVAVF-N
Sequence121-NP-G-Jp-ABCC      HDGV-MFVDYSNFKYT-NASKNPNKGVGVTNG-VSHLEAG-FNKMAVF-N
4dvy                          HDGV-MFVDYSNFKYT-NASKNPNKGVGATNG-VSHLEAG-FNKVAVF-N
Sequence186-NP-UP-Jp-ABD      YDGL-MFVNYSNFKYT-NASKSPDKGVGATNG-VSHLEAN-FSKVAVF-N
Sequence62-NP-UD-OCC-ABD      HDGV-MFVNYSNFKYT-NASKSPDKGVGATNG-VSHLEAN-LSKVAVF-N
Sequence9-NP-GCA-Jp-ABD       HDGV-MFVNYSNFKYT-NASKSPNKGLGTTNG-VSHLEAN-FSKVAVF-K
Sequence12-NP-UP-Jp-ABD       YDGV-MFVNYSNFKYT-NASKSPNKGVGTTNG-VSHLEAN-FSKVAVF-N
Sequence35-NP-GA-Jp-ABABD     YDGV-MFVDYSNFKYT-NASKSPNKGLGATNG-VSHLEAN-FSKVAVF-N
Sequence54-NP-GA-OCC-ABC      HDGV-MFVNYSNFKYT-NASKSPDKGVGATNG-VSHLEAN-LSKVAVF-N
Sequence109-P-CG-Jp-ABD       YDGV-MFVDYSNFKYT-NASKSPDKGVGATNG-VSHLEAN-FSKVAVF-N
Sequence96-NP-UD-Vit-ABD      MMGV-IFVNYSNFNTP-TPPRVLIRVWVLRMG-VSHLEAN-FSKVAVF-N
Sequence80-P-CG-EA-ABD        YDGV-MFVNYSNFKYT-NASKSPDKGVGATNG-VSHLEAN-FSKVAVF-N
Sequence82-P-CG-EA-ABD        YDGV-MFVNYSNFKYT-NASKSPNKGVGATNG-VSHLEAN-FSKVAVF-N
Sequence10-NP-GCA-Jp-ABD      YDGV-MFVDYSNFKYT-NASKSPNKGLGATNG-VSHLEAN-FSKVAVF-N
Sequence19-NP-UP-Jp-ABBD      YDGV-MFVNYSNFKYT-NASKSPNKGLGATNG-VSHLEAN-FSKVAVF-D
Sequence115-NP-UG-Jp-ABC      HDGV-MFVDYSNFKYT-NASKSPHKGVGVTNG-VSHLDAG-FSKVAVF-N
Sequence45-NP-GA-Jp-ABCC      HDGV-MFVDYSNFKYT-NASKSPNKGVGVTNG-VSHLEAG-FNKVAVF-N
Sequence119-NP-UG-Jp-ABC      HDGV-MFVDYSNFKYT-NASKNPNKGVGVTNG-VSHLEAG-LNKVAVF-N
Sequence70-NP-GA-Ch-ABD       YDGV-MFVNYSNFKYT-NASKSPDKGVGATNG-VSHLEAN-FSKVAVF-N
Sequence52-NP-UD-EA-ABD       YDGV-MFVNYSNFKYT-NASKSPDKGVGATNG-VSHLEAN-FSKVAVF-N
```

```
Sequence97-P-CG-Vit-ABD        Y D G V - M F V N Y S N F K Y T - N A S K S P D K G L G A T N G - V S H L E A N - F S K V A V F - N
Sequence94-NP-UD-Vit-ABD       A C C L - L I I L I S N T P T P - - - P R V L I R V Q A L R M A - F P I W K R I - L A R Q L S F I C
Sequence176-NP-GA-Col-ABCC     H D G V - M F V D Y S N F K Y T - N A S K N P N K G V G V T N G - V S H L D A G - F S K V A V F - N
Sequence68-NP-D-Am-ABBC        H D G V - M F V D Y S N F K Y T - N A S K N P N K G V G A T N G - V S H L D A G - F S K V A V F - N
Sequence47-NP-GA-Jp-ABD        Y D G V - M F V N Y S N F K Y T - N A S K S P D K G V G A A N G - V S H L E A N - F S K V A V F - N
Sequence56-NP-UD-OCC-ABC       H D G V - M F V N Y S N F K Y T - N A S K S P D K G V G A T N G - V S H L E A N - L S K V A V F - N
Sequence31-NP-GA-Jp-ABD        Y D G V - M F V N Y S N F K Y T - N A S K S P D K G V G A T N G - V S H L E A N - F S K V A V F - N
Sequence46-NP-UD-Jp-ABD        H D G V - M F V N Y S N F K Y T - N V S K S P D K G V G T T N G - V S H L E A N - F S K V A V F - N
Sequence102-NP-UG-Vit-ABD      Y D G V - M F V D Y S N F K Y T - N A S K S P D K G V S A T N G - V S H L E A I - L A K V A V F - Y
Sequence71-P-CG-EA-ABD         Y D G V - M F V N Y S N F K Y T - N A S K S P N K G L G A T N G - V S H L E A N - F S K V A V F - N
Sequence30-NP-GA-Jp-ABBD       Y D G V - M F V N Y S N F K Y T - N A S K S P D K G V G A T N G - V S H L E A N - F S K V A V F - N
Sequence28-NP-GA-Jp-AABD       Y D G V - M F V D Y S N F K Y T - N A S K S P N K G L G A T N G - V S H L E A N - F S K V A V F - N
Sequence98-P-CG-Vit-AD         Y D G G - M F V N Y S N F K Y P - N A S Q S P D K G V G T T N G - V S H L E A N - F S K V A V F - N
Sequence99-P-CG-Vit-ABD        Y D G V - M F V D Y S N F K Y T - N A S K S P D K G V G T T N G - V S H L E A N - F S K V A V F - N
Sequence24-NP-UD-USA-ABD       Y D G V - M F V N Y S N F K Y T - N A S K S P D K G V D T T N G - V S P L E A N - F S K V A V F - N
Sequence124-NP-G-Jp-ABC        H D G V - M F V N Y S N F K Y T - N A S Q S P N K G V G V T N G - V S H L E A G - F S K V A V F - N
Sequence77-P-CG-OCC-ABCC       H D G V - M F V N Y S N F K Y T - N A S K S P D K G V G A T N G - V S H L E A N - F S K V A V F - N
Sequence113-NP-UG-Jp-ABC       H D G V - I F V D Y S N F K Y T - N A S K S P N K G V G V T N G - V S H L E A G - F N K V A V F - N
Sequence88-NP-UD-Vit-ABD       Y D G V - M F V D Y S N F K Y T - N A S Q S P D K G V S A T N G - V S H L E A N - F S K G A V L - N
Sequence100-NP-UD-Vit-ABD      Y D G V - M F V N Y S H F K Y T - N A S K S P D K G V G T T N G - V S H L E A N - F S K V A V F - N
Sequence137-NP-G-Col-ABCC      H D G V - M F V D Y S N F K Y T - N A S K N P N K G V G V T N G - V S H L D A G - F N K V A V F - N
Sequence117-NP-G-Jp-ABC        H D G V - M F V N Y S N F K Y T - N A S K N P N K G V G V T N G - V S H L E A G - F N K V A V F - N
Sequence171-NP-UD-Col-ABCC     H D G V - M F V D Y S N F K Y T - N A S K N P N K G V G A T N G - V S H L D A G - F S K V A V F - N
Sequence180-P-CG-Col-ABC       H D G V - M F V D Y S N F K Y T - N A S K N P N K G V G V T N G - V S H L D A G - F S K V A V F - N
Sequence112-P-CG-Jp-ABCC       H D G V - M F V N Y S N F K Y T - N A S K N P N K G V G V T N G - V S H L E A G - F N K V A V F - N
Sequence127-NP-UD-Jp-ABC       H D G V - M F V N Y S N F K Y T - N A S K N P N K G V G V T N G - V S H L E A G - F D K V A V F - N
Sequence51-NP-GA-OCC-ABC       H D G V - M F V N Y S N F K Y T - N A S K S P D K G V G T T N G - V S H L E A N - F S K V A V F - N
Sequence73-P-CG-EA-ABD         Y D G V - M F V N Y S N F K Y T - N A S K S P D K G V G A T N G - V S H L E A N - F S K I A V F - N
Sequence183-NP-UD-Col-ABD      H D G V - M F V D Y S N F K Y T - N A S K N P N K G V G V T N G - V S H L D A G - F S K V A V F - N
Sequence34-NP-UD-Jp-ABDD       Y D G V - M F V N Y S N F K Y T - N A S K S P N K G V G A T N G - V S H L E A N - F S K V A V F - N
Sequence21-P-CG-Jp-ABD         Y D G V - M F V N Y S N F K Y T - N A S K S P D K G V G T T N G - V S H L E A N - F S K V A V F - N
Sequence32-NP-GA-Jp-ABD        Y D G V - M F V N Y S N F K Y N S T L P R V L I R A L G A T N G - V S H L E A N - F S K V A V F - N
Sequence116-NP-UD-Jp-ABC       H D G V - M F V D Y S N F K Y T - N A S K S P N K G V G V T N G - V S H L D A G - F S K V A V F - N
Sequence8-NP-GCA-Jp-ABD        Y D G V - M F V N Y S N F K Y T - N A S K S P N K G V G A T N G - V S H L E A N - F S K V A V F - N
Sequence83-P-CG-EA-ABD         Y D G V - M F V N Y S N F K Y T - N A S K S P D K G V G A T N G - V S H L E A N - F S K V A V F - N
Sequence48-NP-UD-OCC-ABC       H D G V - M F V N Y S N F K Y T - N A S K S P D K G V G A T N G - V S H L E A N - L S K V A V F - N
Sequence120-NP-G-Jp-ABC        H D G V - M F V D Y S N F K Y T - N A S Q N P N K G V G A T N G - V S H L D A G - F S K V A V F - N
Sequence187-NP-UG-Jp-ABCCC     H D S V - M F V N Y S N F K Y T - N A S K S P D K G V G V T N G - V S H L D A G - F S K V A V F - N
Sequence118-NP-G-Jp-ABD        Y D G V - M F V N Y S N F K Y T - N A S K S P D K G V G T T N G - V S H L E A N - F S K V A V F - N
Sequence87-NP-UG-Vit-ABD       Y D G M - M F V N Y S N F K Y T - N A S K S P D K G V G A P N G R F P I W E Q N - F S K V A V F - Y
```

```
                               601                                                                                             650
Sequence140-NP-GA-Col-ABABC    L P D L N N L A I T S F V R R N L E D K L I A - K G L - S L Q E V N K L I K D - - F L S S N K E L V
Sequence123-NP-UD-Jp-ABD       L P N L N N L A I T N Y I R R D L E D K L W A - K G L - S S Q E A N K L I K D - - F L N S N K E M V
Sequence111-NP-G-Jp-ABD        L P N L N N L A I T N Y I R R D L E D K L L A - K G L - S P Q E A N K L I K D - - F L N S N K E M V
Sequence57-NP-GA-OCC-AB        L P N L N N L A I N S Y I R R D L E D K L M A - K G L - S P Q E A N K L I K D - - F L N S N K E L V
Sequence172-P-CG-Col-ABCC      L P D L N N L A I T S F V R R N L E D K L I A - K G L - T P Q E A N K L I K D - - F L S S N K E L V
Sequence110-NP-UD-Jp-ABD       L P N L N N L A I T N Y I R R D L E D K L W V - K G L - S P Q E A S K L I K D - - F L N S N K E M V
Sequence95-NP-UD-Vit-ABD       L P N L N N L A I T N Y I R R D L E D N C - - - - G L - K D C P H K K L I S S S R L F E Q H Q D W L
Sequence107-P-CG-Vit-ABD       L P N L N N L A I T N Y I R R D L E D K L W A - K G L - S S Q E A N K L I K D - - F L N S N K E L V
Sequence106-P-CG-Vit-ABD       L P K L N N L A I T N F I R Q D L E D K L V A - K G L - S L Q E T N K L I K D - - F L S N N K E L V
Sequence105-NP-UD-Vit-ABD      C P N L N N L A I T N S I R R D L E D K L W A - K G L - S P Q E A N K L I K D - - F L N S N K E L V
Sequence126-NP-UG-Jp-ABC       L P N L N N L A I T S S V R R N L E D K L I A - K G L - P P Q D A N K L V K D - - F L S S N K E L V
Sequence135-P-CG-Col-ABCCC     L P D L N N L A I T S F V R R H L E D K L V V - K G L - S L Q E T N K L I K D - - F L S S N K E L V
Sequence138-NP-GA-Col-ABC      L P D L N N L A I T S F V R R N L E D K L I A - K G L - S L R E A N K L I K D - - F L S S N K E L V
Sequence13-NP-UP-Jp-ABD        L P N L N N L A I T N Y I R R D L E D K L L A - K G L - S P Q E A N K L I I D - - F L N S N K E L V
Sequence11-NP-UP-Jp-ABD        L P N L N N L A I T N Y I R R D L E D K L W A - K G L - S P Q E A N K L I K D - - F L N S N K E M V
Sequence50-NP-GA-OCC-ABC       L P N L N N L A I T S Y I R R D L E D K L W A - K G L - S P Q E T N K L I K D - - F L N S N K E L V
Sequence26-NP-GA-Jp-ABD        L P N L N N L A I T N Y I R R D L E D K L W A - K G L - S R Q E S N K L I K D - - F L N S N K E M V
Sequence55-NP-UD-OCC-ABCCC     L P N L N N L A I T S Y I R R D L E D K L W A - K G L - S P Q E V N K L I K D - - F L N S N K E L V
Sequence178-NP-M-Col-ABCC      L S D L N N L A I T S F V R R N L E D K L V A - K G L - S L Q E V N K L I K D - - F L S S N K E L V
```

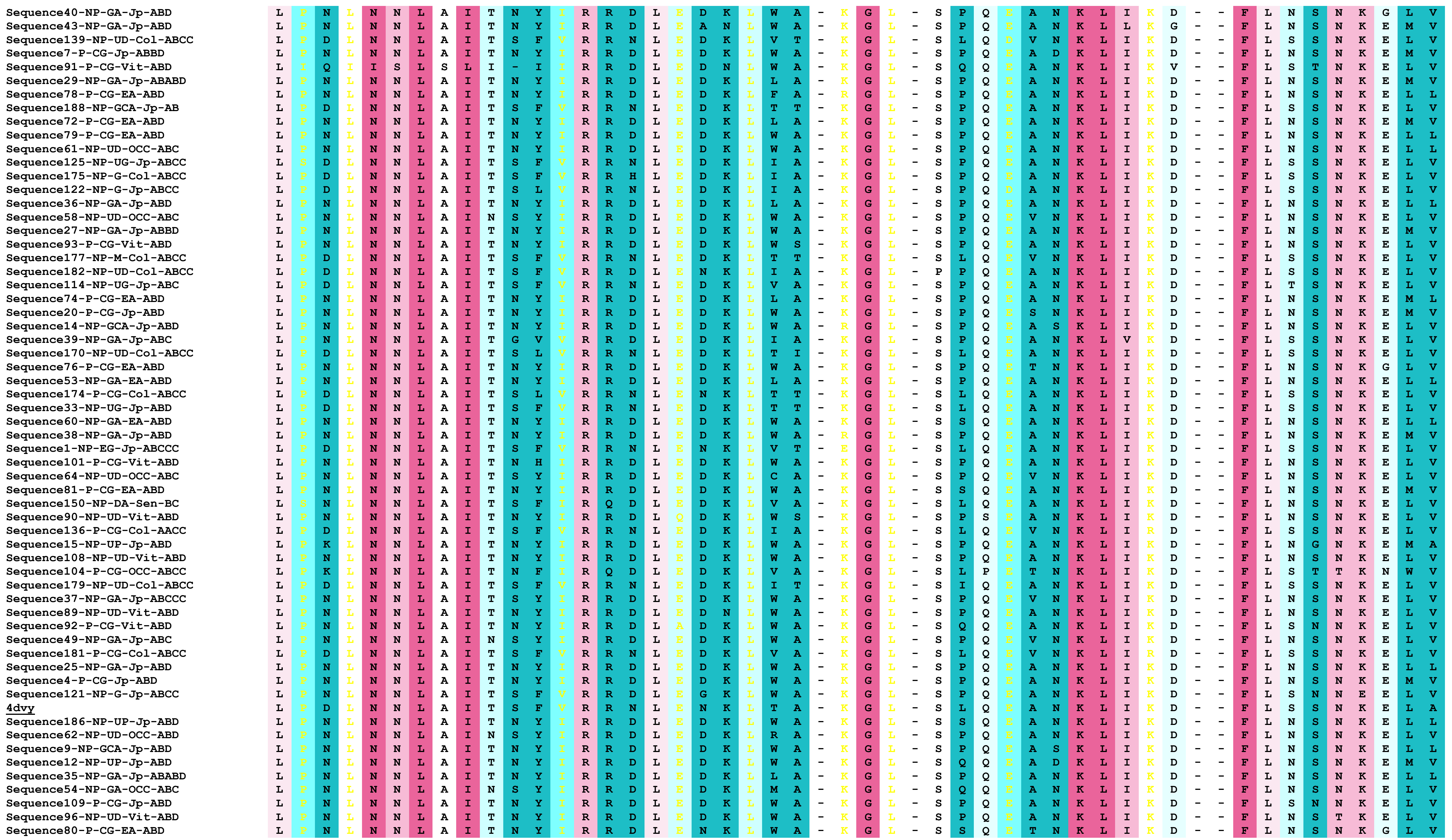
Sequence40-NP-GA-Jp-ABD LPNLNNLAITNYIRRDLEDKLWA-KGL-SPQEANKLIKD--FLNSNKGLV
Sequence43-NP-GA-Jp-ABD LPNLNNLAITNYIRRDLEANLWA-KGL-SPQEANKLLKG--FLNSNKEMV
Sequence139-NP-UD-Col-ABCC LPDLNNLAITSFVRRNLEDKLVT-KGL-SLQDVNKLIKD--FLSSNKELV
Sequence7-P-CG-Jp-ABBD LPNLNNLAITNYIRRDLEDKLWA-KGL-SPQEADKLIKD--FLNSNKEMV
Sequence91-P-CG-Vit-ABD LIQIISLSLI-IIRRDLEDNLWA-KGL-SQQEANKLIKV--FLSTNKELV
Sequence29-NP-GA-Jp-ABABD LPNLNNLAITNYIRRDLEDKLLA-KGL-SPQEANKLIKD--FLNSNKEMV
Sequence78-P-CG-EA-ABD LPNLNNLAITNYIRRDLEDKLFA-RGL-SPQEANKLIKD--FLNSNKELL
Sequence188-NP-GCA-Jp-AB LPDLNNLAITSFVRRNLEDKLTT-KGL-SPQEANKLIKD--FLSSNKELV
Sequence72-P-CG-EA-ABD LPNLNNLAITNYIRRDLEDKLLA-KGL-SPQEANKLIKD--FLNSNKEMV
Sequence79-P-CG-EA-ABD LPNLNNLAITNYIRRDLEDKLWA-KGL-SPQEANKLIKD--FLNSNKELL
Sequence61-NP-UD-OCC-ABC LPNLNNLAITNYIRRDLEDKLWA-KGL-SPQEANKLIKD--FLNSNKELL
Sequence125-NP-UG-Jp-ABCC LSDLNNLAITSFVRRNLEDKLIA-KGL-SPQEANKLIKD--FLSSNKELV
Sequence175-NP-G-Col-ABCC LPDLNNLAITSFVRRHLEDKLIA-KGL-SPQEANKLIKD--FLSSNKELV
Sequence122-NP-G-Jp-ABCC LPDLNNLAITSLVRRNLEDKLIA-KGL-SPQDANKLIKD--FLSSNKELV
Sequence36-NP-GA-Jp-ABD LPNLNNLAITNYIRRDLEDKLLA-KGL-SPQEANKLIKD--FLNSNKELL
Sequence58-NP-UD-OCC-ABC LPNLNNLAINSYIRRDLEDKLWA-KGL-SPQEVNKLIKD--FLNSNKELV
Sequence27-NP-GA-Jp-ABBD LPNLNNLAITNYIRRDLEDKLWA-KGL-SPQEANKLIKD--FLNSNKEMV
Sequence93-P-CG-Vit-ABD LPNLNNLAITNYIRRDLEDKLWS-KGL-SPQEANKLIKD--FLNSNKELV
Sequence177-NP-M-Col-ABCC LPDLNNLAITSFVRRNLEDKLTT-KGL-SLQEVNKLIKD--FLSSNKELV
Sequence182-NP-UD-Col-ABCC LPDLNNLAITSFVRRDLENKLIA-KGL-PPQEANKLIKD--FLSSNKELV
Sequence114-NP-UG-Jp-ABC LPDLNNLAITSFVRRNLEDKLVA-KGL-SPQEANKLIKD--FLTSNKELV
Sequence74-P-CG-EA-ABD LPNLNNLAITNYIRRDLEDKLLA-KGL-SPQEANKLIKD--FLNSNKEML
Sequence20-P-CG-Jp-ABD LPNLNNLAITNYIRRDLEDKLWA-KGL-SPQESNKLIKD--FLNSNKEMV
Sequence14-NP-GCA-Jp-ABD LPNLNNLAITNYIRRDLEDKLWA-RGL-SPQEASKLIKD--FLNSNKELV
Sequence39-NP-GA-Jp-ABC LPNLNNLAITGVVRRDLEDKLIA-KGL-SPQEANKLVKD--FLSSNKELV
Sequence170-NP-UD-Col-ABCC LPDLNNLAITSLVRRNLEDKLTI-KGL-SLQEANKLIKD--FLSSNKELV
Sequence76-P-CG-EA-ABD LPNLNNLAITNYIRRDLEDKLWA-KGL-SPQETNKLIKD--FLNSNKGLV
Sequence53-NP-GA-EA-ABD LPNLNNLAITNYIRRDLEDKLLA-KGL-SPQEANKLIKD--FLNSNKELL
Sequence174-P-CG-Col-ABCC LPDLNNLAITSLVRRNLENKLTT-KGL-SLQEANKLIKD--FLSSNKELV
Sequence33-NP-UG-Jp-ABD LPDLNNLAITSFVRRNLEDKLTT-KGL-SLQEANKLIKD--FLSSNKELV
Sequence60-NP-GA-EA-ABD LPNLNNLAITNYIRRDLEDKLWA-KGL-SSQEANKLIKD--FLNSNKELL
Sequence38-NP-GA-Jp-ABD LPNLNNLAITNYIRRDLEDKLWA-RGL-SPQEANKLIKD--FLNSNKEMV
Sequence1-NP-EG-Jp-ABCCC LPDLNNLAITSFVRRNLENKLVT-EGL-SLQEANKLIKD--FLSSNKELV
Sequence101-P-CG-Vit-ABD LPNLNNLAITNHIRRDLEDKLWA-KGL-SPQEANKLIKD--FLNSNKELV
Sequence64-NP-UD-OCC-ABC LPNLNNLAITSYIRRDLEDKLCA-KGL-SPQEVNKLIKD--FLNSNKELV
Sequence81-P-CG-EA-ABD LPNLNNLAITNYIRRDLEDKLWA-KGL-SSQEANKLIKD--FLNSNKEMV
Sequence150-NP-DA-Sen-BC LSNLNNLAITSFIRQDLEDKLVA-KGL-SLQEANKLIKD--FLNSNKELV
Sequence90-NP-UD-Vit-ABD LPNLNNLAITNYIRRDLQDKLWS-KGL-SPSEANKLIKD--FLNSNKELV
Sequence136-P-CG-Col-AACC LPDLNNLAITSFVRRNLEDKLIA-KGL-SLQEVNKLIKD--FLSSNKELV
Sequence15-NP-UP-Jp-ABD LPKLNNLAITNYIRRDLEDKLWA-KGL-SPQEANKLIKD--FLNGNKEMA
Sequence108-NP-UD-Vit-ABD LPNLNNLAITNYIRRDLEDKLWA-KGL-SPQEANKLIKD--FLNSNKELV
Sequence104-P-CG-OCC-ABCC LPKLNNLAITNFIRQDLEDKLVA-KGL-SLPETNKLIKD--FLSTTKNWV
Sequence179-NP-UD-Col-ABCC LPDLNNLAITSFVRRNLEDKLIT-KGL-SIQEANKLIKD--FLSSNKELV
Sequence37-NP-GA-Jp-ABCCC LPNLNNLAITSYIRRDLEDKLWA-KGL-SPQEVNKLIKD--FLNSNKELV
Sequence89-NP-UD-Vit-ABD LPNLNNLAITNYIRRDLEDNLWA-KGL-SPQEANKLIKD--FLNSNKELV
Sequence92-P-CG-Vit-ABD LPNLNNLAITNYIRRDLADKLWA-KGL-SQQEANKLIKD--FLSNNKELV
Sequence49-NP-GA-Jp-ABC LPNLNNLAINSYIRRDLEDKLWA-KGL-SPQEVNKLIKD--FLNSNKELV
Sequence181-P-CG-Col-ABCC LPDLNNLAITSFVRRNLEDKLVA-KGL-SLQEVNKLIRD--FLSSNKELV
Sequence25-NP-GA-Jp-ABD LPNLNNLAITNYIRRDLEDKLWA-KGL-SPQEANKLIKD--FLNSNKELL
Sequence4-P-CG-Jp-ABD LPNLNNLAITNYIRRDLEDKLWA-KGL-SPQEANKLIKD--FLNSNKEMV
Sequence121-NP-G-Jp-ABCC LPNLNNLAITSFVRRDLEGKLWA-KGL-SPQEANKLIKD--FLSNNEELV
4dvy LPDLNNLAITSFVRRNLENKLTA-KGL-SLQEANKLIKD--FLSSNKELA
Sequence186-NP-UP-Jp-ABD LPNLNNLAITNYIRRDLEDKLWA-KGL-SSQEANKLIKD--FLNSNKELL
Sequence62-NP-UD-OCC-ABD LPNLNNLAINSYIRRDLEDKLRA-KGL-SPQEANKLIKD--FLNSNKELV
Sequence9-NP-GCA-Jp-ABD LPNLNNLAITNYIRRDLEDKLWA-KGL-SPQEASKLIKD--FLNSNKELL
Sequence12-NP-UP-Jp-ABD LPNLNNLAITNYIRRDLEDKLWA-KGL-SQQEADKLIKD--FLNSNKEMV
Sequence35-NP-GA-Jp-ABABD LPNLNNLAITNYIRRDLEDKLLA-KGL-SPQEANKLIKD--FLNSNKELL
Sequence54-NP-GA-OCC-ABC LPNLNNLAINSYIRRDLEDKLMA-KGL-SQQEANKLIKD--FLNSNKELV
Sequence109-P-CG-Jp-ABD LPNLNNLAITNYIRRDLEDKLWA-KGL-SPQEANKLIKD--FLSNNKELV
Sequence96-NP-UD-Vit-ABD LPNLNNLAITNYIRRDLEDKLWA-KGL-SPQEANKLIKD--FLNSTKELV
Sequence80-P-CG-EA-ABD LPNLNNLAITNYIRRDLENKLWA-KGL-SSQETNKLIKD--FLNSNKGLV

| | |
|---|---|
| Sequence82-P-CG-EA-ABD | L P N L N N L A I T N Y I R R D L E D K L L A - K G L - S P Q E A N K L I K D - - F L N S N K E L L |
| Sequence10-NP-GCA-Jp-ABD | L P N L N N L A I T N Y I R R D L E D K L W A - K G L - S P Q E A N K L I K D - - F L N S N K E L L |
| Sequence19-NP-UP-Jp-ABBD | L P N L N N L A I T N Y I R R D L E D K L W A - K G L - S S Q E A N K L I K D - - F L N S N K E M V |
| Sequence115-NP-UG-Jp-ABC | L P D L N N L A I T S F V R R N L E D K L I A - K G L - S P Q E T N K L I K G - - F L S S N K E L V |
| Sequence45-NP-GA-Jp-ABCC | L P D L N N L A I T S F V R R N L E D K L V A - K G L - S P Q E A N K L I K D - - F L S S N K E L V |
| Sequence119-NP-UG-Jp-ABC | L P N L N N L A I T N F I R R D L E Y K L R A - K G L - S P Q E A H K L I K D - - F L S N N K E L V |
| Sequence70-NP-GA-Ch-ABD | L P N L N N L A I T N Y I R R D L E D K L W A - K G L - S S Q E A N K L I K D - - F L N S N K E L L |
| Sequence52-NP-UD-EA-ABD | L P N L N N L A I T N Y I R R D L E D K L W A - K G L - S S Q E A N K L I K D - - F L N S N K E L L |
| Sequence97-P-CG-Vit-ABD | L P N L N N L A I S N Y I R R D L E D K L W A - K G L - S P Q E A N K L I K D - - F L N S N K E L V |
| Sequence94-NP-UD-Vit-ABD | L I E I I S L S L I I Y G E I Q - K T T L W A - K G L - S P Q E A S K L I K D - - F L N S N K E M V |
| Sequence176-NP-GA-Col-ABCC | L P D L N N L A I T S F V R R N L E D K L T T - K G L - S L Q E A N K L I K D - - F L S S N K E L V |
| Sequence68-NP-D-Am-ABBC | L P D L N N L A I T S L V R R D L E D K L I A - K G L - S L Q E V N K L I K D - - F L N S N K E L V |
| Sequence47-NP-GA-Jp-ABD | L P N L N N L A I T N Y I R R D L E D K L L A - K G L - S P Q E A N K L I K D - - F L N S N K E M V |
| Sequence56-NP-UD-OCC-ABC | L P N L N N L A I N S Y I R R D L E D K L W A - K G L - S P Q E V N K L I K D - - F L N S N K E L V |
| Sequence31-NP-GA-Jp-ABD | L P D L N N L A I T N Y I R R D L E D K L L A - K G L - S P Q E T N K L I K D - - F L N S N K E L A |
| Sequence46-NP-UD-Jp-ABD | L P N L N N L A I T N Y I R R D L E D K L L A - K G L - S P Q E A N K L I K D - - F L N S N K E L V |
| Sequence102-NP-UG-Vit-ABD | L P N L N N L A I T N H I R R D L E D K L W A - K G L - S P Q E A N K L I Q D - - F L N S H K G I G |
| Sequence71-P-CG-EA-ABD | L P T L N N L A I T N Y I R R D L E D K L W A - K G L - S P Q E A N K L I K D - - F L N S N K E L L |
| Sequence30-NP-GA-Jp-ABBD | L P N L N N L A I T N Y I R R D L E D K L W A - K G L - S P Q E A N K L I K D - - F L N S N K E M V |
| Sequence28-NP-GA-Jp-AABD | L P N L N N L A I T N Y I R R D L E D K L L A - K G L - S P Q E A N K L I K D - - F L N S N K E L V |
| Sequence98-P-CG-Vit-AD | L P N L N N L A I T N Y I R R D L E D K L W A - K G L - S P Q E A N K L I K D - - F L N S N K E L V |
| Sequence99-P-CG-Vit-ABD | R P N L N N L S I T N Y L R A R F R R H I V G L K D Y - P P Q E A D K L I K D - - F F N S N K E M V |
| Sequence24-NP-UD-USA-ABD | L P N L N N L A I T S V V R R D L E D K L I S - K G L - S P Q E A N K L I K D - - F L N G N K E L A |
| Sequence124-NP-G-Jp-ABC | L P N L N D L A I T S S V R Q N L E D K L I A - K G L - S P Q D A N K L V K D - - F L S S N K E L V |
| Sequence77-P-CG-OCC-ABCC | L P N L N N L A I T S Y M R R D L E G K L S A - K G L - S L Q E A N K L I K D - - F L N S N K E L V |
| Sequence113-NP-UG-Jp-ABC | L P D L N N L T I T S F V R R N L E D K L V A - K G L - S P Q E A N K L I K D - - F L S S N K E L V |
| Sequence88-NP-UD-Vit-ABD | L P N F N N L A I P N L Y K A R F R K T N C G A K G M - S Q Q E A N K L I K V - - F L S T N K E L V |
| Sequence100-NP-UD-Vit-ABD | L P N L N N L A I T N Y I R R D L E D K L W A - K G L - S P Q E V N K L I K D - - F L N S N K E M V |
| Sequence137-NP-G-Col-ABCC | L P D L N N L A I T S F V R R N L E D K L I A - K G L - S L Q D T N K L I K D - - F L S S N K E L V |
| Sequence117-NP-G-Jp-ABC | L P N L N N L A I T N F I R R D L E D K L I A - K G L - S P Q E A N K L I K D - - F L S N N K E L V |
| Sequence171-NP-UD-Col-ABCC | L P D L N N L A I T S F V R Q N L E D K L T T - K G L - S L Q E A N K L I K D - - F L S S N K E L V |
| Sequence180-P-CG-Col-ABC | L P D L N N L A I T S F V R R N L E D K L I A - K G L - S P Q D T N K L I K D - - F L S S N K E L V |
| Sequence112-P-CG-Jp-ABCC | L P N L N N I A I T S F I R R D L E D K L A T - K G L - S L Q E A N K L I K D - - F L S S N K E L V |
| Sequence127-NP-UD-Jp-ABC | L P N L N N L A I T N F I R R D L E G K L V A - K G L - S P Q E A N K L I K D - - F L S N N K E L V |
| Sequence51-NP-GA-OCC-ABC | L P N L N N L A I T S Y I R R D L E G K L S A - K G L - S L Q E A N K L I K D - - F L N S N K E L V |
| Sequence73-P-CG-EA-ABD | L P N L N N L A I T N H I R R D L E D K L W A - K G L - S S Q E A N K L I K D - - F L N S N K E L V |
| Sequence183-NP-UD-Col-ABD | L P D L N N L A I T S F V R R N L E D K L I A - K G L - S P Q D T N K L I K D - - F L S S N K E L V |
| Sequence34-NP-UD-Jp-ABDD | L P N L N N L A I T N Y I R R D L E D K L W A - K G L - S P Q E A S K L I K D - - F L N S N K E L V |
| Sequence21-P-CG-Jp-ABD | L P N L N N L A I T N Y I R R D L E D K L W A - K G L - S P Q E A N K L I K D - - F L N S N K E L L |
| Sequence32-NP-GA-Jp-ABD | L P N L N N L A I T N Y I R R D L E D K L W A - K G L - S P Q E A N K L I K D - - F L N S N K E L L |
| Sequence116-NP-UD-Jp-ABC | L P D L N N L A I T S F V R R N L E D K L I A - K G L - S P Q E T N K L I K G - - F L S S N K E L V |
| Sequence8-NP-GCA-Jp-ABD | L P N L N N L A I T N Y I R R D L E D K L W A - K G L - S P Q E A N K L I K D - - F L N S N K E L L |
| Sequence83-P-CG-EA-ABD | L P N L N N L A I T S Y I R R D L E D K L L A - K G L - S P Q E V N K L I K D - - F L N S N K E L L |
| Sequence48-NP-UD-OCC-ABC | L P N L N N L A I T S Y I R R D L E D K L W A - K G L - S P Q E V N K L I K D - - F L N S N K E L V |
| Sequence120-NP-G-Jp-ABC | L P D L N N L S I T S F V R Q N L E D K L I A - K G L - S I Q E A N K L I K D - - F L S S N K E L V |
| Sequence187-NP-UG-Jp-ABCCC | L P D L N N L A I T S F V R R N L E N K L V T - E G L - S L Q E A N K L I K D - - F L S S N K E L V |
| Sequence118-NP-G-Jp-ABD | L P N L N N L A I T N Y I R R D L E D K L W A - K G L - S P Q E A N K L I K D - - F L N S N K E M V |
| Sequence87-NP-UG-Vit-ABD | L P N L N N L A I T S Y I R R D L K D N C G A - K G I V P P R R L I S F I K D - - F L N S N K E M V |

| | 651 … 700 |
|---|---|
| Sequence140-NP-GA-Col-ABABC | G K A L N F N K A V A D A K N T G N Y D K V K K A Q K D L E K S L R K R E H L E K E V E K K L - E S |
| Sequence123-NP-UD-Jp-ABD | G K V S N F N K A V A E A K N T G N Y D G V K K A Q K D L E K S L R K R E H L E K E V A K K L - E S |
| Sequence111-NP-G-Jp-ABD | G K V S N L N K A V A E A K N T G N Y D E V K K A Q K D L E K S L R K R E H L E K E I A K K L - E S |
| Sequence57-NP-GA-OCC-AB | E K A L N F N K T V A E A K N T G N Y D E V K K A Q K D L E K S I R K R E H L E K E V A K K L - E S |
| Sequence172-P-CG-Col-ABCC | G K A L N F N K A V A D A K N T G N Y D E V K K A Q K D L E K S L R K R E H L E K E V E K K L - E S |
| Sequence110-NP-UD-Jp-ABD | G K V S N F N K A V A E A K N T G N Y D E V K K A Q K D L E K S L R K R E H L E K E V A K K L - E S |
| Sequence95-NP-UD-Vit-ABD | - K G S N F N K L Y L K L K H R - H Y D E V K K A Q K D L E K S L R K R E H L E K E V A K K L - E S |
| Sequence107-P-CG-Vit-ABD | G K V S N F N Q A V A E A K N T G N Y D E V K K A Q K D L E K S L R K R E H L E K E V A K K L - E S |
| Sequence106-P-CG-Vit-ABD | G K V S N F N Q A V A E A K N T G N Y D E V K K A Q K D L E K S L R K R E H L E K E V A K N L - E S |
| Sequence105-NP-UD-Vit-ABD | G K V V N F N Q A V A E A K N T G N Y D E V K K A Q K D P E K S L R K R E P F R E R S S E K I R E Q |
| Sequence126-NP-UG-Jp-ABC | G K A L N F N K A V A D A K N T G N Y D E V K K A Q K D L E K S L R K R E H L E K E V E K K L - E S |

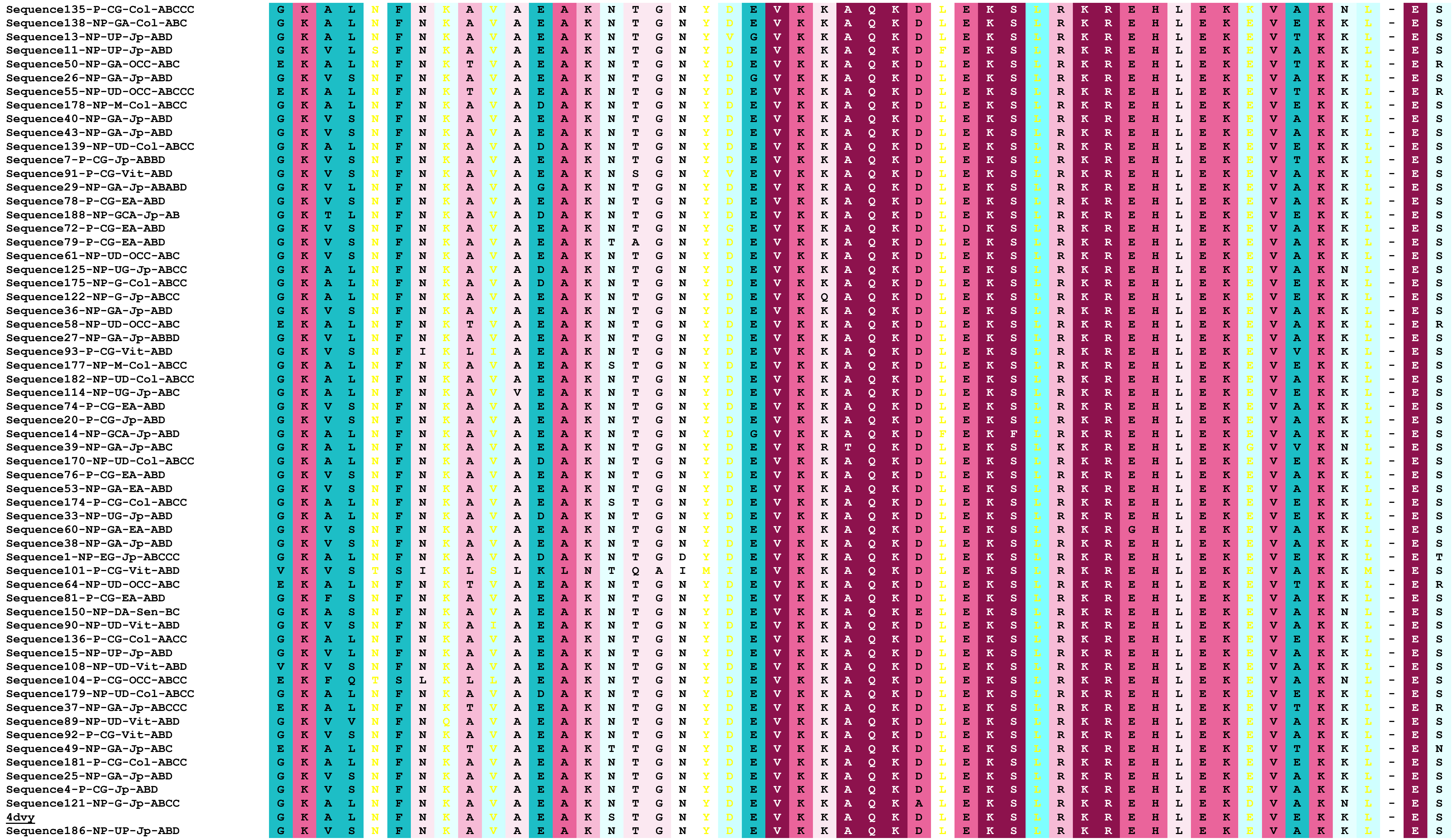
Sequence135-P-CG-Col-ABCCC
Sequence138-NP-GA-Col-ABC
Sequence13-NP-UP-Jp-ABD
Sequence11-NP-UP-Jp-ABD
Sequence50-NP-GA-OCC-ABC
Sequence26-NP-GA-Jp-ABD
Sequence55-NP-UD-OCC-ABCCC
Sequence178-NP-M-Col-ABCC
Sequence40-NP-GA-Jp-ABD
Sequence43-NP-GA-Jp-ABD
Sequence139-NP-UD-Col-ABCC
Sequence7-P-CG-Jp-ABBD
Sequence91-P-CG-Vit-ABD
Sequence29-NP-GA-Jp-ABABD
Sequence78-P-CG-EA-ABD
Sequence188-NP-GCA-Jp-AB
Sequence72-P-CG-EA-ABD
Sequence79-P-CG-EA-ABD
Sequence61-NP-UD-OCC-ABC
Sequence125-NP-UG-Jp-ABCC
Sequence175-NP-G-Col-ABCC
Sequence122-NP-G-Jp-ABCC
Sequence36-NP-GA-Jp-ABD
Sequence58-NP-UD-OCC-ABC
Sequence27-NP-GA-Jp-ABBD
Sequence93-P-CG-Vit-ABD
Sequence177-NP-M-Col-ABCC
Sequence182-NP-UD-Col-ABCC
Sequence114-NP-UG-Jp-ABC
Sequence74-P-CG-EA-ABD
Sequence20-P-CG-Jp-ABD
Sequence14-NP-GCA-Jp-ABD
Sequence39-NP-GA-Jp-ABC
Sequence170-NP-UD-Col-ABCC
Sequence76-P-CG-EA-ABD
Sequence53-NP-GA-EA-ABD
Sequence174-P-CG-Col-ABCC
Sequence33-NP-UG-Jp-ABD
Sequence60-NP-GA-EA-ABD
Sequence38-NP-GA-Jp-ABD
Sequence1-NP-EG-Jp-ABCCC
Sequence101-P-CG-Vit-ABD
Sequence64-NP-UD-OCC-ABC
Sequence81-P-CG-EA-ABD
Sequence150-NP-DA-Sen-BC
Sequence90-NP-UD-Vit-ABD
Sequence136-P-CG-Col-AACC
Sequence15-NP-UP-Jp-ABD
Sequence108-NP-UD-Vit-ABD
Sequence104-P-CG-OCC-ABCC
Sequence179-NP-UD-Col-ABCC
Sequence37-NP-GA-Jp-ABCCC
Sequence89-NP-UD-Vit-ABD
Sequence92-P-CG-Vit-ABD
Sequence49-NP-GA-Jp-ABC
Sequence181-P-CG-Col-ABCC
Sequence25-NP-GA-Jp-ABD
Sequence4-P-CG-Jp-ABD
Sequence121-NP-G-Jp-ABCC
4dvy
Sequence186-NP-UP-Jp-ABD

```
Sequence62-NP-UD-OCC-ABD      E K A L N F N K T V A E A K N T G N Y D E V K K A Q K D L E K S I R K R E H L E K E V M K K L - E N
Sequence9-NP-GCA-Jp-ABD       G K V S N F N K A V A E A K N T G N Y D E V K K A Q K D L E K S L R K R E H L E K E V V K K L - E S
Sequence12-NP-UP-Jp-ABD       G K V L S F N K A V A E A K N T G N Y D G V K K A Q K D L E K S L R K R E H L E K E V E K K L - E S
Sequence35-NP-GA-Jp-ABABD     G K V S N F N K A V A E A K N T G N Y D E V K K A Q K D L E K S L R K R E H L E K E V A K K L - E S
Sequence54-NP-GA-OCC-ABC      E I A L N F N K T V A E A K N T G N Y D E V K K A Q K D L E K S I R K R E H L E K E V T K K L - E N
Sequence109-P-CG-Jp-ABD       G K V S N F N K A V A E A K N T G N Y D E V K K A Q K D L E K S L R K R E H L E K E V A K K L - E S
Sequence96-NP-UD-Vit-ABD      G K V - Q L H K A V A E A K - T Q A I D E V K K A Q K D L E K S L R K R E H L E K E V A K K L - E S
Sequence80-P-CG-EA-ABD        G K V S N F N K A V A E A K N T G N Y D E V K K A Q K D L E K S L R K R E H L E K E V A K K L - E S
Sequence82-P-CG-EA-ABD        G K V S N F N K A V A E A K N T G N Y D E V K K A Q K D L E K S L R K R E H L E K E V A K K L - E S
Sequence10-NP-GCA-Jp-ABD      G K V S N F N K A V A E A K N T G N Y D G V K K A Q K D L E K S L R K R E H L E K E V A K K L - E S
Sequence19-NP-UP-Jp-ABBD      G K V S N F N K A V A E A K N T G N Y D E V K K A Q K D L E K S L R K R E H L E K E V A K K L - E S
Sequence115-NP-UG-Jp-ABC      G K A L N F N K A V A D A K N T G N Y D E V K K A Q K D L E K S L R K R E H L E K E V A K N L - E S
Sequence45-NP-GA-Jp-ABCC      G K A L N F N K A V A E A K N T G N Y D E V K K A Q K D L E K S L R K R E H L E K E V E K K L - E S
Sequence119-NP-UG-Jp-ABC      G K A L N F N K A V A E A K N T G N Y D E V K K A Q K D L E K S L K K R E H L E K E V A K N L - E S
Sequence70-NP-GA-Ch-ABD       G K V S N F N K A V A E A K N T G N Y D E V K K A Q K D L E K S L R K R E H L E K E V A K K L - E S
Sequence52-NP-UD-EA-ABD       G K V S N F N K A V A E A K N T G N Y D E V K K A Q K D L E K S L R K R E H L E K E V A K K L - E S
Sequence97-P-CG-Vit-ABD       G K L S N I N Q A V A E A K N T G N Y D E V K K A Q K D L E K S L R K R E H L E K E V A K K L - E S
Sequence94-NP-UD-Vit-ABD      G K V S N F N K A V A E A E N T G S Y D E V K K A Q K D L E K S L R K R E H L E K E V A K K L - E S
Sequence176-NP-GA-Col-ABCC    G K A L N F N K A V A E A K N T G N Y D E V K K A Q K D L E K S L R K R E H L E K E V E K K L - E S
Sequence68-NP-D-Am-ABBC       G K V L N L N K A V V E A K N T G N Y D E V K K A Q K D L E K S L R K R E H L E K E V E K K L - E S
Sequence47-NP-GA-Jp-ABD       G K A S Y F N K A V A E A K N T G N Y D E V K K A Q K D L E K S L R K R E H L E K E V A K K L - E S
Sequence56-NP-UD-OCC-ABC      E K A L N F N K T V A E A K N T G N Y D E V K K A Q K D L E K S L R K R E H L E K E V A K K L - E R
Sequence31-NP-GA-Jp-ABD       R K L S N F N K A V T E A K N T G N Y D E V K K A Q K D L E K S L R K R E H L E K E V T K K L - E S
Sequence46-NP-UD-Jp-ABD       E K A L N F N K T V A E A K N T G N Y D E V K K A Q K D L E K S L R K R E H L E K E V T K K L - E S
Sequence102-NP-UG-Vit-ABD     C K V S T S I K L L L K L K H R - R L D E V R K A Q K D L E K S L R K R E H L E K E V A K K M - E S
Sequence71-P-CG-EA-ABD        G K V S N F N K A V A E A K N T G N Y D E V K K A Q K D L E K S L R K R E H L E K E V A K K L - E S
Sequence30-NP-GA-Jp-ABBD      G K V S N L N K A V A E A K N T G N Y D G V K K A Q K D L E K S L R K R E L L E K E V A K K L - E S
Sequence28-NP-GA-Jp-AABD      G K V S N F N K A V A E A K N T G N Y D E V K K A Q K D L E K S L R K R E H L E K E V A K K L - E S
Sequence98-P-CG-Vit-AD        G K V S N F N K A V A E A K N T G N Y D E V K K A Q K D L E K S L R K R E H L E K E V A K K L - E S
Sequence99-P-CG-Vit-ABD       G K V S N F N T A I A E A K N T G N Y D E V K K A Q K D L E K S L R K R E H F E K E V A K K L - E S
Sequence24-NP-UD-USA-ABD      G K V L N F N K A E A K - K N T G N Y G E V K K A Q K D L E K S L R K R E H L E K E V V K K L - E S
Sequence124-NP-G-Jp-ABC       G K A L N F N K A V A D A K N T G N Y D E V K R A Q K D L E K S L R K R E H L E K E V E K K L - E S
Sequence77-P-CG-OCC-ABCC      E K A L N F N K T V A E A K N T G N Y D E V K K A Q K D L E K S I R K R E H L E K E V T K K M - E N
Sequence113-NP-UG-Jp-ABC      G K A L N F N K A V V E A K N T G N Y D E V K K A Q K D L E K S L R K R E H L E K E V E K K L - E S
Sequence88-NP-UD-Vit-ABD      G K V S N F N K A V A E A K N S G N Y V E V K K A Q K D L E K S L R K R E H L E K E V A K K L - E S
Sequence100-NP-UD-Vit-ABD     G K V L N F N Q A V A E A K N T G N Y D E V K K A Q K D L E K S L R K R E H L E K E V A K K L - E S
Sequence137-NP-G-Col-ABCC     E K A L N F N K A V A E A K N T G N Y D E V K K A Q K D L E K S L R K R E H L E K E V E K K L - E S
Sequence117-NP-G-Jp-ABC       G K A L N F N K A V A E A K N T G N Y D E V K K A Q K D L E K S L R K R E H L E K E V A K N L - E S
Sequence171-NP-UD-Col-ABCC    G K A L N F N K A V A D A K N T G N Y D E V K K A Q K D L E K S L R K R E H L E K E V E K K L - E S
Sequence180-P-CG-Col-ABC      E K A L N F N K A V A E A K N T G N Y D E V K K A Q K D L E K S L R K R E H L E K E V E K K L - E S
Sequence112-P-CG-Jp-ABCC      G K A L N F N K A V A E A K N T G N Y D E V K K A Q K D L E K S L R K R E H L E K E V A K N L - E S
Sequence127-NP-UD-Jp-ABC      G K A L N F N K A V A E A K N T G N Y D G V K K A Q K D L E K S L K K R E H L E K D V A K N L - E S
Sequence51-NP-GA-OCC-ABC      E K A L N F N K T V A E A K N T G N Y D E V K K A Q K D L E K S L R K R E H L E K E V T K K M - E S
Sequence73-P-CG-EA-ABD        R K V L N F N K A V A E A K N T G N Y D E V K K A Q K D L E K S L R K R E H L E K E V A K K L - E S
Sequence183-NP-UD-Col-ABD     E K A L N F N K A V A E A K N T G N Y D E V K K A Q K D L E K S L R K R E H L E K D V A K K L - E S
Sequence34-NP-UD-Jp-ABDD      G K T L N F N K A V A E A K N T G N Y D G V K K A Q K D L E K S I R K R E H L E K E V A K K L - E S
Sequence21-P-CG-Jp-ABD        G K V S N F N K A V A E A K N T G N Y D E V K K A Q K D L E K S L R K R E H L E K E V A K K L - E S
Sequence32-NP-GA-Jp-ABD       G K V S N F N K A V A G S K N T G N Y D E V K K A Q K D L E K S L R K R E H L E K E V A K K L - E S
Sequence116-NP-UD-Jp-ABC      G K A L N F N K A V A D A K N T G N Y D E V K K A Q K D L E K S L R K R E H L E K E V A K N L - E S
Sequence8-NP-GCA-Jp-ABD       G K V S N F N K A V A E A K N T G N Y D E V K K A Q K D L E K S L R K R E H L E K E V A K K L - E S
Sequence83-P-CG-EA-ABD        G K V S N F N K A V A G A K N T G N Y D E V K K A Q K D L E K S L R K R E H L E K E V A K K L - E S
Sequence48-NP-UD-OCC-ABC      E K A L N F N K T V A E A K N T G N Y D E V K K A Q K D L E K S L R K R E H L E K E V T K K L - E R
Sequence120-NP-G-Jp-ABC       G K A L N F N K A V A E A K N T G N Y D E V K K A Q K D L E K S L R K R E H L E K E V E K K L - E S
Sequence187-NP-UG-Jp-ABCCC    G K A L N F N K A V A D A K N T G N Y D E V K K A Q K D L E K S L R K R E H L E K E V E K K L - E S
Sequence118-NP-G-Jp-ABD       G K V S N F N K A V A E A K N T G N Y D G V K K A Q K D L E K S L R K R E H L E K E V A K K L - E S
Sequence87-NP-UG-Vit-ABD      G K V S N F N K A V A E A K N T G N Y D E V K K A Q K D L E K S L R K R E H L E K E V T K K L - E S

                              701                                                                                          750
Sequence140-NP-GA-Col-ABABC   K S G N K N K M E A K A Q A N S Q K D E I F A L I N K E A N R D A R A I A Y S Q N L K G I K R E L S
Sequence123-NP-UD-Jp-ABD      R N D N K N R M E A K A Q A N S Q K D K I F A L I N K E A S K E A R A V A F D P N L K G V R S E L S
Sequence111-NP-G-Jp-ABD       R N D N K N R M E A K A Q A N S Q K D K I F A L I N K E A S K E A R A A A F N P N L K G I R S E L S
```

| | |
|---|---|
| Sequence57-NP-GA-OCC-AB | RNDNKNRMEAKAQANSQKDKIFALINQEASKEARATACVQNLKGIRSELS |
| Sequence172-P-CG-Col-ABCC | KSGNKNKMEAKAQANSQKDEIFALINKEANRDARAITYAQNLKGIKRELS |
| Sequence110-NP-UD-Jp-ABD | RNDNKNGMEAKAQANSQKDKIFALINKEASKEARAAAFDPNLKGVRSELS |
| Sequence95-NP-UD-Vit-ABD | RNDNKNRMEAKAQANSQKDKIFALINKEASKEARAAAFDPNLKGVRSELS |
| Sequence107-P-CG-Vit-ABD | RNDNKNRMEAKAQANSQKDKIFALINKEASKEARAAAFDPNLKGVRSELS |
| Sequence106-P-CG-Vit-ABD | RSNNKNKMEAKSQANSQKDEIFALINKEANKDARAIAYASNLKGIKRELS |
| Sequence105-NP-UD-Vit-ABD | NTTTKIEWKQKLKLTAKKIRFLHLSIKRLVRKQERPLLIRILKASGANCL |
| Sequence126-NP-UG-Jp-ABC | KSGNKNKMEAKAQANSQKDEIFALINKEANRDARAIAYAQNLKGIKRELS |
| Sequence135-P-CG-Col-ABCCC | KSGNKNKMEAKAQANSQKDEIFALINKEANRDARAIAYSQNLKGIKRELS |
| Sequence138-NP-GA-Col-ABC | KSGNKNKMEAKAQANSQKDEIFALINKEANRDARAIAYAQNLKGIKRELS |
| Sequence13-NP-UP-Jp-ABD | RNENKNRMEAKAQANSQKDKIFALINKEASKEARAASFDPNLKGIRSELS |
| Sequence11-NP-UP-Jp-ABD | RNDNKNRMEAKAQANSQKDKIFALINQEASKEARAAAFDPSFKGIRSELS |
| Sequence50-NP-GA-OCC-ABC | KSDNKNRMEAKAQANSQKDKIFAIINEEAGKEARVAACVQNLKGIRMELS |
| Sequence26-NP-GA-Jp-ABD | RNDNKNRMEVKAQANSQKDKIFALINQEASKEARAAAFDPNLKGIRSELS |
| Sequence55-NP-UD-OCC-ABCCC | KSDNKNRMEAKAQANSQKDKIFAIINEEAGKEARVVACVQNLKGIRMELS |
| Sequence178-NP-M-Col-ABCC | KSGNKNKMEAKAQANSQKNEIFALINKEANRDTRAIAYAQNLKGIKRELS |
| Sequence40-NP-GA-Jp-ABD | RNDNKNRMEAKAQANSQKDKIFALINQEASKEARAAAFDPNLKGIRSELS |
| Sequence43-NP-GA-Jp-ABD | RNDNKNRMEAKAQANSQKDKIFALINQEASKEARAAAFDPNLKGIRSELS |
| Sequence139-NP-UD-Col-ABCC | KSGNKNKMEAKAQANSQKDEIFALINKEANRDARAIAYAQNLKGIKRELS |
| Sequence7-P-CG-Jp-ABBD | RNENKNRMEAKAQANSQKDKIFALINKEASKEARAASFDPNLKGIRSELS |
| Sequence91-P-CG-Vit-ABD | RNDNKNRMEAKAQANSQKDKIFALINKEASKEARAAAFDPNLKGIRSELS |
| Sequence29-NP-GA-Jp-ABABD | RNNNKNRMEAKAQANSQKDKIFALINQEASKEARAAAFDPSLKGIRSELS |
| Sequence78-P-CG-EA-ABD | RNDNKNRMEAKAQANSQKDKIFALINQEASKEARAAAFDPNLKGIRNELS |
| Sequence188-NP-GCA-Jp-AB | KSGNKNKMEAKAQANSQKDEIFALINKEANRDARAIAYAQNLKGIKRELS |
| Sequence72-P-CG-EA-ABD | RNDNKNRMEAKAQANSQKDKIFALINKEASKEARAAAFDPNLKGIRSELS |
| Sequence79-P-CG-EA-ABD | RNDNKNRMEAKAQANSQKDKIFALINQEAGKEARAAAFDPSLKGIRSELS |
| Sequence61-NP-UD-OCC-ABC | RNDNKNRMEAKAQANSQKDKIFALINQEASKEARAAAFDPSLKGIRSELS |
| Sequence125-NP-UG-Jp-ABCC | KSGNKNKMEAKSQANSQKDGIFALINKEATREARAIAYAQNLKGIRRELS |
| Sequence175-NP-G-Col-ABCC | KSGNKNKMEAKAQANSQKDEIFALINKEANRDARAIAYSQNLKGIKRELS |
| Sequence122-NP-G-Jp-ABCC | KSGNKNKMEAKAQANSQKDEIFTLINKEANRDARAIAYAQNLKGIKRELS |
| Sequence36-NP-GA-Jp-ABD | RNDNKNRMEAKAQANSQKDKIFALINQEASKEARAAAFDPNLKGIRNELS |
| Sequence58-NP-UD-OCC-ABC | KSDNKNRMEAKAQANSQKDKIFAIINEEAGKEARVAACVQNLKGIRMELS |
| Sequence27-NP-GA-Jp-ABBD | RNDNKNRMEAKAQANSQKDKIFALINTEASKEARVAAFDPNLKSTRSELS |
| Sequence93-P-CG-Vit-ABD | RNDNKNRMEAKAQANSQKDKIFALINKEASKEARAAAFDPNLKGIRSELS |
| Sequence177-NP-M-Col-ABCC | KSGNKNKMEAKSQANSQKDEIFALINKEANRDARAIAYAQNLKGIKRELS |
| Sequence182-NP-UD-Col-ABCC | KSGNKNKMEAKSQANSQKDEIFALINKEANRDARAIAYAQNLKGIKRELS |
| Sequence114-NP-UG-Jp-ABC | KSGNKNKMEAKSQANSRKDEIFMLINKEANRDARAIAYAQNLKGIKRELS |
| Sequence74-P-CG-EA-ABD | RNDNKNRMEAKAQANSQKDKIFALINQEASKEARAAAFDPNLKGIRNELS |
| Sequence20-P-CG-Jp-ABD | RNDNKNRMEAKAQANSQKDKIFALINQEASKEARAAAFDPSLKGIRSELS |
| Sequence14-NP-GCA-Jp-ABD | RNDNKNRMEAKAQANSQKDKIFALINQEASKEARAAAFDPNLKGIRSELS |
| Sequence39-NP-GA-Jp-ABC | KSGNKNKMEAKSQANSQKDEIFALINKEANRDARAITYAQNLKGIKRELS |
| Sequence170-NP-UD-Col-ABCC | KSGNKNKMEAKAQANSQKDEIFALINKEANRDARAIAYAQNLKGIKRELS |
| Sequence76-P-CG-EA-ABD | RNDNKNRMEAKAQANSQKDKIFALINQEASKEARAAAFDPNLKGIRSELS |
| Sequence53-NP-GA-EA-ABD | SNDNKNRMEAKAQANSQKDKIFALINQEASKEARAAAFDPNLKGIRNELS |
| Sequence174-P-CG-Col-ABCC | KSGNKNKMEAKAQANSQKDEIFALINKEANRDARAIAYSQNLKGIKRELS |
| Sequence33-NP-UG-Jp-ABD | KSGNKNKMEAKAQANSQKDEIFMLINKEANRDARAIICAQNLKGIRRELS |
| Sequence60-NP-GA-EA-ABD | RNDNKNRMEAKAQANSQKDKIFALINQEASKEARVAAFDSSLKGIRSELS |
| Sequence38-NP-GA-Jp-ABD | RNDNKNRMEAKAQANSQKDKIFALINQEASKEARAVAFDPNLKGIRSELS |
| Sequence1-NP-EG-Jp-ABCCC | KSGNKNKMEAKAQANGQKDKIFALINKEANRDARAIAYSQNLKGIKRELS |
| Sequence101-P-CG-Vit-ABD | RYDNKNRMEAKAQAHSQKDKISALINKEASKEARAAAFDPNLKGVRSELS |
| Sequence64-NP-UD-OCC-ABC | KSDNKNRMEAKAQANSQKDKIFAIINEEAGKEARVAACVQNLKGIRMELS |
| Sequence81-P-CG-EA-ABD | RNGNKNRMEAKAQANSQKDKIFALINKEASKEARAVAFDPNLKGVRSELS |
| Sequence150-NP-DA-Sen-BC | KSNNKNKMEAKSQANSQKDEIFALINKEANREARAATYALNSKGIKRELS |
| Sequence90-NP-UD-Vit-ABD | RNDNKNRMEAKAQANSQKDKIFALINKEASKEARAAAFDPNLKGVRSELS |
| Sequence136-P-CG-Col-AACC | KSGNKNKMEAKAQANSQKDEIFALINKEANRDARAIAYSQNLKGIKRELS |
| Sequence15-NP-UP-Jp-ABD | RNDNKNRMEAKAQANSQKDKIFALINQEASKEARAAAFDPNLKGIRSELS |
| Sequence108-NP-UD-Vit-ABD | RIDNKNRMEAKAQANSQKDKIFALINKEASKEARAAAFDPNLKGIRSELS |
| Sequence104-P-CG-OCC-ABCC | KSNNKNKMEAKSQANSQKDEIFALINKEANKDARAIAYASNLKGIKRELS |
| Sequence179-NP-UD-Col-ABCC | KSGNKNKMEAKAQANSQKDEIFALINKEANRDARAIAYSQNLKGIKRELS |
| Sequence37-NP-GA-Jp-ABCCC | KSDNKNRMEAKAQANSQKDKIFAIINEEAGKEARVAACVQNLKGIRMELS |
| Sequence89-NP-UD-Vit-ABD | RNDNKNRMEAKAQANSQKDKIFALINKEASKEARAAAFDPNLKGVRSELS |

| | |
|---|---|
| Sequence92-P-CG-Vit-ABD | RNDNKNRMEAKAQANSQKDKIFALINKEASKEARAAAFDPNLKGIRSELS |
| Sequence49-NP-GA-Jp-ABC | RNGNKNRMEAKAQANSQKDKIFAIINEEAGKEARVAPCVQNLKGIRMELS |
| Sequence181-P-CG-Col-ABCC | KSGNKNKMEAKAQANSQKDEIFALINKEANRDARVIAYSQNLKGIKRELS |
| Sequence25-NP-GA-Jp-ABD | RNDNKNRMEAKAQANSQKDKIFALINQEASKEARATAFDPNLKGIRIELS |
| Sequence4-P-CG-Jp-ABD | RNDNKNRMEAKAQANSQKDKIFALISQEASKEARVATFDPYLKGVRSELS |
| Sequence121-NP-G-Jp-ABCC | KSGNKNKMEAKSQANSQKDEIFALINKEASREARAVAYASNLKGIKRELS |
| 4dvy | KSGNKNKMEAKAQANSQKDEIFALINKEANRDARAIAYTQNLKGIKRELS |
| Sequence186-NP-UP-Jp-ABD | RNDNKNRMEAKAQANSQKDKIFALINQEASKEARAAAFDPNLKGIRSELS |
| Sequence62-NP-UD-OCC-ABD | RNDNKNRMEAKAQANSQKDKIFAIINEEAGKEARAAACVQNLKGIRMELS |
| Sequence9-NP-GCA-Jp-ABD | RNDNKNRMEAKAQANSQKDKIFALINQEASKEARAVAFDPSLKGIRSELS |
| Sequence12-NP-UP-Jp-ABD | RNDNKNRMEAKTQANSQKDKIFALINQEASKEARATAFDPNLKGIRSELS |
| Sequence35-NP-GA-Jp-ABABD | RNDNKNRMEAKAQANSQKDKIFALINKEASKEARAAAFDPNLKGIRSELS |
| Sequence54-NP-GA-OCC-ABC | RNGNKNRMEAKAQANSQKDKIFAIINEEAGKEARAVACVQNLKGIRMELS |
| Sequence109-P-CG-Jp-ABD | RNDNKNRMEAKAQANSQKDKIFALINKEASKEARAAAFDPNLKGIRSELS |
| Sequence96-NP-UD-Vit-ABD | RNDNKNRMEAKAQANSQKDKIFALINQEASKEARAAAFDPNLKGVRRELS |
| Sequence80-P-CG-EA-ABD | RNDNKNRMEAKAQANSQKDKIFALINQEASKEARAAAFDPNLKGIRSELS |
| Sequence82-P-CG-EA-ABD | RNDNKNRMEAKAQANSQKDKIFALINKEASKEARAAAFDPNLKGIRNELS |
| Sequence10-NP-GCA-Jp-ABD | RNDNKNRMEAKAQANSQKDKIFALINQEASKEARAAAFDPSFKGIRSELS |
| Sequence19-NP-UP-Jp-ABBD | RNDNKNRMEAKAQANSQKDKIFALINQEASKEARAATFDPSLKGIRSELS |
| Sequence115-NP-UG-Jp-ABC | KSGNKNKMEAKSQANSQKDKIFALINKEANRDARAITYAQNFKGIGRELS |
| Sequence45-NP-GA-Jp-ABCC | KSGNKNKMEAKSQANSQKDGIFMLINKEANRDARAIAYAQNLKGIKRELS |
| Sequence119-NP-UG-Jp-ABC | KSDNKNKMEAKSQANSQKDEIFALINKEASREARAVIYASNLKGIKRELS |
| Sequence70-NP-GA-Ch-ABD | RNDNKNRMEAKAQANSQKDKIFALINQEASKEARAA-------------- |
| Sequence52-NP-UD-EA-ABD | RNDSKNRMEAKAQANSQKDKIFMLINQEASKEARAAAFDPNLKGIRNELS |
| Sequence97-P-CG-Vit-ABD | RDDNKNRMEAKAQAHSQKDKISALINKEASKEARSAAFDPNLKGIRSELS |
| Sequence94-NP-UD-Vit-ABD | RNDNKNRMEAKAQANSQKDKIFALINKEASKEARVAAFDPNLKGIRSELS |
| Sequence176-NP-GA-Col-ABCC | KSGNKNKIEAKSQANSQKDEIFALINKEANRDARAIAYSQNLKGIKRELS |
| Sequence68-NP-D-Am-ABBC | KSGNKNKMEAKAQANSQKDEIFALINKEANRDARAIAYTQNLKGIKRELS |
| Sequence47-NP-GA-Jp-ABD | RNDNKNRMEAKAQANSQKDKIFALINQEASKEARAAAFDPNLKGIRSELF |
| Sequence56-NP-UD-OCC-ABC | KSDNKNRMEAKAQANSQKDKIFAIINEEAGKEARVAACVQNLKGIRMELS |
| Sequence31-NP-GA-Jp-ABD | RNDNKNRMEAKAQANSQKDKIFALINQEASKEARATAFDPNLKGVRGELS |
| Sequence46-NP-UD-Jp-ABD | RNDNKNRMEAKAQANSQKDKIFALINQEASKEARAVAFDQNLKSIRSELF |
| Sequence102-NP-UG-Vit-ABD | RNDNKNRMEAKAQANSQKDKISALINKEASKEARAAAFDPNLKGVRSELS |
| Sequence71-P-CG-EA-ABD | RNDNKNRMEAKAQANSQKDKIFALINQEASKEARAAAFDPNLKGIRNELS |
| Sequence30-NP-GA-Jp-ABBD | RNDNKNRMEAKAQANSQKDKIFALINKEASKEARAAAFDPNLKGIRSELF |
| Sequence28-NP-GA-Jp-AABD | RNDNKNRMEAKAQANSQKDKIFALINQEASKEARAAAFDPNLKGIRSELS |
| Sequence98-P-CG-Vit-AD | RNDNKNRMEAKAQANSQKDKIFALINKEASKEARAVAFDPNLKGIRSELS |
| Sequence99-P-CG-Vit-ABD | RNDNKNRMEAQAQANSQKDKIFALINKEASKEARAVAFDPNLKGIRSELS |
| Sequence24-NP-UD-USA-ABD | RNDNQNRMEAKAQANSQKDKIFALINKEASKEARAVAFDPNLKGIRSELS |
| Sequence124-NP-G-Jp-ABC | KSGNKNKMEAKAQANSQKDEIFALINKEANRDARAIAYAQNLKGIERELS |
| Sequence77-P-CG-OCC-ABCC | RNGNKNRMEAKAQANSQKDKIFAIINEEAGKEARGAACVQNLKSIRMELS |
| Sequence113-NP-UG-Jp-ABC | KSGNKNKMEAKSQANSRKDEIFMLINKEANRDARAIAYAQNLKGIKRELS |
| Sequence88-NP-UD-Vit-ABD | RNDNKNRMEAKAQANSQKDKIFALINKEASKEARAAAFDPNLKGIRSELS |
| Sequence100-NP-UD-Vit-ABD | RNDNKNRMEAKAQANSQKDKIFALINKEASKEARAAAFDPNLKGIRSELS |
| Sequence137-NP-G-Col-ABCC | KSGNKNKMEAKAQANSQKDEIFALINKEANRDARAIAYSQNLKGIKRELS |
| Sequence117-NP-G-Jp-ABC | KSGNKNKMEAKSQANSQKDKIFALINKEATREARAAIYASNLKGIKRELS |
| Sequence171-NP-UD-Col-ABCC | KSGNKNKMEAKAQANSQKDEIFALINKEANRDARAIAYSQNLKGIKRELS |
| Sequence180-P-CG-Col-ABC | KSGNKNKMEAKAQANSQKDKIFALINKEANRDARAIAYAQNLKGIKRELS |
| Sequence112-P-CG-Jp-ABCC | KSGNKNKMEAKSQANSQKDEIFALINKEATREARAATYASNLKGIKRELS |
| Sequence127-NP-UD-Jp-ABC | KSGNKNKMEAKSQANSQKDEIFAFINKEANREARAAAYASNLKGIKRELS |
| Sequence51-NP-GA-OCC-ABC | KSGNKNKMEAKAQANSQKDKIFAIINKEASREARAATYTQSLKIIRRELH |
| Sequence73-P-CG-EA-ABD | RNDNKNRMETKAQANSQKDKIFALINQEASKEARAAAFDPSLKGIRIELS |
| Sequence183-NP-UD-Col-ABD | KSGNKNKMEAKAQANSKKDEIFALINKEANRDARAIAYSQNLKGIKRELS |
| Sequence34-NP-UD-Jp-ABDD | RNDNKNRMEAKAQANSQKDKIFALINQEASKEARAAAFDPSLKGIRSELS |
| Sequence21-P-CG-Jp-ABD | RNDNKNRMEAKAQANSQKDKIFALINQEASKEARAAAFDPNLKGIRSELS |
| Sequence32-NP-GA-Jp-ABD | RNENKNRMEAKAQANSQKDKIFALINQEASKEARAAAFDPNLKGVRSELS |
| Sequence116-NP-UD-Jp-ABC | KSGNKNKMEAKSQANSQKDKIFALINKEANRDARAITYAQNFKGIGRELS |
| Sequence8-NP-GCA-Jp-ABD | RNDNKNRMEAKAQANSQKDKIFALINTEASKEARVVAFDPNLKGIRSELS |
| Sequence83-P-CG-EA-ABD | RNDNKNRMEAKAQANSQKDKIFALINQEASKEARAAAFDPNLKGVRSELS |
| Sequence48-NP-UD-OCC-ABC | KSDNKNRMEAKAQANSQKDKIFAIINEEAGKEARVAACVQNLKGIRMELS |
| Sequence120-NP-G-Jp-ABC | KSGNKNKMEAKAQANSQKDGIFALINKEANRDARAIAYSQNLKGIKRELS |

```
Sequence187-NP-UG-Jp-ABCCC      KSGNKNKMEAKAQANSQKDKIFALINKEANRDARAIAYSQNLKGIKRELS
Sequence118-NP-G-Jp-ABD         RNDNKNRMEAKAQANSQKDKIFALINKEASKEARAAAFDPNLKGIRSELS
Sequence87-NP-UG-Vit-ABD        RNDNKNRMEAKAQANSQKDKIFALINKEASKEARVAAFDPNLKGVRSELS

Sequence140-NP-GA-Col-ABABC     DKLENINKDLKDFNKSFDEFKNGKNKDFSKAEETLKALKGSVKDLGINP-
Sequence123-NP-UD-Jp-ABD        DKLENINKNLKDFGKSFDELKNGKNNDFSKAEETLKALKDSVKDLGINP-
Sequence111-NP-G-Jp-ABD         DKLENINKNLKDFGKSFDELKNGKNKDFSKAEETLKALKDSVKDLGINP-
Sequence57-NP-GA-OCC-AB         DKLENINKNLKDFDKSFDELKNGKNNDFSKAEETLKALKDSVKDLGINP-
Sequence172-P-CG-Col-ABCC       DKLENINKNLKDFSKSFDEFKNGKNKDFSKAEETLKALKGSVKDLGINP-
Sequence110-NP-UD-Jp-ABD        DKLENINKNLKDFGKSFDELKNGKNNDFSKAEETLKALKDSVKDLGINP-
Sequence95-NP-UD-Vit-ABD        DKLENINKNLKDFGKSFDELKNGKNNDFSKAEETLKALKDSVKDLGINP-
Sequence107-P-CG-Vit-ABD        DKLENINKNLKDFGKSFDELKNGKNNDFSKAEETLKALKDSVKDLGINP-
Sequence106-P-CG-Vit-ABD        DKLENVNKNLKDFSKSFDELKNGKNKDFSKAEETLKTLKDSVKDLGINP-
Sequence105-NP-UD-Vit-ABD       INLKISTRNLKDFGKSFDELKNGNNKDFSKAEETLKALKDSVKDLGINL-
Sequence126-NP-UG-Jp-ABC        GKLEKINKDLKDFSKSFDEFKNGKNKDFSKAEETLKALKGSVKDLGINP-
Sequence135-P-CG-Col-ABCCC      DKLENVNKDLKDFSKSFDDFKNGKNKDFSKAEETLKALKGSVKDLGINP-
Sequence138-NP-GA-Col-ABC       DKLENINKDLRNFSKSFDEFKNGKNKDFSKAEETLKALKGSVKDLGINP-
Sequence13-NP-UP-Jp-ABD         DKLENINKNLKDFGKSFDELKNGKNNDFSKVEDTFKALKDSVRDLGINP-
Sequence11-NP-UP-Jp-ABD         DKLENINKNLKDLGKSLDELKNGKNNDFSKAEETLKALKDSVKDLGINP-
Sequence50-NP-GA-OCC-ABC        DKLENINKNLKDFDKSFDEFKNGKNKDFSKTEETLKALKDSVKDLGINP-
Sequence26-NP-GA-Jp-ABD         DKLENINKNLKDFGKSFDELKNSKNKDFSKAEETLKALKDSVKDLGINP-
Sequence55-NP-UD-OCC-ABCCC      DKLENINKNLKDFDKSFDEFKNGKNKDFSKTEETLKALKDSVKDLGINP-
Sequence178-NP-M-Col-ABCC       DKLENINKDLRNFSKSFDEFKNGKNKDFSKAEETLKALKGSVKDLGINP-
Sequence40-NP-GA-Jp-ABD         DKLENINKNLKDFGKSFDELKNGKNNDFSKVEETLKALKDSVKDLGINP-
Sequence43-NP-GA-Jp-ABD         DKLANINKNLKDFGKSFDELKNGKNNDFSKAEETLKALKDSVKDLGINP-
Sequence139-NP-UD-Col-ABCC      DKLENINKDLKDFSKSFDEFKNGKNKDFSKAEETLKALKGSVKDLGINP-
Sequence7-P-CG-Jp-ABBD          DKLENINKNLKDFGKSFDELKNGKNNDFSKAEETLKALKDSVKDLGINP-
Sequence91-P-CG-Vit-ABD         DKLENINKNLKDLWQSFDELKNGKNNDFSKAEETLKALKDSVKDLGINP-
Sequence29-NP-GA-Jp-ABABD       DKLENINKNLKDFGKSFDELKNGKNNDFSKAEETLKALKDSVKDLAINP-
Sequence78-P-CG-EA-ABD          DKLENINKNLKDFGKSFDELKNGKNNDFNKAEETLKALKDLVKDLGINP-
Sequence188-NP-GCA-Jp-AB        DKLENVNKNLKDFDKSFDEFKNGKNKDFSKAEETLKALKGSVKDLGINP-
Sequence72-P-CG-EA-ABD          DKLENINKNLKDFGKSFDELKNGKNKDFSKAEETLKALKNSVKDLGINP-
Sequence79-P-CG-EA-ABD          DKLENINKNLKDFGKSFDELKNGKNNDFSKAEETLKALKDLVKDLGINP-
Sequence61-NP-UD-OCC-ABC        NKLENINKNLKDFGKSFDELKNGKNKDFSKAEETLKALKDLVKDLGINP-
Sequence125-NP-UG-Jp-ABCC       DKLENVNKDLKDFSKSFDEFKNGKNKDFSKAEETLKALKGSVKDLGINP-
Sequence175-NP-G-Col-ABCC       DKLEKINKDLKDFSKSFDEFKNGKNKDFSKTEETLKALKGSVKDLGINP-
Sequence122-NP-G-Jp-ABCC        DKLENVNKNLKDFSKSFDEFKNGKNKDFSKTEETLKALKGSVKDLGINP-
Sequence36-NP-GA-Jp-ABD         DKLENINKNLKDFGKSFDELKNGKNNDFNKAEETLKALKDSVKDLGINP-
Sequence58-NP-UD-OCC-ABC        DKLENINKNLKDFDKSFDEFKNGKNKDFSKTEETLKALKDSVKDLGINP-
Sequence27-NP-GA-Jp-ABBD        DKLENINKNLKDFGKSFDELKNGKNNDFSKAEETLKALKDSVKDLGINP-
Sequence93-P-CG-Vit-ABD         DKLENINKNLKDFGKSFDELKNGNNKDFSKAEETLKALKDSVKDLGINP-
Sequence177-NP-M-Col-ABCC       DKLENVNKNLKDFSKSFDEFKNGKNKDFSKAEETLKALKGSVKDLGINP-
Sequence182-NP-UD-Col-ABCC      DKLENINKNLKDFSKSFDEFKNGKNKDFSKAEETLKALKGSVKDLGINP-
Sequence114-NP-UG-Jp-ABC        DKLENVNKNLKDFSKSFDEFKNGKNKDFSKAEETLKALKGSVKDLGINP-
Sequence74-P-CG-EA-ABD          DKLENINKNLKDFGKSFDELKNGKNNDSSKAEETLKALKDSVKDLGINP-
Sequence20-P-CG-Jp-ABD          DKLENINKNLKDFGKSFDELKNGKNNDFSKAEETLKALKDSVKDLGINP-
Sequence14-NP-GCA-Jp-ABD        DKLENINKNLKDFGKSFDELKNGKNNDFSKAEETLKALKDLVKDLNINP-
Sequence39-NP-GA-Jp-ABC         DKLENINKDLKDFSKSFDGFKNGKNKDFSKAEETLKALKGSVKDLGINP-
Sequence170-NP-UD-Col-ABCC      DKLENVNKDLKGFSKSFDEFKNGKNKDFSKAEETLKALKGSVKDLGINP-
Sequence76-P-CG-EA-ABD          DKLENINKNLKDFGKSFDELKNGKNNDFSKVEETLKALKDSVKDLGINP-
Sequence53-NP-GA-EA-ABD         DKLENINKNLKDFGKSFDELKNGKNNDFNKAEETLKALKDSVKDLGINP-
Sequence174-P-CG-Col-ABCC       DKLENINKDLKDLSKSFDEFKNGKNKDFSKAEETLKALKGSVKDLGINP-
Sequence33-NP-UG-Jp-ABD         DKLENVNKDLKDFSKSFDEFKNGKNKDFSKAEETLKALKGSVKDLGINP-
Sequence60-NP-GA-EA-ABD         DKLENINKNLKDFGKSFDELKNGKNNDFSKAEETLKALKDSVKDLGINP-
Sequence38-NP-GA-Jp-ABD         DKLENINKNLKDFGKSFDELKNGKNNDFSKAEETLKALKDSVKDLGINP-
Sequence1-NP-EG-Jp-ABCCC        DKLEKINTDLKDFSKSFDEFKNGKNKDFSKAEETLKALKGSVKDLGINP-
Sequence101-P-CG-Vit-ABD        DKLENITKNLKDFGKSFDELKNGKNKDFSKAEETLKALKDSVKDLGINP-
Sequence64-NP-UD-OCC-ABC        DKLENINKNLKDFDKSFDEFKNGKNKDFSKTEETLKALKDSVKDLGINP-
Sequence81-P-CG-EA-ABD          DKLENINKNLNAFNKSFDELKNGKNNDFSKAEETLKALKDSVKDLGINP-
Sequence150-NP-DA-Sen-BC        DKLENVNKNLKDFSKSFDEFKNGKNKDFSKAEETLKALKGSVKDLGINP-
```

| | |
|---|---|
| Sequence90-NP-UD-Vit-ABD | DKLENINKNLKDFGKSFDELKNGNNNDFSKAEETLKALQDSVKDLGINP- |
| Sequence136-P-CG-Col-AACC | DKLENINKDLKDFSKSFDEFKNGKNKDFSKAEETLKALKGSVKDLGINP- |
| Sequence15-NP-UP-Jp-ABD | DKLENINKNLKDFGKSFDELKNGKNNDFSKAEETLKALKDLVKDLGINP- |
| Sequence108-NP-UD-Vit-ABD | DKLENINKNLKDFGKSFDELKNGKNNDFSKAEETLKALKDSVKDLGINP- |
| Sequence104-P-CG-OCC-ABCC | DKLENVNKNLKDFSKSFDEFKNGKNKDFSKAEETLKALKGLVKDLGINP- |
| Sequence179-NP-UD-Col-ABCC | DKLENINKDLRNFSKSFDEFKNGKNKDFSKAEETLKALKGSVKDLGINP- |
| Sequence37-NP-GA-Jp-ABCCC | DKLENINKNLKDFDKSFDEFKNGKNKDFSKTEETLKALKDSVKDLGINP- |
| Sequence89-NP-UD-Vit-ABD | DKLENINKNLKDFGKSFDELKNGKNNDFSKAEETLNALKDSVKDLGINP- |
| Sequence92-P-CG-Vit-ABD | DKLENINKNLKDFGKSFDELKNGKNNDFSKAEETLKALKDSVKDLGINP- |
| Sequence49-NP-GA-Jp-ABC | DKLENINKNLKDFDKSFDEFKNGKNKDFSKTEETLKALKDSVKDLGINP- |
| Sequence181-P-CG-Col-ABCC | DKLENINKDLKDFSKSFDEFKNGKNKDFSKAEETLKALKGSVKDLGINP- |
| Sequence25-NP-GA-Jp-ABD | DKLENINKNLKDFGKSFDELKNGKNNDFSKAEETLKALKDSVKDLGINP- |
| Sequence4-P-CG-Jp-ABD | DKLENINKNLKDFGKSFDELKSGKNNDFSKAEETLKALKDSVKDLGINP- |
| Sequence121-NP-G-Jp-ABCC | DKLENVNKNLKDFSKSFDEFKNGKNKDFSKAEETLKALKGSVKDLGINP- |
| 4dvy | DKLEKISKDLKDFSKSFDEFKNGKNKDFSKAEETLKALKGSVKDLGINP- |
| Sequence186-NP-UP-Jp-ABD | DKLENINKNLKDFGKSFDELKSGKNKDFSKAEETLKALKDSVKDLGINP- |
| Sequence62-NP-UD-OCC-ABD | DKLENINKNLKDFDKSFDEFKNGKNKDFSKAEETLKALKDSVKDLGINP- |
| Sequence9-NP-GCA-Jp-ABD | DKLENINKNLKDFGKSFDELKNGKNNDFSKAEETLKALKDSVKDLAINP- |
| Sequence12-NP-UP-Jp-ABD | DKLENINKNLKDFGKSLDELKNGKNNDFSKVEETLKALKDSVKDLGINP- |
| Sequence35-NP-GA-Jp-ABABD | DKLENINKNLKDFGKSFDELKNGKNNDFSKAEETLKALKDSVKDLGINP- |
| Sequence54-NP-GA-OCC-ABC | DKLENINKNLKDFDKSFDEFKNGKNKDFSKTEETLKALKSLVKDSGISL- |
| Sequence109-P-CG-Jp-ABD | DKLENINKNLKDFGKSFDELKNGKNNDFSKAEETLKALKDSVKDLGINP- |
| Sequence96-NP-UD-Vit-ABD | DKLENISKNLKDFGKSFDELKNGKNNDFSKAEETLKALKDSVKDLGINP- |
| Sequence80-P-CG-EA-ABD | DKLENINKNLKDFGKSFDELKNGKNNDFSKVEETLKALKDSVKDLGINP- |
| Sequence82-P-CG-EA-ABD | DKLENINKNLKDFGKSFDELKNGKNNDFNKAEETLKALKDSVKDLGINP- |
| Sequence10-NP-GCA-Jp-ABD | DKLENINKNLKDFGKSFDELKNGKNNDFSKAEETLKALKDSVKDLGINP- |
| Sequence19-NP-UP-Jp-ABBD | DKLENINKNLKDFGKSFDELKNG-NNDFSKAEETLKALKDSVKDLAINP- |
| Sequence115-NP-UG-Jp-ABC | DKLENINKDLKDFDKSFDEFKNGKNKDFSKAEETLKALKGSVKDLGINP- |
| Sequence45-NP-GA-Jp-ABCC | DKLENVNKNLKDFSKSFDEFKNGKNKDFSKAEETLKALKGSVKDLGINP- |
| Sequence119-NP-UG-Jp-ABC | DKLENVNKNLKDFNKSFDEFKNGKNKDFSKAEETLKALKGSVKDLGISP- |
| Sequence70-NP-GA-Ch-ABD | -------------------------------------------------- |
| Sequence52-NP-UD-EA-ABD | DKLENINKNLKDFGKSFDELKNGKNNDFNKAEETLKALKDSVKDLGINP- |
| Sequence97-P-CG-Vit-ABD | DKLENINKNLKVFGKSFDELKNGTNSDFSKAEETLKALKDSVKDLGINP- |
| Sequence94-NP-UD-Vit-ABD | DKLENIDKNLKDFGQSFDELKNGKNNDFSKAEETLKALKDSVKDLGINP- |
| Sequence176-NP-GA-Col-ABCC | DKLENINKDLKDFSKSFDEFKNGKNKDFSKAEETLKALKGSVKDLGINP- |
| Sequence68-NP-D-Am-ABBC | DKLENVNKNLKDFSKSFDEFKNGKNKDFSKAEETLKALKGSVKDLGINP- |
| Sequence47-NP-GA-Jp-ABD | DKLENINKDLKDFGKSFDELKNGKNNDFNKAEETLKALKDSVKDLGINP- |
| Sequence56-NP-UD-OCC-ABC | DKLENINKNLKDFDKSFDEFKNGKNKDFSKTEETLKALKDSVKDLGINP- |
| Sequence31-NP-GA-Jp-ABD | DKLENINKNLKDFGKSFDELKSGKNKDFSKAEETLKALKDSVKDLGINP- |
| Sequence46-NP-UD-Jp-ABD | DKLENINKNLKNFGKSFDELKNGKNNDFSKAEETLKALKDSVKDLGINP- |
| Sequence102-NP-UG-Vit-ABD | DKLETINKNLKDFGNSFDELKNGKNKDFSKAEETLKALKDSVKDLGINP- |
| Sequence71-P-CG-EA-ABD | DKLENINKNLKDFGKSFDELKNGKNNDFNKAEETLKALKDSVKDLGINP- |
| Sequence30-NP-GA-Jp-ABBD | DKLENINKNLKDFGKSFDELKNGKNNDFSKAEETLKALKDLVKDLAINP- |
| Sequence28-NP-GA-Jp-AABD | DKLENINKNLKDFGKSFDELKNGKNNDFSKAEETLKALKDSVKDLGINP- |
| Sequence98-P-CG-Vit-AD | DKLENINKNLKDFGKSFDELKNGKNKDFSKAEETLKALKDSVKDLGIQSR |
| Sequence99-P-CG-Vit-ABD | DKLANINKNLKDFGKSFDELKNGKNNDFSKAEETLKALKDSVKDLGINP- |
| Sequence24-NP-UD-USA-ABD | DKLENINKNLKDFGKSFDELKNGKNNDFSKAEETLKALKDSVKDLGINP- |
| Sequence124-NP-G-Jp-ABC | DKLEKINKDLKDFSKSFDEFKNGKNKDFSKAEETLKALKGSVKDLGINP- |
| Sequence77-P-CG-OCC-ABCC | DKLENINKNLKDLDKSFDEFKNGKNKDFSKTEETLKALKDSVKDLGINP- |
| Sequence113-NP-UG-Jp-ABC | DKLENVNKNLKDFSKSFDEFKNGKNKDFSKAEETLKALKGSVKDLGINP- |
| Sequence88-NP-UD-Vit-ABD | DKLENINKNLKDLWQSFDELKNGKNNDFSKAEETLKALKDSVKDLGINP- |
| Sequence100-NP-UD-Vit-ABD | DKLENINKNLKDFGKSFDELKNGKNNDFSKVEETLKALKDSVKDLGINP- |
| Sequence137-NP-G-Col-ABCC | DKLENINKDLKDFSKSFDEFKNGKNKDFSKAEETLKALKGSVKDLGINP- |
| Sequence117-NP-G-Jp-ABC | DKLENINKNLKGFDKSFDEFKNGKNKDFSKAEETLKALKGSVKDLGINP- |
| Sequence171-NP-UD-Col-ABCC | DKLENINKDLRNFSKSFDEFKNGKNKDFSKAEETLKALKGSVKDLGINP- |
| Sequence180-P-CG-Col-ABC | DKLENINKNLKDLSKSFDEFKNGKNKDFSKAEETLKALKGSVKDLGINP- |
| Sequence112-P-CG-Jp-ABCC | DKLENINKNLKGFDKSFDEFKNGKNKDFSKAEETLKALKGSVKDLGINP- |
| Sequence127-NP-UD-Jp-ABC | DKLENVNKDLKDFSKSFDEFKNGKNKDFSKAEETLKALKGSVKDLGINP- |
| Sequence51-NP-GA-OCC-ABC | DKLENINKNLNAFDKSFDEFKNGKNKDFSKAEETLKALKDSVKDLGINP- |
| Sequence73-P-CG-EA-ABD | DKLENINKNLKDFGKSFDELKNGKNNDFSKTEETLKALKDSVEDLGINP- |
| Sequence183-NP-UD-Col-ABD | DKLENVNKDLKDFSKSFDEFKNGKNKDFSKAEETLKALKGSVKDLGINP- |

```
Sequence34-NP-UD-Jp-ABDD        DKLENINKNLKDFGKSFDELKNGKNNDFSKAEETLKVLKDSVKDLAINP-
Sequence21-P-CG-Jp-ABD          DKLENINKNLKDFGKSFDELKSGKNNDFSKAEETLKALKDSVKDLGINP-
Sequence32-NP-GA-Jp-ABD         DKLENINKNLKDFGKSFDDFKNGKNNDFSKAEETLKALKDSVKDLGINP-
Sequence116-NP-UD-Jp-ABC        DKLENINKDLKDFDKSFDEFKNGKNKDFSKAEETLKALKGSVKDLGINP-
Sequence8-NP-GCA-Jp-ABD         DKLENINKNLKDFGKSFDELKNGKNNDFSKAEETLKALKDSVKDLGINP-
Sequence83-P-CG-EA-ABD          DKLENINKNLKDFGKSFDDFKNGKNNDFSKAEETLKALKDSVKDLGINP-
Sequence48-NP-UD-OCC-ABC        DKLENINKNLKDFDKSFDEFKNGKNKDFSKTEETLKALKDSVKDLGINP-
Sequence120-NP-G-Jp-ABC         DKLENVNKNLKDFSKSFDEFKNGKNKDFSKAEETLKALKGSVKDLGINP-
Sequence187-NP-UG-Jp-ABCCC      DKLEKINKDLKDFSKSFDEFKNGKNKDFSKAEETLKALKGSVKDLGINP-
Sequence118-NP-G-Jp-ABD         DKLENINKNLKDFGKSFDDFKNGKNNDFSKAEETLKALKDSVKDLGINP-
Sequence87-NP-UG-Vit-ABD        DKLENINKNLKDFGKSFDELKNDKNKDFSKAEETLKALKDSVKDLGINP-

                                751                                            800
Sequence140-NP-GA-Col-ABABC     EWISKVENLNAALNDFKNGKNKDLSK-VTQAKSDLENSIKDV-IINQKIT
Sequence123-NP-UD-Jp-ABD        EWISKIENLNAALNDFKNGKNKDFSK-VTQAKSDLENSIKDV-IINQKIT
Sequence111-NP-G-Jp-ABD         EWISKIENLNAALNDFKNGKNNDFSK-VTQAKSDLENSIKDV-IINQKIT
Sequence57-NP-GA-OCC-AB         EWISKIENLNAALNDFKNGKNKDFSK-VTQAKSDLENSIKDV-IINQEIT
Sequence172-P-CG-Col-ABCC       EWISKVENLNAALNDFKNGKNKDFSK-VTQAKSDLENSVKDV-IINQKIT
Sequence110-NP-UD-Jp-ABD        EWISKIENLNAALNDFKNGKNNDFSK-VTQAKSDLENSIKDV-IINQKIT
Sequence95-NP-UD-Vit-ABD        EWISKIENLNAALNDFKNGKNNDFSK-VTQAKSDLENSIKDV-IINQKIT
Sequence107-P-CG-Vit-ABD        EWISKIENLNAALNDFKNGKNKDFSK-VTQAKSDLENSIKDV-IINQKIT
Sequence106-P-CG-Vit-ABD        EWISKIENLNAALNDFKNGKNKDFSK-VTQAKSDLENSIKDV-IINQKIT
Sequence105-NP-UD-Vit-ABD       EWISKIENLNAALNDFKNGKNKDFSK-VTQAKNDLENSIKDV-IINQKIT
Sequence126-NP-UG-Jp-ABC        EWISKVENLNAALNEFKNGKNKDFSK-VTQAKSDLENSVKDV-IINQKVT
Sequence135-P-CG-Col-ABCCC      EWISKVENLNAALNDFKNGKNKDFSK-VTQAKSDLENSVKDV-IINQKIT
Sequence138-NP-GA-Col-ABC       EWISKVENLNAALNDFKNGKNKDFSK-VTQAKSDLENSIKDA-IFNQKIT
Sequence13-NP-UP-Jp-ABD         EWISIIEYLNAALNDFKNGKNKDFSK-VTQAKSDLENSIKDV-IINQKIT
Sequence11-NP-UP-Jp-ABD         EWISKIENLNAALNDFKNGKNKDFSK-VTQAKSDLENSIKDV-IINQKIT
Sequence50-NP-GA-OCC-ABC        EWISKVENLNTALNEFKNGKNKDFSK-VTQAKSDLENSIKDV-IINQKIT
Sequence26-NP-GA-Jp-ABD         EWISKIENLNAALNDFKNSKNKDFSK-VTQAKSDLENSIKDV-IINQKIT
Sequence55-NP-UD-OCC-ABCCC      EWISKVENLNTALNEFKNGKNKDFSK-VTQAKSDLENSIKDV-IINQKIT
Sequence178-NP-M-Col-ABCC       EWISKVENLNAALNDFKNGKNKDLSK-VTQAKSDLENSVKDV-IIDQKIT
Sequence40-NP-GA-Jp-ABD         EWISKIENLNAALNDFKNGKNKDFSK-VTQAKSDLENSIKDV-IINQKIT
Sequence43-NP-GA-Jp-ABD         EWISKIENLNAALNDFKNGKNKDFSK-VTQAKSDLENSIKDV-IINQKIT
Sequence139-NP-UD-Col-ABCC      EWISKVENLNAALNDFKNGKNKDFSK-VTQAKSDLENSVKDV-IINQKIT
Sequence7-P-CG-Jp-ABBD          EWISKIENLNAALNDFKNGKNKDFSK-VTQAKSDLENSIKDV-IINQKIT
Sequence91-P-CG-Vit-ABD         EWISKIEDLNAALNDFKNGKNSDFSK-VTQAKSDLENSIKDV-IINQKIT
Sequence29-NP-GA-Jp-ABABD       EWISKIENLNAALNDFKNGKNKDFSK-VTQAKSDLENSIKDV-IINQKIT
Sequence78-P-CG-EA-ABD          EWISKIENLNAALNDFKNGKNKDFSK-VTQAKSDLENSIKDV-IINQKIT
Sequence188-NP-GCA-Jp-AB        EWISKVENLNAALNEFKNGKNKDFSK-VTQAKSDLENSVKDV-IINQKVT
Sequence72-P-CG-EA-ABD          EWISKIENLNAALNDFKNGKNKDFSK-VTQAKSDLENSIKDV-IINQKIT
Sequence79-P-CG-EA-ABD          EWISKIENLNAALNDFKNGKNKDFSK-VTQAKSDLENSIKDV-IINQKIT
Sequence61-NP-UD-OCC-ABC        EWISKIENLNAALNDFKNGKNKDFSK-VTQAKSDLENSIKDV-IINQKIT
Sequence125-NP-UG-Jp-ABCC       EWISKVENLNAALNDFKNSKNKDFSK-VTQAKSDLENSIKDV-IINQKIT
Sequence175-NP-G-Col-ABCC       EWISKVENLNAALNDFKNGKNRDFSK-VTQAKSDLENSVKDA-IFNQKIT
Sequence122-NP-G-Jp-ABCC        EWISKVENLNAALNDFKNGKNKDFSK-VTQAKSDLENSIKDA-IFNQKIT
Sequence36-NP-GA-Jp-ABD         EWISKIENLNAALNDFKNGKNKDFSK-VTQAKSDLENSIKDV-IINQKIT
Sequence58-NP-UD-OCC-ABC        EWISKVENLNTALNEFKNGKNKDFSK-VTQAKSDLENSIKDV-IINQKIT
Sequence27-NP-GA-Jp-ABBD        EWISKIENLNAALNDFKNGKNKDFSK-VTQAKSDLENSIKDV-IINQKIT
Sequence93-P-CG-Vit-ABD         EWISKIENLNAALNDFKNGKDSDFSK-VTQAKSDLENSIKDV-IINQKIA
Sequence177-NP-M-Col-ABCC       EWISKVENLNAALNDFKNGKNKDFSK-VTQAKSDLENSVKDV-IINQKVT
Sequence182-NP-UD-Col-ABCC      EWISKVENLNAALNDFKNGKNKDFSK-VTQAKSDLENSIKDA-IFNQKIT
Sequence114-NP-UG-Jp-ABC        EWISKVENLNAALNDFKNGKNKDFSK-VTQAKSDLENSIKDA-IFNQKIT
Sequence74-P-CG-EA-ABD          EWISKIENLNAALNDFKNGKNKDFSK-VTQAKSDLENSIKDV-IINQKIT
Sequence20-P-CG-Jp-ABD          EWISKIENLNAALNDFKNGKNKDFSK-VTQAKSDLENSIKDV-IINQKIT
Sequence14-NP-GCA-Jp-ABD        EWISKIENLNAALNDFKNGKNKDFSK-VTQAKSDLENSIKDV-IINQKIT
Sequence39-NP-GA-Jp-ABC         EWISKVENLNAALNEFKNGKNKDFSK-VTQAKSDLENSIKDA-IINQKIT
Sequence170-NP-UD-Col-ABCC      EWISKVENLNAALNDFKNGKNKDLSK-VTQAKSDLENSIKDV-IFNQKIT
Sequence76-P-CG-EA-ABD          EWISKIENLNAALNDFKNGKNKDFSK-VTQAKSDLENSIKDV-IINQKIT
Sequence53-NP-GA-EA-ABD         EWISKIENLNAALNDFKNGKNKDFSK-VTQAKSDLENSIKDV-IINQKIT
Sequence174-P-CG-Col-ABCC       EWISKVENLNAALNDFKNGKNKDFSK-VTQAKSDLENSVKDV-IINQKVT
```

| | | | | | | | | | | | | | | | | | | | | | | | | | | | | | | | | | | | | | | | | | | | | | | | | | | |
|---|---|---|---|---|---|---|---|---|---|---|---|---|---|---|---|---|---|---|---|---|---|---|---|---|---|---|---|---|---|---|---|---|---|---|---|---|---|---|---|---|---|---|---|---|---|---|---|---|---|---|
| Sequence33-NP-UG-Jp-ABD | E | W | I | S | K | V | E | N | L | N | A | A | L | N | E | F | K | N | G | K | N | K | D | F | S | K | - | V | T | Q | A | K | S | D | L | E | N | S | I | K | D | A | - | I | F | N | Q | K | I | T |
| Sequence60-NP-GA-EA-ABD | E | W | I | S | K | I | E | N | L | N | A | A | L | N | D | F | K | N | G | K | N | K | D | F | S | K | - | V | T | Q | A | K | S | D | L | E | N | S | I | K | D | V | - | I | I | N | Q | K | I | T |
| Sequence38-NP-GA-Jp-ABD | E | W | I | S | K | I | E | N | L | N | A | A | L | N | D | F | K | N | G | K | N | K | D | F | S | K | - | V | T | Q | A | K | S | D | L | E | N | S | I | K | D | V | - | I | I | N | Q | K | I | T |
| Sequence1-NP-EG-Jp-ABCCC | E | W | I | S | K | V | E | N | L | N | A | A | L | N | E | F | K | N | G | K | N | K | D | F | S | K | - | V | T | Q | A | K | S | D | L | E | N | S | V | K | D | V | - | I | I | N | Q | K | I | T |
| Sequence101-P-CG-Vit-ABD | E | W | I | S | K | I | E | N | L | N | A | A | L | N | D | F | K | N | G | K | N | N | D | F | S | K | - | V | T | Q | V | K | S | D | L | E | N | S | I | K | D | V | - | I | I | N | Q | K | I | T |
| Sequence64-NP-UD-OCC-ABC | E | W | I | S | K | V | E | N | L | N | T | A | L | N | E | F | K | N | G | K | N | K | D | F | S | K | - | V | T | Q | A | K | S | D | L | E | N | S | I | K | D | V | - | I | I | N | Q | K | I | T |
| Sequence81-P-CG-EA-ABD | E | W | I | S | K | I | E | N | L | N | A | A | L | N | D | F | K | N | G | K | N | K | D | F | S | K | - | V | T | Q | A | K | S | D | L | E | N | S | I | K | D | V | - | I | I | N | Q | K | I | T |
| Sequence150-NP-DA-Sen-BC | E | W | I | S | K | V | E | N | L | N | A | A | L | N | E | F | K | N | G | K | N | K | D | F | S | K | - | V | T | Q | A | K | S | D | L | E | N | S | V | K | D | V | - | I | I | N | Q | K | V | T |
| Sequence90-NP-UD-Vit-ABD | E | W | I | S | K | I | E | N | L | N | A | A | L | N | D | F | K | N | G | K | N | N | D | F | S | K | - | V | T | Q | A | K | S | D | L | E | N | S | I | K | D | V | - | I | I | N | Q | K | I | T |
| Sequence136-P-CG-Col-AACC | E | W | I | S | K | V | E | N | L | N | A | A | L | N | E | F | K | N | G | K | N | K | D | F | S | K | - | V | T | Q | A | K | S | D | L | E | N | S | I | K | D | V | - | I | I | N | Q | K | I | T |
| Sequence15-NP-UP-Jp-ABD | E | W | I | S | K | I | E | N | L | N | T | A | L | N | D | F | K | N | G | K | N | K | D | F | S | K | - | V | T | Q | A | K | S | D | L | E | N | S | I | K | D | V | - | I | I | N | Q | K | I | T |
| Sequence108-NP-UD-Vit-ABD | E | W | I | S | K | I | E | N | L | N | A | A | L | N | D | F | K | N | G | K | N | K | D | F | S | K | - | V | T | Q | A | K | S | D | L | E | N | S | I | K | D | V | - | I | I | N | Q | K | I | T |
| Sequence104-P-CG-OCC-ABCC | E | W | I | S | K | V | K | T | L | M | Q | L | L | N | E | F | K | N | S | K | N | K | D | F | S | K | - | V | T | Q | A | K | S | D | L | E | N | S | V | K | D | V | - | I | I | N | Q | K | I | T |
| Sequence179-NP-UD-Col-ABCC | E | W | I | S | K | V | E | N | L | N | A | A | L | N | D | F | K | N | G | K | N | K | D | F | S | K | - | V | T | Q | A | K | S | D | L | E | N | S | I | K | D | A | - | I | F | N | Q | K | I | T |
| Sequence37-NP-GA-Jp-ABCCC | E | W | I | S | K | V | E | N | L | N | T | A | L | N | E | F | K | N | G | K | N | K | D | F | S | K | - | V | T | Q | A | K | S | D | L | E | N | S | I | K | D | V | - | I | I | N | Q | K | I | T |
| Sequence89-NP-UD-Vit-ABD | E | W | I | S | K | I | E | N | L | N | A | A | L | N | D | F | K | N | G | K | N | K | D | F | S | K | - | V | T | Q | A | K | S | D | L | E | N | S | I | K | D | V | - | I | I | N | Q | K | I | T |
| Sequence92-P-CG-Vit-ABD | E | W | I | S | K | L | K | D | L | N | A | A | L | N | D | F | K | N | G | K | N | S | D | F | S | K | - | V | T | Q | A | K | S | D | L | E | N | S | I | K | D | V | - | I | I | N | Q | K | I | T |
| Sequence49-NP-GA-Jp-ABC | E | W | I | S | K | V | E | N | L | N | T | A | L | N | E | F | K | N | G | K | N | K | D | F | S | K | - | V | I | Q | A | K | S | D | L | E | N | S | I | K | D | V | - | I | I | N | Q | K | I | T |
| Sequence181-P-CG-Col-ABCC | E | W | I | S | K | V | E | N | L | N | A | A | L | N | D | F | K | N | G | K | N | K | D | F | S | K | - | V | T | Q | A | K | S | D | L | E | N | S | I | K | D | V | - | I | I | N | Q | K | I | T |
| Sequence25-NP-GA-Jp-ABD | E | W | I | S | K | I | E | N | L | N | A | A | L | N | D | F | K | N | G | K | N | K | D | F | S | K | - | V | T | Q | A | K | S | D | L | E | N | S | I | K | D | V | - | I | I | N | Q | K | I | T |
| Sequence4-P-CG-Jp-ABD | E | W | I | S | K | I | E | N | L | N | A | A | L | N | D | F | K | N | G | K | N | K | D | F | S | K | - | V | T | Q | A | K | S | D | L | E | N | S | I | K | D | V | - | I | I | N | Q | K | I | T |
| Sequence121-NP-G-Jp-ABCC | E | W | I | S | K | V | E | N | L | N | A | A | L | N | E | F | K | N | G | K | N | K | D | F | S | K | - | V | T | Q | A | K | S | D | L | E | N | S | V | K | D | V | - | I | I | N | Q | K | I | T |
| 4dvy | E | W | I | S | K | V | E | N | L | N | A | A | L | N | E | F | K | N | G | K | N | K | D | F | S | K | - | V | T | Q | A | K | S | D | L | E | N | S | V | K | D | V | - | I | I | N | Q | K | V | T |
| Sequence186-NP-UP-Jp-ABD | E | W | I | S | K | I | E | N | L | N | T | A | L | N | D | F | K | N | G | K | N | K | D | F | S | K | - | V | T | Q | A | K | S | D | L | E | N | S | I | K | D | V | - | I | I | N | Q | K | I | T |
| Sequence62-NP-UD-OCC-ABD | E | W | I | S | K | V | E | N | L | N | T | A | L | N | E | F | K | N | G | K | N | K | D | F | S | K | - | V | T | Q | A | K | S | D | L | E | N | S | I | K | D | V | - | I | I | N | Q | E | I | T |
| Sequence9-NP-GCA-Jp-ABD | E | W | I | S | K | I | E | N | L | N | A | A | L | N | D | F | K | N | G | K | N | K | D | F | S | K | - | V | T | Q | A | K | S | D | L | E | N | S | I | K | D | V | - | I | I | N | Q | K | I | T |
| Sequence12-NP-UP-Jp-ABD | E | W | I | S | K | I | E | N | L | N | A | A | L | N | D | F | K | N | G | K | N | K | D | F | S | K | - | V | T | Q | A | K | S | D | L | E | N | S | I | K | D | V | - | I | I | N | Q | K | I | T |
| Sequence35-NP-GA-Jp-ABABD | E | W | I | S | K | I | E | N | L | N | A | A | L | N | D | F | K | N | G | K | N | K | D | F | S | K | - | V | T | Q | A | K | S | D | L | E | N | S | I | K | D | V | - | I | I | N | Q | K | I | T |
| Sequence54-NP-GA-OCC-ABC | E | W | I | S | K | V | E | N | L | N | T | A | L | N | E | F | K | N | G | K | N | K | D | F | S | K | - | V | I | Q | A | K | S | D | L | E | N | S | I | K | D | V | - | I | I | N | Q | K | I | T |
| Sequence109-P-CG-Jp-ABD | E | W | I | S | K | I | E | N | L | N | A | A | L | N | D | F | K | N | G | K | N | K | D | F | S | K | - | V | T | Q | A | K | S | D | L | E | N | S | I | K | D | V | - | I | I | N | Q | K | I | T |
| Sequence96-NP-UD-Vit-ABD | E | W | I | S | E | I | E | N | L | N | A | A | L | N | D | F | K | N | G | K | Y | K | D | F | S | K | - | V | T | Q | A | K | S | D | L | E | N | S | I | K | D | V | - | I | I | N | Q | K | I | T |
| Sequence80-P-CG-EA-ABD | E | W | I | S | K | I | E | N | L | N | A | A | L | N | D | F | K | N | G | K | N | K | D | F | S | K | - | V | I | Q | A | K | S | D | L | E | N | S | I | K | D | V | - | I | I | N | Q | K | I | T |
| Sequence82-P-CG-EA-ABD | E | W | I | S | K | I | E | N | L | N | A | A | L | N | D | F | K | N | G | K | N | K | D | F | S | K | - | V | T | Q | A | K | S | D | L | E | N | S | I | K | D | V | - | I | I | N | Q | K | I | T |
| Sequence10-NP-GCA-Jp-ABD | E | W | I | S | K | I | E | N | L | N | A | A | L | N | D | F | K | N | G | K | N | K | D | F | S | K | - | V | T | Q | A | K | S | D | L | E | N | S | I | K | D | V | - | I | I | N | Q | K | I | T |
| Sequence19-NP-UP-Jp-ABBD | E | W | I | S | K | I | E | N | L | N | A | A | L | N | D | F | K | N | G | K | N | K | D | F | S | K | - | V | T | Q | A | K | S | D | L | E | N | S | I | K | D | V | - | I | I | N | Q | K | I | T |
| Sequence115-NP-UG-Jp-ABC | E | W | I | S | K | V | E | N | L | N | A | A | L | N | E | F | K | S | G | K | D | K | D | F | S | K | - | V | T | Q | A | K | R | D | L | E | N | S | G | K | D | V | - | I | I | N | Q | K | I | T |
| Sequence45-NP-GA-Jp-ABCC | E | W | I | S | K | V | E | N | L | N | A | A | L | N | D | F | K | N | G | K | N | K | D | F | S | K | - | V | T | Q | A | K | S | D | L | E | N | S | I | K | D | A | - | I | F | N | Q | K | I | T |
| Sequence119-NP-UG-Jp-ABC | E | W | I | S | K | V | E | N | L | N | A | A | L | N | D | F | K | N | G | K | N | K | D | F | S | K | - | V | T | Q | A | K | S | D | L | E | N | S | V | K | D | V | - | I | I | N | Q | K | I | T |
| Sequence70-NP-GA-Ch-ABD | - | - | - | - | - | - | - | - | - | - | - | - | - | - | - | - | - | - | - | - | - | - | - | - | - | - | - | - | - | - | - | - | - | - | - | - | - | - | - | - | - | - | - | - | - | - | - | - | - | - |
| Sequence52-NP-UD-EA-ABD | E | W | I | S | K | I | E | N | L | N | A | A | L | N | D | F | K | N | G | K | N | K | D | F | S | K | - | V | T | Q | A | K | S | D | L | E | N | S | I | K | D | V | - | I | I | N | Q | K | I | T |
| Sequence97-P-CG-Vit-ABD | E | W | I | S | K | I | E | N | L | N | A | A | L | N | D | F | K | N | G | K | N | K | D | F | S | K | - | V | T | Q | A | K | S | D | L | E | N | S | I | K | D | V | - | I | I | N | Q | K | I | T |
| Sequence94-NP-UD-Vit-ABD | E | W | I | S | K | I | E | N | L | N | A | A | L | N | D | F | K | N | G | K | N | N | G | F | Q | Q | G | N | T | S | P | K | A | T | L | K | I | P | L | R | M | C | - | S | L | I | K | K | V | T |
| Sequence176-NP-GA-Col-ABCC | E | W | I | S | K | V | E | N | L | N | A | A | L | N | D | F | K | N | G | K | N | K | D | F | S | K | - | V | T | Q | A | K | S | D | L | E | N | S | V | K | D | V | - | I | I | N | Q | K | V | T |
| Sequence68-NP-D-Am-ABBC | E | W | I | S | K | V | E | N | L | N | A | A | L | N | E | F | K | N | G | K | N | K | D | F | S | K | - | V | T | Q | A | K | S | D | L | E | N | S | I | K | D | V | - | I | I | N | Q | K | I | T |
| Sequence47-NP-GA-Jp-ABD | E | W | I | S | K | I | E | N | L | N | A | A | L | N | E | L | K | N | G | K | N | K | D | F | S | K | - | V | T | Q | A | K | S | D | L | E | N | S | I | K | D | V | - | I | I | N | Q | K | I | T |
| Sequence56-NP-UD-OCC-ABC | E | W | I | S | K | V | E | N | L | N | T | A | L | N | E | F | K | N | G | K | N | K | D | F | S | K | - | V | T | Q | A | K | S | D | L | E | N | S | I | K | D | V | - | I | I | N | Q | K | I | T |
| Sequence31-NP-GA-Jp-ABD | E | W | I | S | K | I | E | N | L | N | A | A | L | N | D | F | K | N | G | K | N | K | D | F | S | K | - | V | T | Q | A | K | S | D | L | E | N | S | I | K | D | V | - | I | I | N | Q | K | I | T |
| Sequence46-NP-UD-Jp-ABD | E | W | I | S | K | V | E | N | L | N | T | A | L | N | E | F | K | N | G | K | N | K | D | F | S | K | - | V | T | Q | A | K | S | D | L | E | N | S | I | K | D | V | - | I | I | N | Q | K | I | T |
| Sequence102-NP-UG-Vit-ABD | E | W | I | S | K | I | E | N | L | N | A | A | L | N | D | F | K | N | G | K | N | N | D | F | S | K | - | V | T | Q | V | K | S | D | L | E | N | S | I | K | D | V | - | I | I | N | Q | K | I | T |
| Sequence71-P-CG-EA-ABD | E | W | I | S | K | I | E | N | L | N | A | A | L | N | D | F | K | N | G | K | N | K | D | F | S | K | - | V | T | Q | A | K | S | D | L | E | N | S | I | K | D | V | - | I | I | N | Q | K | I | T |
| Sequence30-NP-GA-Jp-ABBD | E | W | I | S | K | I | E | N | L | N | A | A | L | N | D | F | K | N | G | K | N | K | D | F | S | K | - | V | I | Q | A | K | S | D | L | E | N | S | I | K | D | V | - | I | I | N | Q | K | I | T |
| Sequence28-NP-GA-Jp-AABD | E | W | I | S | K | I | E | N | L | N | A | A | L | N | D | F | K | N | G | K | N | K | D | F | S | K | - | V | T | Q | A | K | S | D | L | E | N | S | I | K | D | V | - | I | I | N | Q | K | I | T |
| Sequence98-P-CG-Vit-AD | D | G | F | Q | K | L | K | T | L | M | Q | L | L | N | D | F | K | N | G | N | Y | K | D | F | S | K | - | V | T | Q | A | K | S | D | L | E | N | S | I | K | D | V | - | I | I | N | Q | K | I | T |
| Sequence99-P-CG-Vit-ABD | E | W | I | S | K | I | E | N | L | N | A | A | L | N | D | F | K | N | G | K | N | N | D | F | S | K | - | V | T | Q | A | K | S | D | L | E | N | S | I | K | D | V | - | I | I | N | Q | K | I | T |
| Sequence24-NP-UD-USA-ABD | E | W | I | S | K | I | E | N | L | N | A | A | L | N | D | F | K | N | G | K | N | K | D | F | S | K | - | V | T | Q | A | K | S | D | L | E | N | S | I | K | D | V | I | I | I | N | Q | K | I | T |
| Sequence124-NP-G-Jp-ABC | E | W | I | S | K | V | E | N | L | N | A | A | L | N | E | F | K | N | G | K | N | K | D | F | S | K | - | V | T | Q | A | K | S | D | L | E | N | S | I | K | D | V | - | I | I | N | Q | K | I | T |
| Sequence77-P-CG-OCC-ABCC | E | W | I | S | K | V | E | N | L | N | T | A | L | N | E | F | K | N | G | K | N | K | D | F | S | K | - | V | I | Q | A | K | S | D | L | E | N | S | I | K | D | V | - | I | I | N | Q | K | I | T |
| Sequence113-NP-UG-Jp-ABC | E | W | I | S | K | V | E | N | L | N | A | A | L | N | D | F | K | N | G | K | N | K | D | F | S | K | - | V | T | Q | A | K | S | D | L | E | N | S | I | K | D | A | - | I | F | N | Q | K | I | T |
| Sequence88-NP-UD-Vit-ABD | E | W | I | S | K | I | E | N | L | N | A | A | L | N | D | F | K | N | G | K | N | N | D | F | S | K | - | V | A | Q | A | K | S | D | L | E | N | S | I | K | D | V | - | I | I | N | Q | K | I | T |
| Sequence100-NP-UD-Vit-ABD | E | W | I | S | K | I | E | N | L | N | A | A | L | N | D | F | K | N | G | K | N | K | D | F | S | K | - | V | T | Q | A | K | S | D | L | E | N | S | I | K | D | V | - | I | I | N | Q | K | I | T |
| Sequence137-NP-G-Col-ABCC | E | W | I | S | K | V | E | N | L | N | A | A | L | N | D | F | K | N | G | K | N | K | D | F | S | K | - | V | T | Q | A | K | S | D | L | E | N | S | V | K | D | V | - | I | I | N | Q | K | V | T |

```
Sequence117-NP-G-Jp-ABC        EWISKVENLNAALNEFKNGKNKDFSK-VTQAKSDLENSVKDV-IINQKIT
Sequence171-NP-UD-Col-ABCC     EWISKVENLNAALNEFKNGKNRDFSK-VTQAKSDLENSVKDV-IINQKIA
Sequence180-P-CG-Col-ABC       EWISKVENLNAALNDFKNGKNKDFSK-VTQAKSDLENSIKDA-IFNQKIT
Sequence112-P-CG-Jp-ABCC       EWISKVENLNAALNEFKNGKNKDFSK-VTQAKSDLENSVKDV-IINQKIT
Sequence127-NP-UD-Jp-ABC       EWISKVENLNAALNEFKNGKNKDFSK-VTQAKSDLENSVKDV-IINQKIT
Sequence51-NP-GA-OCC-ABC       EWISKVENLNTALNEFKNGKNKDFSK-VIQAKSDLENSIKDV-IINQKIT
Sequence73-P-CG-EA-ABD         EWISKIENLNAALNDFKNGKNKDFSK-VTQAKSDLENSIKDV-IINQKIT
Sequence183-NP-UD-Col-ABD      EWISKVENLNAALNDFKNGKNKDFSK-VTQAKSDLENSIKDA-IFNQKIT
Sequence34-NP-UD-Jp-ABDD       EWISKIENLNAALNDFKNGKNKDFSK-VTQAKSDLENSIKDV-IINQKIT
Sequence21-P-CG-Jp-ABD         EWISKIENLNAALNDFKNGKNKDSSK-VTQAKSDLENSIKDV-IINQKIT
Sequence32-NP-GA-Jp-ABD        EWISKIENLNAALNDFKNGKNKDFSK-VTQAKSDLENSIKDV-IINQKIT
Sequence116-NP-UD-Jp-ABC       EWISKVENLNAALNEFKNGKNKDFSK-VTQAKSDLENSVKDV-IINQKIT
Sequence8-NP-GCA-Jp-ABD        EWISKIENLNAALNDFKNGKNKDFSK-VTQAKSDLENSIKDV-IINQKIT
Sequence83-P-CG-EA-ABD         EWISKIENLNAALNDFKNGKNKDFSK-VTQAKSDLENSIKDV-IINQKIT
Sequence48-NP-UD-OCC-ABC       EWISKVENLNTALNEFKNGKNKDFSK-VTQAKSDLENSIKDV-IINQKIT
Sequence120-NP-G-Jp-ABC        EWISKVENLNAALNEFKNGKNKDFSK-VTQAKSDLENSVKDV-IINQKIT
Sequence187-NP-UG-Jp-ABCCC     EWISKVENLNAALNEFKNGKNKDFSK-VTQAKSDLENSVKDV-IINQKIT
Sequence118-NP-G-Jp-ABD        EWISKIENLNAALNDFKNGKNKDFSK-VTQAKSDLENSIKDV-IINQKIT
Sequence87-NP-UG-Vit-ABD       EWISKIENLNAALNDFKNGKNKDFSK-VTQAKSDLENSIKDV-IINQKIT

                               801                                             850
Sequence140-NP-GA-Col-ABABC    DKVDDLNQAVPVAKATGDFSMVEQALADLKNFSKEQLAQQAQKNESLNAG
Sequence123-NP-UD-Jp-ABD       DKVDNLNQAVSETKLTGDFSKVEQALAELKNLS-------------LDLG
Sequence111-NP-G-Jp-ABD        DKVDNLNQAVSETKLTGDFSKVEQALAELKNLS------LDQKNESFNVG
Sequence57-NP-GA-OCC-AB        DKVDNLNQAVSIANSTGDFSGVEQALADLKNFSKGQLTQQAQKNEDFNTG
Sequence172-P-CG-Col-ABCC      DKVDDLNQAVSVAKATGDFSRVGQALADLKNFSKEQLAQQTQKNESFNVG
Sequence110-NP-UD-Jp-ABD       DKVENLNQAVSETKLTGDFSKVEQALAELKNLS-------------LDLG
Sequence95-NP-UD-Vit-ABD       DKVDNLNQAVSETKLTGDFSKVEQALAELKNLS------LDQKNESFNVG
Sequence107-P-CG-Vit-ABD       DKVENLNQAVSETKLTGDFSKVEQALAELKNLS------LDQKNESFNVG
Sequence106-P-CG-Vit-ABD       DKVDNLNQAVSETKLTGDFSKVEQALAELKNLS------LDQKNESFNVG
Sequence105-NP-UD-Vit-ABD      DEVDNLNQAVSETKLTGDFSKVEQALAELKNLS------LDQKNESFNVG
Sequence126-NP-UG-Jp-ABC       DKVDNLNQAVSVAKATGDFSRVEQALADLKNFSKEQLAQQTQKNESFNVG
Sequence135-P-CG-Col-ABCCC     DKVDNLNQEVSVAKATGDFSGVEQALADLKNFSKEQLAQQTQKNESFNVG
Sequence138-NP-GA-Col-ABC      DKVDDLNQAVSVAKATDDFSRVEQALADLKNFSKEQLAQQTQKNESFNVG
Sequence13-NP-UP-Jp-ABD        DKVDNLNQAVSETKLTGDFSKVEQALAELKSLS-------------LDLG
Sequence11-NP-UP-Jp-ABD        DKVDNLNQAVSETKLAGDFSKVEQALAELKSLS-------------LDLG
Sequence50-NP-GA-OCC-ABC       DKVDNLNQAVSIAKATGDFSRVEQALADLKNFSKGQLTQQAQKNEDFNTG
Sequence26-NP-GA-Jp-ABD        DKVDNLNQAVLETKSTGNFSKVEQALAELKSLS-------------LDLG
Sequence55-NP-UD-OCC-ABCCC     DKVDNLNQAVSIAKGTGDFSRVEQALADLKNFSKGQLTQQAQKNEDFNTG
Sequence178-NP-M-Col-ABCC      DKVDDLNQAVSVAKATGDFSRVEQALADLKNFSKEQLAQQAQKNESFNVG
Sequence40-NP-GA-Jp-ABD        DKVDNLNQAVSETKLTGDFSKVEQALAELKNLS-------------LDLG
Sequence43-NP-GA-Jp-ABD        DKVDNLNQAVSETKLTGDFSKVEQALAELKNLS-------------LDLG
Sequence139-NP-UD-Col-ABCC     DKVDDLNQAVSVAKATGDFSRVEQALADLKNFSKEQLAQQTQKNESFNVG
Sequence7-P-CG-Jp-ABBD         DKVDNLNQAVSETKLTGDFSKVEQALAELKNLS-------------LDLG
Sequence91-P-CG-Vit-ABD        DKVDKLNQAVSETKLTGDFSKVEQALAEFKSLS-------------LDLG
Sequence29-NP-GA-Jp-ABABD      DKVDNLNQAVSETKLTGDFSKVEQVLAELKNLS-------------LDLG
Sequence78-P-CG-EA-ABD         DKVDNLNQAVSETKLTGDFSKVEQALAELKNLS-------------LDLG
Sequence188-NP-GCA-Jp-AB       DKVDNLNQAVSVAKATGDFSRVEQALADLKNFSKEQLAQQAQKNESLNAR
Sequence72-P-CG-EA-ABD         DKVDNLNQAVSETKLTGDFSKVEQALAELKNLS-------------LDLG
Sequence79-P-CG-EA-ABD         DKVDNLNQAVSETKLTGDFSKVEQALAELKSLS-------------LDLG
Sequence61-NP-UD-OCC-ABC       DKVDNLNQAVSIAKETGDFSRVEQALADLKNFSKGQLTQQAQKNEDFNTG
Sequence125-NP-UG-Jp-ABCC      DKVDDLNQAVSIAKAMGDFSRVEQALADLKNFSKEQLAQQTQKNESFHTG
Sequence175-NP-G-Col-ABCC      DKVDDLNQAVSVAKTMGDFSRVEQALADLKNFSKEQLAQQTQKNESFNVG
Sequence122-NP-G-Jp-ABCC       DKVDDLNQAVSVAKTMGDFSRVEQALADLKNFSKEQLAQQTQKNESFNVG
Sequence36-NP-GA-Jp-ABD        DKVDNLNQAVSETKLTGDFSKVEQALAELKNLS-------------LDLG
Sequence58-NP-UD-OCC-ABC       DKVDNLNQAVSIAKATGDFSRVEQALADLKNFSKGQLTQQAQKNEDFNTG
Sequence27-NP-GA-Jp-ABBD       DKVDNLNQAVSETKLTGDFSKVEQALAELKSLS-------------LDLG
Sequence93-P-CG-Vit-ABD        DKVDNLNQAVSETKLTGDFSKVEQALAELKSLS------LDQKNESFNVG
Sequence177-NP-M-Col-ABCC      DKVDNLNQAVSVAKATGDFSGVEQALADLKNFSKEQLAQQTQKNESLNVG
Sequence182-NP-UD-Col-ABCC     DKVDDLNQAVSVAKAMGDFSRVEQALADLKNFSKEQLAQQAQ--------
Sequence114-NP-UG-Jp-ABC       DKVDDLNQAVSVAKATGDFSRVEQALADLKNFSKEQLAQQAQKNESFNVG
```

| | |
|---|---|
| Sequence74-P-CG-EA-ABD | DKVDNLNQAVSETKLTGDFSKVEQALAELKNLS-------------LDLG |
| Sequence20-P-CG-Jp-ABD | DKVDNLNQAVSETKLTGDFSRVEQALAELKNLS-------------LDLG |
| Sequence14-NP-GCA-Jp-ABD | DKVDNLNQAVSETKLTGDFSKVEQALAELKNLS-------------LDLG |
| Sequence39-NP-GA-Jp-ABC | DKVDDLNQAVSVAKIAGDFSGIEQALADLKNFSKEQLAQQAQKNEDFNTG |
| Sequence170-NP-UD-Col-ABCC | DKVDNLNQAVLMAKATGDFSRVEQALADLKNFSKEQLAQQAQKNESVNVG |
| Sequence76-P-CG-EA-ABD | DKVDNLNQAVSETKLTGDFSKVEQALAELKSLS-------------LDLG |
| Sequence53-NP-GA-EA-ABD | DKVDNLNQAVSETKLTGDFSKVEQALAELKNLS-------------LDLG |
| Sequence174-P-CG-Col-ABCC | DKVDNLNQAASVAKATGDFSRVEQALADLKNFSKEQLAQQTQKNESLNVG |
| Sequence33-NP-UG-Jp-ABD | DKVDDLNQAVSVAKATGDFSRVEQALADLKNFSKEQLAQQTQKNESFNVG |
| Sequence60-NP-GA-EA-ABD | DRVDNLNQAVSETKLTGDFSKVEQALAELKSLS-------------LDLG |
| Sequence38-NP-GA-Jp-ABD | DKVDNLNQAVSETKLAGDFSKVEQALAELKSLS-------------LDLG |
| Sequence1-NP-EG-Jp-ABCCC | DKVDNLNQAVSMAKATGGFSRVEQALADLKNFSKEQLAQQTQKNESFNVG |
| Sequence101-P-CG-Vit-ABD | DKVENLNQAVSETKLTGNFSKVEQALAELKSLS------LDQKNESFNVG |
| Sequence64-NP-UD-OCC-ABC | DKVDNLNQAVSIAKATGDFSRVEQALADLKNFSKGQLTQQAQKNEDFNTG |
| Sequence81-P-CG-EA-ABD | DKVDNLNQAVSETKLTGDFSKVEQALAELKSLS-------------LDLG |
| Sequence150-NP-DA-Sen-BC | DKVDNLNQAVSVAKATGDFSKVEQALADLKNFSKEQLAQQAQKNEDFNTG |
| Sequence90-NP-UD-Vit-ABD | DKIANLNQAVSETKLTGDFSKVEQALAELKNLS-------------LDLG |
| Sequence136-P-CG-Col-AACC | DKVDDLNQAVLVAKATGDFSGVEQALADLKNFSKEQLAQQTQKNESFNVG |
| Sequence15-NP-UP-Jp-ABD | DKVDNLNQAVSETKLTGDFSKVEQVLAELKSLS-------------LDLG |
| Sequence108-NP-UD-Vit-ABD | DKVDNLNQAVSETKLTGDFSKVEQALAELKNLS------LDQKNESFNVG |
| Sequence104-P-CG-OCC-ABCC | DKVDNLNQAVSVAKATGDFSRVNQVLADLKNFSKEQLAQQAQKNEDFNTG |
| Sequence179-NP-UD-Col-ABCC | DKVDDLNQAVSVAKAMGDFSRVEQALADLKNFSKEQLAQQAQKNESLNAG |
| Sequence37-NP-GA-Jp-ABCCC | DKVDNLNQAVSIAKETGDFSRVEQALADLKNFSKGQLTQQAQKNEDFNTG |
| Sequence89-NP-UD-Vit-ABD | DKVDNLNQAVSETKLTGDFSKVEQALAELKSLS------LDQKNESFNVG |
| Sequence92-P-CG-Vit-ABD | DKVDKLNQAVSETKLTGDFSKVEQALAEFKSLS-------------LDLG |
| Sequence49-NP-GA-Jp-ABC | DKVDNLNQAVSIAKATGDFSRVEQALADLKNFSKGQLTQQAQKNEDFNTG |
| Sequence181-P-CG-Col-ABCC | DKVDNLNQAVSVAKATGDFSRVEQALADLKNFSKEQLAQQTQKNESFNVG |
| Sequence25-NP-GA-Jp-ABD | DKVDNLNQAVSETKLTGDFSKVEQALAELKNLS-------------LDLG |
| Sequence4-P-CG-Jp-ABD | DKVDNLNQAVSEIKLTGDFSKVEQALAELKNLS-------------LDLG |
| Sequence121-NP-G-Jp-ABCC | DKVDNLNQAVSVAKATGDFSRVEQALADLKNFSKEQLAQQAQKNEDFNAG |
| 4dvy | DKVDNLNQAVSVAKAMGDFSRVEQVLADLKNFSKEQLAQQAQKNEDFNTG |
| Sequence186-NP-UP-Jp-ABD | DKVDNLNQAVSETKLTGDFSKVEQALAELKNLS-------------LDLG |
| Sequence62-NP-UD-OCC-ABD | DKVDNLNQAVSIAKETGDFSGVEQALADLKNFSKGQLTQQAQKNEDFNTG |
| Sequence9-NP-GCA-Jp-ABD | DKVDNLNQAVSETKLTGDFSKVEQALAELKNLS-------------LDLG |
| Sequence12-NP-UP-Jp-ABD | DKVDNLNQAVSETKLTGDFSKVEQALAELKSLS-------------LDLG |
| Sequence35-NP-GA-Jp-ABABD | DKVDNLNQAVSETKLTGDFSKVEQALAELKSLS-------------LDLG |
| Sequence54-NP-GA-OCC-ABC | DKVDNLNQAVSIARAIGDFSGVEQALADLKNFSKGQLTQQAQKNEDFNTG |
| Sequence109-P-CG-Jp-ABD | DKVDNLNQAVSETKLTGDFSKVEQALAELKNLS-------------LDLG |
| Sequence96-NP-UD-Vit-ABD | DKVENLNQAVSETKLTGDFSKVEQALAELKSLS------LDQKNESFNVG |
| Sequence80-P-CG-EA-ABD | DKVDNLNQAVSETKLTGNFSKVEQALAELKSLS-------------LDLG |
| Sequence82-P-CG-EA-ABD | DKVDNLNQAISETKLTGDFSKVEQALAELKSLS-------------LDLG |
| Sequence10-NP-GCA-Jp-ABD | DKVDNLNQAVSETKLTGDFSRVEQALAELKSLS-------------LDLG |
| Sequence19-NP-UP-Jp-ABBD | DKVDNLNQAVSETKLTGDFSKVEQALAELKSLS-------------LDLG |
| Sequence115-NP-UG-Jp-ABC | DKVDDLNQAVSVAKATGDFSRVEQALADLKNFSKEQLAQQAQKNEDFHTG |
| Sequence45-NP-GA-Jp-ABCC | DKVDDLNQAVSVAKATGDFSRVEQALADLKNFSKEQLAQQAQKNESLNAG |
| Sequence119-NP-UG-Jp-ABC | DKVDNLSSAVSVAKATGDFSMVEQVLADLKNFSKDQLAQQAPKNEDFNTG |
| Sequence70-NP-GA-Ch-ABD | -------------------------------------------------- |
| Sequence52-NP-UD-EA-ABD | DKVDNLNQAVSETKLTGDFSKVEQALAELKNLS-------------LDLG |
| Sequence97-P-CG-Vit-ABD | DKVENLNQAVSETKLTGDFSKVEQALAELKNLS------LDQKNESFNVG |
| Sequence94-NP-UD-Vit-ABD | DKVDNLNQAVSETKLTGDFSKVEQALAELKSLS------LDQKNESFNVG |
| Sequence176-NP-GA-Col-ABCC | DKVDNLNQAVSVAKATGDFSGVEQALADLKNFSKEQLAQQAQKNE----- |
| Sequence68-NP-D-Am-ABBC | DKVDNLNQAVSVAKATGDFSGVEQALADLKNFSKEQLAQQAQKNESFNVG |
| Sequence47-NP-GA-Jp-ABD | DKVDNLNQAVSETKLTGDFSKVEQALAELKNLS-------------LDLG |
| Sequence56-NP-UD-OCC-ABC | DKVDNLNQAVSIAKATGDFSRVEQALADLKNFSKGQLTQQAQKNEDFNTG |
| Sequence31-NP-GA-Jp-ABD | DKVDNLNQAVSETKLTGDFSKVEQALAELKSLS-------------LDLG |
| Sequence46-NP-UD-Jp-ABD | DKVDNLNQAVSIAKATSDFSRVEQALAELKNLS-------------LDLG |
| Sequence102-NP-UG-Vit-ABD | DKVENLNQAVSETKLTGNFSKVEQALAELKSLS------LDQKNESFNVG |
| Sequence71-P-CG-EA-ABD | DKVDNLNQAVSETKLTGDFSKVEQALAELKNLS-------------LDLG |
| Sequence30-NP-GA-Jp-ABBD | DKVDNLNQAVSETKLTGDFSKVEQALAELKNLS------LDQKNESFNVG |
| Sequence28-NP-GA-Jp-AABD | DKVDNLNQAVSETKLTGDFSKVEQALAELKSLS-------------LDLG |
| Sequence98-P-CG-Vit-AD | DKVDNLNQAVSETKLTGDFSKVEQALAELKSLS------LDQKNESFNVG |

```
Sequence99-P-CG-Vit-ABD         DKVDNLNQAVSETKLTGDFSKVEQALAELKNLS------LDQKNESFNVG
Sequence24-NP-UD-USA-ABD        DKVDNLNQAVSETKLTGDFSKVEQALSELKSLS-------------LDLG
Sequence124-NP-G-Jp-ABC         DKVDNLNQAVSVAKATGDFSGVEQALADLKNFSKEQLAQQAQKSESFNVG
Sequence77-P-CG-OCC-ABCC        DKVDNLNQAVSIAKATGDFSGVEQALADLKNFSKGQLTQQAQKNEDFNTG
Sequence113-NP-UG-Jp-ABC        DKVDDLNQAVSVAKATGDFSRVEQALADLKNFSKEQLAQQAQKNESFNVG
Sequence88-NP-UD-Vit-ABD        DKVDNLNQAVSETKLTGDFSKVEQALAELKNLS------LDQKNESFNVG
Sequence100-NP-UD-Vit-ABD       DKVDKLNQAVSETKLTGDFSKVEQALAELKSLS------LDQKNESFNVG
Sequence137-NP-G-Col-ABCC       DKVDNLNQAVSVAKATGDFSGVEQALADLKNFSKEQLAQQAQKNESFNAG
Sequence117-NP-G-Jp-ABC         DKVDNLNQAVSVAKATGDFSRVEQALADLKNFSKEQLAQQAQKNEDFNAG
Sequence171-NP-UD-Col-ABCC      DKVDDLNQAVSVAKATGDFSRVEQALADLKNFSKEQLAQQTQKNESFNVG
Sequence180-P-CG-Col-ABC        DKVDDLNQAVSVAKAMGDFSRVEQALADLKNFSKEQLAQQAQKNESLNAG
Sequence112-P-CG-Jp-ABCC        DKVDNLNQAVSVAKATGDFSRVEQALADLKNFSKEQLAQQAQKNEDFNAG
Sequence127-NP-UD-Jp-ABC        DKVDNLNQAVSVAKATGDFSRVEQALADLKNFSKEQLAQQAQKNESFNAG
Sequence51-NP-GA-OCC-ABC        DKVDNLNQAISIAKATGDFSGVEQALADLKNFSKGQLTQQAQKNEDFNTG
Sequence73-P-CG-EA-ABD          DKVDNLNQAVSETKLTGDFSKVEQALAELKNLS-------------LDLG
Sequence183-NP-UD-Col-ABD       DKVDDLNQAVSVAKATGDFSRVEQALADLKNFSKEQLAQQAQKNESLNAG
Sequence34-NP-UD-Jp-ABDD        DKVDNLNQAVSETKLTGDFSKVEQVLADLKNFSKEQLAQQTQKNESLNAG
Sequence21-P-CG-Jp-ABD          DKVDNLNQAVSETKLTGDFSKVEQALAELKSLS-------------LDLG
Sequence32-NP-GA-Jp-ABD         DKVDNLNQAVSETKLTGDFSKVEQALAELKSLS-------------LDLG
Sequence116-NP-UD-Jp-ABC        DKVDDLNQAVSVAKATGDFSRVEQALADLKNFSKEQLAQQAQKNEDFNTG
Sequence8-NP-GCA-Jp-ABD         DKVDNLNQAVSETKLTGDFSKVEQALAELKSLS-------------LDLG
Sequence83-P-CG-EA-ABD          DKVDNLNQAVSETKLTGDFSKVEQALAELKNLS-------------LDLG
Sequence48-NP-UD-OCC-ABC        DKVDNLNQAVSIAKATGDFSGVEQVLADLKNFSKGQLAQQAQKNEDFNTG
Sequence120-NP-G-Jp-ABC         DKVDNLNQGVLVAKATGDFSKVEQALADLKNFSKEQLAQQTQKNESFNVG
Sequence187-NP-UG-Jp-ABCCC      DKVDNLNQAVSMAKATGDFSRVEQALADLKNFSKEQLAQQTQKNESFNVG
Sequence118-NP-G-Jp-ABD         DKVDNLNQAVSETKLTGDFSKVEQALAELKNLS-------------LDLG
Sequence87-NP-UG-Vit-ABD        DKVDNLNQAVSETKLTGDFSKVEQALAELKNLS------LDQKNESFNVG

                                851                                            900
Sequence140-NP-GA-Col-ABABC     KKSEIYQSV-----------------------------------------
Sequence123-NP-UD-Jp-ABD        KNSDLQ-SVKNSVNGTLVGNGLSKTEATTLTKNFSEIRKELNEKLFGNSN
Sequence111-NP-G-Jp-ABD         KNSDLQ-SVRDSVRGTLVGNGLSKTEATKLSKNFSDIRKELSEKLFG---
Sequence57-NP-GA-OCC-AB         KNSELYQSVKNGVNGTLVGNGLSKAEATTLSKNFSDIKKELN--------
Sequence172-P-CG-Col-ABCC       KKSEIYQSVKNGVNGTLVGNGLSQAEATTLSKNFSDIKKELN--------
Sequence110-NP-UD-Jp-ABD        KNSDLQ-SVKNSVNGTLVGNGLSKTEATTLTKNFSDIRKELNEKLFGNSN
Sequence95-NP-UD-Vit-ABD        KNSDLQ-SVRDSVRGTLVGNGLSKTEATKLSKNFSDIRKELSEKLFGKSN
Sequence107-P-CG-Vit-ABD        KNSDLQ-SVRDSVRGTLVGNGLSKTEATKLSKNFSDIRKELSEKLFG---
Sequence106-P-CG-Vit-ABD        KNSDLQ-SVRDSVKGTLVGNGLSKTEATKLSKNFSDIRKELSEK------
Sequence105-NP-UD-Vit-ABD       KNSDLQ-SVRDSVRGTLVGNGLSKTEATKLSKNFSDIRKELSEKLF----
Sequence126-NP-UG-Jp-ABC        KKSEIYQSVKNNVNGTLVGNGLSQAEATTLSKNFSDIKKELNEKF-----
Sequence135-P-CG-Col-ABCCC      KKSEIYQSVKNGVNGVLVGNGLSGIEVTALAKNFSDIKKELN--------
Sequence138-NP-GA-Col-ABC       KKSEIYQSVKNGVNGTLVGNGLSGIEATALAKNFSDIKKELNEKF-----
Sequence13-NP-UP-Jp-ABD         KNSDLQKSVKNGVNGTLVGNGLSKTEATTLTKNFSDIRKELNEKLFGNSN
Sequence11-NP-UP-Jp-ABD         KNSDLQKSVKNGVNGTLVGNGLSKTEATTLTKNFSDIRKELNEKLFGNSN
Sequence50-NP-GA-OCC-ABC        KNSKLYQSVKNGVNGTLVGNGLSGIEATALAKKFSDIKKELN--------
Sequence26-NP-GA-Jp-ABD         KNSDLQKSVKNGVNGTLVGNGLSKTEATTLTKNFSDIRKELNEKLFGNSN
Sequence55-NP-UD-OCC-ABCCC      KNSKLYQSVKNGVNGTLVGNGLSGIEATALAKKFSDIKKELN--------
Sequence178-NP-M-Col-ABCC       KKSEIYQSVKNGVNGTLVGNGLSGIEATALAKNFSDIKKELN--------
Sequence40-NP-GA-Jp-ABD         KNSDLQKSVKNGVNGTLVGNGLSKTEATTLTKNFSDIRKELNEKLFGNSN
Sequence43-NP-GA-Jp-ABD         KNSDLQKSVKNGVNGTLVGNGLSKTEATTLTKNFSDIRKELNEKLFGNSN
Sequence139-NP-UD-Col-ABCC      KKSEIYQSVKNGVNGTLVGNGLSQAEATTLSKNFSDIKKELNA-------
Sequence7-P-CG-Jp-ABBD          KNSDLQKSVKNGVNGTLVGNGLSKTEATTLTKNFSDIRKELNEKLFGNSN
Sequence91-P-CG-Vit-ABD         KNSDLQKSVKNGVNGTLVGNGLSKTEATKLTKNFSDIRRELNEKLFGNSN
Sequence29-NP-GA-Jp-ABABD       KNSDLQKSVKNGVNGTLVGNGLSKTEATTLTKNFSDIRKELNEKLFGNSN
Sequence78-P-CG-EA-ABD          KNSDLQKSVKNGVNGTLVGNGLSKTEATTLTKNFSDIRKELNEKLFGNSN
Sequence188-NP-GCA-Jp-AB        KKSEIYQSVKNGVNGTLVGNGLSQAEATTLSKNFSDIKKELNAKL-----
Sequence72-P-CG-EA-ABD          KNSDLQKSVKNGVNGTLVGNGLSKTEATTLTKNFSDIRKELNEKLFGNSN
Sequence79-P-CG-EA-ABD          KNSDLQKSVKNNVNGTLVGNGLSKTEATTLTKNFSDIRKELNEKLFGNSN
Sequence61-NP-UD-OCC-ABC        KNSKLYQSVKN-VNGTLVGNGLSGIEATALAKKFSDIKKEL---------
Sequence125-NP-UG-Jp-ABCC       KKSEIYQSVKNGVNGTLVGNGLSQTEATTLSKNFSDIKKELNAKL-----
Sequence175-NP-G-Col-ABCC       KKSEIYQSVKNGVNGTLVGNGLSGIEATALAKNFSDIK------------
```

```
Sequence122-NP-G-Jp-ABCC       KKSEIYQSVKNGVNGTLVGNGLSGIEATALAKNFSDIKKELNEKF-----
Sequence36-NP-GA-Jp-ABD        KNSDLQKSVKNGVNGTLVGNGLSKTEATTLTKNFSDIRKELNEKLFGNSN
Sequence58-NP-UD-OCC-ABC       KNSKLYQSVKNGVNGTLVGNGLSGIEATALAKKFSDIKKELN--------
Sequence27-NP-GA-Jp-ABBD       KNSDLQKSVKNGVNGTLVGNGLSKTEATTLTKNFSDIRKELNEKLFGNSN
Sequence93-P-CG-Vit-ABD        KNSDLQ-SVRDSVRGTLVGNGLSKTEATKLSKNFSDIRKELSEKLFGK--
Sequence177-NP-M-Col-ABCC      KKSEIYQSVKNGVNGTLVGNGLSGIEATALAKNFSDIKKEL---------
Sequence182-NP-UD-Col-ABCC     --------------------------------------------------
Sequence114-NP-UG-Jp-ABC       KKSEIYQSVKNGVNGTLVGNGLSKAEATTLSKNFSDIKKELNAKL-----
Sequence74-P-CG-EA-ABD         KNSDLQKSVKNGVNGTLVGNGLSKTEATTLTKNFSDIRKELNEKLFGNSN
Sequence20-P-CG-Jp-ABD         KNSDLQKSVKNGVNGTLVGNGLSKTEATTLTKNFSDIRKELNEKLFGNSN
Sequence14-NP-GCA-Jp-ABD       KNSDLQKSVKNGVNGTLVGNGLSKAEATTLTKSFSDIRKELNEKLFGNSN
Sequence39-NP-GA-Jp-ABC        KNSALYQSVKNGVNGTLVGNGLSKAEATTLSKNFSDIKKELNAKL-----
Sequence170-NP-UD-Col-ABCC     KKSEIYQSVKNGVNGTLVGNGLSQAEATTLSKNFSDIKKELN--------
Sequence76-P-CG-EA-ABD         KNSDLQKSVKNGVNGTLVGNGLSKTEATTLTKNFSDIRKELNEKLFGNSN
Sequence53-NP-GA-EA-ABD        KNSDLQKSVKNGVNGTLVGNGLSKTEATTLTKNFSEIRKELNEKLFGNSN
Sequence174-P-CG-Col-ABCC      KKSEIYQSVKNGVNGTLVGNG-----------------------------
Sequence33-NP-UG-Jp-ABD        KKSEIYQSVKNGVNGTLVGNGLSGIEATALAKNFSDIKKELNEKL-----
Sequence60-NP-GA-EA-ABD        KNSDLQKSVKNGVNGTLVGNGLSKTEATTLTKNFSDIRKELNEKLFGNSN
Sequence38-NP-GA-Jp-ABD        KNSDLQKSVKNGVNGTLVGNGLSETDATTLTKNFSDIRKELNEKLFGNSN
Sequence1-NP-EG-Jp-ABCCC       KKSEIYQSVKNGVNGTLVGNGLSGIEATALAKNFSDIKKELNEKF-----
Sequence101-P-CG-Vit-ABD       KNSDLQ-SVRDSVRGTLVGNGLSKTEATKLSKNFSDIRKELSEKLFG---
Sequence64-NP-UD-OCC-ABC       KNSKLYQSVKNGVNGTLVGNGLSGIEATALAKKFSD--------------
Sequence81-P-CG-EA-ABD         KNSDLQKSIKNGVNGTLVGNGLSKTEATTLTKNFSDIRRELNEKLFGNSN
Sequence150-NP-DA-Sen-BC       KNSALYQSVKNGVNGTLVGNGLSKAEATTLSKNFSDIKKELNAKL-----
Sequence90-NP-UD-Vit-ABD       KNSDLQKSVKNGVNGTLVGNGLSKTEATNLTKNFSDIKKELSEKLFGKSN
Sequence136-P-CG-Col-AACC      REFEIYQFVK----------------------------------------
Sequence15-NP-UP-Jp-ABD        KNSDLQKSVKNGVNGTLVGNGLSKTEATTLTKNFSDIRKELNEKLFGNSN
Sequence108-NP-UD-Vit-ABD      KNSDLQ-FVIDSVRGTLVGNGLSKTEATKLSKNFLDIRKELSEKLFGK--
Sequence104-P-CG-OCC-ABCC      KNSALYQSVKNGVNGTLVGNGLSKAEATTLSKNFSDIKKELN--------
Sequence179-NP-UD-Col-ABCC     KKSEIYQSVKNGVNGTLVGNG-----------------------------
Sequence37-NP-GA-Jp-ABCCC      KNSKLYQSVKNGVNGTLVGNGLSGIEATALAKKFSDIKKELN--------
Sequence89-NP-UD-Vit-ABD       KNSDLQ-SVRDSVRGTLVGNGLSKTEATKLSKNFSDIRKELSEKLFG---
Sequence92-P-CG-Vit-ABD        KNSDLQKSVKNGVNGTLVGNGLSKTEATKLTKNFSDIRRELNEKLFGNSN
Sequence49-NP-GA-Jp-ABC        KNSKLYQSVKNGVNGTLVGNGLSGIEATALAKKFSDIKKELN--------
Sequence181-P-CG-Col-ABCC      KISEIYQSVKNGVNGTLVGNGLSKAE------------------------
Sequence25-NP-GA-Jp-ABD        KNSDLQKSVKNGINGTLVGNGLSKTEATTLTKNFSDIRKELNEKLFGNSN
Sequence4-P-CG-Jp-ABD          KNSDLQKSVKNGVNGTLVSNGLSKTEATTLTKNFSDIRKELNEKLFGNSN
Sequence121-NP-G-Jp-ABCC       KKSEIYQSVKNGVNGTLVGNGLSKAEATTLSKNFSDIKKELNAKL-----
4dvy                           KNSELYQSVKNSVNKTLVGNGLSGIEATALAKNFSDIKKE----------
Sequence186-NP-UP-Jp-ABD       KNSDLQKSVKNGVNGTLVGNGLSKTEATTLTKNFSDIRKELNEKLFGNSN
Sequence62-NP-UD-OCC-ABD       KNSELYQSVKNGVNGTLVGNGLSKTEATTLTKNFSDIRKELN--------
Sequence9-NP-GCA-Jp-ABD        KNSDLQKSVKNGVNGTLVGNGLSKTEATTLTKNFSDIRKELNEKLFGNSN
Sequence12-NP-UP-Jp-ABD        KNSDLQKSVKNGVNGTLVGNGLSKTEATTLTKNFSDIRKELNEKLFGNSN
Sequence35-NP-GA-Jp-ABABD      KNSDLQKSVKNGVNGTLVGNGLSKTEATTLTKNFSDIRKELNEKLFGNSN
Sequence54-NP-GA-OCC-ABC       KNSELYQSVKNGVNGTLVGNGLSKAEATTLSKNFSDIRKELN--------
Sequence109-P-CG-Jp-ABD        KNSDLQ-SVRDSVRGTLVGNGLSKTEATKLSKNFSDIRKELSEKLFGK--
Sequence96-NP-UD-Vit-ABD       KNSDLQ-SVRDSVRGTLVGNGLSKTEATKLSKNFSDIRKELSEKLFGKSN
Sequence80-P-CG-EA-ABD         KNSDLQKSVKNGVNGTLVGNGLSKTEATTLTKNFSDIRKELNEKLFGNSN
Sequence82-P-CG-EA-ABD         KNSDLQKSVKNGVNGTLVGNGLSKAEATTLTKTFSDIRKELNEKLFGNSN
Sequence10-NP-GCA-Jp-ABD       KNSDLQKSVKNGVNGTLVGNGLSKTEATTLTKNFSDIRKELNEKLFGNSN
Sequence19-NP-UP-Jp-ABBD       KNSDLQKSVKNGVNGTLVGNGLSKTEATTLTKNFSDIRKELNEKLFGNSN
Sequence115-NP-UG-Jp-ABC       KNSALYQSVKNGVNGTLVGNGLSKAEATTLSKNFSDIKKELNAKL-----
Sequence45-NP-GA-Jp-ABCC       KKSEIYQSVKNGVNGTLVGNGLSGIEATALAKNFSDIKKELNEKF-----
Sequence119-NP-UG-Jp-ABC       KNSALYQSVKNGVNGTLVGNGLSKAEATTLSKNFSDIKKELNAKL-----
Sequence70-NP-GA-Ch-ABD        --------------------------------------------------
Sequence52-NP-UD-EA-ABD        KNSDLQKSVKNGVNGTLVGNGLSKTEATTLTKNFSDIRKELNEKLFGNSN
Sequence97-P-CG-Vit-ABD        KNSDLQ-SVRDSVRGTLVGNGLSKTEATKLSKNFSEIRKELSEKLFGK--
Sequence94-NP-UD-Vit-ABD       KNSDLQ-SVRDSVRGTLVGNGLSKTEATKLSKNFSDIRKELSEKLFGKS-
Sequence176-NP-GA-Col-ABCC     --------------------------------------------------
Sequence68-NP-D-Am-ABBC        KKSEIYQSVKNGVNGTLVGNGLSKAEATTLSKSFSDIKKELNAKLGNFN-
Sequence47-NP-GA-Jp-ABD        KNSDLQKSVKNGVNGTLVGNGLSKTEATTLTKNFSDIRKELNEKLFGNSN
```

```
Sequence56-NP-UD-OCC-ABC      K N S K L Y Q S V K N G V N G T L V G N G L S G I E A T A L A K K F S D I K K E L N - - - - - - - -
Sequence31-NP-GA-Jp-ABD       K N S D L Q K S V K N G V N G T L V G N G L S K T E A T T L T K N F S D I R K E L N E K L F G N S N
Sequence46-NP-UD-Jp-ABD       K N S D L Q K S V K N G V N G T L V G N G L S K T E A T T L T K N F S D I R K E L N E K L F G N S -
Sequence102-NP-UG-Vit-ABD     K N S D L Q - S V R D S V R G T L V G N G L S K T E A T K L S K N F S D I R K E L S E K L F G - - -
Sequence71-P-CG-EA-ABD        K N S D L Q K S V K N G V N G T L V G N G L S K T E A T T L T K N F S D I R K E L N E K L F G N S N
Sequence30-NP-GA-Jp-ABBD      K N S D L Q K S V K N S V N G T L V G N G L P K T E A T K L T K N F S D I R K E L N E K L F - - - -
Sequence28-NP-GA-Jp-AABD      K N S D L Q K S V K N G V N G T L V G N G L S K T E A T T L T K N F S D I R K E L N E K L F G N S N
Sequence98-P-CG-Vit-AD        R N S D L Q - S V R D S V R G T L V G N G L S K T E A T Q L S Q N F S D I R K E L S E K L F G - - -
Sequence99-P-CG-Vit-ABD       K N S D L Q - S V R D S V R G T L V G N G L S K T E A T K L S K N F S D I R K E L N E K L F G N S N
Sequence24-NP-UD-USA-ABD      K N S D L Q K S V K N G V N G T L V G N G L S K T E A T T L T K N F S D I R K E L N E K L F G N S N
Sequence124-NP-G-Jp-ABC       K K S E I Y Q S V K N G V N G I L V G N G L S G I E A T A L T K N F S D I K K E L N E K F - - - - -
Sequence77-P-CG-OCC-ABCC      K N S E L Y Q S V K N G V N G T L V G N G L S K A E A T T L S K N F S D I K K E L N A K - - - - - -
Sequence113-NP-UG-Jp-ABC      K K S E I Y Q S V K N G V N G T L V G N G L S K A E A T T L S K N F S D I K K E L N A K L - - - - -
Sequence88-NP-UD-Vit-ABD      K N S D L Q - F V R D S V R G T L V G N G L S K T E A T K L S K N F S D I R K E L S - - - - - - - -
Sequence100-NP-UD-Vit-ABD     K N S D L Q - S V R D S V R G T L V G N G L S K T E A T K L S K N F S D I R K E L S E K L F G K S N
Sequence137-NP-G-Col-ABCC     K K S E I Y Q S V K N G V N R T L V G N G L S Q A E A T T L S K N F S D I K K E L N A K L - - - - -
Sequence117-NP-G-Jp-ABC       K N S A L Y Q S V K N G V N G T L V G N G L S K A E A T T L S K N F S D I K K E L N A K L - - - - -
Sequence171-NP-UD-Col-ABCC    K K S E I Y Q S V K N G V N G T L V G N G L S Q A E - - - - - - - - - - - - - - - - - - - - - - - -
Sequence180-P-CG-Col-ABC      K K S E I Y Q S V K N G V N G T L V G N G L S Q A E A T T - - - - - - - - - - - - - - - - - - - - -
Sequence112-P-CG-Jp-ABCC      K N S A L Y Q S V K N G V N G T L V G N G L S K A E A T T L S K N F S D I K K E L N A K L - - - - -
Sequence127-NP-UD-Jp-ABC      K K S E I Y Q S V K N G V N G T L V G N G L S Q A E A T T L S K N F S D I K K E L N A K L - - - - -
Sequence51-NP-GA-OCC-ABC      K N S E L Y Q S V K N G V N G T L V G N G L S K A E A T T L S K N F S D I K K E L N - - - - - - - -
Sequence73-P-CG-EA-ABD        K N S D L Q K S V K N G V N G T L V G N G L S K T E A T T L T K N F S D I R K E L N E K L F G N S N
Sequence183-NP-UD-Col-ABD     K K S E I Y Q S V K N G V N G T L V G N G L S G I E A T A L A K N F S D I K K E L N - - - - - - - -
Sequence34-NP-UD-Jp-ABDD      K K S E I Y Q S V K N G V N G T L V G N G L S Q A E A T T L S K N F S D I K K E L N A K L - - - - -
Sequence21-P-CG-Jp-ABD        K N S D L Q K S V K N G I N G T L V G N G L S K T E A T T L T K N F S D I R K E L N E K L F G N S N
Sequence32-NP-GA-Jp-ABD       K N S D L Q K S V K N G V N G T L V G N G L S K T E A T T L T K N F S D I R K E L N E K L S G N S N
Sequence116-NP-UD-Jp-ABC      K N S A L Y Q S V K N G V N G T L V G N G L S K A E A T T L S K N F S D I K K E L N A K L - - - - -
Sequence8-NP-GCA-Jp-ABD       K N S D L Q K S V K N G V N G T L V G N G L S K T E A T T L T K N F S D I R K E L N E K L F G N S N
Sequence83-P-CG-EA-ABD        K N S D L Q K S V K N G V N G T L V G N G L S K T E A T T L T K N F S D I R K E L N E K L F G N S N
Sequence48-NP-UD-OCC-ABC      K N S E L Y Q S V K N G V N K T L V G N G L S Q A E A T T L S K N F S D I K K E L N - - - - - - - -
Sequence120-NP-G-Jp-ABC       K K S E I Y Q F V K N G V N G S L V G N G L S Q A E A T T L S K N F S D I K K E L N A K L - - - - -
Sequence187-NP-UG-Jp-ABCCC    K K S E I Y Q S V K N G V N G T L V G N G L S G I E A T A L A K N F S D I K K E L N E K F - - - - -
Sequence118-NP-G-Jp-ABD       K N S D L Q K S V K N G V N G T L V G N G L S K T E A T T L T K N F S D I R K E L N E K L F G N S N
Sequence87-NP-UG-Vit-ABD      K N S D L Q - S V R D S V R G T L V G N G L S K T E A T K L S K N F S D I R K E L S - - - - - - - -
```

```
                              901                912
Sequence140-NP-GA-Col-ABABC   - - - - - - - - - - -
Sequence123-NP-UD-Jp-ABD      N N N N G L K N N T E
Sequence111-NP-G-Jp-ABD       - - - - - - - - - - -
Sequence57-NP-GA-OCC-AB       - - - - - - - - - - -
Sequence172-P-CG-Col-ABCC     - - - - - - - - - - -
Sequence110-NP-UD-Jp-ABD      N N N N - - - - - - -
Sequence95-NP-UD-Vit-ABD      S - - - - - - - - - -
Sequence107-P-CG-Vit-ABD      - - - - - - - - - - -
Sequence106-P-CG-Vit-ABD      - - - - - - - - - - -
Sequence105-NP-UD-Vit-ABD     - - - - - - - - - - -
Sequence126-NP-UG-Jp-ABC      - - - - - - - - - - -
Sequence135-P-CG-Col-ABCCC    - - - - - - - - - - -
Sequence138-NP-GA-Col-ABC     - - - - - - - - - - -
Sequence13-NP-UP-Jp-ABD       N N N N G F K - - - -
Sequence11-NP-UP-Jp-ABD       N N N - - - - - - - -
Sequence50-NP-GA-OCC-ABC      - - - - - - - - - - -
Sequence26-NP-GA-Jp-ABD       N N N N G L K - - - -
Sequence55-NP-UD-OCC-ABCCC    - - - - - - - - - - -
Sequence178-NP-M-Col-ABCC     - - - - - - - - - - -
Sequence40-NP-GA-Jp-ABD       N N S N G L K - - - -
Sequence43-NP-GA-Jp-ABD       N N N N G L K - - - -
Sequence139-NP-UD-Col-ABCC    - - - - - - - - - - -
Sequence7-P-CG-Jp-ABBD        N N N - - - - - - - -
Sequence91-P-CG-Vit-ABD       N N N - - - - - - - -
```

| | | | | | | | | | | | |
|---|---|---|---|---|---|---|---|---|---|---|---|
| Sequence29-NP-GA-Jp-ABABD | N | N | N | - | - | - | - | - | - | - | - |
| Sequence78-P-CG-EA-ABD | N | N | N | N | G | L | K | N | S | - | - |
| Sequence188-NP-GCA-Jp-AB | - | - | - | - | - | - | - | - | - | - | - |
| Sequence72-P-CG-EA-ABD | N | N | N | N | G | L | K | - | - | - | - |
| Sequence79-P-CG-EA-ABD | N | N | N | N | G | L | K | N | N | - | - |
| Sequence61-NP-UD-OCC-ABC | - | - | - | - | - | - | - | - | - | - | - |
| Sequence125-NP-UG-Jp-ABCC | - | - | - | - | - | - | - | - | - | - | - |
| Sequence175-NP-G-Col-ABCC | - | - | - | - | - | - | - | - | - | - | - |
| Sequence122-NP-G-Jp-ABCC | - | - | - | - | - | - | - | - | - | - | - |
| Sequence36-NP-GA-Jp-ABD | N | N | N | N | G | L | K | - | - | - | - |
| Sequence58-NP-UD-OCC-ABC | - | - | - | - | - | - | - | - | - | - | - |
| Sequence27-NP-GA-Jp-ABBD | N | N | N | - | - | - | - | - | - | - | - |
| Sequence93-P-CG-Vit-ABD | - | - | - | - | - | - | - | - | - | - | - |
| Sequence177-NP-M-Col-ABCC | - | - | - | - | - | - | - | - | - | - | - |
| Sequence182-NP-UD-Col-ABCC | - | - | - | - | - | - | - | - | - | - | - |
| Sequence114-NP-UG-Jp-ABC | - | - | - | - | - | - | - | - | - | - | - |
| Sequence74-P-CG-EA-ABD | N | N | N | N | G | L | K | N | S | - | - |
| Sequence20-P-CG-Jp-ABD | N | N | N | N | G | L | K | - | - | - | - |
| Sequence14-NP-GCA-Jp-ABD | N | N | N | - | - | - | - | - | - | - | - |
| Sequence39-NP-GA-Jp-ABC | - | - | - | - | - | - | - | - | - | - | - |
| Sequence170-NP-UD-Col-ABCC | - | - | - | - | - | - | - | - | - | - | - |
| Sequence76-P-CG-EA-ABD | N | N | N | - | - | - | - | - | - | - | - |
| Sequence53-NP-GA-EA-ABD | N | N | N | N | G | L | K | - | - | - | - |
| Sequence174-P-CG-Col-ABCC | - | - | - | - | - | - | - | - | - | - | - |
| Sequence33-NP-UG-Jp-ABD | - | - | - | - | - | - | - | - | - | - | - |
| Sequence60-NP-GA-EA-ABD | N | N | N | - | - | - | - | - | - | - | - |
| Sequence38-NP-GA-Jp-ABD | N | N | N | - | - | - | - | - | - | - | - |
| Sequence1-NP-EG-Jp-ABCCC | - | - | - | - | - | - | - | - | - | - | - |
| Sequence101-P-CG-Vit-ABD | - | - | - | - | - | - | - | - | - | - | - |
| Sequence64-NP-UD-OCC-ABC | - | - | - | - | - | - | - | - | - | - | - |
| Sequence81-P-CG-EA-ABD | N | N | N | - | - | - | - | - | - | - | - |
| Sequence150-NP-DA-Sen-BC | - | - | - | - | - | - | - | - | - | - | - |
| Sequence90-NP-UD-Vit-ABD | S | N | - | - | - | - | - | - | - | - | - |
| Sequence136-P-CG-Col-AACC | - | - | - | - | - | - | - | - | - | - | - |
| Sequence15-NP-UP-Jp-ABD | N | N | N | - | - | - | - | - | - | - | - |
| Sequence108-NP-UD-Vit-ABD | - | - | - | - | - | - | - | - | - | - | - |
| Sequence104-P-CG-OCC-ABCC | - | - | - | - | - | - | - | - | - | - | - |
| Sequence179-NP-UD-Col-ABCC | - | - | - | - | - | - | - | - | - | - | - |
| Sequence37-NP-GA-Jp-ABCCC | - | - | - | - | - | - | - | - | - | - | - |
| Sequence89-NP-UD-Vit-ABD | - | - | - | - | - | - | - | - | - | - | - |
| Sequence92-P-CG-Vit-ABD | N | N | N | - | - | - | - | - | - | - | - |
| Sequence49-NP-GA-Jp-ABC | - | - | - | - | - | - | - | - | - | - | - |
| Sequence181-P-CG-Col-ABCC | - | - | - | - | - | - | - | - | - | - | - |
| Sequence25-NP-GA-Jp-ABD | N | N | N | - | - | - | - | - | - | - | - |
| Sequence4-P-CG-Jp-ABD | N | N | N | N | G | L | K | - | - | - | - |
| Sequence121-NP-G-Jp-ABCC | - | - | - | - | - | - | - | - | - | - | - |
| 4dvy | - | - | - | - | - | - | - | - | - | - | - |
| Sequence186-NP-UP-Jp-ABD | N | N | N | N | G | L | K | N | N | - | - |
| Sequence62-NP-UD-OCC-ABD | - | - | - | - | - | - | - | - | - | - | - |
| Sequence9-NP-GCA-Jp-ABD | N | N | N | - | - | - | - | - | - | - | - |
| Sequence12-NP-UP-Jp-ABD | N | N | N | N | G | L | K | - | - | - | - |
| Sequence35-NP-GA-Jp-ABABD | N | N | N | - | - | - | - | - | - | - | - |
| Sequence54-NP-GA-OCC-ABC | - | - | - | - | - | - | - | - | - | - | - |
| Sequence109-P-CG-Jp-ABD | - | - | - | - | - | - | - | - | - | - | - |
| Sequence96-NP-UD-Vit-ABD | - | - | - | - | - | - | - | - | - | - | - |
| Sequence80-P-CG-EA-ABD | N | N | N | - | - | - | - | - | - | - | - |
| Sequence82-P-CG-EA-ABD | N | N | N | - | - | - | - | - | - | - | - |
| Sequence10-NP-GCA-Jp-ABD | N | N | N | N | G | L | K | - | - | - | - |
| Sequence19-NP-UP-Jp-ABBD | N | N | N | N | G | L | K | N | - | - | - |
| Sequence115-NP-UG-Jp-ABC | - | - | - | - | - | - | - | - | - | - | - |
| Sequence45-NP-GA-Jp-ABCC | - | - | - | - | - | - | - | - | - | - | - |

| | | | | | | | | | | | |
|---|---|---|---|---|---|---|---|---|---|---|---|
| Sequence119-NP-UG-Jp-ABC | - | - | - | - | - | - | - | - | - | - | - |
| Sequence70-NP-GA-Ch-ABD | - | - | - | - | - | - | - | - | - | - | - |
| Sequence52-NP-UD-EA-ABD | N | N | N | N | G | L | K | - | - | - | - |
| Sequence97-P-CG-Vit-ABD | - | - | - | - | - | - | - | - | - | - | - |
| Sequence94-NP-UD-Vit-ABD | - | - | - | - | - | - | - | - | - | - | - |
| Sequence176-NP-GA-Col-ABCC | - | - | - | - | - | - | - | - | - | - | - |
| Sequence68-NP-D-Am-ABBC | - | - | - | - | - | - | - | - | - | - | - |
| Sequence47-NP-GA-Jp-ABD | N | N | N | - | - | - | - | - | - | - | - |
| Sequence56-NP-UD-OCC-ABC | - | - | - | - | - | - | - | - | - | - | - |
| Sequence31-NP-GA-Jp-ABD | N | N | N | N | G | L | K | N | S | - | - |
| Sequence46-NP-UD-Jp-ABD | - | - | - | - | - | - | - | - | - | - | - |
| Sequence102-NP-UG-Vit-ABD | - | - | - | - | - | - | - | - | - | - | - |
| Sequence71-P-CG-EA-ABD | N | N | N | N | G | L | K | - | - | - | - |
| Sequence30-NP-GA-Jp-ABBD | - | - | - | - | - | - | - | - | - | - | - |
| Sequence28-NP-GA-Jp-AABD | N | N | N | - | - | - | - | - | - | - | - |
| Sequence98-P-CG-Vit-AD | - | - | - | - | - | - | - | - | - | - | - |
| Sequence99-P-CG-Vit-ABD | - | - | - | - | - | - | - | - | - | - | - |
| Sequence24-NP-UD-USA-ABD | N | N | N | - | - | - | - | - | - | - | - |
| Sequence124-NP-G-Jp-ABC | - | - | - | - | - | - | - | - | - | - | - |
| Sequence77-P-CG-OCC-ABCC | - | - | - | - | - | - | - | - | - | - | - |
| Sequence113-NP-UG-Jp-ABC | - | - | - | - | - | - | - | - | - | - | - |
| Sequence88-NP-UD-Vit-ABD | - | - | - | - | - | - | - | - | - | - | - |
| Sequence100-NP-UD-Vit-ABD | S | N | G | L | - | - | - | - | - | - | - |
| Sequence137-NP-G-Col-ABCC | - | - | - | - | - | - | - | - | - | - | - |
| Sequence117-NP-G-Jp-ABC | - | - | - | - | - | - | - | - | - | - | - |
| Sequence171-NP-UD-Col-ABCC | - | - | - | - | - | - | - | - | - | - | - |
| Sequence180-P-CG-Col-ABC | - | - | - | - | - | - | - | - | - | - | - |
| Sequence112-P-CG-Jp-ABCC | - | - | - | - | - | - | - | - | - | - | - |
| Sequence127-NP-UD-Jp-ABC | - | - | - | - | - | - | - | - | - | - | - |
| Sequence51-NP-GA-OCC-ABC | - | - | - | - | - | - | - | - | - | - | - |
| Sequence73-P-CG-EA-ABD | N | N | N | - | - | - | - | - | - | - | - |
| Sequence183-NP-UD-Col-ABD | - | - | - | - | - | - | - | - | - | - | - |
| Sequence34-NP-UD-Jp-ABDD | - | - | - | - | - | - | - | - | - | - | - |
| Sequence21-P-CG-Jp-ABD | N | N | N | N | G | L | K | - | - | - | - |
| Sequence32-NP-GA-Jp-ABD | N | N | - | - | - | - | - | - | - | - | - |
| Sequence116-NP-UD-Jp-ABC | - | - | - | - | - | - | - | - | - | - | - |
| Sequence8-NP-GCA-Jp-ABD | N | N | N | - | - | - | - | - | - | - | - |
| Sequence83-P-CG-EA-ABD | N | N | N | N | G | L | K | - | - | - | - |
| Sequence48-NP-UD-OCC-ABC | - | - | - | - | - | - | - | - | - | - | - |
| Sequence120-NP-G-Jp-ABC | - | - | - | - | - | - | - | - | - | - | - |
| Sequence187-NP-UG-Jp-ABCCC | - | - | - | - | - | - | - | - | - | - | - |
| Sequence118-NP-G-Jp-ABD | N | N | N | - | - | - | - | - | - | - | - |
| Sequence87-NP-UG-Vit-ABD | - | - | - | - | - | - | - | - | - | - | - |

1 2 3 4 5 6 7 8 9

Variable Average Conserved

X - Insufficient data - the calculation for this site was performed on less than 10% of the sequences.
